# Electrochemical Synthesis of C(sp^3^)-Rich Heterocycles *via* Mesolytic Cleavage of Anodically Generated Aromatic Radical Cations

**DOI:** 10.1021/acs.orglett.4c03091

**Published:** 2024-10-21

**Authors:** Hussain A. Maashi, Abdulrahman H. Husayni, Kharou M, Michael E. Reid, James Harnedy, Ethan C. Herneman, Marc Pera-Titus, Louis C. Morrill

**Affiliations:** † Cardiff Catalysis Institute, School of Chemistry, Cardiff University, Main Building Park Place, Cardiff, CF10 3AT, United Kingdom; ∇ Department of Chemistry, College of Science, University of Bisha, Bisha 61922, Saudi Arabia; § Department of Chemistry, University of Bath, Claverton Down, Bath, BA2 7AY, United Kingdom; # Department of Chemistry, College of Science, Jazan University, Jizan 45142, Saudi Arabia

## Abstract

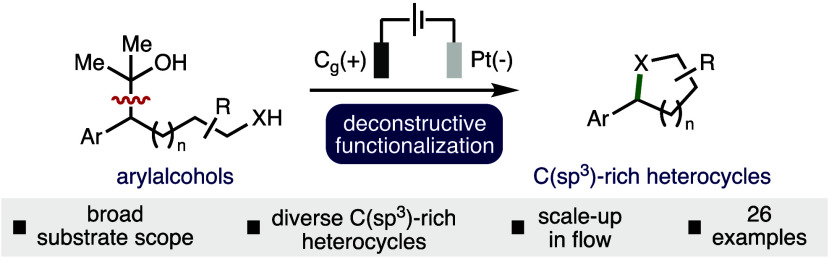

Herein we report an electrochemical deconstructive functionalization approach for the synthesis of C(sp^3^)-rich heterocycles. The reaction proceeds *via* the mesolytic cleavage of anodically generated aromatic radical cations and the trapping of formed carbocation intermediates with internal nucleophiles. The method has been demonstrated across various arylalcohol substrates to access a diverse range of C(sp^3^)-rich heterocycles including tetrahydrofuran, tetrahydropyran, and pyrrolidine scaffolds (26 examples). The electrochemical method was demonstrated on a 5 mmol scale *via* single pass continuous flow, which utilized lower supporting electrolyte concentration and exhibited increased productivity in relation to the batch process.

Electrochemistry can be utilized to selectively oxidize or reduce organic molecules.^1^ Through control of various electrochemical parameters,^2^ specific single electron transfer processes can be targeted, which provide access to a diverse array of synthetically versatile radical intermediates.^3^ Oxidation of aromatic systems to the corresponding aromatic radical cation results in the weakening of β-C–C σ-bonds present within the molecule (Scheme 1A).^4,5^ This intriguing, yet somewhat underutilized, mode of substrate activation has been employed in the development of electrosynthetic methodologies,^6^ including the deconstructive functionalization of arylcyclopropanes,^7^ donor–acceptor cyclopropanes/cyclobutanes,^8^ and 5-, 6- and 7-membered arylcycloalkanes.^9^ In this area, our group recently reported an electrochemical method for the deconstructive functionalization of unstrained arylcycloalkanols,^10^ where various alcohols, carboxylic acids, and N-heterocycles were employed as external nucleophiles to generate a diverse array of synthetically useful remotely functionalized ketones (Scheme 1B).^11^

**Scheme 1:**
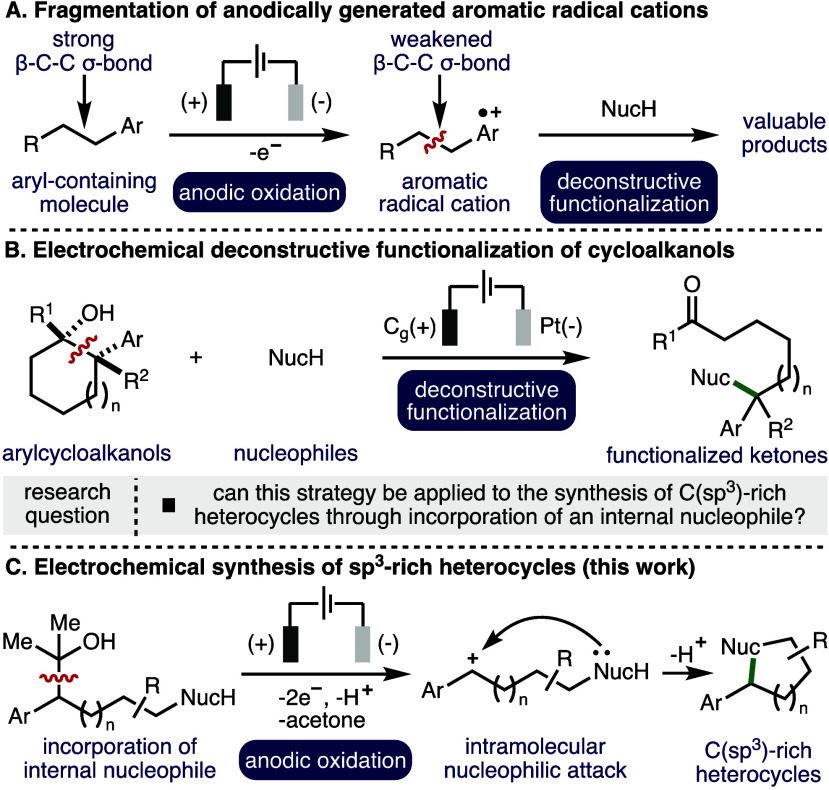
Background and Context

More than 85% of all biologically active chemical entities contain a heterocycle,^12^ which highlights their importance in the development of new pharmaceuticals. Saturated heterocycles can offer further advantages such as improved aqueous solubility and lower toxicity of metabolites, while increasing the level of saturation (C(sp^3^)-rich) and structural diversity in drug discovery programmes.^13^ Building upon our previous work, it was envisaged that the electrochemical deconstructive functionalization^14^ strategy could be applied to the synthesis of C(sp^3^)-rich heterocycles through incorporation of an internal nucleophile.^15^

Herein, we report the successful realization of this strategy, which enables the electrochemical synthesis of various heterocycles,^16^ including substituted tetrahydrofuran, tetrahydropyran, and pyrrolidine scaffolds (26 examples) (Scheme 1C).

The electrochemical conversion of 2-arylalcohol **1** (E_p/2_ = 1.64 V vs Fc/Fc^+^) to form 2-phenyltetrahydrofuran (**2**) was selected as the model system for reaction optimization due to facile determination of conversion data *via*^1^H NMR analysis of crude reaction mixtures (Table 1).^17^ The optimized electrochemical reaction conditions employed *n*-Bu_4_NClO_4_ as the supporting electrolyte in DCM:TFE (19:1, [**1**] = 0.05 M), galvanostatic electrolysis (*i* = 7.5 mA, *j*_anode_ = 5.9 mA/cm^2^, 2 *F*), a graphite anode and a Pt foil cathode in an undivided cell at 25 °C under N_2_, which gave 90% conversion to **2** (87% isolated yield) (Table 1, entry 1). 2-Arylalcohol **1** was prepared in one step from lactone **3***via* reaction with MeLi (2.5 equiv.). As such, a formal two-step carbonyl deletion sequence from lactone **3** to tetrahydrofuran **2** has been achieved. A Faradaic efficiency of 90% indicated that most of the electricity passing through the cell is utilized productively. No product formation or quantitative recovery of **1** was observed in the absence of electricity (entry 2). Employing a constant cell potential (*E*_cell_ = 7 V) resulted in only 67% conversion to **2** after 2 *F* of charge was passed (entry 3). Alterations to the current applied (*i* = 5 or 10 mA) lowered the yield of **2** (entry 4), as did variation of electrode materials (entries 5 and 6), electrolyte (entry 7), electrolyte/substrate concentration (entries 8 and 9), solvent mixture (entries 10 and 11), and the amount of charge passed (entry 12). When DCM was replaced by MeCN in the solvent mixture (entry 10), a high cell potential and anode fouling was observed, which may be explained by DCM being reduced at the cathode, acting as an electron sink. It was also found that employing MeOH as cosolvent, which is more nucleophilic and less acidic than TFE, resulted in lower conversion to **2** (entry 11). An experiment that involved lowering the concentration of supporting electrolyte to 0.025 M was halted due to the high cell potential observed.

**Table 1:**
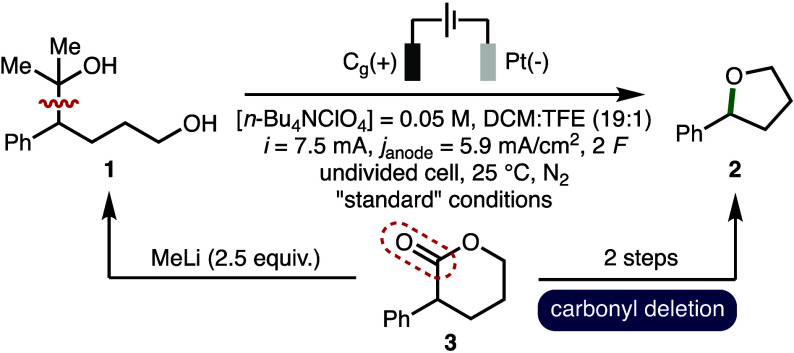
Optimization of the Electrochemical Process^a^

entry	variation from “standard” conditions	yield^b^ (%)
**1**	**none**	**90 (87)**
2	no electricity	<2
3	*E*_cell_ = 7 V	67
4	*i* = 5 mA or 10 mA	69, 80
5	Graphite as cathode	70
6	Pt foil as anode	<2
7	*n*-Bu_4_NBF_4_ or *n*-Bu_4_NPF_6_ as electrolyte	75, 67
8	[*n*-Bu_4_NClO_4_] = 0.1 or 0.025 M	81, N.D.
9	[**1**] = 0.033 or 0.1 M	84,^c^ 64^d^
10	MeCN:TFE (19:1) as solvent	<2
11	DCM:MeOH (19:1) as solvent	52
12	1.5 *F* or 2.5 *F*	60, 73

a Reactions performed with 0.3 mmol of **1** using the ElectraSyn 2.0 batch electrochemical reactor. [**1**] = 0.05 M.

b As determined by^1^H NMR analysis of the crude reaction mixture with 1,3,5-trimethylbenzene as the internal standard. Isolated yield given in parentheses. N.D. = not determined.

c 1 (0.2 mmol).

d **1** (0.6 mmol).

With optimized electrochemical reaction conditions in hand, the scope and limitations of the heterocycle formation were investigated (Scheme 2). Initially, it was found that a variety of substituents and functional groups were tolerated on the aromatic ring present within the 2-arylalcohol substrates, which enabled access to the corresponding 2-aryl substituted tetrahydrofuran products in high isolated yields (products **4**–**10** and **13**–**17**). These included halogens (4-F, 4-Cl, 4-Br, 4-I), electron-releasing groups (e.g., 4-OMe, 4-OTBS), aryl (e.g., 4-Ph), and alkyl substituents (e.g., 4-*t*-Bu). A substrate that contained a phenol motif was insoluble and did not result in any observable conversion to the desired tetrahydrofuran product **11**, whereas a 2-arylalcohol that contained an electron-withdrawing aromatic substituent (4-CF_3_) gave product **12** in a modest 33% yield. This latter observation may be attributed to the higher oxidation potential of the substrate (no observable oxidation in the 0–2.5 V vs Fc/Fc^+^ potential window). 2-Arylalcohol substrates that contained *o*-tolyl, mesityl, or 1-naphthyl substituents were converted into the corresponding 2-aryl tetrahydrofurans **17**–**19** in 55–83% isolated yields, which demonstrated that heterocycle formation was not particularly sensitive toward increased steric encumbrance on the aromatic ring. Additional heterocycles could be incorporated into the tetrahydrofuran products, including cyclic acetal (**20**), 2-thiophenyl (**21**), and 2-furanyl (**22**) motifs. 2,4-Disubstituted tetrahydrofuran **23** was formed as a 1.4:1 mixture of diastereoisomers, which were isolated in a combined 81% yield. 2,2-Disubstituted tetrahydrofuran products **24** and **25** were formed in 85% and 71% yields, respectively, where **25** was derived from the nonsteroidal anti-inflammatory drug, ibuprofen. Next, the impact of chain length upon successful heterocycle formation was investigated. While the electrosynthetic protocol was optimized for the formation of 5-membered rings (e.g., tetrahydrofuran **2**), it was found that 2-phenyltetrahydro-2H-pyran **27** could also be isolated in 41% yield. However, the electrosynthetic method was not applicable to the formation of 4-membered rings (e.g., 2-phenyloxetane **26**) or 7-membered rings (e.g., 2-phenyloxepane **28**). Finally, substituting the internal hydroxyl nucleophile for a sulfonamide enabled the formation of 2-phenyl-1-tosylpyrrolidine (**29**) in 53% isolated yield. A complex mixture of products was observed upon the attempted formation of 2-phenyltetrahydrothiophene (**30**) using the optimized reaction conditions, which may be attributed to undesired reactivity resulting from oxidation of the sulfur atom.

**Scheme 2:**
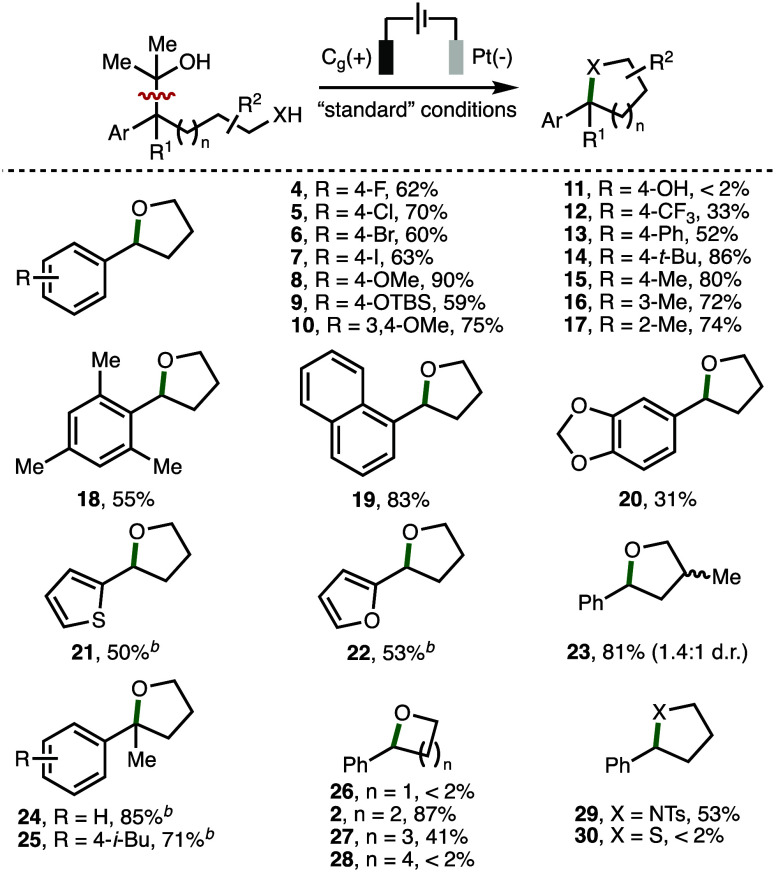
Scope and Limitations (2-Arylalcohols) Reactions performed using optimized reaction conditions (Table 1, entry 1) with isolated yields after chromatographic purification quoted unless stated otherwise. As determined by ^1^H NMR analysis of the crude reaction mixture with 1,3,5-trimethylbenzene or 1,3,5-trimethoxybenzene as the internal standard.

Next, two 1-arylalcohol substrates were synthesized and subjected to the optimized electrochemical reaction conditions (Scheme 3). 2-Methyltetrahydrofuran (**31**) and 2,2-dimethyltetrahydrofuran (**32**) were formed in 46% and 36% NMR yields, respectively, which confirmed that nonaromatic substituents could be incorporated at the 2-position within the tetrahydrofuran products.

**Scheme 3:**
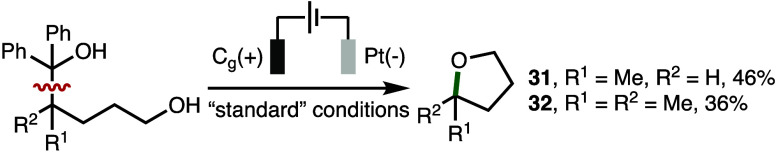
Further Substrate Scope (1-Arylalcohols) Reactions performed using optimized reaction conditions (Table 1, entry 1). Yields as determined by ^1^H NMR analysis of the crude reaction mixture with 1,3,5-trimethylbenzene as the internal standard.

To demonstrate scalability, the electrochemical formation of 2-phenyltetrahydrofuran (**2**) was performed in flow employing a syringe pump (flow rate = 2 mL/min) in combination with the commercially available Ammonite8 flow electroreactor (volume = 1 mL)^18^ equipped with a carbon anode and platinum plate cathode (Scheme 4). Using galvanostatic electrolysis (*i* = 320 mA, *j*_anode_ = 14.0 mA/cm^2^, 2 *F*), 2-Arylalcohol **1** (5 mmol) was converted to **2** in 83% isolated yield (0.62 g) in a continuous single pass. In comparison to batch, the flow process was performed using a lower electrolyte concentration ([*n*-Bu_4_NClO_4_] = 0.025 M vs [*n*-Bu_4_NClO_4_] = 0.05 M) and increased current density (*j*_anode_ = 16 mA/cm^2^ vs *j*_anode_ = 5.9 mA/cm^2^), which resulted in higher productivity (4.98 mmol/h vs 0.12 mmol/h).

**Scheme 4:**
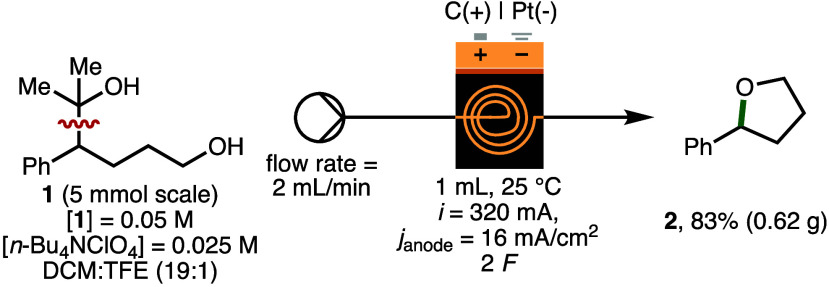
Electrochemical Scale up in Flow

A selection of experiments were performed to gain insight into the reaction mechanism (Scheme 5). First, it was found that aliphatic alcohol **33**, which does not undergo any observable oxidation in the 0–2.5 V vs Fc/Fc^+^ potential window, was unreactive when subjected to the optimized electrochemical reaction conditions (Scheme 5A). Replacing the phenyl group present within substrate **1** with a homobenzyl motif (substrate **34**) also resulted in no observable conversion to the corresponding tetrahydrofuran product **36**. Taken together, these results indicate that (i) a 1- or 2-arylalcohol structural motif is required for successful heterocycle formation (cf., Schemes 3 and 4); (ii) the reaction proceeds *via* an initial oxidation of the aromatic ring to form an aromatic radical cation; and (iii) alkoxy radical intermediates are not involved in the reaction mechanism. Next, we investigated the impact of the deconstructive functionalization strategy on the reaction efficiency (Scheme 5B). When 4-phenylbutan-1-ol (**37**) (E_p/2_ = 1.82 V vs Fc/Fc^+^) was subjected to the optimized electrochemical reaction conditions, only 20% conversion to 2-phenyltetrahydrofuran (**2**) was observed alongside 70% unreacted **37**.^19^ Furthermore, it was found that a selection of related substrates (**38**–**40**) that contained various aromatic substituents (4-F, 4-OMe, and 4-CF_3_) underwent no observable conversion to the corresponding tetrahydrofuran products. As such, it was clear that the deconstructive functionalization strategy employed facilitated the electrochemical heterocycle formation. Finally, it was found that subjecting (*S*)-**1** (>99% e.e.) to the electrochemical reaction conditions produced 2-phenyltetrahydrofuran (**2**) in racemic form (Scheme 5C), which confirmed the involvement of a planar benzylic secondary carbocation intermediate in the reaction mechanism. Taking the formation of product **2** as a representative example, and based upon related studies,^6−11^ a plausible reaction mechanism initiates with single electron anodic oxidation of the phenyl ring within the 2-arylalcohol substrate to give the corresponding aromatic radical cation (Scheme 5D). This species can be converted to the corresponding benzylic carbocation *via* hydroxyl-assisted mesolytic cleavage of the weakened benzylic β-C–C σ-bond and single-electron anodic oxidation, while generating acetone as an innocent byproduct. Subsequent intramolecular nucleophilic attack by the hydroxyl group and deprotonation generates the observed tetrahydrofuran products. The counter cathodic reaction is hydrogen gas production *via* proton reduction.

**Scheme 5:**
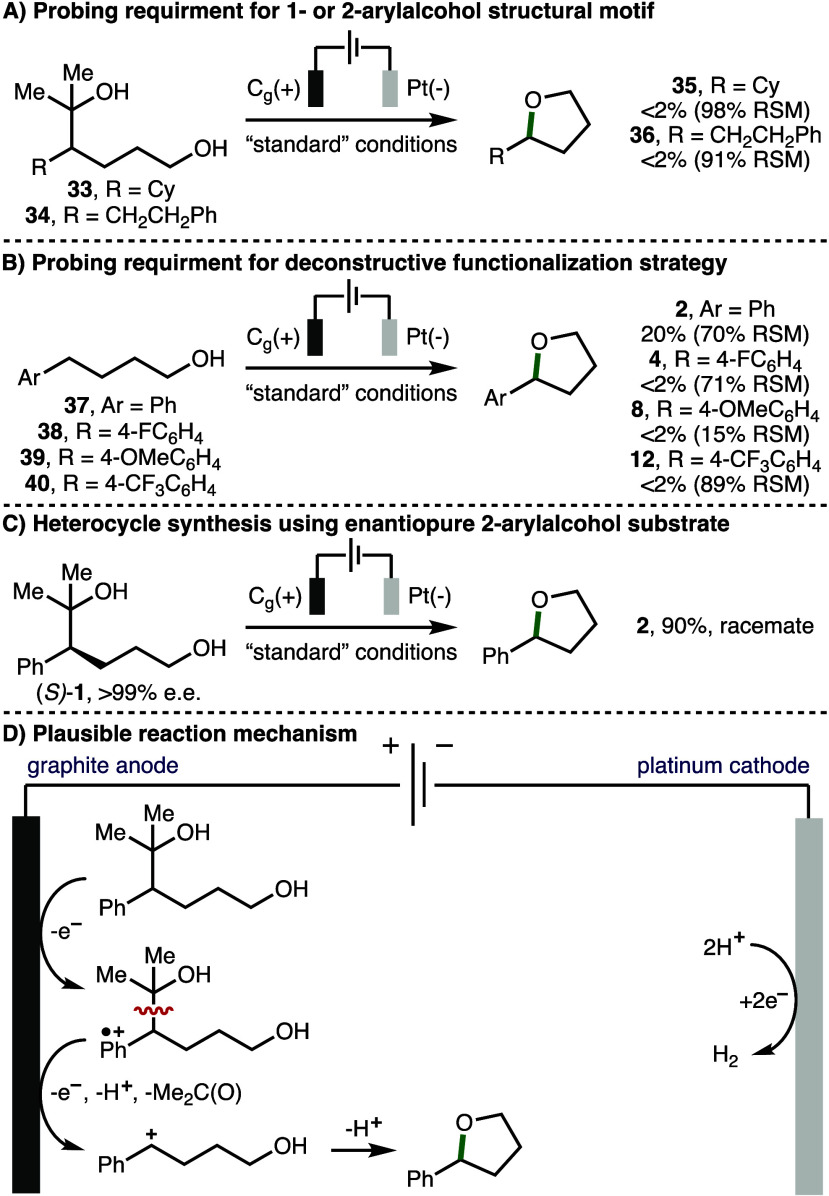
Reaction Mechanism Reactions performed using optimized reaction conditions (Table 1, entry 1). Yields as determined by ^1^H NMR analysis of the crude reaction mixture with 1,3,5-trimethylbenzene as the internal standard. RSM = returned starting material.

In summary, an electrochemical deconstructive functionalization strategy has been employed to access various C(sp^3^)-rich heterocyclic products from readily accessible arylalcohol substrates (26 examples). The reaction proceeds *via* the mesolytic cleavage of anodically generated aromatic radical cations and trapping of carbocation intermediates with internal nucleophiles. The method was demonstrated on a 5 mmol scale *via* single pass continuous flow, which exhibited increased productivity in relation to the batch process. Ongoing work in our laboratory is focused on developing further applications of the mesolytic cleavage of anodically generated aromatic radical cations in organic synthesis.

## Data Availability

The data underlying this study are available in the published letter, in its Supporting Information, and openly available in the Cardiff University data catalogue at: 10.17035/cardiff.26362525.

## Supporting Information Available

Optimization data, experimental procedures, characterization of new compounds and spectral data (PDF)
