# Supplementary material for: Electrochemical Synthesis of C(sp3)-Rich Heterocycles via Mesolytic Cleavage of Anodically Generated Aromatic Radical Cations
Source: Org Lett. 2024 Oct 21;26(42):9051–5. doi: 10.1021/acs.orglett.4c03091 (PMC11519923; doi:10.1021/acs.orglett.4c03091)
Supplement: Supplementary file 1 — ol4c03091_si_001.pdf [file ol4c03091_si_001.pdf]

SUPPORTING INFORMATION

Electrochemical Synthesis of C(sp^3)-Rich Heterocycles *via* Mesolytic Cleavage of Anodically Generated Aromatic Radical Cations

Hussain A. Maashi,[†, ∇] Abdulrahman H. Husayni,[†] Kharou M,[†] Michael E. Reid,[†] James Harnedy,[†] Ethan C. Herneman,[†] Marc Pera-Titus,[†] and Louis C. Morrill*[,†,§]

[†] Cardiff Catalysis Institute, School of Chemistry, Cardiff University, Main Building, Park Place, Cardiff, CF10 3AT, United Kingdom.

[∇] Department of Chemistry, College of Science, University of Bisha, Bisha 61922, Saudi Arabia.

[§] Department of Chemistry, University of Bath, Claverton Down, Bath, BA2 7AY, United Kingdom.

*Email: Lcm71@bath.ac.uk

Contents

General Information 3

General Procedures 6

Substrate Synthesis and Characterisation 10

Characterisation of Products 85

    Electrochemical General Procedure 85

    Faradaic Efficiency 86

Electrochemical Flow Scale Up 140

    General Information 140

    Gram Scale Flow Experimental 141

Cyclic Voltammetry Studies of Selected Substrates 144

Proposed Mechanism 149

References 150

General Information

Unless otherwise stated, all non-electrochemical reactions were conducted in flame-dried glassware under an atmosphere of dry nitrogen, sealed with septum seals and were stirred with Teflon coated magnetic stirrer bars. Unless otherwise stated, all electrochemical reactions were performed using oven-dried 10 mL ElectraSyn vials under an atmosphere of dry nitrogen, sealed with an ElectraSyn Teflon cap fitted with a graphite anode and platinum cathode and were stirred with Teflon coated magnetic stirrer bars. Dry solvents (THF, Et_2O, MeCN and CH_2Cl_2) were obtained after passing these previously degassed solvents through activated alumina columns (Mbraun, SPS-800). Electrolytes (tetra-*n*-butylammonium hexafluorophosphate (n-Bu_4NPF_6), tetra-*n*-butylammonium tetrafluoroborate (n-Bu_4NBF_4) and tetra-*n*-butylammonium perchlorate (n-Bu_4NClO_4) were recrystallised from ethanol or water, and dried in the oven before use. Commercial tetrafluoroethylene (TFE) was used as supplied without further purification. Unless otherwise stated, all other solvents and commercial reagents were used as supplied without further purification.

All electrochemical reactions were conducted using an ElectraSyn 2.0 apparatus, purchased from IKA. Graphite electrodes were used as supplied from IKA or were cut from a sheet of carbon foil (2 mm thickness) purchased from Goodfellow. The electrodes were cut to the dimension of 8 mm × 52 mm using a Startrite Bandsaw (model 18-T-5) with a Starrett, Durate SFB high carbon steel blade (2870 mm x 10 mm x 0.65 mm, 3 mm pitch, regular tooth). Graphite electrodes could be used several times by renewing the top surface of the graphite. This was achieved by scraping away the top layer with a razor blade, sonicating in MeCN for 5 minutes, followed by oven drying for 30 mins. Platinum electrodes were cut from a sheet of platinum foil (0.05 mm thickness) purchased from Goodfellow using scissors to a width of 5 mm and to a standard length of an IKA supplied electrode (52 mm). Platinum electrodes were washed with water and acetone, then burned over a Bunsen burner before every reaction. The electrodes were set up in the standard IKA supplied Electrasyn vial cap with an electrode distance of 7 mm. Reactions were stirred at 400 rpm at room temperature.

Cyclic voltammetry (CV) experiments were conducted at room temperature using an Autolab PGSTAT204, controlled using Nova 2.1 software. The working electrode was a GC disc (3 mm dia., BASi part number MF-2012), the counter electrode was a Pt-wire (BASi part number MW-

4130) and an Ag/AgCl reference electrode was used (BASi part number – MF-2052). The working electrode was polished on a water wet alumina pad in a figure of 8 motion for 30 seconds before being rinsed with deionised water and acetone. The counter electrode was rinsed with deionised water and acetone before being burned over a Bunsen burner. The scans were oxidative, and the oxidative potential window was (0.0 – 2.5 V vs. Fc/Fc^+), then referenced to Fc. The solvent was deoxygenated by bubbling through N_2 for 5 minutes before scan was initiated.

Room temperature (rt) refers to 20-25 °C. Ice/water and CO_2(s)/acetone baths were used to obtain temperatures of 0 °C and -78 °C respectively. All reactions involving heating were conducted using DrySyn blocks and a contact thermometer. *In vacuo* refers to reduced pressure through the use of a rotary evaporator.

Analytical thin layer chromatography (TLC) was carried out using aluminium plates coated with silica (Kieselgel 60 F254 silica) and visualisation was achieved using ultraviolet light (254 nm), followed by staining with a 1% aqueous $KMnO_4$ solution, or a 10% w/v solution of phosphomolybdic acid in ethanol. Flash column chromatography was performed using Kieselgel 60 silica in the solvent system stated using head-pressure by means of a compressed air line.

Melting points were recorded on an a Gallenkamp melting point apparatus and are reported corrected by linear calibration to benzophenone (47 - 49 °C) and benzoic acid (121 - 123 °C). Infrared spectra were recorded on a Shimadzu IRAffinity-1 Fourier Transform ATR spectrometer as thin films using a Pike MIRacle ATR accessory. The most intense peaks and structurally important peaks are quoted. Absorption maxima (ν_{max}) are recorded in wavenumbers (cm^{-1}). ^1H, ^{13}C and ^{19}F NMR spectra were obtained on a Bruker Avance 300 (300 MHz ^1H, 75 MHz ^{13}C), Bruker Avance 400 (400 MHz ^1H, 101 MHz ^{13}C, 376 MHz ^{19}F) or a Bruker Avance 500 (500 MHz ^1H, 126 MHz ^{13}C, 471 MHz ^{19}F) spectrometer at rt in the solvent stated. Chemical shifts are reported in parts per million (ppm) relative to the residual solvent signal. All coupling constants, *J*, are quoted in Hz. Multiplicites are reported with the following symbols: br = broad, s = singlet, d = doublet, t = triplet, q = quartet, m = multiplet and combinations of these were used to denote higher order multiplicities. High resolution mass spectrometry (HRMS, m/z) data was acquired at Cardiff University. EI/CI HRMS data was

collected on a Thermo Scientific Exactive GC machine with an orbitrap mass analyser. ES HRMS data was collected on a Walters Xevo G2XS machine with a TOF (Time of Flight) mass analyser.

HPLC analyses were obtained on a Gilson HPLC consisting of a Gilson 305 pump, Gilson 306 pump, Gilson 811C dynamic mixer, Gilson 805 manometric module, Gilson 401C dilutor, Gilson 213XL sample injector and sample detection was performed with a Gilson 118 UV/vis detector, at 211 nm. Separation was achieved using a Chiralpak IC column.

“Petrol” and ‘’hexanes’’ refers to the fraction boiling in the range of 40-60 ^{o}C unless otherwise stated.

General Procedures

General Procedure A: Synthesis of Aryl Substituted Diols *via* Lactone Ring Opining:

Step 1: Synthesis of 3-Aryl Lactones:

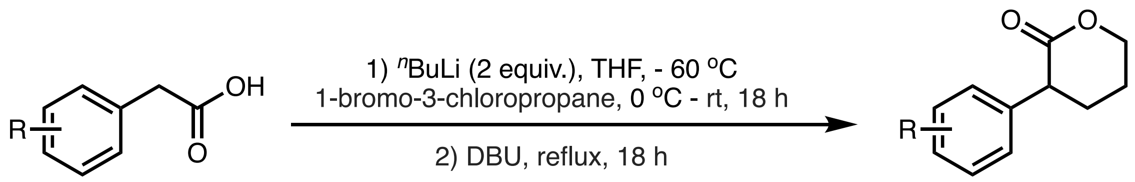

To arylacetic acid (1 equiv.) in THF (0.25 M with respect to arylacetic acid) at -60 °C was added ^{n}BuLi (2.0 equiv.) dropwise under N_2 atmosphere and the temperature was maintained under -40 °C. After complete addition, the resulted mixture was warmed slowly to 0 °C and stirred for 2 hours. After 2 hours, 1-bromo-3-chloropropane (1.2 equiv.) was added. The mixture was warmed to room temperature and left to stir for 18 hours. After 18 hours, the reaction was quenched with 1M NaOH (50 mL), transferred to a separatory funnel and the aqueous layer was collected. The organic layer was extracted with 1M NaOH (50 mL) and the combined aqueous layers were re-acidified with 2M HCl (70 mL), extracted with EtOAc (75 mL), washed with water (50 mL). The organic layer was collected and dried over $MgSO_4$, filtered and concentrated *in vacuo* to obtain an oil. The resulting oil was re-dissolved in THF (0.5 M with respect to arylacetic acid). DBU (1 equiv.) was added, and the mixture was refluxed for 18 hours. After 18 hours, the resulted slurry was cooled down to room temperature, filtered through a suction funnel and the mixture was concentrated *in vacuo* affording crude 3-aryl lactone. The crude residue was used for the next step without further purification.

Step 2: Lactone Ring Opining with Commercial Methyllithium or Methylmagnesium bromide:

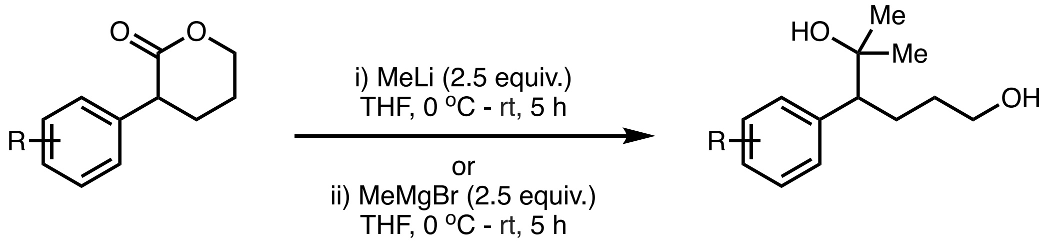

i: To crude 3-aryl lactone (1 equiv.) in THF (0.5 M with respect to 3-aryl lactone) at -30 °C was added commercial methyllithium solution (2.5 equiv.) dropwise. The reaction mixture was allowed to warm to room temperature and left to stir overnight. The mixture was quenched with saturated NH_4Cl solution and then extracted with EtOAc (x 2). The organics were combined, dried over $MgSO_4$, filtered and concentrated *in vacuo* yielding crude aryl substituted diol. The crude residue was purified by flash column chromatography (eluent = EtOAc in hexanes, silica gel) to afford product.

ii: To crude 3-aryl lactone (1 equiv.) in THF (0.5 M with respect to 3-aryl lactone) at 0 °C was added commercial Methylmagnesium bromide solution (2.5 equiv.) dropwise. The reaction mixture was allowed to warm to room temperature and left to stir overnight. The mixture was quenched with saturated NH_4Cl solution and then extracted with EtOAc (x 2). The organics were combined, dried over $MgSO_4$, filtered and concentrated *in vacuo* yielding crude aryl substituted diol. The crude residue was purified by flash column chromatography (eluent = EtOAc in hexanes, silica gel) to afford product.

General Procedure B: Synthesis of Aryl Substituted Diols *via* Hydroboration Oxidation of Olefin Containing 2-Aryl Alkanols:

Step 1: Synthesis of Olefin Containing 2-Aryl Alkanols:

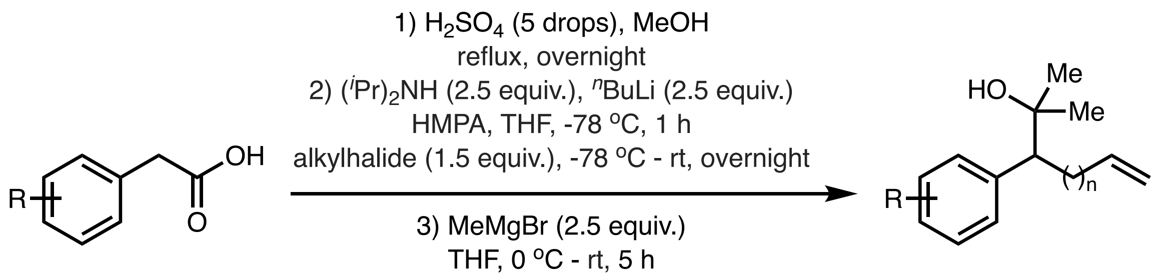

To a solution of aryl acetic acid (1 equiv.) in MeOH was added five drops of concentrated sulfuric acid and the mixture refluxed overnight. The mixture was cooled to room temperature, concentrated *in vacuo,* redissolved in EtOAc, washed with water and brine, dried over $MgSO_4$, filtered and concentrated *in vacuo* to obtain methyl 2-aryl acetate.

To an *in situ* preprepared solution of lithium diisopropylamide solution (1.2 equiv.) in THF (0.9 M) at -78 °C was added a solution of the methyl 2-aryl acetate (1 equiv.) in THF (0.6 M) dropwise. The mixture was stirred for 30 min at -78 °C and warmed up to 0°C. Olefin containing alkyl halide (1.6 equiv.) was added slowly and the mixture was left stirring overnight. The mixture was quenched by adding 2 M hydrochloric acid solution and extracted with EtOAc (3 x), organics combined, washed with brine, dried over $MgSO_4$, filtered and concentrated *in vacuo* yielding crude residue. If required, the crude residue was purified by flash column chromatography (eluent = EtOAc in hexanes, silica gel) to afford product and used in the next step.

To the afforded product (1 equiv.) in THF (0.5 M) at 0 °C was added commercial methylmagnesium bromide solution (2.5 equiv.) dropwise. The reaction mixture was allowed to warm to room temperature and left to stir overnight. The mixture was quenched with saturated NH_4Cl solution and then extracted with EtOAc (x 2). The organics were combined, dried over $MgSO_4$, filtered and concentrated *in vacuo* yielding crude olefin containing 2-aryl alkanols. If required, the crude residue was purified by flash column chromatography (eluent = EtOAc in hexanes, silica gel) to afford product and used in the next step.

Step 2: Hydroboration Oxidation of Olefin Containing 2-Aryl Alkanols:

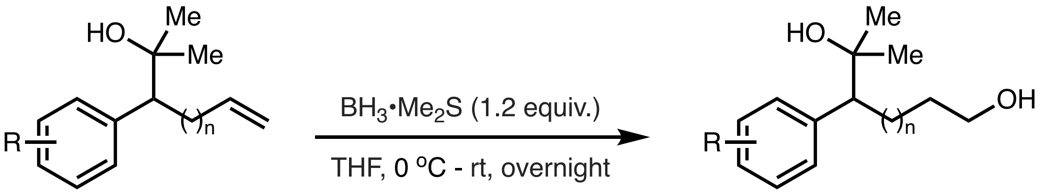

To the solution of the olefin containing 2-aryl alkanols (1 equiv.) in THF (0.5 M with respect to 2-aryl alkanols) was added BH_3•Me_2S solution (1.2 equiv.) at 0°C. The mixture was stirred overnight at room temperature and quenched by sequential addition of water, 3 M NaOH, and 30% H_2O_2. The mixture was extracted with Et_2O (x 3), and the combined organics were dried over $MgSO_4$, filtered and concentrated *in vacuo* yielding crude aryl substituted diol. The crude residue was purified by flash column chromatography (eluent = EtOAc in hexanes, silica gel) to afford product.

General Procedure C: Synthesis of Aryl Substituted Diols *via* Lactone Alkylation and Ring Opining:

Step 1: Lactone Alkylation:

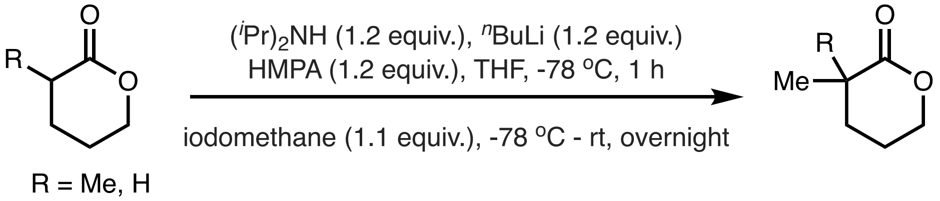

To an *in situ* preprepared solution of Lithium diisopropylamide solution (1.2 equiv.) in THF (0.9 M) at 0 °C was added HMPA (1.2 equiv.) over a period of 10 min. The mixture was cooled down to -78 °C and a solution of the lactone (1 equiv.) in THF (0.25 M with respect to the lactone) was added dropwise. The mixture was stirred for 20 min and iodomethane (1.1 equiv.) was added slowly and the mixture was left stirring for 1 hour. The mixture was quenched with saturated NH_4Cl solution and then extracted with EtOAc (x 2). The organics were combined, washed with brine, dried over $MgSO_4$, filtered and concentrated *in vacuo* yielding crude alkylated lactone. The crude residue was used for the next step without further purification following **modified general procedure A, Step 2** using commercial phenyl magnesium bromide solution (2.5 equiv.).

Substrate Synthesis and Characterisation

(1)

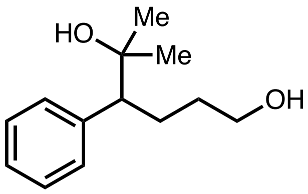

Prepared according to general procedure A, step 2 (i) using 3-phenyltetrahydro-2*H*-pyran-2-one (8.8 g, 50 mmol), THF (100 mL) and methyllithium solution (78 mL, 125 mmol). The crude residue was purified by flash column chromatography (eluent = 30 to 50% EtOAc in hexanes, silica gel) to afford product as white solid (8.65 g, 83% yield) as a racemate.

Mp.: 69-71 °C; **R_f** = 0.16 (eluent = 30% EtOAc in hexanes); **ν_{max} / cm^{-1}** (thin film) 3250 (br), 2980, 2866, 1386; **^{1}H NMR (500 MHz, $CDCl_3$)** δ 7.32 – 7.29 (m, 2H), 7.26 – 7.21 (m, 3H), 3.59 – 3.57 (t, *J* = 6.5 Hz, 2H), 2.62 – 2.59 (dd, *J* = 12.1, 3.2 Hz, 1H), 1.99 – 1.94 (m, 1H), 1.86 – 1.80 (m, 1H), 1.37 – 1.32 (m, 2H), 1.19 – 1.18 (d, *J* = 2.2 Hz, 6H); **^{13}C NMR (126 MHz, $CDCl_3$)** δ 141.1, 129.6, 128.3, 126.8, 72.9, 63.0, 57.1, 31.5, 28.0, 27.9, 25.6; **HRMS (EI^+) *m/z*** $[M - H_2O - CH_3]^+$ Calcd for $C_{12}H_{15}O$ 175.1117; found 175.1115.

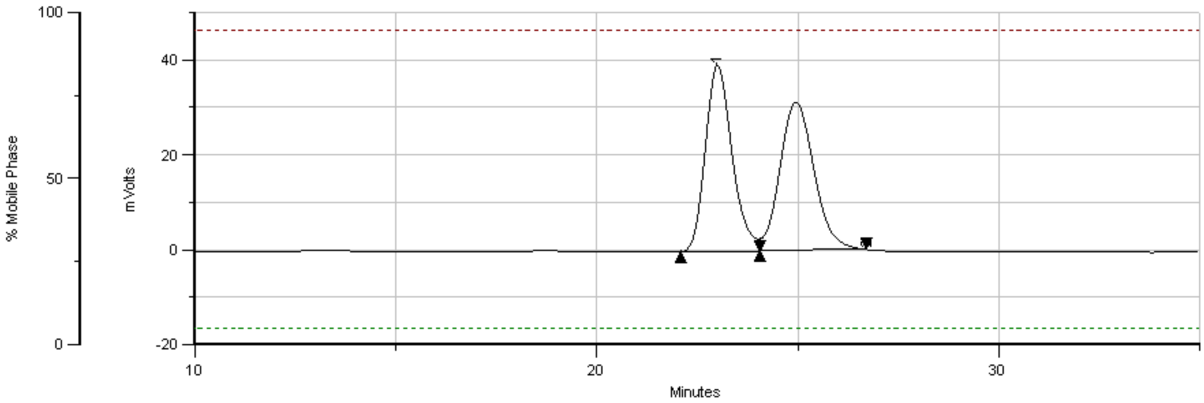

	Inj. Number	Peak Name	R. Time	Area	Area %
1	1.00	*1	23.00	3086400.50	49.78
2	1.00	*2	24.96	3113183.00	50.22

Figure S1. *HPLC trace for racemic **1**. HPLC Chiralpak IC column, 211 nm, 25 °C, 95:5 Hexane/IPA, flow rate 1 mL/min, R = 23.0 min, S = 25.0 min.*

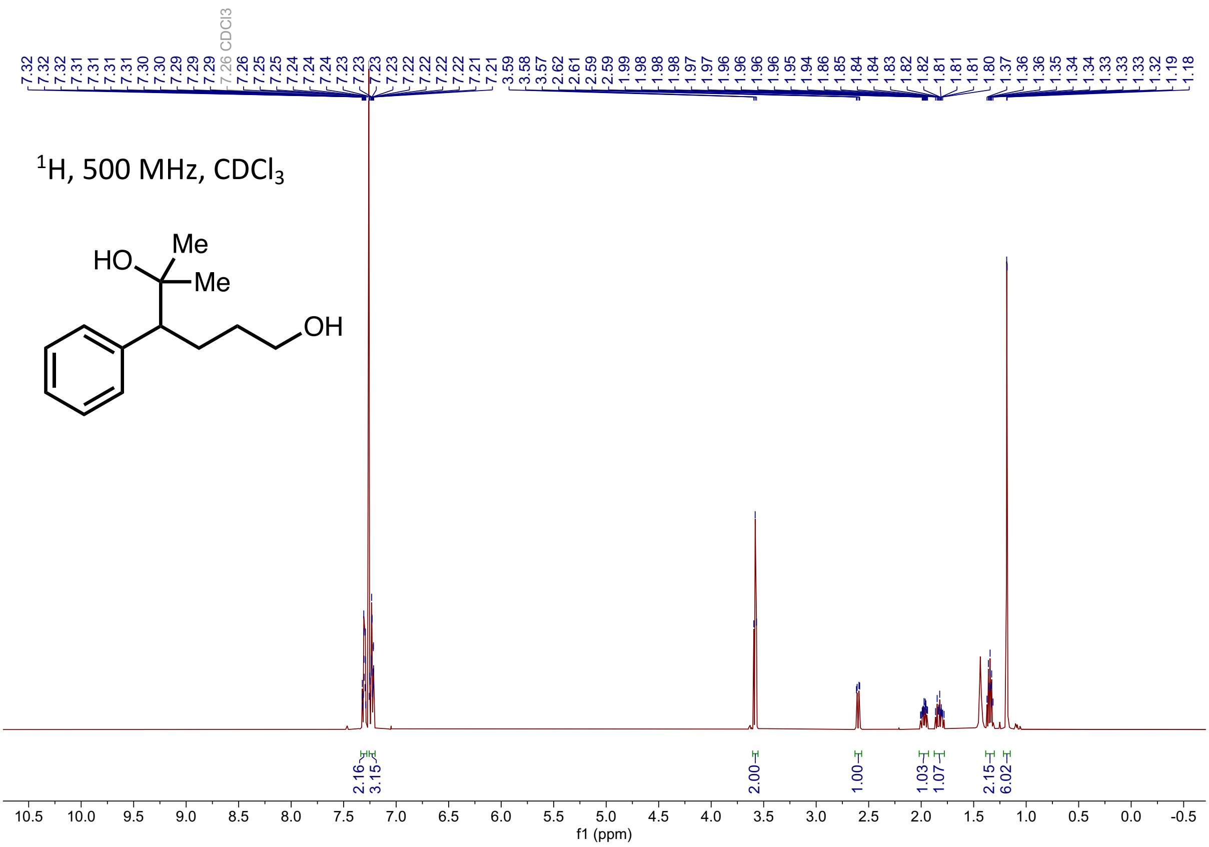

^{1}H, 500 MHz, CDCl$_3$
HO
Me
Me
OH
f1 (ppm)

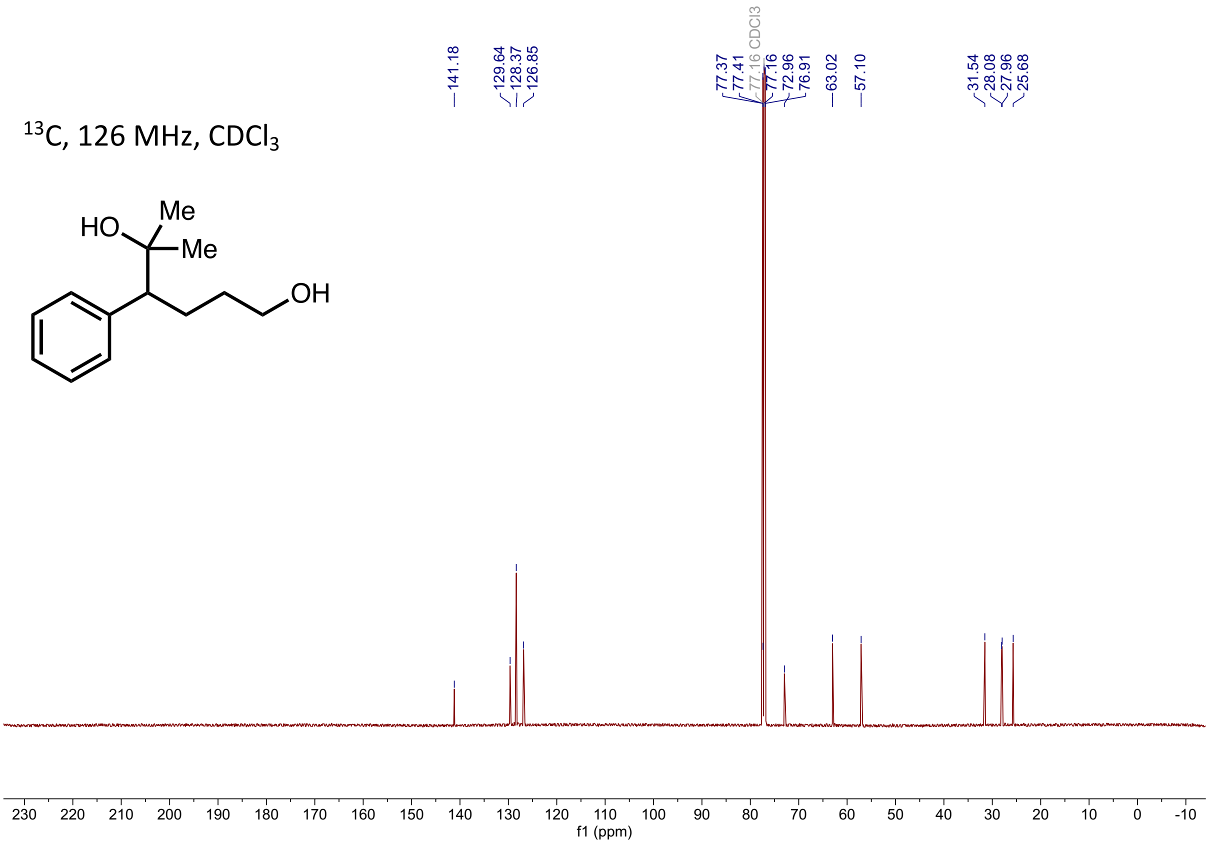

^{13}C, 126 MHz, CDCl$_3$
HO
Me
Me
OH
141.18
129.64
128.37
126.85
77.37
77.41
77.16 CDCl3
77.16
72.96
76.91
63.02
57.10
31.54
28.08
27.96
25.68
f1 (ppm)

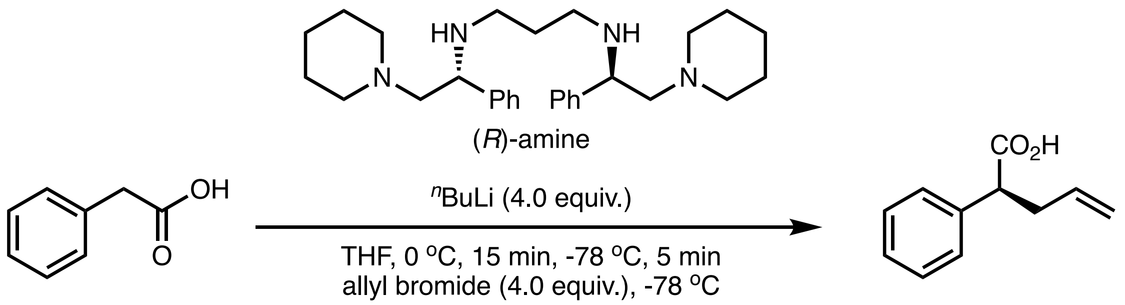

(*S*)-2-phenylpent-4-enoic acid was needed to start the synthesis of (*S*)-1 and it was prepared following literature method.[1]

*ⁿ*BuLi (9.1 ml, 20 mmol) was added dropwise to a solution of phenylacetic acid (680 mg, 5 mmol) and (*R*)-amine* (2.3 g, 5.15 mmol) in THF (33.5 ml, 0.15 M) at 0 °C and the reaction mixture was allowed to stir for 15 min. The reaction mixture was cooled to -78 °C, stirred for an additional 5 min and allyl bromide (1.7 ml, 20 mmol) was added to the reaction mixture dropwise over 10 min. After complete addition, the reaction was immediately quenched with a 3:1 mixture of (THF/MeOH, 40 mL) at -78 °C. After 4 min, 1M aqueous HCl was added, and the reaction mixture was diluted with ethyl acetate and water. The aqueous layer was extracted with EtOAc (x 3). The organics were combined, washed with 1M aqueous HCl and brine, dried over $MgSO_4$, filtered and concentrated *in vacuo* yielding crude acid which was used for the next step without further purification.

*(*R*)-amine was prepared starting from (*R*)-styrene oxide following literature method.[2]

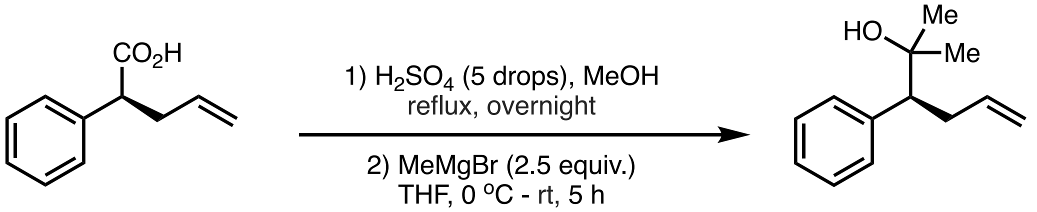

To a solution of (*S*)-2-phenylpent-4-enoic acid in MeOH (50 mL) was added five drops of concentrated sulfuric acid and the mixture refluxed overnight. The mixture was cooled to room temperature, concentrated *in vacuo,* redissolved in EtOAc, washed with water and brine, dried over $MgSO_4$, filtered and concentrated *in vacuo* to obtain crude ester which was used for the next step following general procedure A, step 2 (ii) to obtain (*S*)-2-methyl-3-phenylhex-5-en-2-ol.

(*S*)-(1)

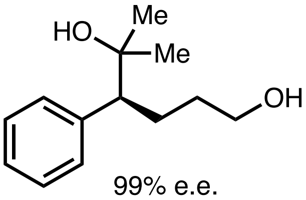

99% e.e.

Prepared according to general procedure B, step 2 using (*S*)-2-methyl-3-phenylhex-5-en-2-ol (305 mg, 1.6 mmol) in THF (5 mL) and BH_3•Me_2S solution (1 mL, 2 mmol, 2.0 M in hexanes). The crude residue was purified by flash column chromatography (eluent = 30 to 50% EtOAc in hexanes, silica gel) to afford product as white solid (200 mg, 60% yield).

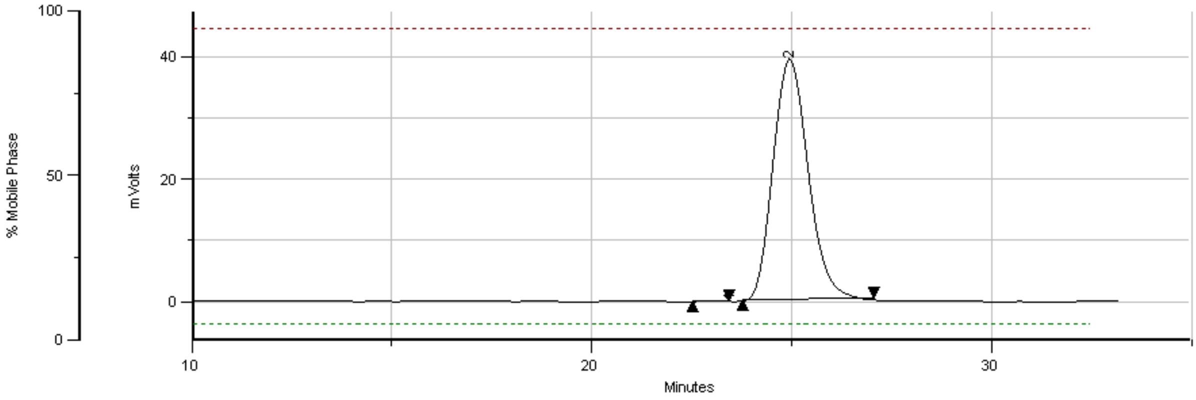

	Inj. Number	Peak Name	R. Time	Area	Area %
1	1.00	*1	22.88	8912.04	0.22
2	1.00	*2	24.97	4040353.00	99.78

Figure S2. *HPLC trace for* ***(S)-1****. HPLC Chiralpak IC column, 211 nm, 25 °C, 95:5 Hexane/IPA, flow rate 1 mL/min, R = 22.9 min S = 25.0 min, e.e. = 99%.*

(3)

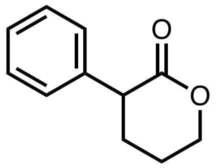

Prepared according to general procedure A, step 1 using phenylacetic acid (13.6 g, 100 mmol), THF (400 mL), ^{n}BuLi (91 mL, 200 mmol, 2.2 M in hexanes), 1-bromo-3-chloropropane (18.9 g, 120 mmol), THF (200 mL) and DBU (15.2 g, 100 mmol). The crude residue was purified by flash column chromatography (eluent = 20 to 30% EtOAc in hexanes, silica gel) to afford product as a pale-yellow oil (14.1 g, 80% yield).

R_f = 0.28 (eluent = 10% EtOAc in hexanes); **^{1}H NMR (500 MHz, $CDCl_3$)** δ 7.37 – 7.33 (m, 2H), 7.30 – 7.26 (m, 1H), 7.25 – 7.23 (m, 2H), 4.46 – 4.42 (m, 2H), 3.79 – 3.76 (dd, *J* = 10.3, 7.2 Hz, 1H), 2.30 – 2.26 (m, 1H), 2.08 – 1.98 (m, 3H); **^{13}C NMR (126 MHz, $CDCl_3$)** δ 172.5, 138.9, 128.7, 128.3, 127.3, 69.3, 47.1, 28.2, 21.9.

Data consistent with the literature.[3]

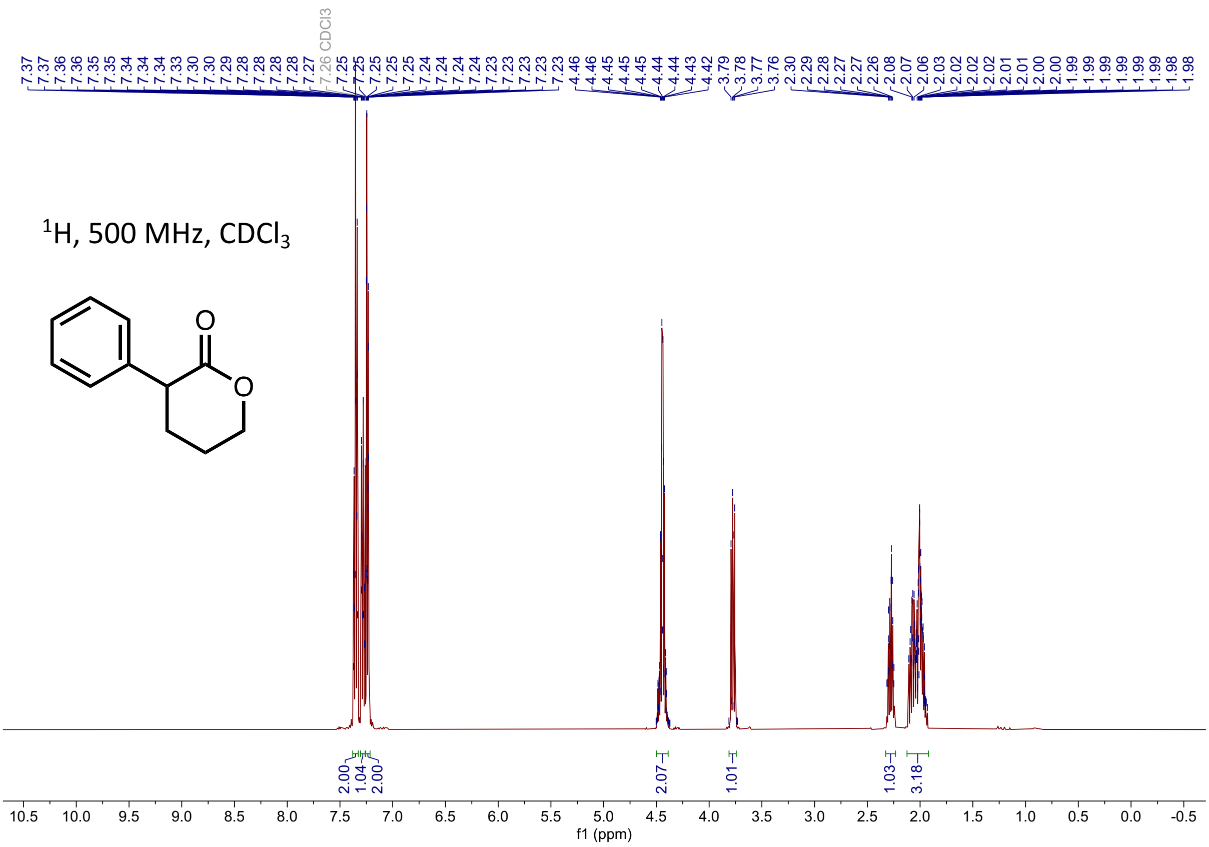

^{1}H, 500 MHz, $CDCl_3$
7.26 CDCl3
2.00
1.04
2.00
2.07
1.01
1.03
3.18
f1 (ppm)

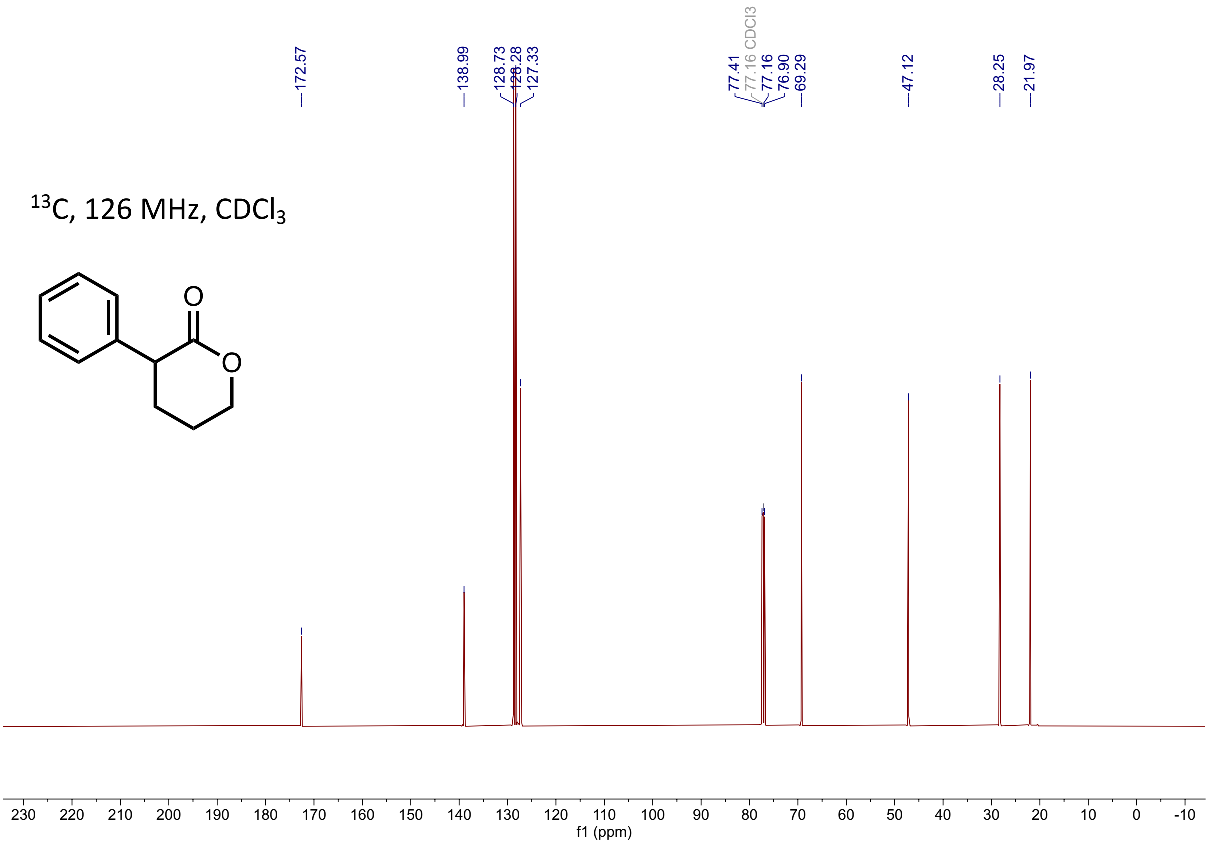

^{13}C, 126 MHz, $CDCl_3$
172.57
138.99
128.73
128.28
127.33
77.41
77.16 CDCl3
76.90
69.29
47.12
28.25
21.97
f1 (ppm)

(S4)

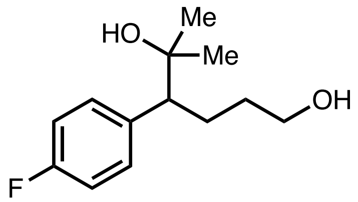

Prepared according to general procedure A, step 2 (i) using 3-(4-fluorophenyl)tetrahydro-2*H*-pyran-2-one (971 mg, 5 mmol), THF (10 mL) and methyllithium solution (7.8 mL, 12.5 mmol). The crude residue was purified by flash column chromatography (eluent = 30 to 50% EtOAc in hexanes, silica gel) to afford product as a yellow solid (611 mg, 54% yield).

Mp.: 74-77 °C; $\mathbf{R_f}$ = 0.18 (eluent = 30% EtOAc in hexanes); $\mathbf{\nu_{max}}$ **/ $\mathbf{cm^{-1}}$** (thin film) 3273 (br), 2982, 2881, 1372; **^{1}H NMR (500 MHz, $CDCl_3$)** δ 7.20 – 7.18 (m, 2H), 7.02 – 6.98 (m, 2H), 3.60 – 3.57 (t, *J* = 6.5 Hz, 2H), 2.60 – 2.57 (dd, *J* = 12.1, 3.2 Hz, 1H), 2.01 – 1.94 (m, 1H), 1.81 – 1.73 (m, 1H), 1.35 – 1.30 (m, 2H), 1.18 – 1.16 (d, 6H); **^{13}C NMR (126 MHz, $CDCl_3$)** δ 160.9, 136.9 (d, *J* = 3.4 Hz), 130.9 (d, *J* = 7.7 Hz), 115.2, 115.0, 72.9, 62.9, 56.3, 31.4, 28.2, 27.9, 25.8; **^{19}F NMR (471 MHz, $CDCl_3$)** δ -116.53; **HRMS (EI^+)** ***m/z*** [M - H_2O - CH_3]$^+$ Calcd for $C_{12}H_{14}FO$ 193.1023; found 193.1020.

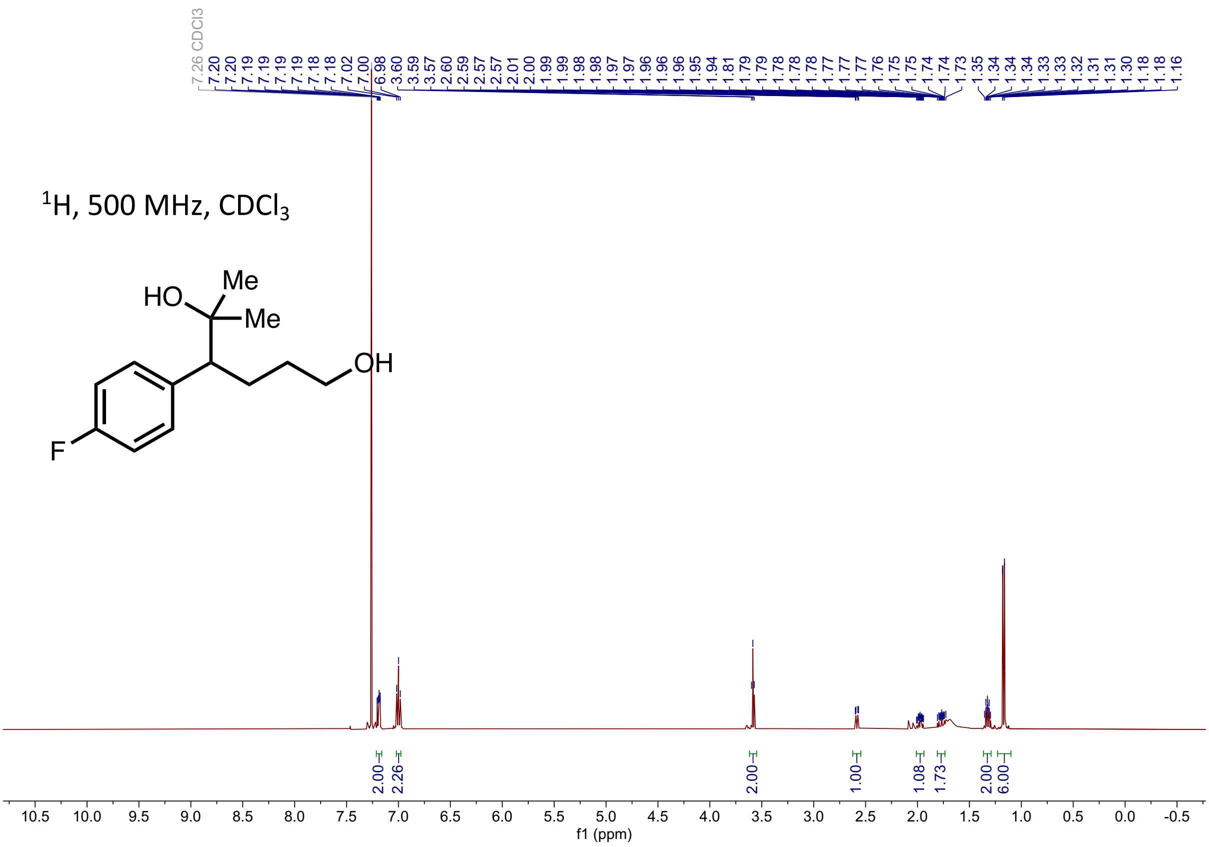
7.26 CDCl3
7.20 7.20 7.19 7.19 7.19 7.19 7.18 7.18 7.02 7.00 6.98 3.60 3.59 3.57 2.60 2.59 2.57 2.57 2.01 2.00 1.99 1.99 1.98 1.98 1.97 1.97 1.96 1.96 1.96 1.95 1.94 1.81 1.79 1.79 1.78 1.78 1.78 1.77 1.77 1.77 1.76 1.75 1.75 1.74 1.74 1.73 1.35 1.34 1.34 1.34 1.33 1.33 1.32 1.31 1.31 1.30 1.18 1.18 1.16
^{1}H, 500 MHz, CDCl$_3$
HO
Me
Me
OH
F
2.00 2.26 2.00 1.00 1.08 1.73 2.00 6.00
10.5 10.0 9.5 9.0 8.5 8.0 7.5 7.0 6.5 6.0 5.5 5.0 4.5 4.0 3.5 3.0 2.5 2.0 1.5 1.0 0.5 0.0 -0.5
f1 (ppm)

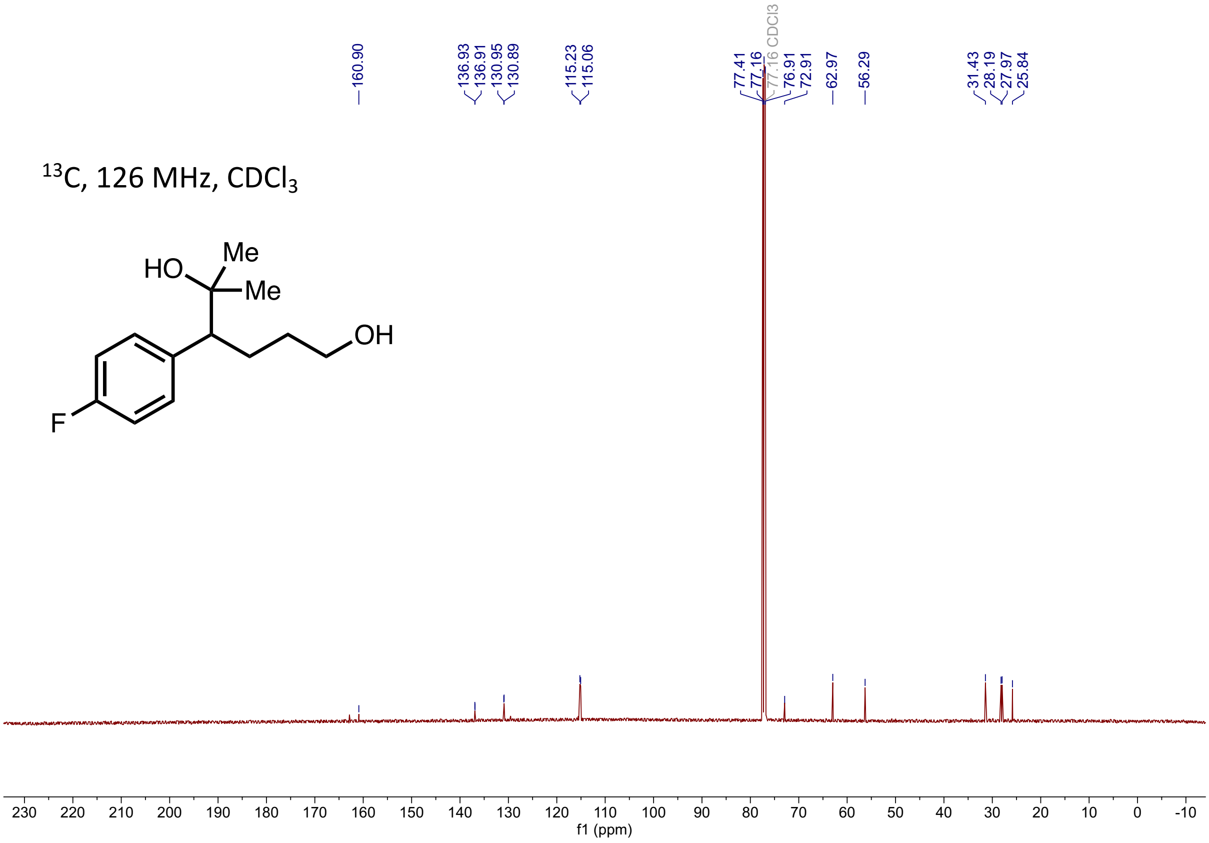
160.90
136.93 136.91 130.95 130.89
115.23 115.06
77.41 77.16 77.16 CDCl3 76.91 72.91
62.97
56.29
31.43 28.19 27.97 25.84
^{13}C, 126 MHz, CDCl$_3$
HO
Me
Me
OH
F
230 220 210 200 190 180 170 160 150 140 130 120 110 100 90 80 70 60 50 40 30 20 10 0 -10
f1 (ppm)

^{19}F, 471 MHz, $CDCl_3$

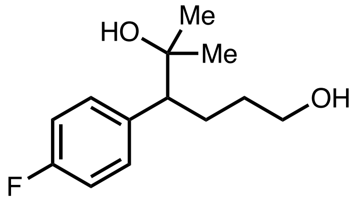

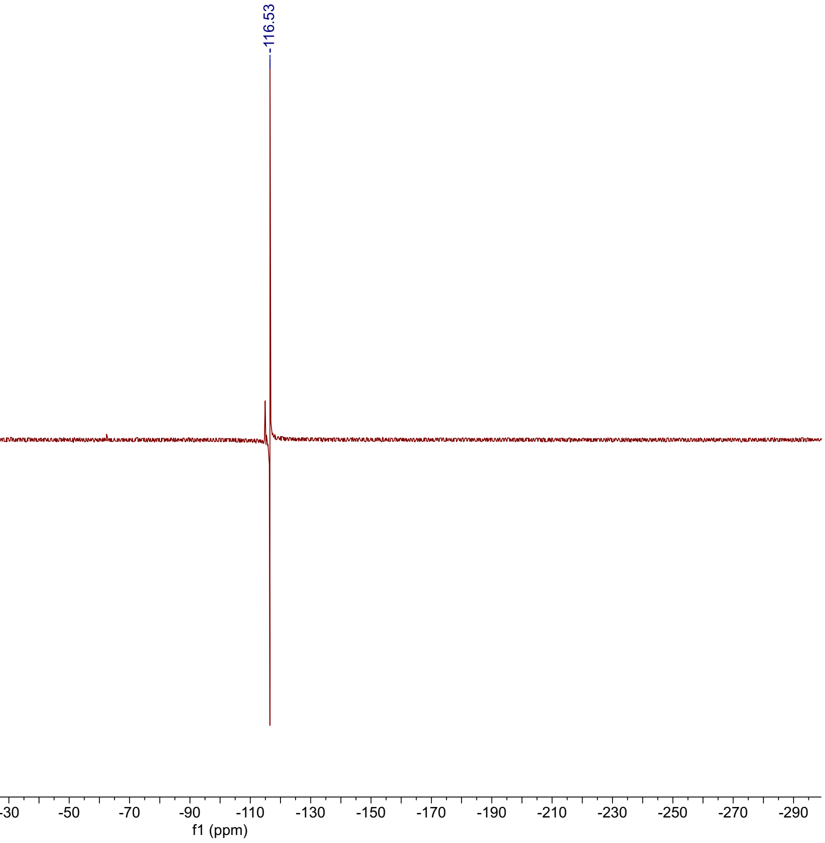

(S5)

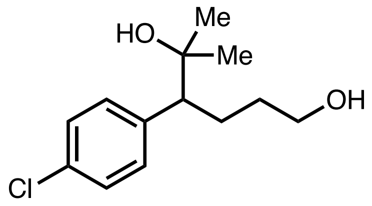

Prepared according to general procedure A, step 2 (i) using 3-(4-chlorophenyl)tetrahydro-2*H*-pyran-2-one (1.05 g, 5 mmol), THF (10 mL) and methyllithium solution (7.8 mL, 12.5 mmol). The crude residue was purified by flash column chromatography (eluent = 30 to 50% EtOAc in hexanes, silica gel) to afford product as a yellow solid (777 mg, 64% yield).

Mp.: 82-85 °C; **R_f** = 0.17 (eluent = 30% EtOAc in hexanes); **ν_{max} / cm^{-1}** (thin film) 3253 (br), 2981, 2881, 1373, 1151; **^{1}H NMR (500 MHz, $CDCl_3$)** δ 7.29 – 7.27 (m, 2H), 7.18 – 7.15 (m, 2H), 3.59 – 3.57 (t, *J* = 6.4 Hz, 2H), 2.59 – 2.56 (dd, *J* = 12.1, 3.2 Hz, 1H), 2.01 – 1.95 (m, 1H), 1.80 – 1.74 (m, 1H), 1.35 – 1.29 (m, 2H), 1.18 – 1.16 (d, 6H); **^{13}C NMR (126 MHz, $CDCl_3$)** δ 139.9, 132.6, 130.9, 128.5, 72.9, 62.9, 56.4, 31.4, 28.3, 27.9, 25.7; **HRMS (EI^+) *m/z*** [M - H_2O - CH_3]$^+$ Calcd for $C_{12}H_{14}ClO$ 209.0727; found 209.0726.

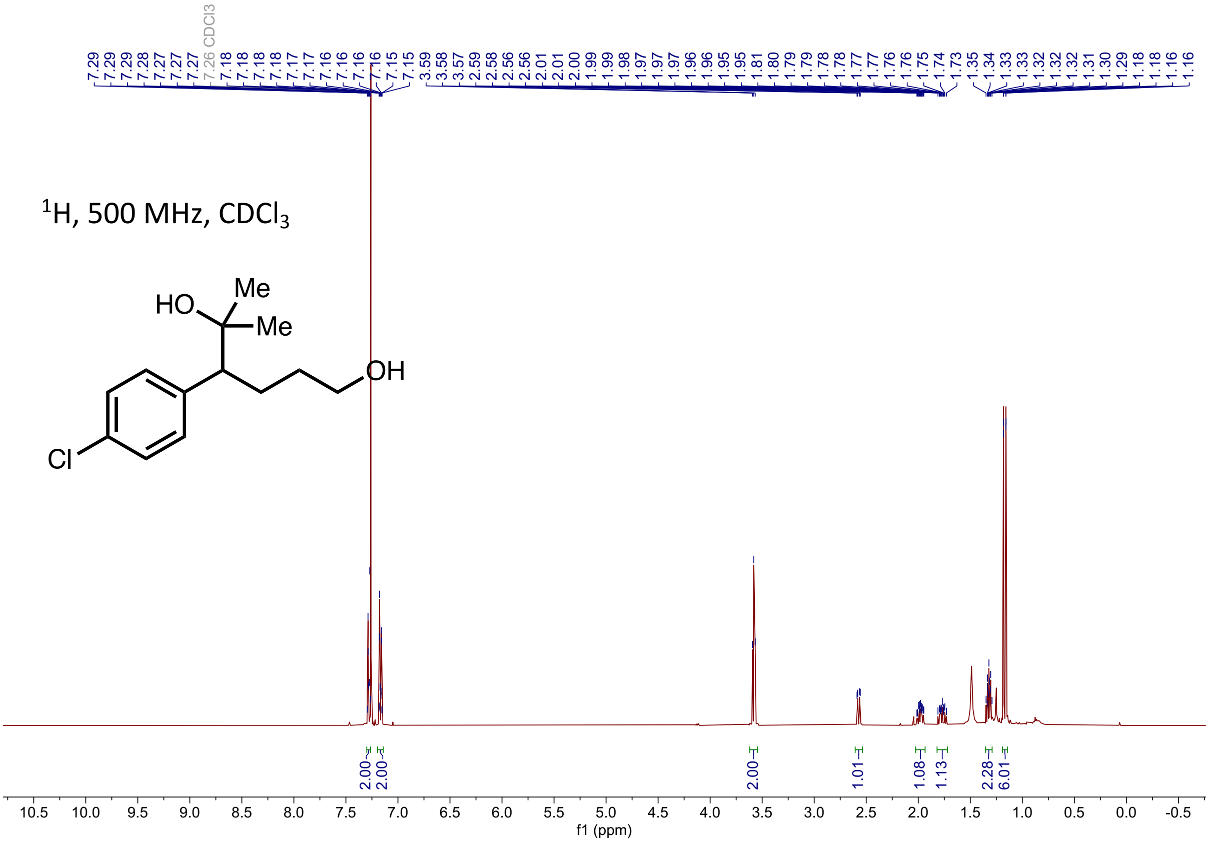
7.29 7.29 7.29 7.28 7.27 7.27 7.27 7.26 CDCl3 7.18 7.18 7.18 7.18 7.17 7.17 7.16 7.16 7.16 7.16 7.15 7.15 3.59 3.58 3.57 2.59 2.58 2.56 2.56 2.01 2.01 2.00 1.99 1.99 1.98 1.97 1.97 1.97 1.96 1.96 1.95 1.95 1.81 1.80 1.79 1.79 1.78 1.78 1.77 1.77 1.76 1.76 1.75 1.74 1.73 1.35 1.34 1.33 1.33 1.32 1.32 1.32 1.31 1.30 1.29 1.18 1.18 1.16 1.16
^{1}H, 500 MHz, CDCl$_3$
HO
Me
Me
OH
Cl
2.00
2.00
2.00
1.01
1.08
1.13
2.28
6.01
f1 (ppm)

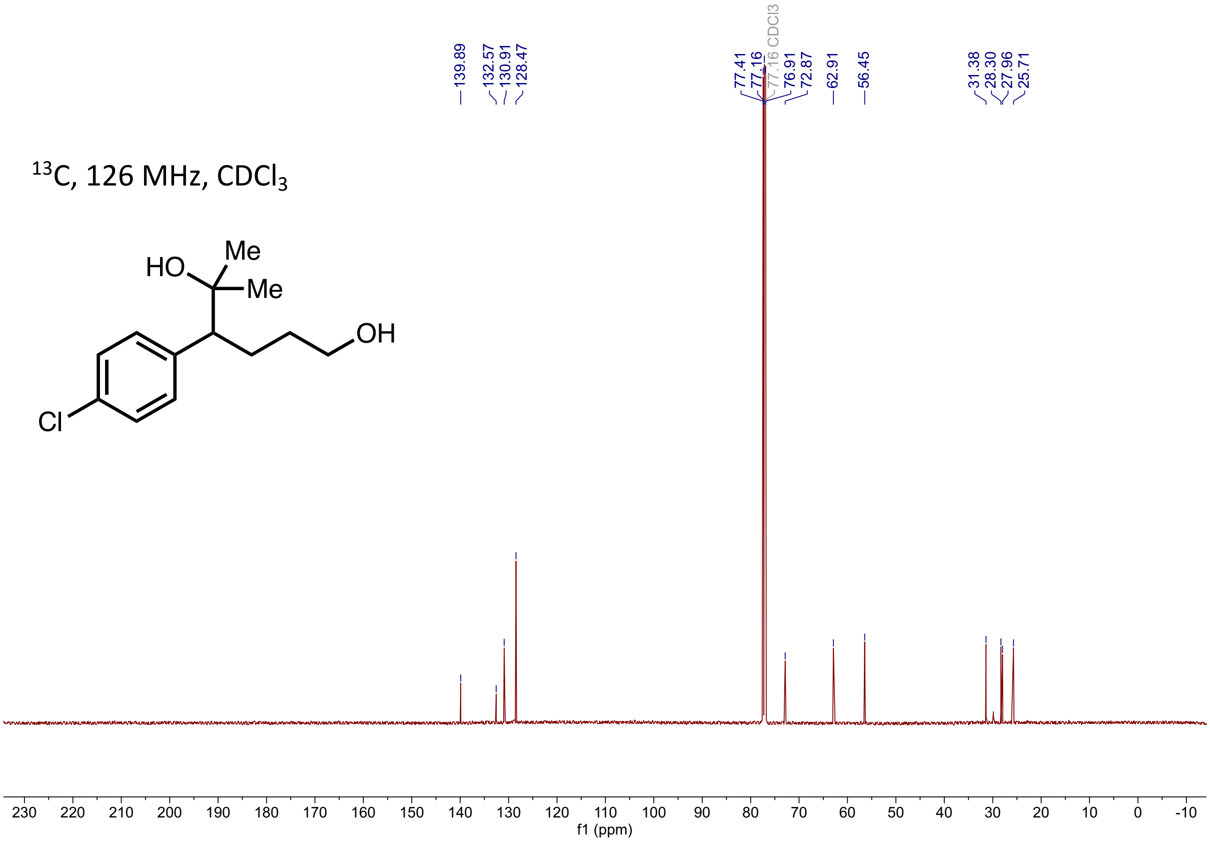
139.89
132.57 130.91 128.47
77.41 77.16 77.16 CDCl3 76.91 72.87
62.91
56.45
31.38 28.30 27.96 25.71
^{13}C, 126 MHz, CDCl$_3$
HO
Me
Me
OH
Cl
f1 (ppm)

(S6)

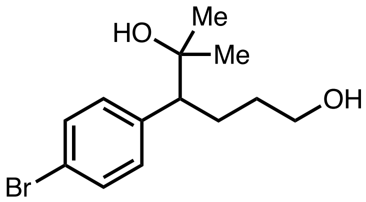

Prepared according to general procedure A, step 2 (i) using 3-(4-bromophenyl)tetrahydro-2*H*-pyran-2-one (3.5 g, 13.7 mmol), THF (28 mL) and methylmagnesium bromide solution (10 mL, 27.4 mmol). The crude residue was purified by flash column chromatography (eluent = 30 to 80% EtOAc in hexanes, silica gel) to afford product as a white solid (2.5 g, 64% yield).

Mp.: 85-90 °C; **R_f** = 0.46 (eluent = 80% EtOAc in hexanes); **ν_{max} / cm^{-1}** (thin film) 3304, 2986, 2920, 2864, 1371, 1152, 1063; **^{1}H NMR (500 MHz, $CDCl_3$)** δ 7.44 – 7.42 (m, 1H), 7.31 – 7.22 (m, 2H), 7.12 – 7.11 (m, 1H), 3.59 – 3.57 (t, *J* = 6.4 Hz, 2H), 2.62 – 2.54 (ddd, *J* = 20.2, 12.1, 3.2 Hz, 1H), 2.00 – 1.95 (m, 1H), 1.81 – 1.75 (m, 1H), 1.36 – 1.31 (m, 2H), 1.18 (s, 3H), 1.16 (s, 3H); **^{13}C NMR (126 MHz, $CDCl_3$)** δ 140.4, 131.4, 129.6, 128.3, 126.8, 120.6, 72.7, 62.9, 57.1, 56.5, 31.4, 28.0, 25.7; **HRMS (Cl^+)** ***m/z*** $[M - H_2O - H]^+$ Calcd for $C_{13}H_{16}BrO$ 267.0379; found 267.0380.

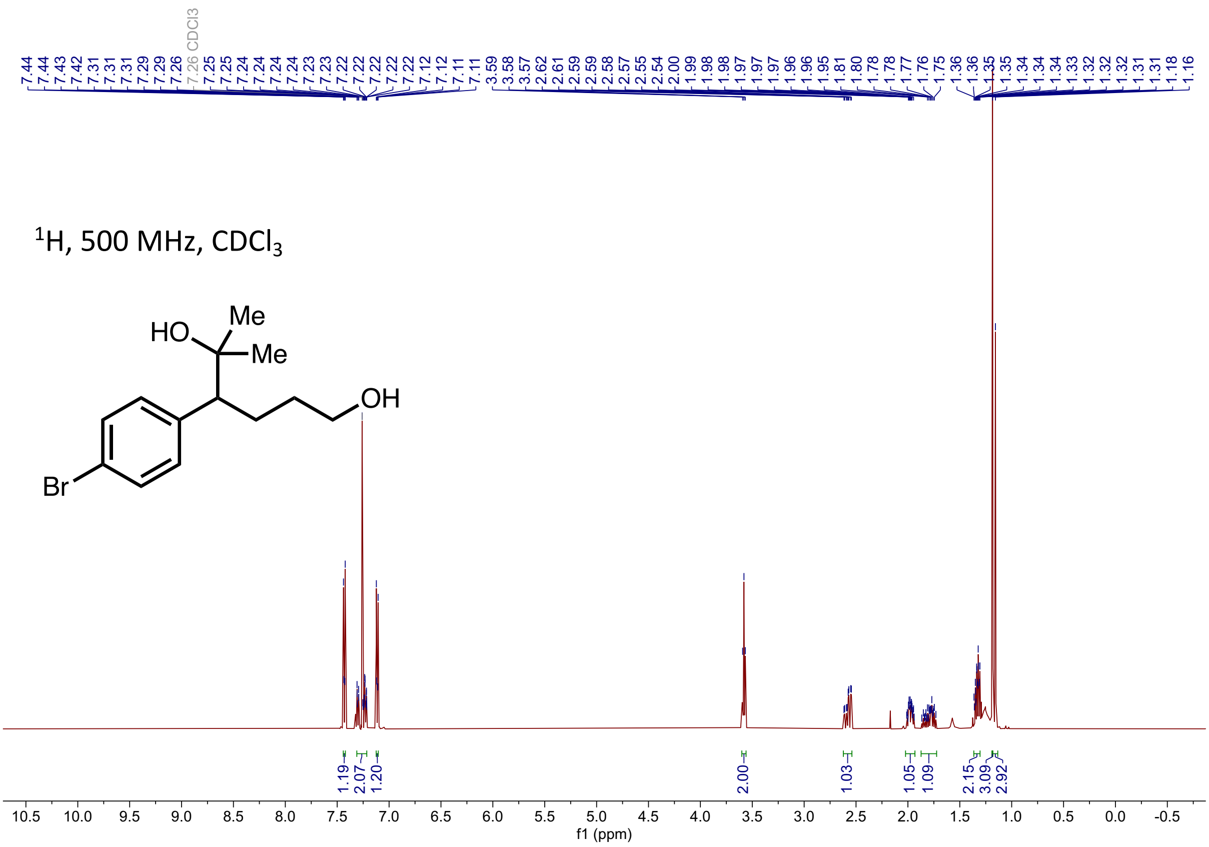
^{1}H, 500 MHz, CDCl$_3$
HO
Me
Me
OH
Br
f1 (ppm)

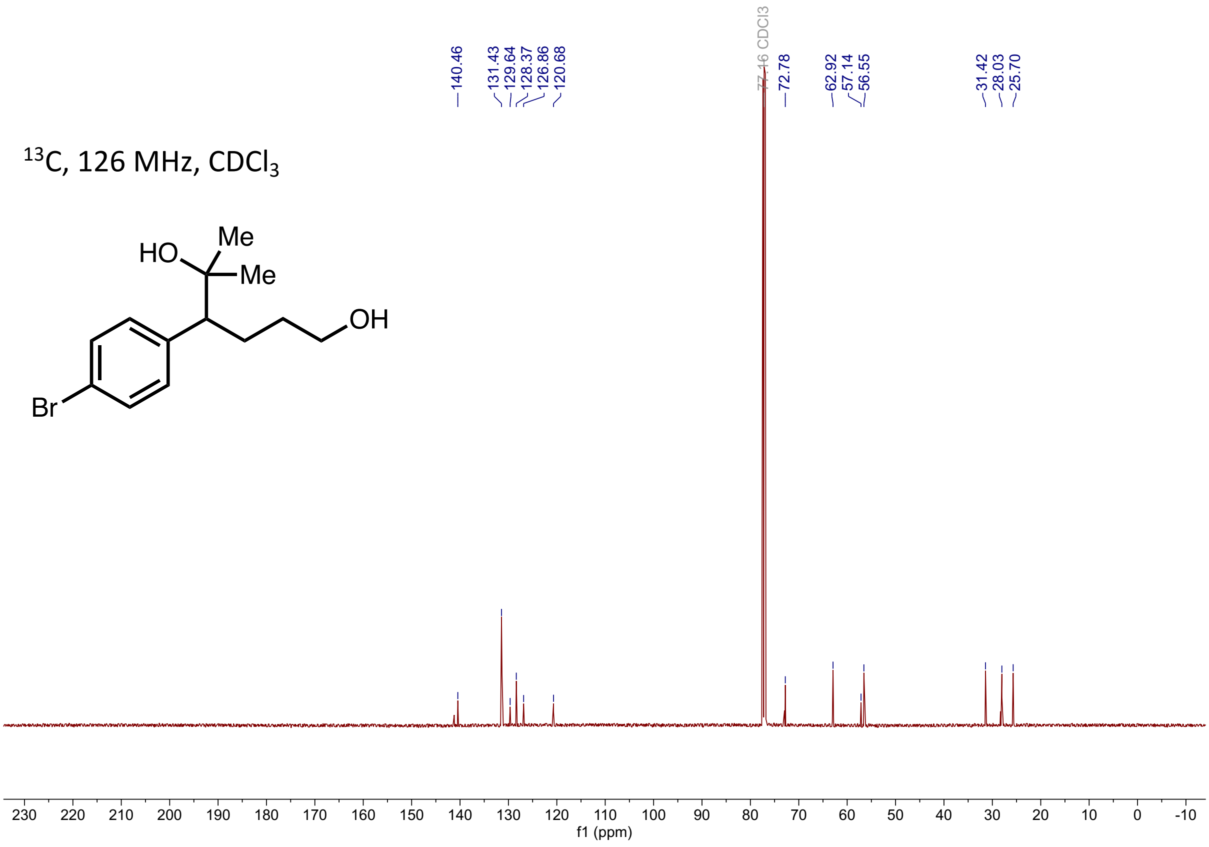
^{13}C, 126 MHz, CDCl$_3$
HO
Me
Me
OH
Br
140.46
131.43
129.64
128.37
126.86
120.68
77.16 CDCl3
72.78
62.92
57.14
56.55
31.42
28.03
25.70
f1 (ppm)

(S7)

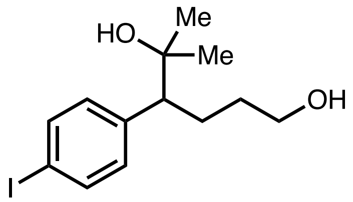

Prepared according to general procedure B, step 2 using 3-(4-iodophenyl)-2-methylhex-5-en-2-ol (3.44 g, 11.0 mmol), THF (22 mL) and borane dimethylsulfide solution (6.8 mL, 13.2 mmol). The crude residue was purified by flash column chromatography (eluent = 30 to 50% EtOAc in hexanes, silica gel) to afford product as a white solid (2.83 g, 77% yield).

Mp.: 98-103 °C; **R_f** = 0.15 (eluent = 50% EtOAc in hexanes); **ν_{max} / cm^{-1}** (thin film) 3260, 2970, 2931, 2866, 1364, 1140, 1065; **^{1}H NMR (500 MHz, $CDCl_3$)** δ 7.64 – 7.62 (m, 2H), 7.00 – 6.98 (m, 2H), 3.59 – 3.57 (t, *J* = 6.4 Hz, 2H), 2.56 – 2.53 (dd, *J* = 12.1, 3.2 Hz, 1H), 1.99 – 1.94 (m, 1H), 1.79 – 1.74 (m, 1H), 1.35 – 1.29 (m, 2H), 1.18 (s, 3H), 1.16 (s, 3H); **^{13}C NMR (126 MHz, $CDCl_3$)** δ 141.1, 137.4, 131.6, 92.1, 72.7, 62.9, 56.6, 31.4, 28.2, 28.0, 25.6; **HRMS (EI^+) *m/z*** [M - H_2O]$^+$ Calcd for $C_{13}H_{17}IO$ 316.0318; found 316.0314.

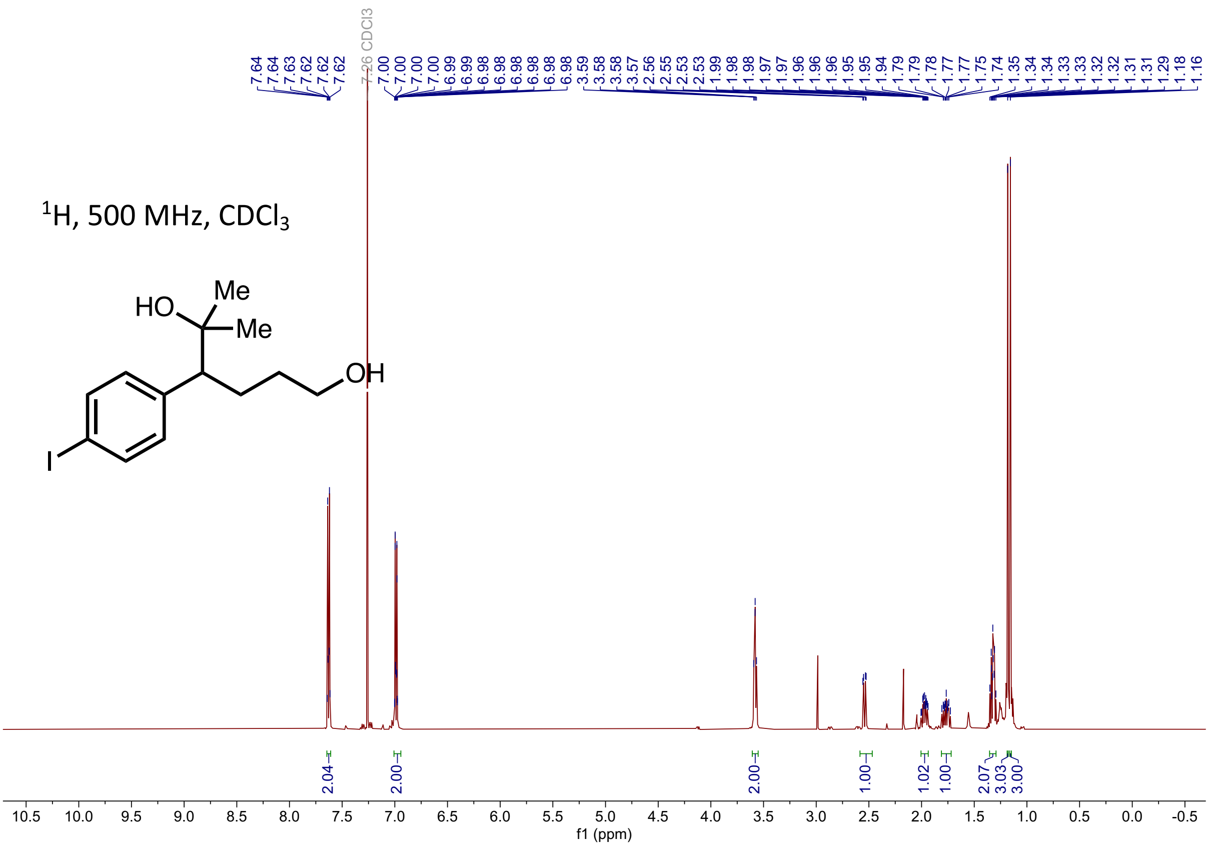

^{1}H, 500 MHz, $CDCl_3$
HO
Me
Me
OH
I
f1 (ppm)

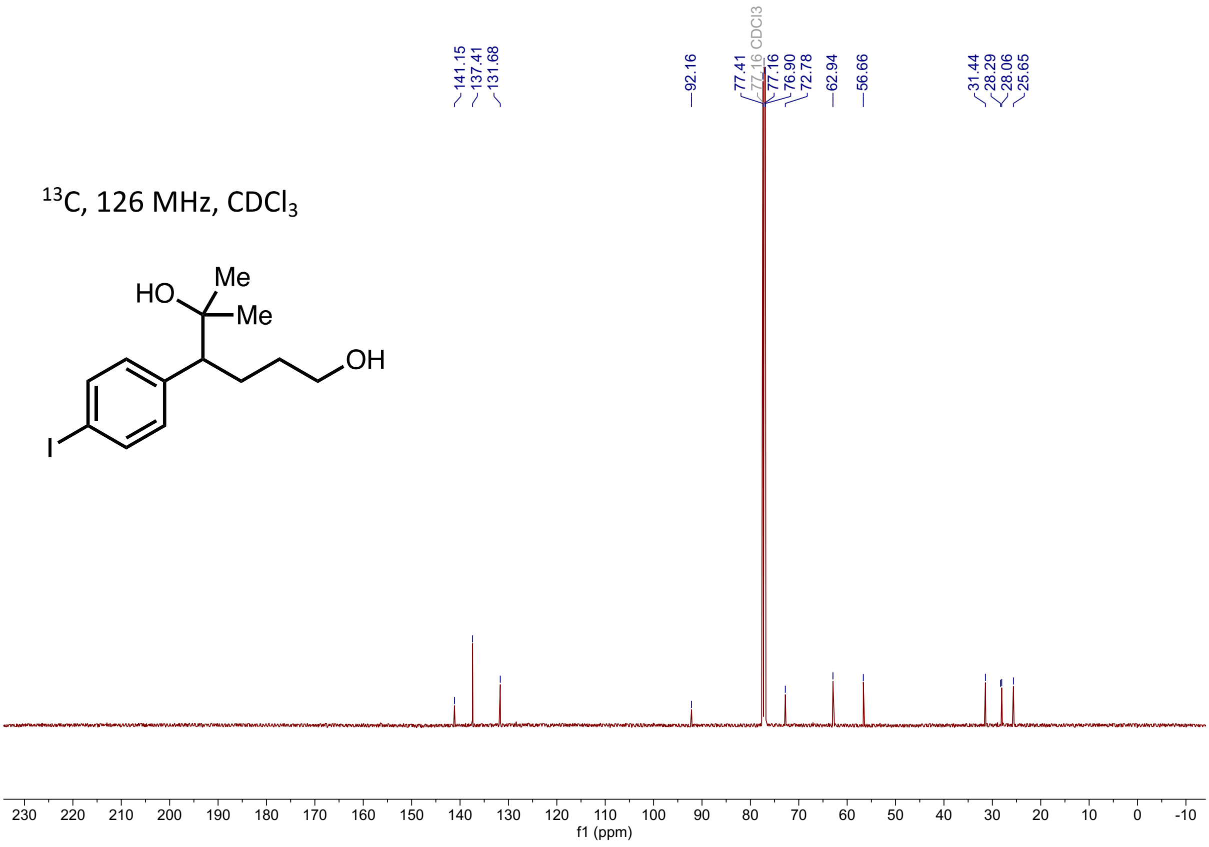

^{13}C, 126 MHz, $CDCl_3$
HO
Me
Me
OH
I
f1 (ppm)

(S8)

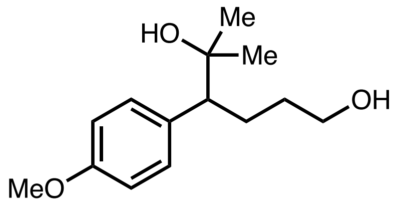

Prepared according to general procedure A, step 2 (i) using 3-(4-methoxyphenyl)tetrahydro-2*H*-pyran-2-one (1.03 g, 5 mmol), THF (10 mL) and methyllithium solution (7.8 mL, 12.5 mmol). The crude residue was purified by flash column chromatography (eluent = 30 to 50% EtOAc in hexanes, silica gel) to afford product as a white solid (1.06 g, 89% yield).

Mp.: 73-78 °C; **R_f** = 0.21 (eluent = 30% EtOAc in hexanes); **ν_{max} / cm^{-1}** (thin film) 3257 (br), 2981, 2887, 2360, 1379**; ^{1}H NMR (500 MHz, $CDCl_3$)** δ 7.15 – 7.12 (m, 2H), 6.87 – 6.84 (m, 2H), 3.80 (s, 3H), 3.59 – 3.56 (t, *J* = 6.5 Hz, 2H), 2.57 – 2.53 (dd, *J* = 12.1, 3.2 Hz, 1H), 1.98 – 1.91 (m, 1H), 1.80 – 1.72 (m, 1H), 1.38 – 1.30 (m, 2H), 1.18 – 1.16 (d, *J* = 9.5 Hz, 6H); **^{13}C NMR (126 MHz, $CDCl_3$)** δ 158.5, 132.9, 130.5, 113.7, 73.0, 63.0, 56.2, 55.3, 31.5, 27.9, 25.7; **HRMS (CI^+) *m/z*** [M - OH]$^+$ Calcd for $C_{14}H_{21}O_2$ 221.1536; found 221.1535.

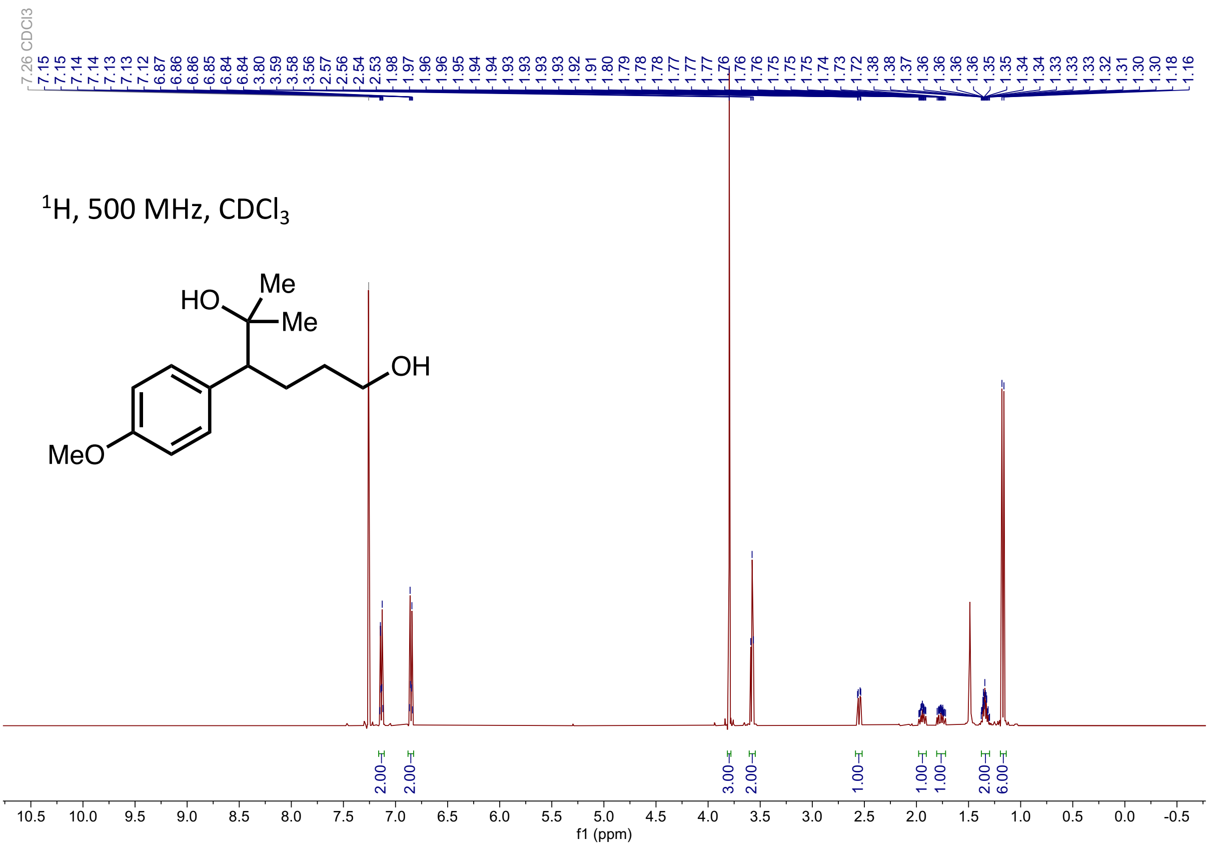
7.26 CDCl3
7.15 7.15 7.14 7.14 7.13 7.13 7.12 6.87 6.86 6.86 6.85 6.84 6.84 3.80 3.59 3.58 3.56 2.57 2.56 2.54 2.53 1.98 1.97 1.96 1.96 1.95 1.94 1.93 1.93 1.93 1.93 1.92 1.91 1.80 1.79 1.78 1.78 1.77 1.77 1.77 1.76 1.76 1.76 1.75 1.75 1.74 1.73 1.72 1.38 1.38 1.37 1.36 1.36 1.36 1.36 1.35 1.35 1.34 1.34 1.33 1.33 1.33 1.32 1.31 1.30 1.30 1.18 1.16
1H, 500 MHz, CDCl3
HO
Me
Me
OH
MeO
2.00 2.00 3.00 2.00 1.00 1.00 1.00 2.00 6.00
10.5 10.0 9.5 9.0 8.5 8.0 7.5 7.0 6.5 6.0 5.5 5.0 4.5 4.0 3.5 3.0 2.5 2.0 1.5 1.0 0.5 0.0 -0.5
f1 (ppm)

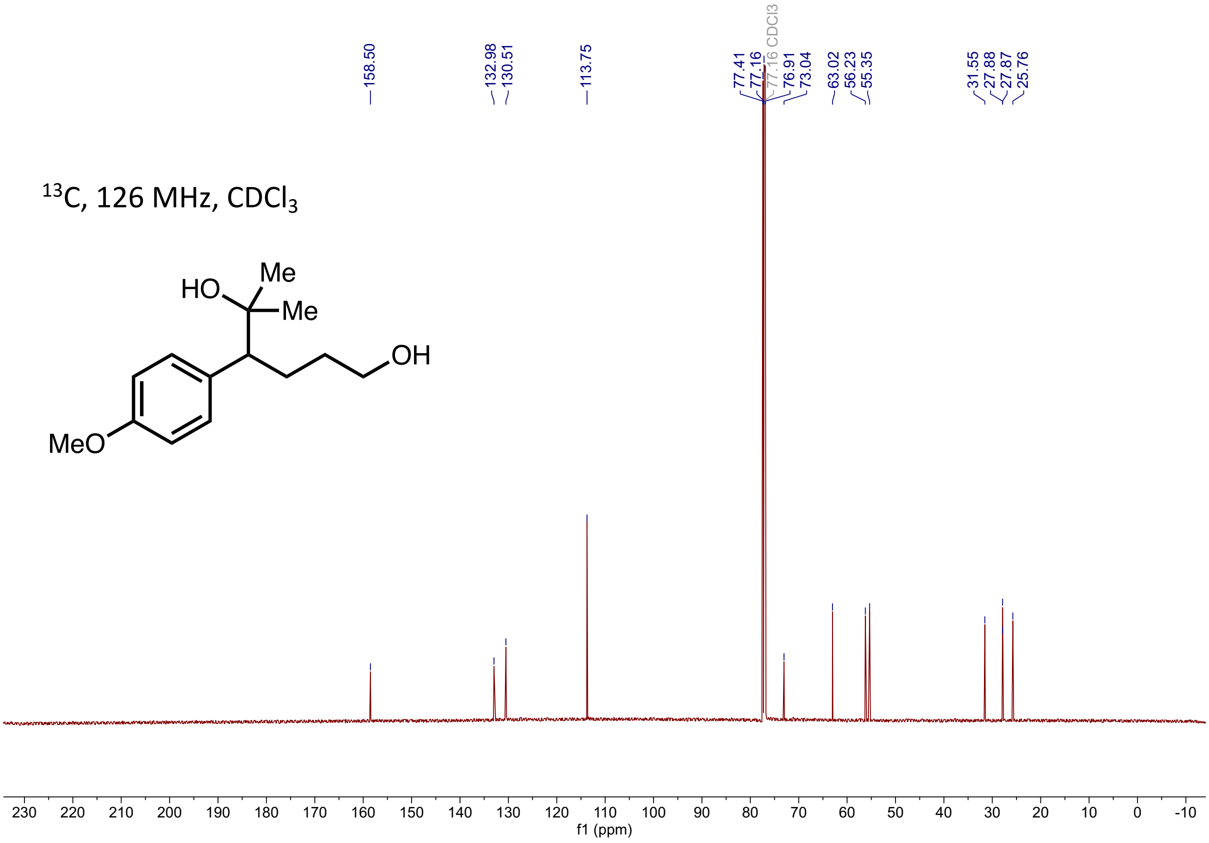
158.50
132.98 130.51
113.75
77.41 77.16 77.16 CDCl3 76.91 73.04
63.02 56.23 55.35
31.55 27.88 27.87 25.76
13C, 126 MHz, CDCl3
HO
Me
Me
OH
MeO
230 220 210 200 190 180 170 160 150 140 130 120 110 100 90 80 70 60 50 40 30 20 10 0 -10
f1 (ppm)

(S9)

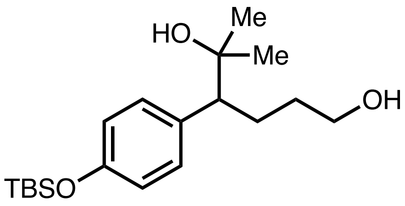

Prepared according to general procedure B, step 2 using 3-(4-((*tert*-butyldimethylsilyl)oxy)phenyl)-2-methylhex-5-en-2-ol (3.4 g, 10.5 mmol), THF (21 mL) and borane dimethylsulfide solution (6.3 mL, 12.6 mmol). The crude residue was purified by flash column chromatography (eluent = 30 to 50% EtOAc in hexanes, silica gel) to afford product as a white solid (2.41 g, 67% yield).

Mp.: 75-80 °C; **R_f** = 0.16 (eluent = 50% EtOAc in hexanes); **ν_{max} / cm^{-1}** (thin film) 3273, 2953, 2928, 2859, 1510, 1255, 1172, 1065, 923,777; **^{1}H NMR (500 MHz, $CDCl_3$)** δ 7.08 – 7.05 (m, 2H), 6.79 – 6.77 (m, 2H), 3.59 – 3.57 (t, *J* = 6.5 Hz, 2H), 2.55 – 2.52 (dd, *J* = 12.1, 3.2 Hz, 1H), 1.95 – 1.90 (m, 1H), 1.79 – 1.71 (m, 1H), 1.38 – 1.32 (m, 2H), 1.17 (s, 3H), 1.16 (s, 3H), 0.98 (s, 9H), 0.20 (s, 6H); **^{13}C NMR (126 MHz, $CDCl_3$)** δ 154.5, 133.5, 130.4, 119.8, 72.9, 63.1, 56.3, 42.8, 31.6, 27.9, 27.8, 25.8, 18.3, -4.2; **HRMS (ESP^{+})** ***m/z*** [M - OH]$^{+}$ Calcd for $C_{19}H_{33}O_2Si$ 321.2250; found 321.2250.

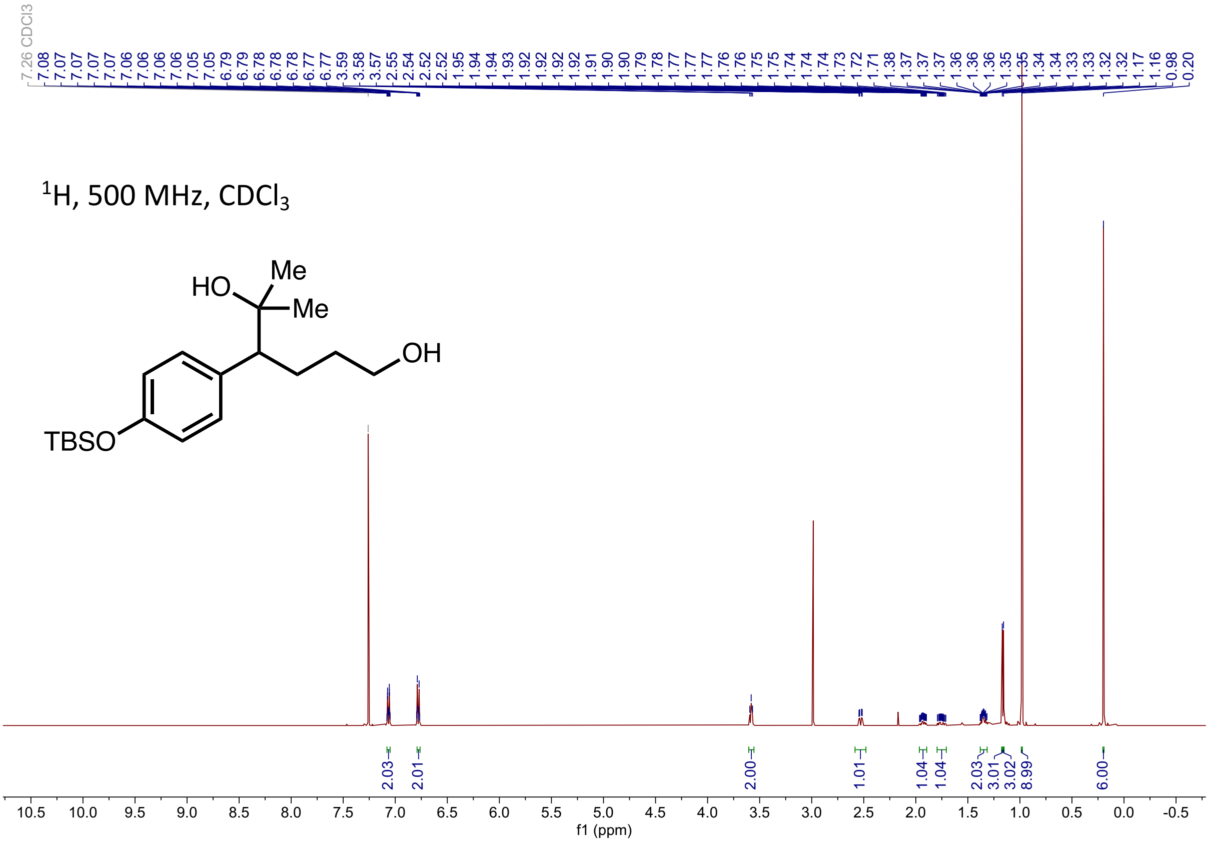

7.26 CDCl3
7.08 7.07 7.07 7.07 7.06 7.06 7.06 7.05 7.05 6.79 6.79 6.78 6.78 6.78 6.77 6.77 3.59 3.58 3.57 2.55 2.54 2.52 2.52 1.95 1.94 1.94 1.93 1.92 1.92 1.92 1.92 1.91 1.90 1.90 1.79 1.78 1.77 1.77 1.77 1.76 1.76 1.75 1.75 1.74 1.74 1.74 1.73 1.72 1.71 1.38 1.37 1.37 1.37 1.36 1.36 1.36 1.35 1.35 1.34 1.34 1.33 1.33 1.32 1.32 1.17 1.16 0.98 0.20
1H, 500 MHz, CDCl3
HO
Me
Me
OH
TBSO
2.03 2.01 2.00 1.01 1.04 1.04 2.03 3.01 3.02 8.99 6.00
10.5 10.0 9.5 9.0 8.5 8.0 7.5 7.0 6.5 6.0 5.5 5.0 4.5 4.0 3.5 3.0 2.5 2.0 1.5 1.0 0.5 0.0 -0.5
f1 (ppm)

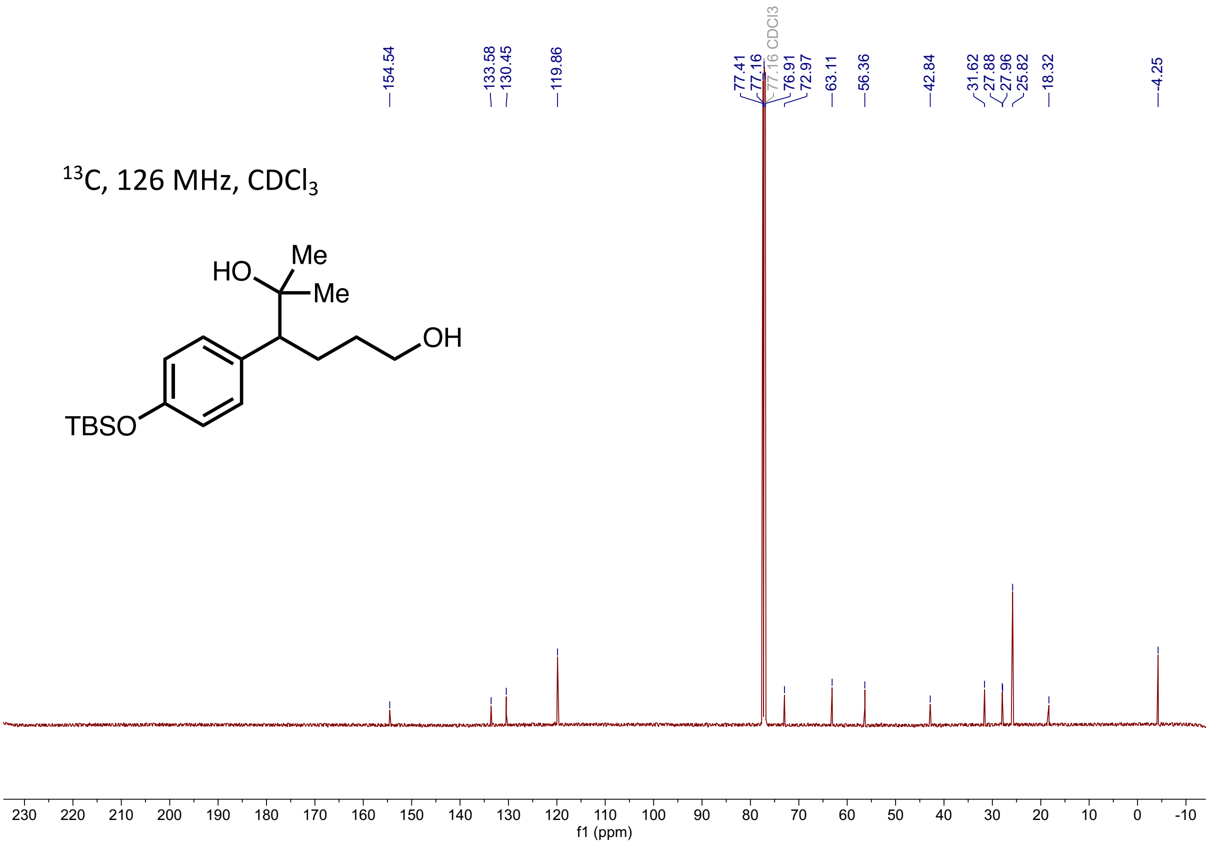

154.54
133.58 130.45
119.86
77.41 77.16 77.16 CDCl3 76.91 72.97
63.11
56.36
42.84
31.62 27.88 27.96 25.82
18.32
-4.25
13C, 126 MHz, CDCl3
HO
Me
Me
OH
TBSO
230 220 210 200 190 180 170 160 150 140 130 120 110 100 90 80 70 60 50 40 30 20 10 0 -10
f1 (ppm)

(S10)

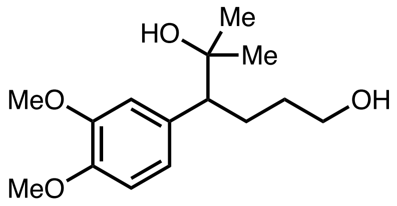

Prepared according to general procedure A, step 2 (ii) using 3-(3,4-dimethoxyphenyl) tetrahydro-2*H*-pyran-2-one (0.76 g, 3.25 mmol), THF (6.5 mL) and methylmagnesium bromide (2.38 mL, 7.16 mmol). The crude residue was purified by flash column chromatography (eluent = 30 to 50% EtOAc in hexanes, silica gel) to afford product as a white solid (0.506 g, 66% yield).

Mp.: 70-74 °C; $\mathbf{R_f}$ = 0.20 (eluent = 50% EtOAc in petroleum ether); $\mathbf{\nu_{max}}$ **/ cm^{-1}** (thin film) 3298(br), 2931, 1377; **^{1}H NMR (500 MHz, $CDCl_3$)** δ 6.82 – 6.81 (m, 1H), 6.77 – 6.75 (m, 2H), 3.87 (d, *J* = 1.1 Hz, 6H), 3.58 (t, *J* = 6.5 Hz, 2H), 2.54 (dd, *J* = 12.1, 3.2 Hz, 1H), 1.97 – 1.91 (m, 1H), 1.80 – 1.72(m, 1H), 1.38 – 1.35 (m, 2H), 1.18 (d, *J* = 6.0 Hz, 6H); **^{13}C NMR (126 MHz, $CDCl_3$)** δ 148.5, 147.7, 133.3, 121.3, 112.6, 110.8, 72.7, 62.7, 56.4, 55.8, 55.7, 31.3, 27.9, 27.5, 25.6; **HRMS (CI$^+$) *m/z*** $[M - H_2O]^+$ Calcd for $C_{15}H_{22}O_3$ 250.1563; found 250.1560.

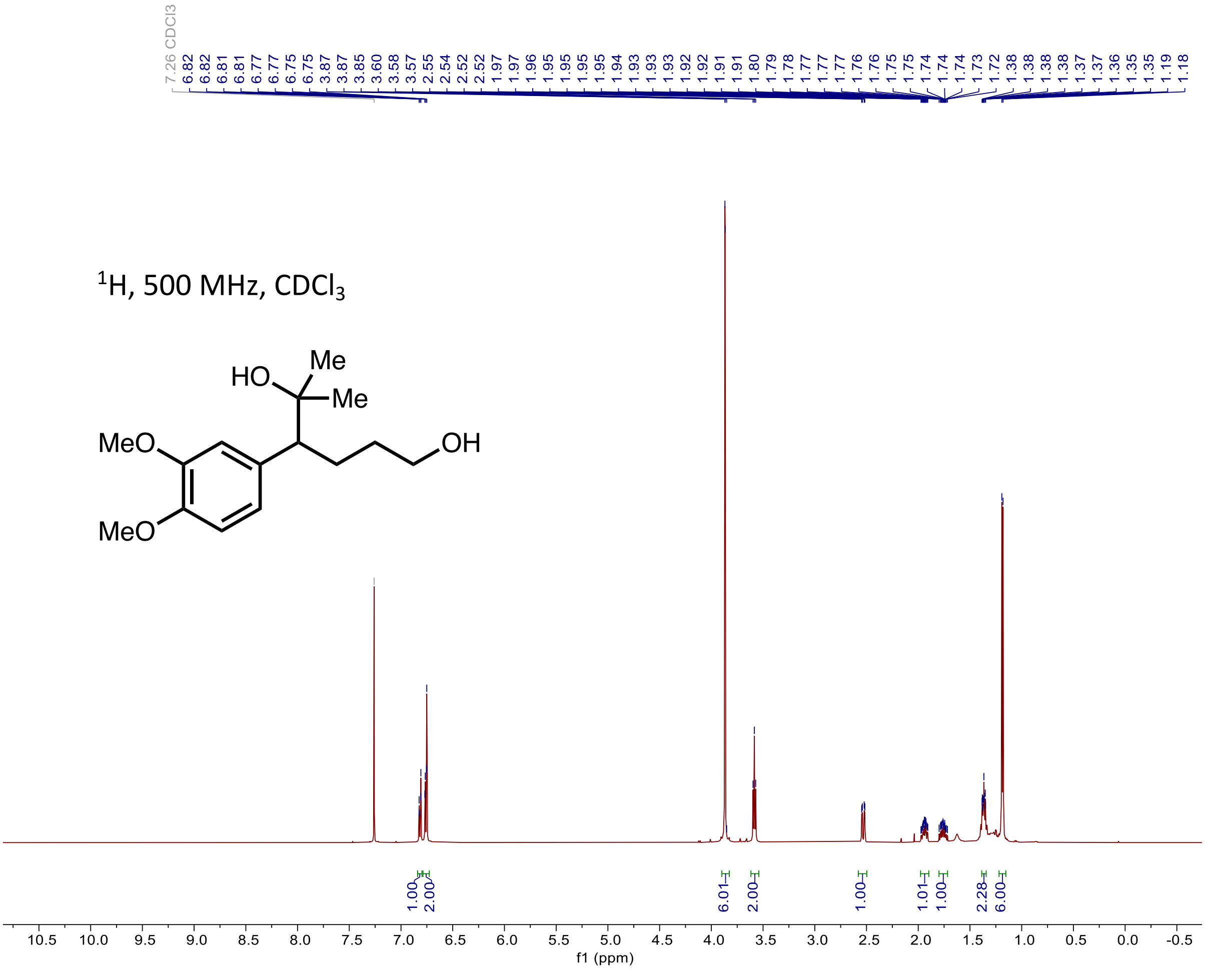
7.26 CDCl3
6.82
6.82
6.81
6.81
6.77
6.77
6.75
6.75
3.87
3.87
3.85
3.60
3.58
3.57
2.55
2.54
2.52
2.52
1.97
1.97
1.96
1.95
1.95
1.95
1.95
1.94
1.93
1.93
1.93
1.92
1.92
1.91
1.91
1.80
1.79
1.78
1.77
1.77
1.77
1.76
1.76
1.75
1.75
1.74
1.74
1.74
1.73
1.72
1.38
1.38
1.38
1.38
1.37
1.37
1.36
1.35
1.35
1.19
1.18
1H, 500 MHz, CDCl3
HO
Me
Me
MeO
OH
MeO
1.00
2.00
6.01
2.00
1.00
1.01
1.00
2.28
6.00
10.5
10.0
9.5
9.0
8.5
8.0
7.5
7.0
6.5
6.0
5.5
5.0
4.5
4.0
3.5
3.0
2.5
2.0
1.5
1.0
0.5
0.0
-0.5
f1 (ppm)

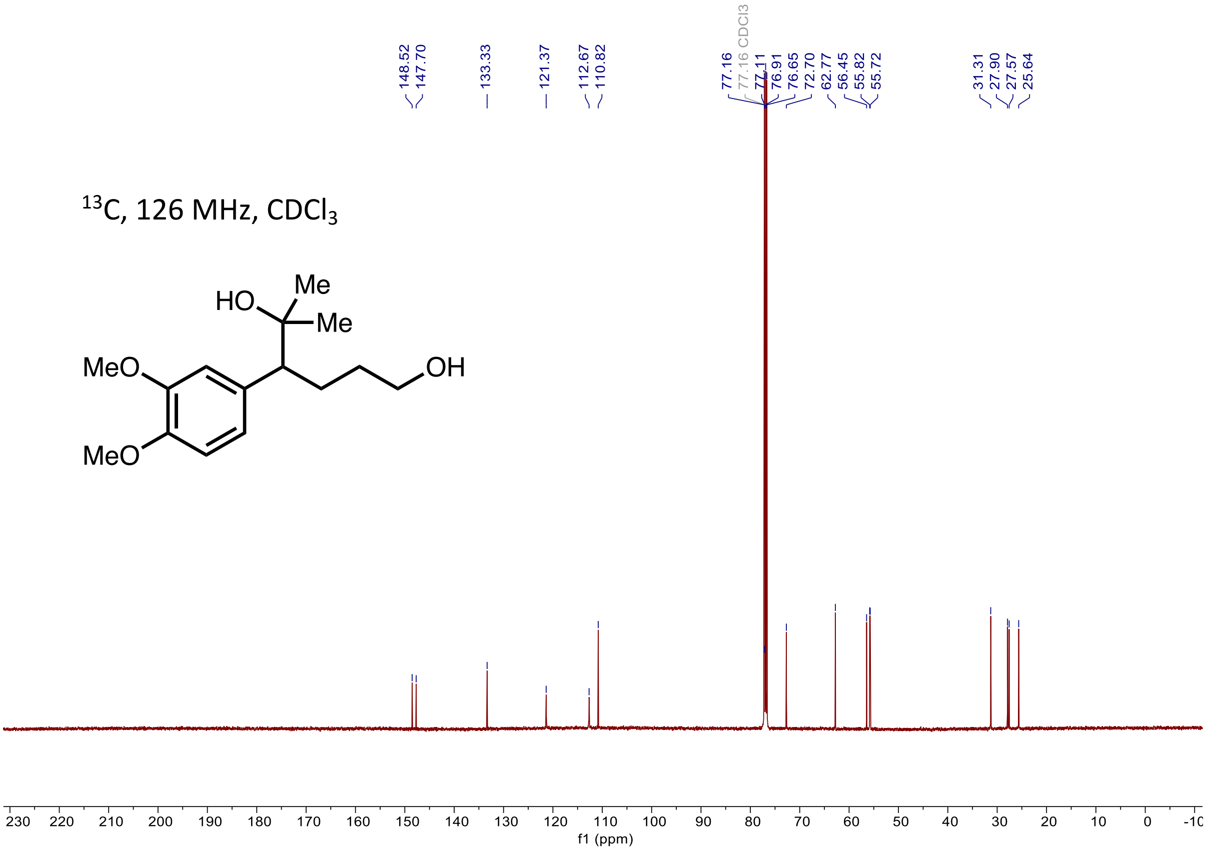
148.52
147.70
133.33
121.37
112.67
110.82
77.16
77.16 CDCl3
77.11
76.91
76.65
72.70
62.77
56.45
55.82
55.72
31.31
27.90
27.57
25.64
13C, 126 MHz, CDCl3
HO
Me
Me
MeO
OH
MeO
230
220
210
200
190
180
170
160
150
140
130
120
110
100
90
80
70
60
50
40
30
20
10
0
-10
f1 (ppm)

(S11)

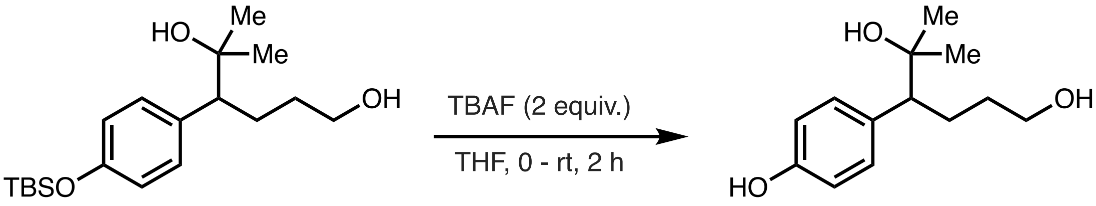

To a flame-dried flask was added **S9** (1.5 g, 4.43 mmol) in dry THF (15 mL) and the solution was cooled to 0 °C. Tetra-*n*-butylammonium fluoride (TBAF) (9 mL, 1M solution in THF, 9 mmol) was added and the resulting mixture was warm up to room temperature and left stirring overnight. The resulting mixture was diluted with DCM (20 mL) and quenched with water (5 mL). The organic layer was washed with brine, collected, dried over $MgSO_4$, filtered and concentrated *in vacuo* yielding crude residue. The crude residue was purified by flash column chromatography (eluent = 20 to 30% EtOAc in hexanes, silica gel) to afford product as a white solid (318 mg, 32% yield).

Mp.: 120-124 °C; **R_f** = 0.20 (eluent = 80% EtOAc in hexanes); **ν_{max} / cm^{-1}** (thin film) 3349, 3321, 3140, 2995, 2937, 1597, 1516,1456, 1244, 1155, 1017; **^{1}H NMR (500 MHz, CD_3OD)** δ 7.03 – 6.96 (m, 2H), 6.68 – 6.63 (m, 2H), 3.45 – 3.41 (t, *J* = 6.7 Hz, 2H), 2.45 – 2.40 (dd, *J* = 12.2, 3.0 Hz, 1H), 1.99 – 1.88 (m, 1H), 1.73 – 1.60 (m, 1H), 1.29 – 1.17 (m, 2H), 1.06 (s, 3H), 1.04 (s, 3H); **^{13}C NMR (126 MHz, $CDCl_3$)** δ 156.9, 133.8, 131.4, 115.7, 115.6, 73.8, 63.1, 57.5, 32.4, 29.3, 26.5, 26.3; **HRMS (ESN$^-$)** ***m/z*** [M - H]$^+$ Calcd for $C_{13}H_{19}O_3$ 223.1334; found 223.1325.

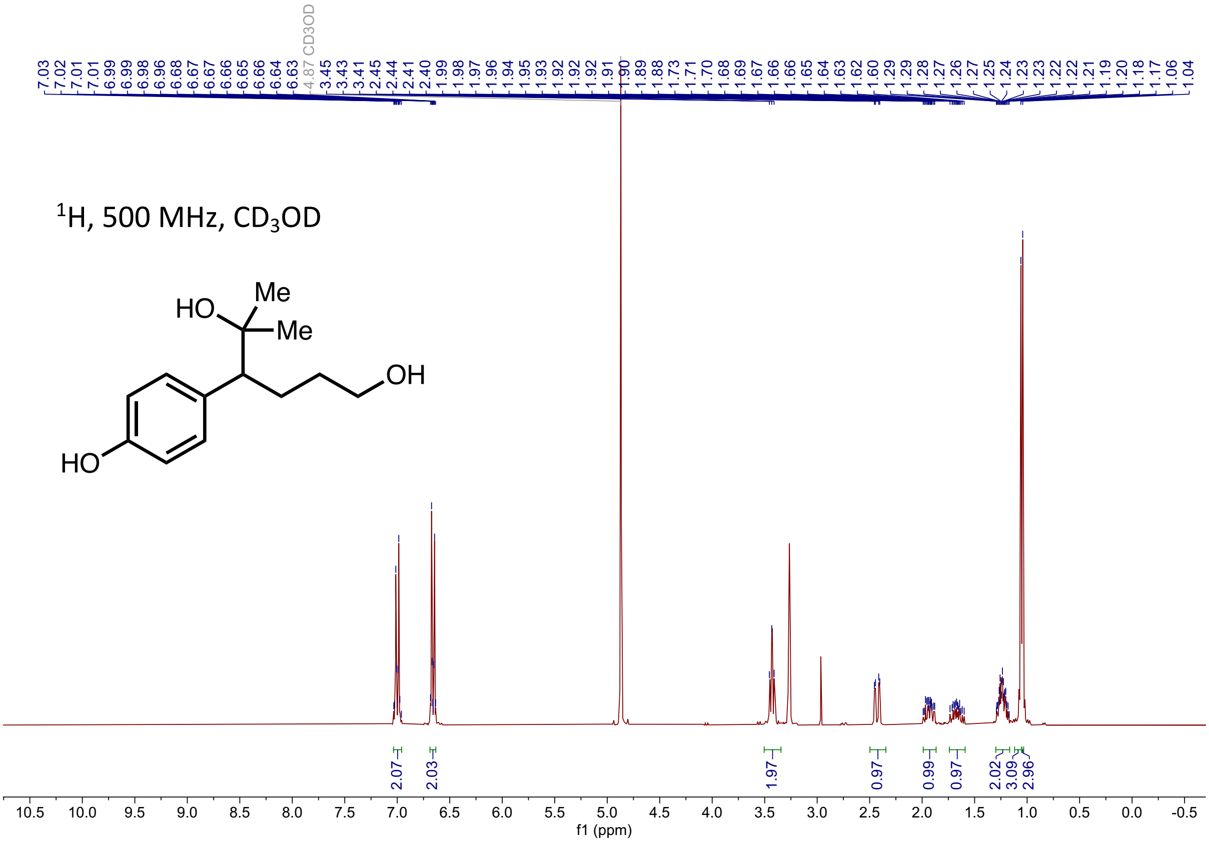
7.03 7.02 7.01 7.01 6.99 6.99 6.98 6.96 6.68 6.67 6.67 6.66 6.65 6.66 6.64 6.63 4.87 CD3OD 3.45 3.43 3.41 2.45 2.44 2.41 2.40 1.99 1.98 1.97 1.96 1.94 1.95 1.93 1.92 1.92 1.92 1.91 1.90 1.89 1.88 1.73 1.71 1.70 1.68 1.69 1.67 1.66 1.66 1.65 1.64 1.63 1.62 1.60 1.29 1.29 1.28 1.27 1.26 1.27 1.25 1.24 1.23 1.23 1.22 1.22 1.21 1.19 1.20 1.18 1.17 1.06 1.04
^{1}H, 500 MHz, CD$_3$OD
HO
Me
Me
OH
HO
2.07
2.03
1.97
0.97
0.99
0.97
2.02
3.09
2.96
10.5 10.0 9.5 9.0 8.5 8.0 7.5 7.0 6.5 6.0 5.5 5.0 4.5 4.0 3.5 3.0 2.5 2.0 1.5 1.0 0.5 0.0 -0.5
f1 (ppm)

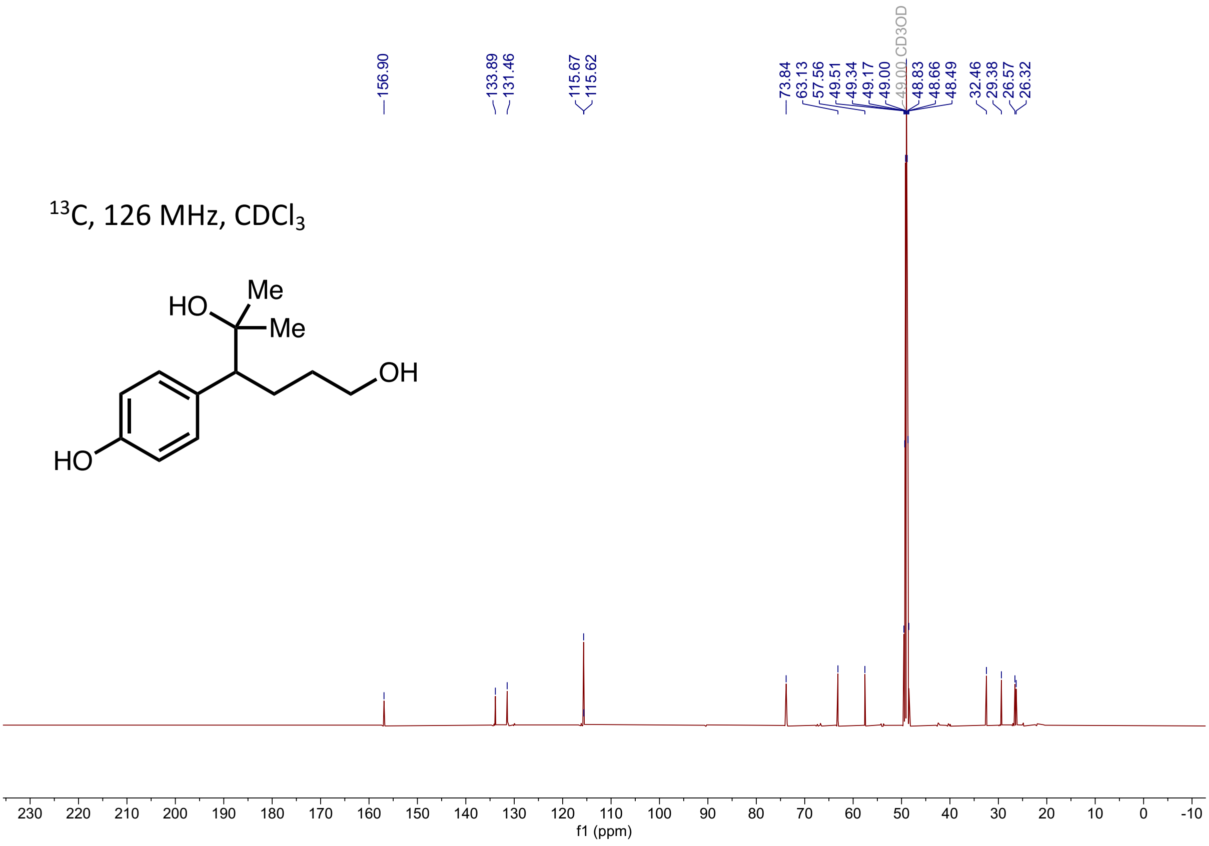
156.90
133.89 131.46
115.67 115.62
73.84 63.13 57.56 49.51 49.34 49.17 49.00 49.00 CD3OD 48.83 48.66 48.49 32.46 29.38 26.57 26.32
^{13}C, 126 MHz, CDCl$_3$
HO
Me
Me
OH
HO
230 220 210 200 190 180 170 160 150 140 130 120 110 100 90 80 70 60 50 40 30 20 10 0 -10
f1 (ppm)

(S12)

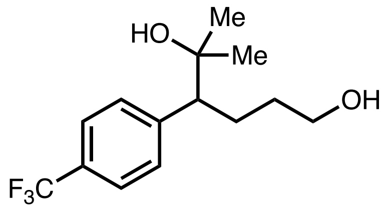

Prepared according to general procedure B, step 2 using 2-methyl-3-(4-(trifluoromethyl)phenyl)hex-5-en-2-ol (775 mg, 3 mmol) in THF (6 mL) and BH_3•Me_2S solution (1.8 mL, 3.6 mmol, 2.0 M in hexanes). The crude residue was purified by flash column chromatography (eluent = 30 to 50% EtOAc in hexanes, silica gel) to afford product as a white solid (572 mg, 69% yield).

Mp.: 103-111 °C; $\mathbf{R_f}$ = 0.23 (eluent = 20% EtOAc in hexanes); $\mathbf{\nu_{max}}$ **/ cm^{-1}** (thin film) 3261 (br), 2981, 2360, 1379; **^{1}H NMR (500 MHz, $CDCl_3$)** δ 7.57 – 7.55 (m, 2H), 7.37 – 7.35 (m, 2H), 3.60 – 3.58 (t, *J* = 6.4 Hz, 2H), 2.68 – 2.65 (dd, *J* = 12.1, 3.2 Hz, 1H), 2.06 – 1.99 (m, 1H), 1.88 – 1.80 (m, 1H), 1.33 – 1.29 (m, 2H), 1.21 (s, 3H), 1.16 (s, 3H); **^{13}C NMR (126 MHz, $CDCl_3$)** δ 145.8, 129.9, 125.1 (q, *J* = 3.8 Hz), 72.8, 62.8, 56.9, 42.8, 31.3, 28.5, 28.0, 25.7; **^{19}F NMR (471 MHz, $CDCl_3$)** δ -62.37; **HRMS (CI^+)** ***m/z*** $[M - OH]^+$ Calcd for $C_{14}H_{18}F_3O$ 259.1304; found 259.1301.

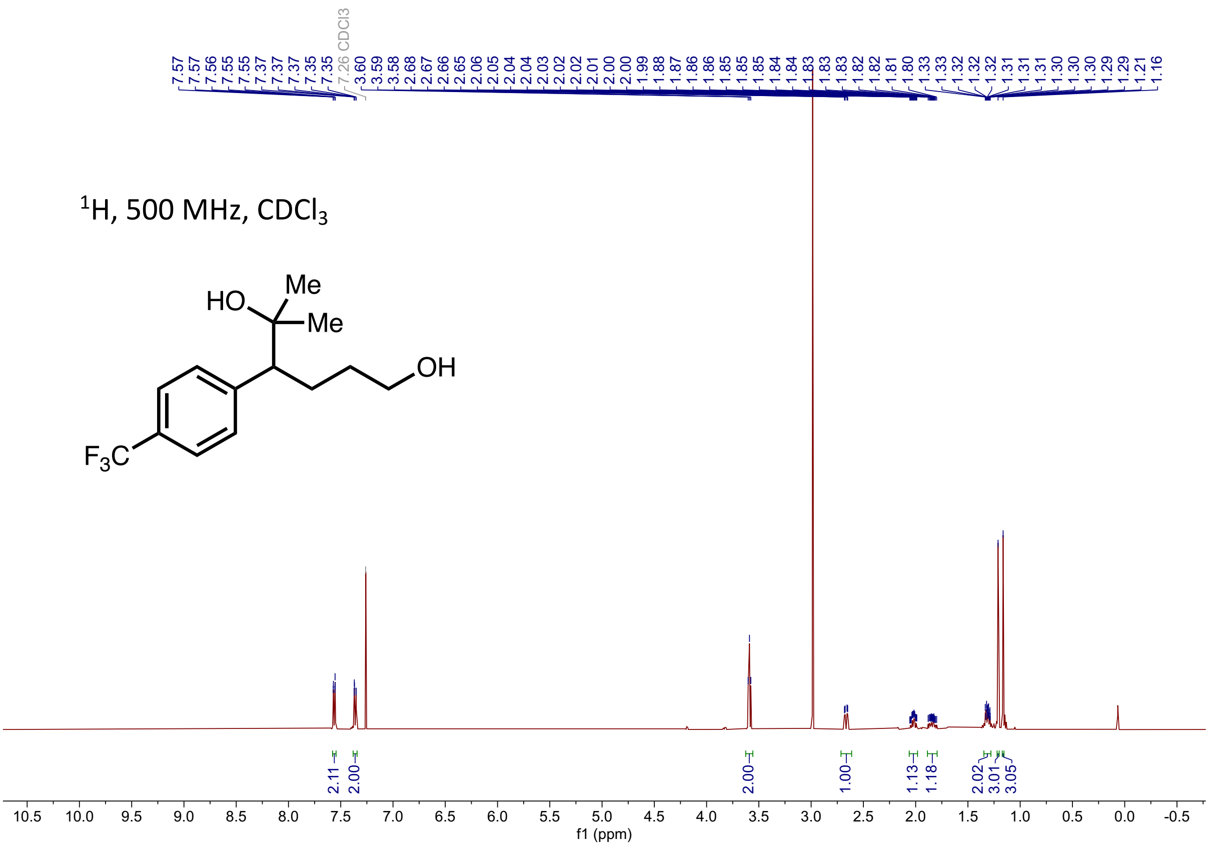
^{1}H, 500 MHz, $CDCl_3$
HO
Me
Me
OH
F_3C
7.57 7.57 7.56 7.55 7.55 7.37 7.37 7.37 7.35 7.35 7.26 CDCl3 3.60 3.59 3.58 2.68 2.67 2.66 2.65 2.06 2.05 2.04 2.04 2.03 2.02 2.02 2.01 2.00 2.00 1.99 1.88 1.87 1.86 1.86 1.85 1.85 1.84 1.84 1.83 1.83 1.83 1.82 1.81 1.80 1.33 1.33 1.32 1.32 1.32 1.31 1.31 1.30 1.30 1.29 1.21 1.16
2.11 2.00 2.00 1.00 1.13 1.18 2.02 3.01 3.05
f1 (ppm)

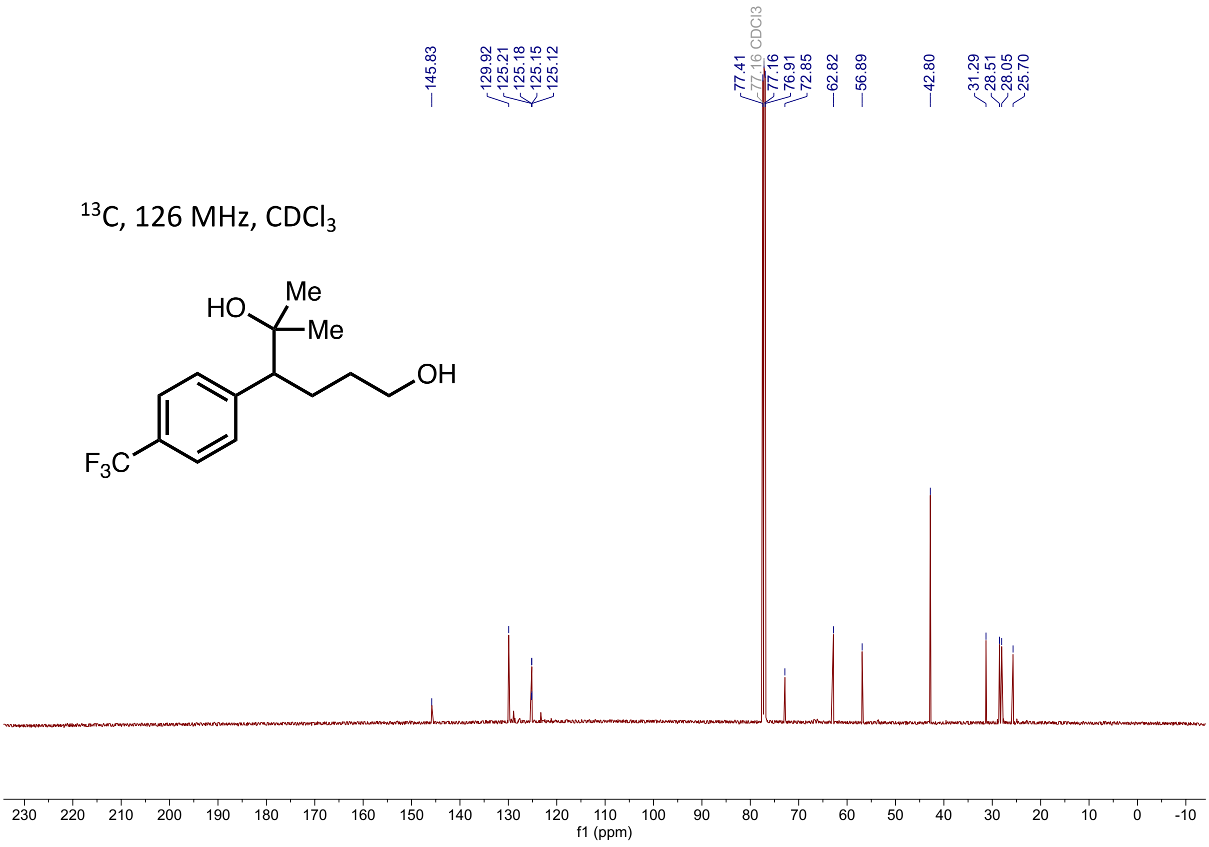
^{13}C, 126 MHz, $CDCl_3$
HO
Me
Me
OH
F_3C
145.83 129.92 125.21 125.18 125.15 125.12 77.41 77.16 CDCl3 77.16 76.91 72.85 62.82 56.89 42.80 31.29 28.51 28.05 25.70
f1 (ppm)

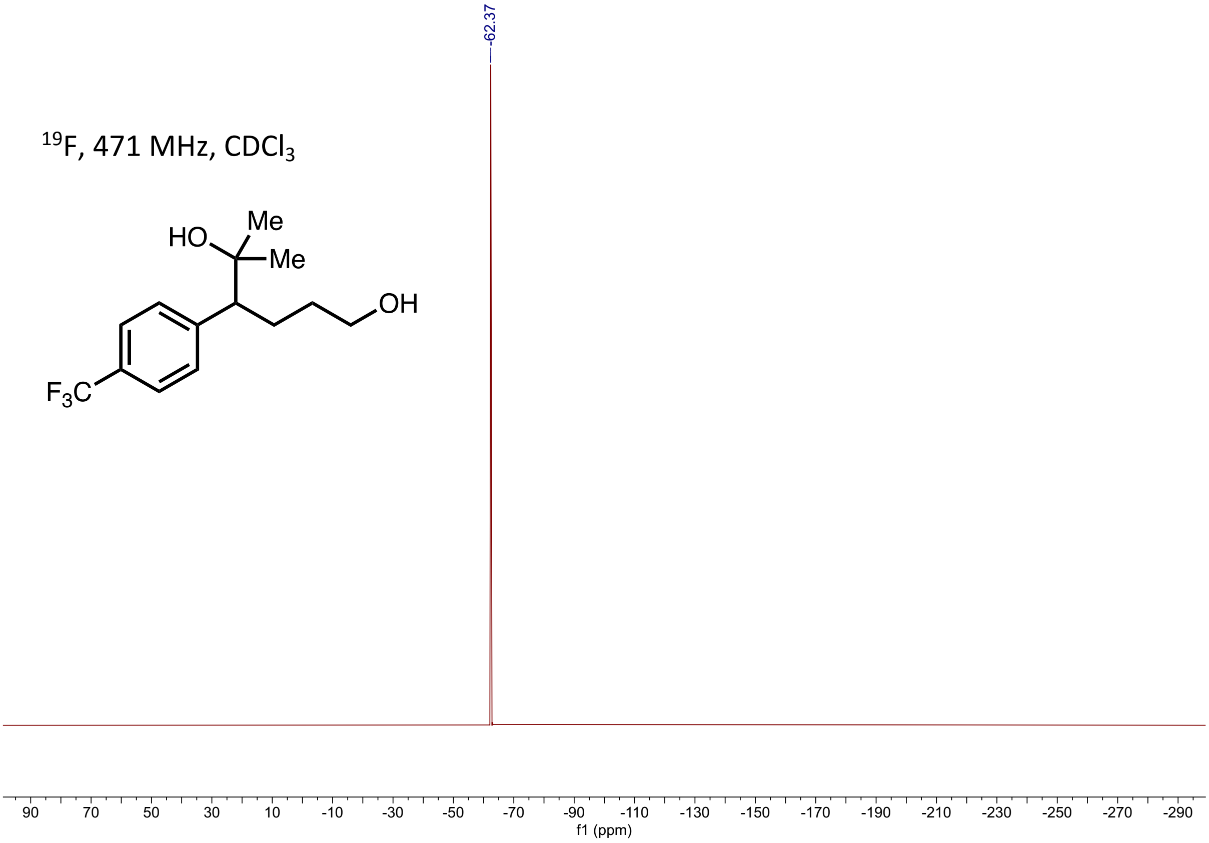
-62.37
^{19}F, 471 MHz, $CDCl_3$
HO
Me
Me
OH
F_3C
90 70 50 30 10 -10 -30 -50 -70 -90 -110 -130 -150 -170 -190 -210 -230 -250 -270 -290
f1 (ppm)

(S13)

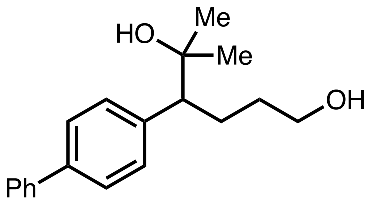

Prepared according to general procedure A, step 2 (ii) using 3-([1,1'-biphenyl]-4-yl)tetrahydro-2*H*-pyran-2-one (1.31 g, 5.2 mmol), THF (10.4 mL) and methylmagnesium bromide solution (3.5 mL, 10.4 mmol). The crude residue was purified by flash column chromatography (eluent = 30 to 50% EtOAc in hexanes, silica gel) to afford product as a white solid (0.89 g, 59% yield).

Mp.: 110 - 117 °C; **R_f** = 0.21 (eluent = 50% EtOAc in hexanes); **ν_{max} / cm^{-1}** (thin film) 3285, 2980,2926, 2860, 1485, 1371, 1153, 1060; **^{1}H NMR (500 MHz, $CDCl_3$)** δ 7.61 – 7.58 (m, 2H), 7.56 – 7.53 (m, 2H), 7.45 – 7.42 (m, 2H), 7.35 – 7.29 (m, 3H), 3.63 – 3.60 (t, *J* = 6.5 Hz, 2H), 2.67 – 2.64 (dd, *J* = 12.1, 3.2 Hz, 1H), 2.02 – 1.98 (m, 1H), 1.90 – 1.82 (m,1H), 1.42 – 1.38 (m, 2H), 1.23 (s, 6H); **^{13}C NMR (126 MHz, $CDCl_3$)** δ 140.9, 140.2, 139.6, 130.0, 128.9, 127.3, 127.1, 127.0, 73.0, 63.0, 56.8, 31.6, 28.1, 25.7; **HRMS (ES^+) *m/z*** $[M - OH]^+$ Calcd for $C_{19}H_{23}O$ 267.1749; found 267.1758.

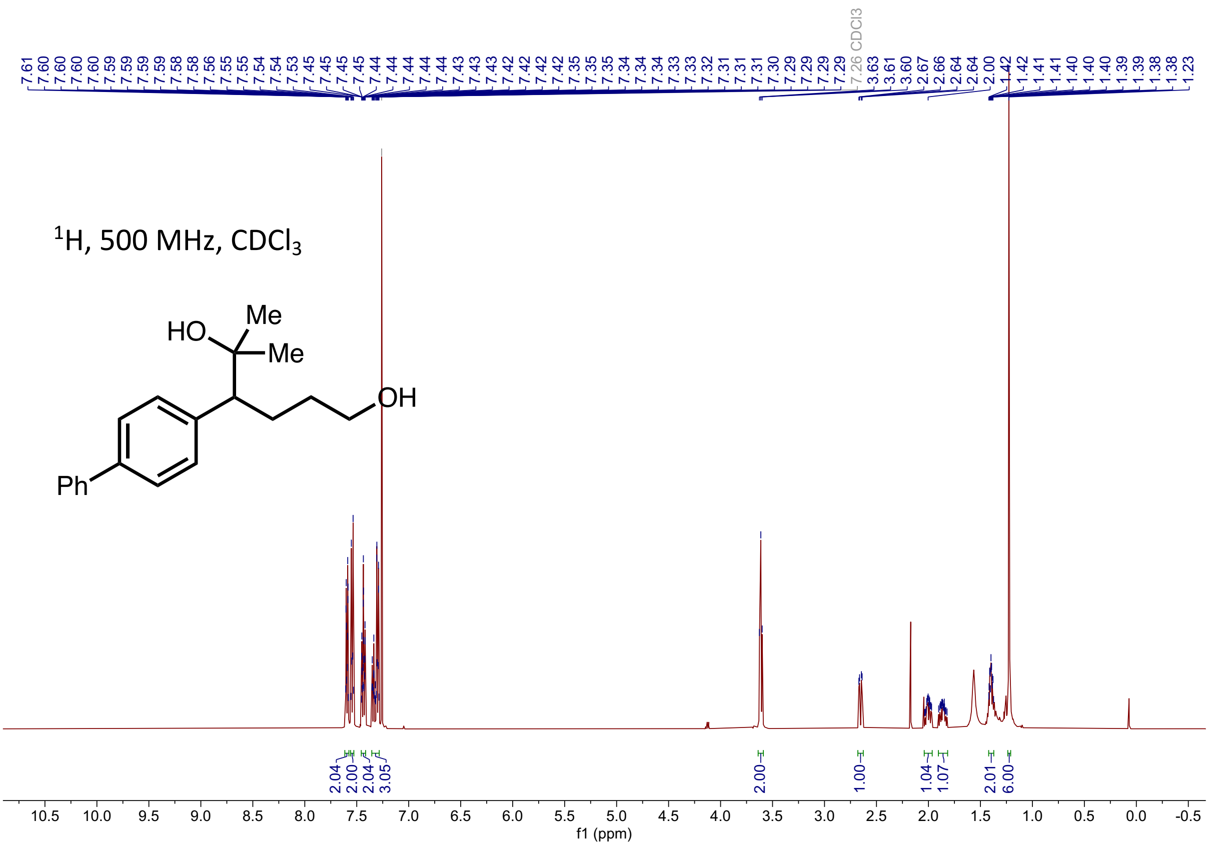

7.61 7.60 7.60 7.60 7.60 7.59 7.59 7.59 7.59 7.58 7.58 7.56 7.55 7.55 7.54 7.54 7.53 7.45 7.45 7.45 7.45 7.44 7.44 7.44 7.44 7.43 7.43 7.43 7.42 7.42 7.42 7.42 7.35 7.35 7.34 7.34 7.34 7.33 7.33 7.32 7.32 7.31 7.31 7.31 7.30 7.29 7.29 7.29 7.29 7.26 CDCl3 3.63 3.61 3.60 2.67 2.66 2.64 2.64 2.00 1.42 1.42 1.41 1.41 1.40 1.40 1.40 1.39 1.39 1.38 1.38 1.23
1H, 500 MHz, CDCl3
HO
Me
Me
OH
Ph
2.04 2.00 2.04 3.05 2.00 1.00 1.04 1.07 2.01 6.00
10.5 10.0 9.5 9.0 8.5 8.0 7.5 7.0 6.5 6.0 5.5 5.0 4.5 4.0 3.5 3.0 2.5 2.0 1.5 1.0 0.5 0.0 -0.5
f1 (ppm)

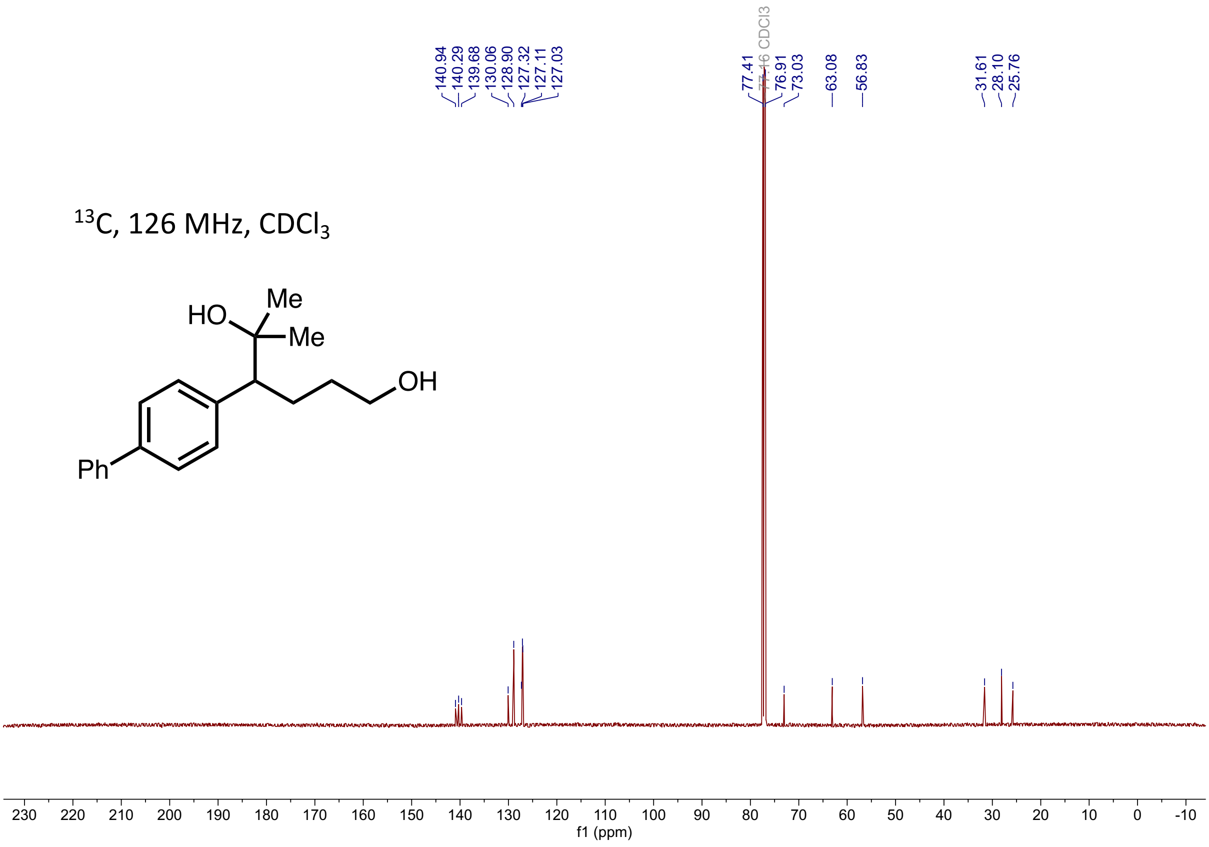

140.94 140.29 139.68 130.06 128.90 127.32 127.11 127.03
77.41 77.16 CDCl3 76.91 73.03 63.08 56.83 31.61 28.10 25.76
13C, 126 MHz, CDCl3
HO
Me
Me
OH
Ph
230 220 210 200 190 180 170 160 150 140 130 120 110 100 90 80 70 60 50 40 30 20 10 0 -10
f1 (ppm)

(S14)

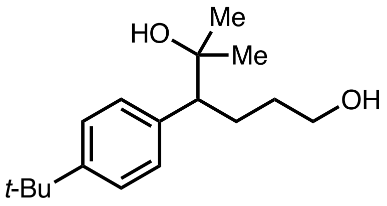

Prepared according to general procedure A, step 2 (ii) using 3-(4-(tert-butyl)phenyl)tetrahydro-2*H*-pyran-2-one (0.51 g, 2.2 mmol), THF (4.4 mL) and methylmagnesium bromide solution (1.9 mL, 5.5 mmol). The crude residue was purified by flash column chromatography (eluent = 30 to 50% EtOAc in hexanes, silica gel) to afford product as a white solid (0.29 g, 50% yield).

Mp.: 100-103 °C; **R_f** = 0.29 (eluent = 50% EtOAc in hexanes); **v_{max} / cm^{-1}** (thin film) 3281, 2970, 2930, 2870, 1364, 1140, 1065; **^{1}H NMR (500 MHz, $CDCl_3$)** δ 7.32 – 7.29 (m, 2H), 7.14 – 7.12 (m, 2H), 3.60 – 3.57 (td, *J* = 6.5, 0.6 Hz, 2H), 2.59 – 2.56 (dd, *J* = 12.0, 3.2 Hz, 1H), 1.95 – 1.90 (m, 1H), 1.85 – 1.79 (m, 1H), 1.38 – 1.34 (m, 2H), 1.31 (s, 9H), 1.20 (s, 3H), 1.17 (s, 3H); **^{13}C NMR (126 MHz, $CDCl_3$)** δ 149.6, 137.7, 129.2, 125.2, 72.9, 63.1, 56.6, 34.5, 31.6, 31.5, 28.1, 27.7, 25.7; **HRMS (ESP$^+$)** ***m/z*** [M + Na]$^+$ Calcd for $C_{17}H_{28}NaO_2$ 287.1990; found 287.1987.

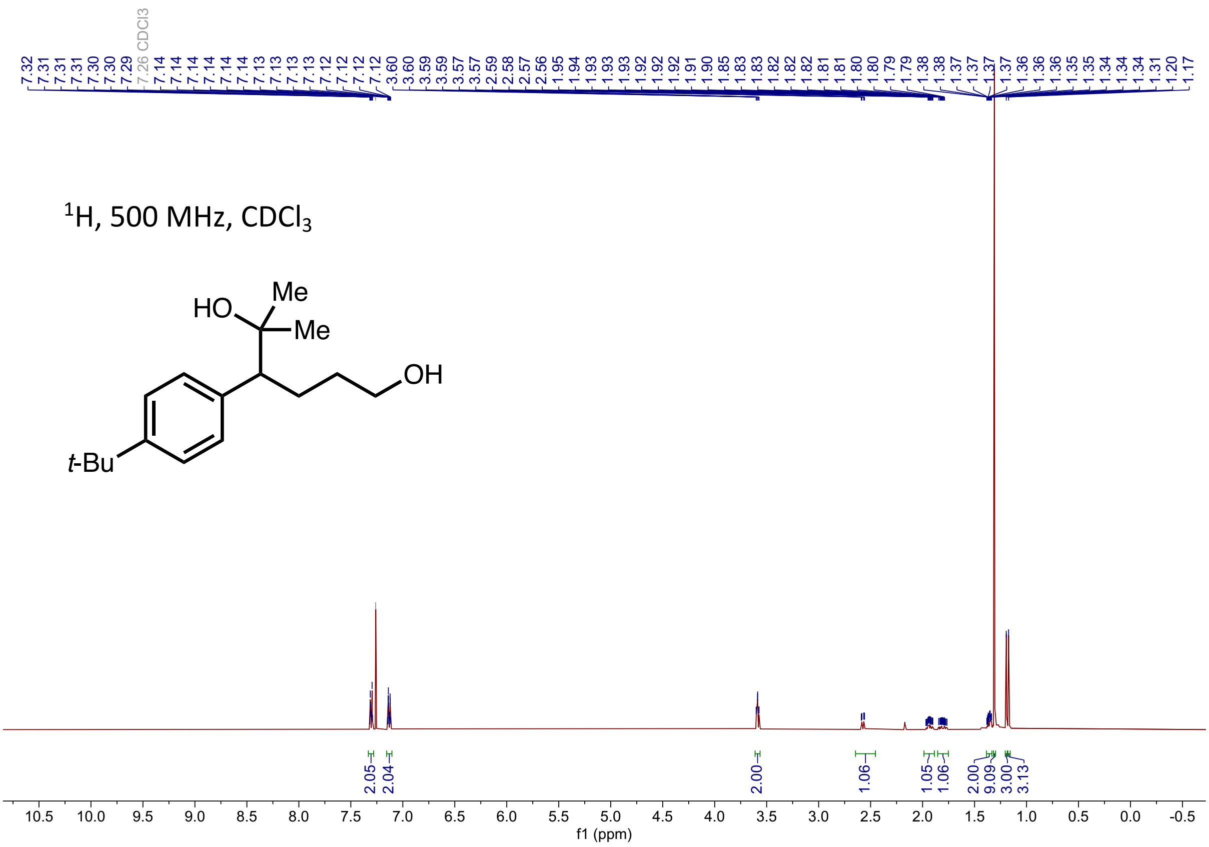

7.32 7.31 7.31 7.30 7.30 7.29 7.26 CDCl3 7.14 7.14 7.14 7.14 7.14 7.14 7.13 7.13 7.13 7.13 7.12 7.12 7.12 7.12 3.60 3.60 3.59 3.59 3.57 2.59 2.58 2.57 2.56 1.95 1.94 1.93 1.93 1.93 1.92 1.92 1.92 1.91 1.90 1.85 1.83 1.83 1.82 1.82 1.82 1.81 1.81 1.80 1.80 1.79 1.79 1.38 1.38 1.37 1.37 1.37 1.37 1.36 1.36 1.36 1.35 1.35 1.34 1.34 1.34 1.31 1.20 1.17
^{1}H, 500 MHz, CDCl$_3$
HO
Me
Me
OH
t-Bu
2.05
2.04
2.00
1.06
1.05
1.06
2.00
9.09
3.00
3.13
f1 (ppm)

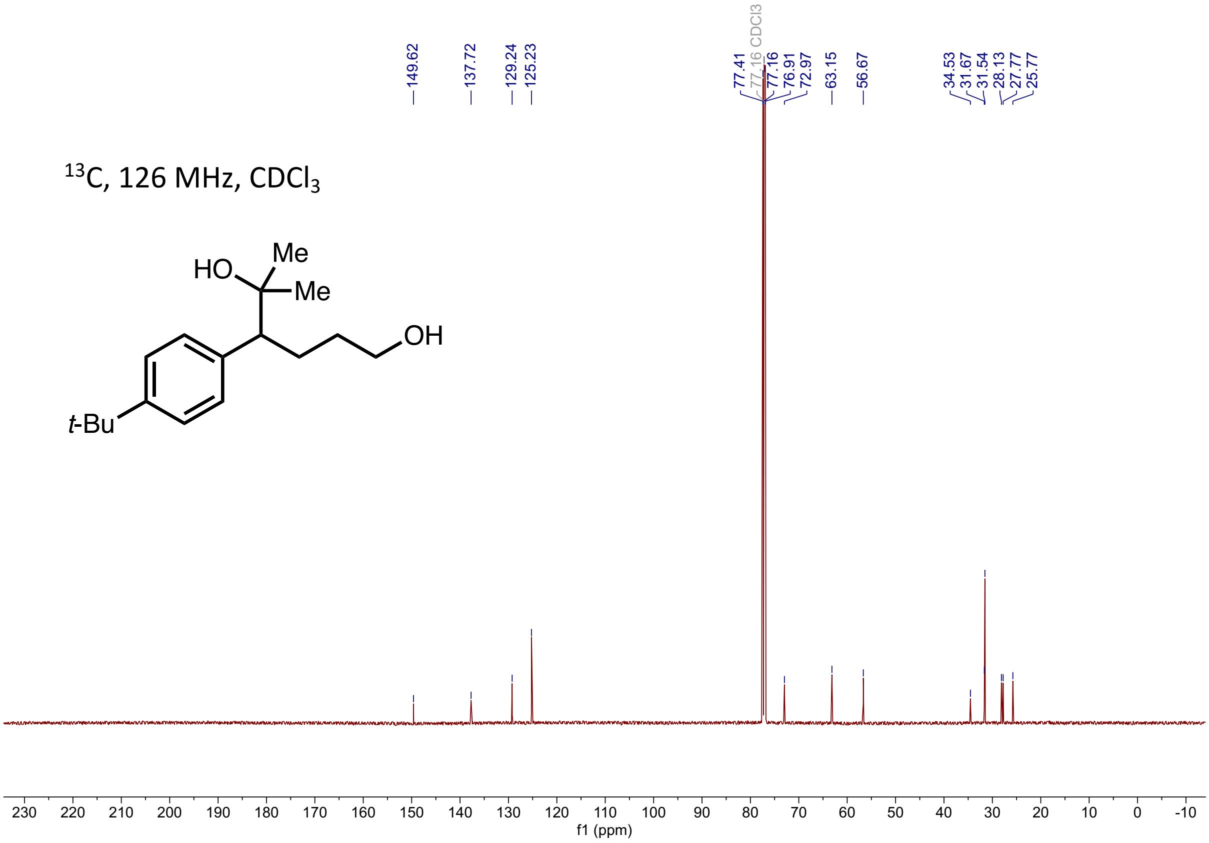

149.62
137.72
129.24
125.23
77.41 77.16 CDCl3 77.16 76.91 72.97
63.15
56.67
34.53 31.67 31.54 28.13 27.77 25.77
^{13}C, 126 MHz, CDCl$_3$
HO
Me
Me
OH
t-Bu
f1 (ppm)

(S15)

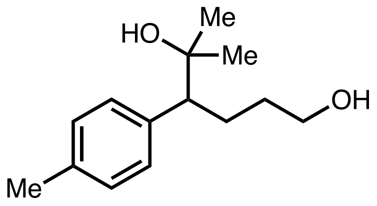

Prepared according to general procedure A, step 2 (i) using 3-(p-tolyl)tetrahydro-2*H*-pyran-2-one (1.16 g, 6.1 mmol), THF (12 mL) and methyllithium solution (8.0 mL, 12.2 mmol). The crude residue was purified by flash column chromatography (eluent = 30 to 50% EtOAc in hexanes, silica gel) to afford product as a white solid (1.1 g, 81% yield).

Mp.: 88-93 °C; **R_f** = 0.22 (eluent = 50% EtOAc in hexanes); **ν_{max} / cm^{-1}** (thin film) 3287, 2986, 2935, 2866, 1375, 1150, 1065; **^{1}H NMR (500 MHz, $CDCl_3$)** δ 7.13 – 7.09 (m, 4H), 3.59 – 3.56 (m, 2H), 2.58 – 2.55 (dd, *J* = 12.1, 3.2 Hz, 1H), 2.33 (s, 3H), 1.99 – 1.91 (m, 1H), 1.83 – 1.75 (m, 1H), 1.38 – 1.33 (m, 2H), 1.19 (s, 3H), 1.17 (s, 3H); **^{13}C NMR (126 MHz, $CDCl_3$)** δ 137.9, 136.4, 129.5, 129.1, 72.9, 63.0, 56.7, 31.6, 28.0, 27.8, 25.7, 21.1; **HRMS (CI^+) *m/z*** $[M - OH]^+$ Calcd for $C_{14}H_{21}O$ 205.1586; found 205.1589.

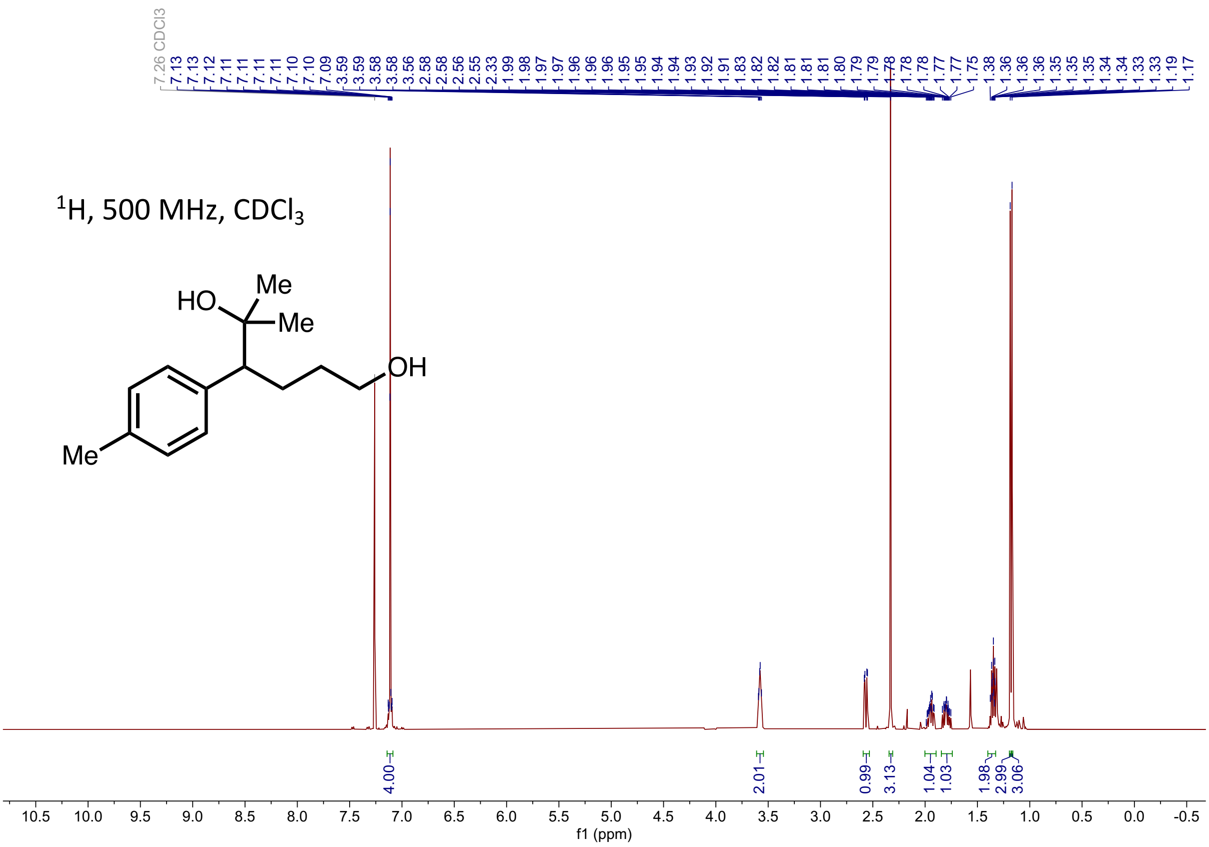
7.26 CDCl3
7.13 7.13 7.12 7.11 7.11 7.11 7.11 7.10 7.10 7.09 3.59 3.59 3.58 3.58 3.56 2.58 2.58 2.56 2.56 2.55 2.33 1.99 1.98 1.98 1.97 1.97 1.96 1.96 1.95 1.95 1.94 1.94 1.93 1.92 1.91 1.83 1.82 1.82 1.81 1.81 1.81 1.80 1.79 1.79 1.78 1.78 1.78 1.77 1.77 1.75 1.38 1.36 1.36 1.36 1.35 1.35 1.35 1.34 1.34 1.33 1.33 1.19 1.17
1H, 500 MHz, CDCl3
HO
Me
Me
OH
Me
4.00
2.01
0.99
3.13
1.04
1.03
1.98
2.99
3.06
10.5 10.0 9.5 9.0 8.5 8.0 7.5 7.0 6.5 6.0 5.5 5.0 4.5 4.0 3.5 3.0 2.5 2.0 1.5 1.0 0.5 0.0 -0.5
f1 (ppm)

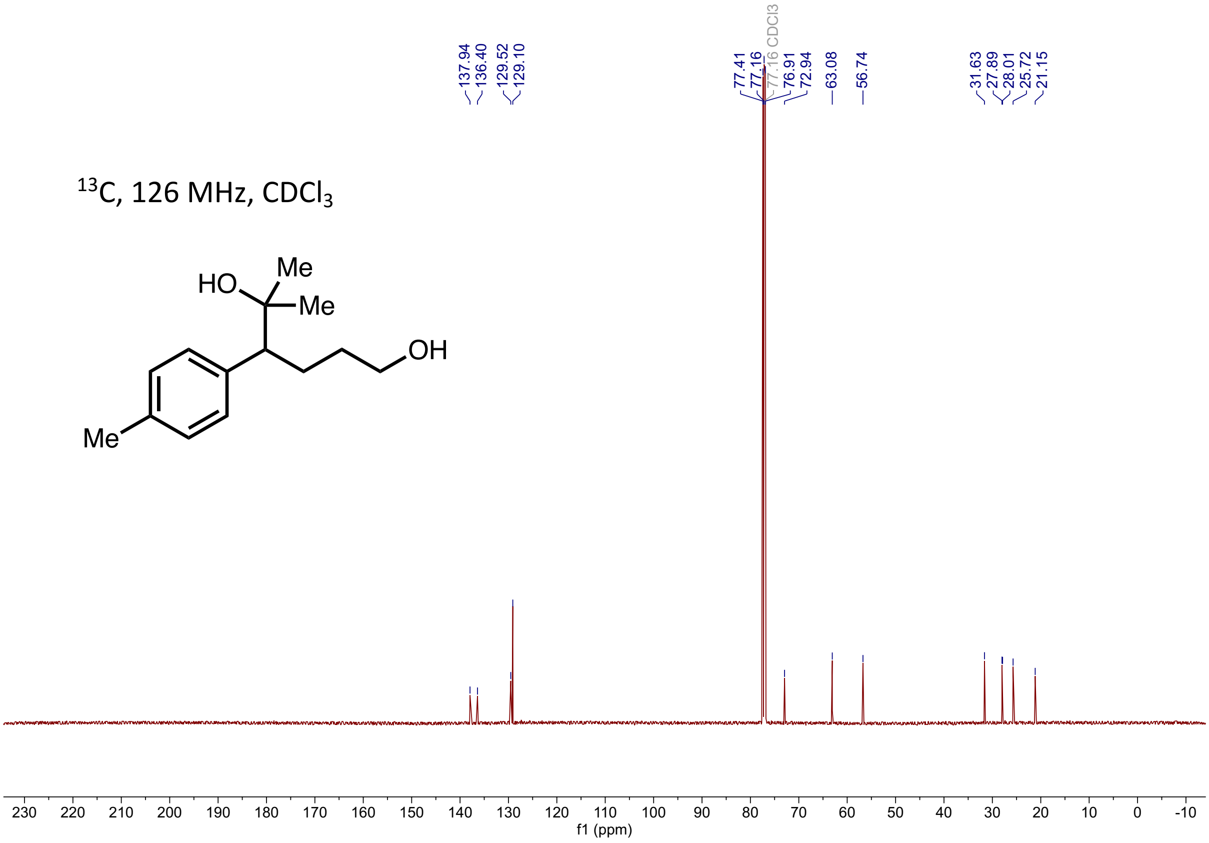
137.94 136.40 129.52 129.10
77.41 77.16 77.16 CDCl3 76.91 72.94 63.08 56.74
31.63 27.89 28.01 25.72 21.15
13C, 126 MHz, CDCl3
HO
Me
Me
OH
Me
230 220 210 200 190 180 170 160 150 140 130 120 110 100 90 80 70 60 50 40 30 20 10 0 -10
f1 (ppm)

(S16)

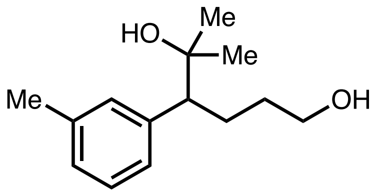

Prepared according to general procedure A, step 2 (ii) using 3-(m-tolyl)tetrahydro-2*H*-pyran-2-one (1.22 g, 6.4 mmol), THF (13 mL) and methylmagnesium bromide solution (4.3 mL, 12.8 mmol). The crude residue was purified by flash column chromatography (eluent = 30 to 50% EtOAc in hexanes, silica gel) to afford product as a white solid (1.03 g, 74% yield).

Mp.: 91-95 °C; $\mathbf{R_f}$ = 0.18 (eluent = 50% EtOAc in hexanes); $\mathbf{\nu_{max}}$ **/ cm^{-1}** (thin film) 3260, 2976, 2926, 2870, 1368, 1152, 1061; **^{1}H NMR (500 MHz, $CDCl_3$)** δ 7.21 – 7.18 (m, 1H), 7.06 – 7.01 (m, 3H), 3.59 – 3.57 (t, *J* = 6.5 Hz, 2H), 2.58 – 2.54 (dd, *J* = 12.1, 3.2 Hz, 1H), 2.34 (s, 3H), 1.96 – 1.92 (m, 1H), 1.83 – 1.79 (m, 1H), 1.37 – 1.34 (m, 2H), 1.19 (s, 3H), 1.18 (s, 3H); **^{13}C NMR (126 MHz, $CDCl_3$)** δ 141.0, 137.8, 130.5, 128.2, 127.6, 126.5, 72.9, 63.0, 57.1, 31.6, 28.0, 27.9, 25.7, 21.6; **HRMS (ESP$^+$)** ***m/z*** $[M + Na]^+$ Calcd for $C_{14}H_{22}O_2Na$ 245.1517; found 245.1517.

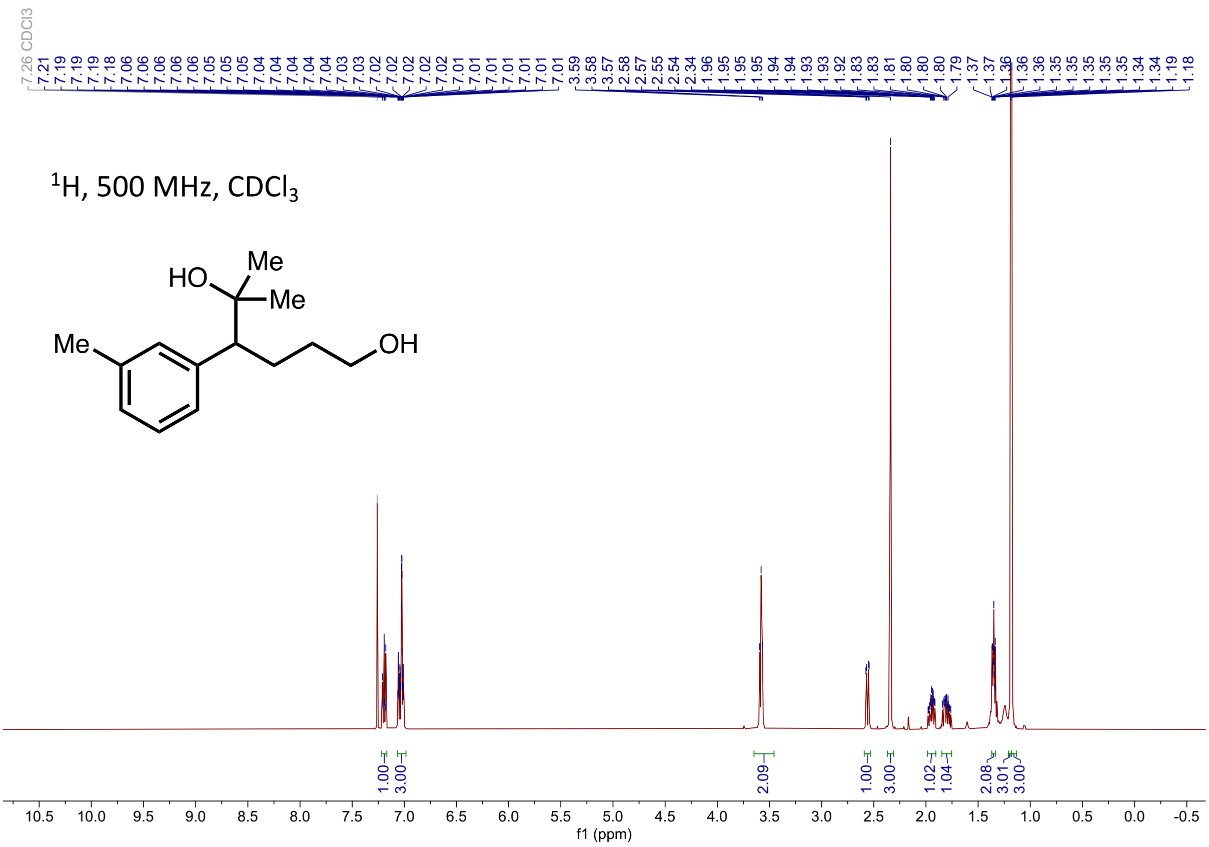
1H, 500 MHz, CDCl3
HO
Me
Me
Me
OH
7.26 CDCl3
f1 (ppm)

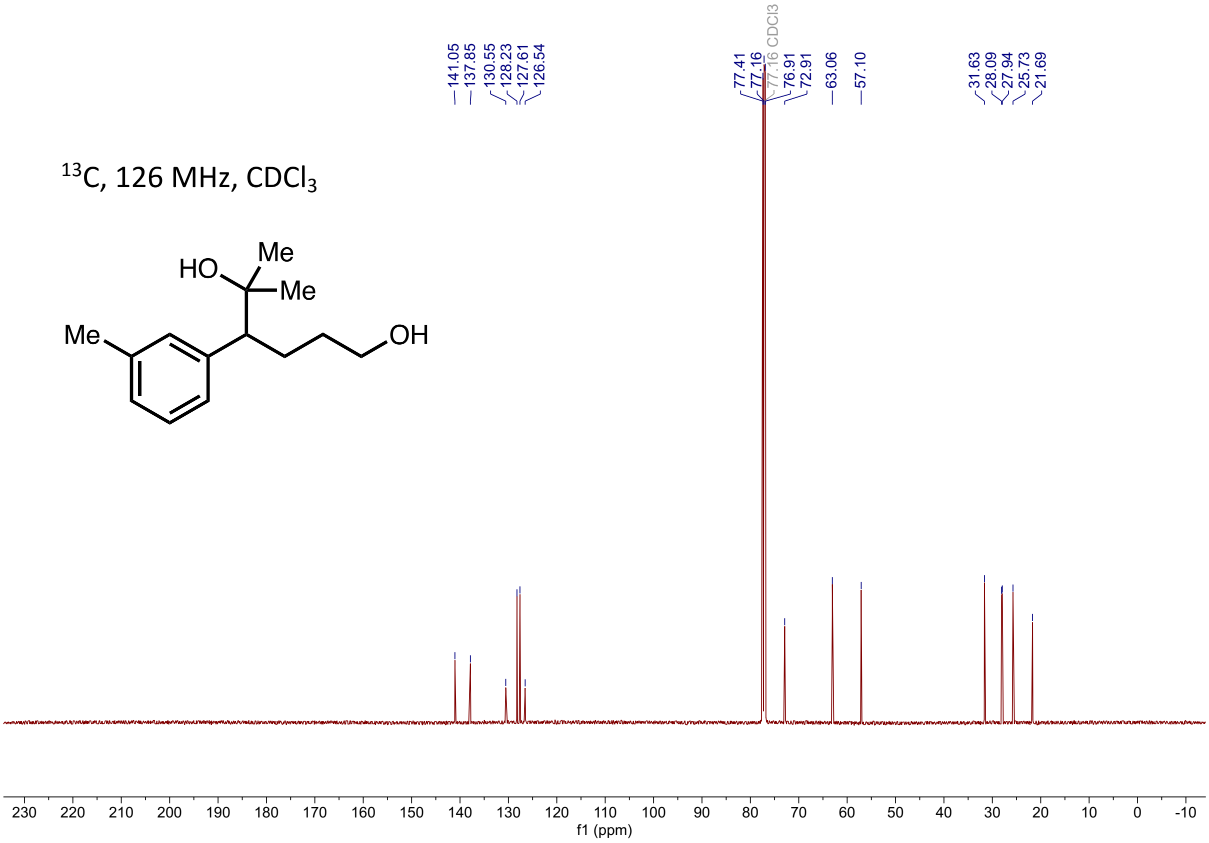
13C, 126 MHz, CDCl3
HO
Me
Me
Me
OH
141.05
137.85
130.55
128.23
127.61
126.54
77.41
77.16
77.16 CDCl3
76.91
72.91
63.06
57.10
31.63
28.09
27.94
25.73
21.69
f1 (ppm)

(S17)

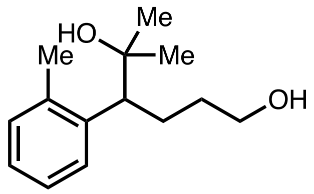

Prepared according to general procedure A, step 2 (ii) using 3-(o-tolyl)tetrahydro-2*H*-pyran-2-one (2.41 g, 12.5 mmol), THF (25 mL) and methylmagnesium bromide solution (8.3 mL, 25 mmol). The crude residue was purified by flash column chromatography (eluent = 30 to 50% EtOAc in hexanes, silica gel) to afford product as a white solid (1.8 g, 64% yield).

Mp.: 94-98 °C; $\mathbf{R_f}$ = 0.25 (eluent = 50% EtOAc in hexanes); $\mathbf{\nu_{max}}$ **/ cm^{-1}** (thin film) 3281, 2981,2916, 2874, 1379, 1151, 1062, **^{1}H NMR (500 MHz, CDCl$_3$)** δ 7.31 – 7.29 (m, 1H), 7.20 – 7.10 (m, 3H), 3.57 – 3.55 (t, *J* = 6.5 Hz, 2H), 3.04 – 3.01 (dd, *J* = 11.9, 3.3 Hz, 1H), 2.36 (s, 3H), 1.98 – 1.95 (m, 1H), 1.80 – 1.78 (m, 1H), 1.34 – 1.29 (m, 2H), 1.24 (s, 3H), 1.19 (s, 3H); **^{13}C NMR (126 MHz, CDCl$_3$)** δ 140.0, 138.1, 130.6, 127.1, 126.3, 126.1, 73.7, 63.1, 50.1, 31.4, 28.3, 27.9, 26.8, 21.0; **HRMS (CI$^+$)** ***m/z*** [M - OH]$^+$ Calcd for $C_{14}H_{21}O$ 205.1586; found 205.1588.

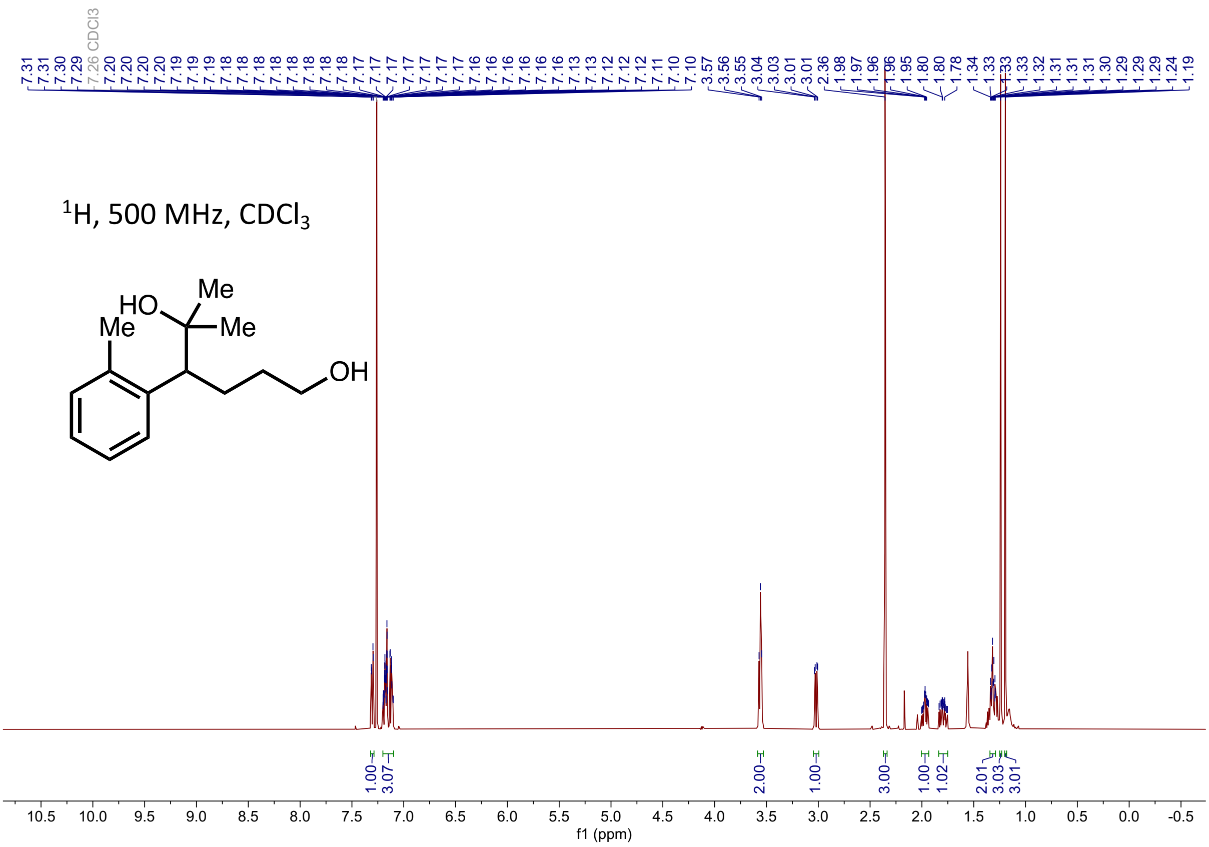

7.31 7.31 7.30 7.29 7.26 CDCl3 7.20 7.20 7.20 7.20 7.19 7.19 7.19 7.18 7.18 7.18 7.18 7.18 7.18 7.18 7.17 7.17 7.17 7.17 7.17 7.17 7.17 7.16 7.16 7.16 7.16 7.16 7.13 7.13 7.12 7.12 7.12 7.11 7.10 7.10 3.57 3.56 3.55 3.04 3.03 3.01 3.01 2.36 1.98 1.97 1.96 1.96 1.95 1.80 1.80 1.78 1.34 1.33 1.33 1.33 1.32 1.31 1.31 1.31 1.30 1.29 1.29 1.29 1.24 1.19
1H, 500 MHz, CDCl3
HO
Me
Me
Me
OH
1.00 3.07 2.00 1.00 3.00 1.00 1.02 2.01 3.03 3.01
f1 (ppm)

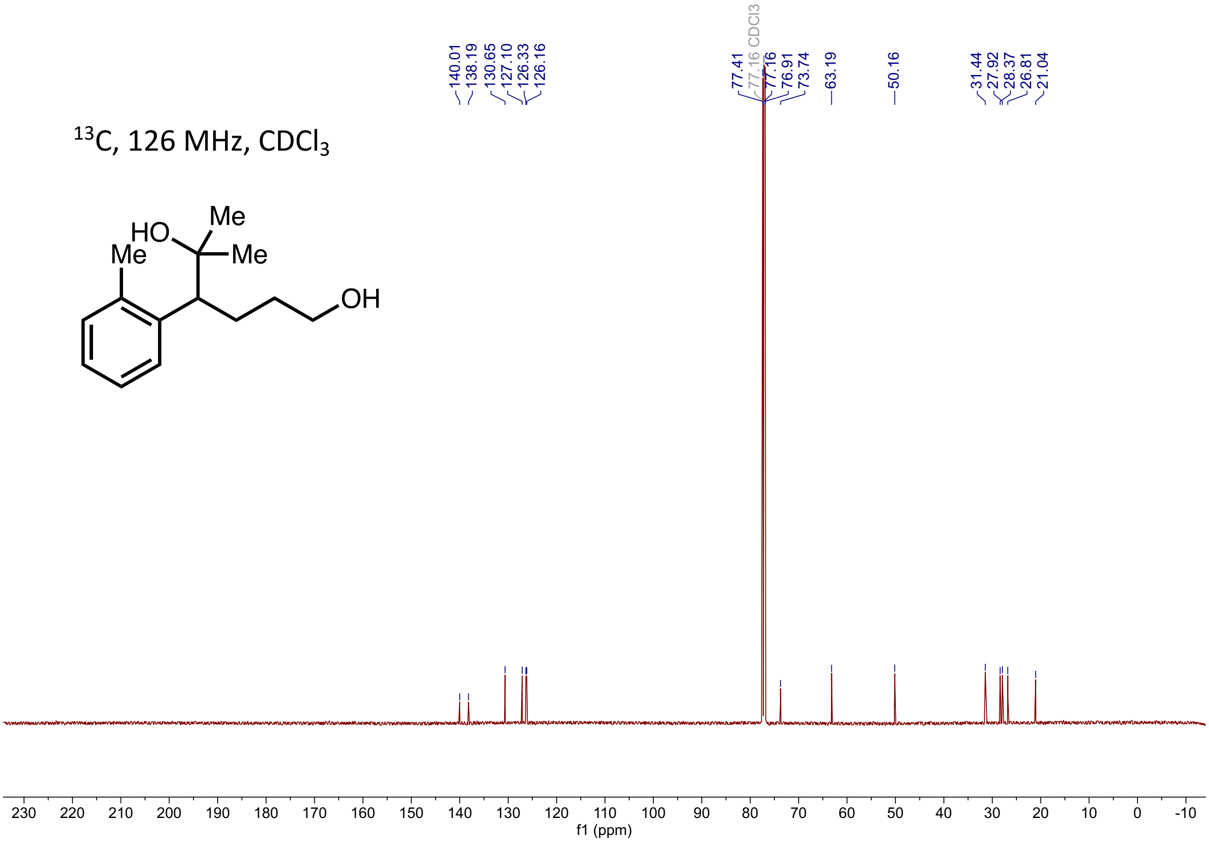

140.01 138.19 130.65 127.10 126.33 126.16 77.41 77.16 CDCl3 77.16 76.91 73.74 63.19 50.16 31.44 27.92 28.37 26.81 21.04
13C, 126 MHz, CDCl3
HO
Me
Me
Me
OH
f1 (ppm)

(S18)

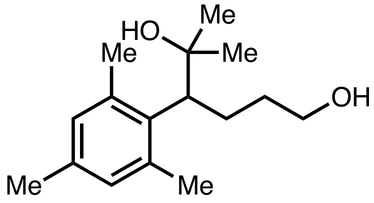

Prepared according to general procedure A, step 2 (ii) using 3-mesityltetrahydro-2H-pyran-2-one (2.2 g, 10.1 mmol), THF (21 mL) and methylmagnesium bromide solution (6.73 mL, 20.2 mmol). The crude residue was purified by flash column chromatography (eluent = 30 to 50% EtOAc in hexanes, silica gel) to afford product as a white solid (1.63 g, 65% yield).

Mp.: 102-107 °C; **R_f** = 0.30 (eluent = 50% EtOAc in hexanes); **ν_{max} / cm^{-1}** (thin film) 3439, 2981, 2924, 2855, 1375, 1150, 1063; **^{1}H NMR (500 MHz, $CDCl_3$)** δ 6.83 – 6.82 (d, *J* = 5.3 Hz, 2H), 3.61 – 3.58 (t, *J* = 6.5 Hz, 2H), 3.24 – 3.21 (dd, *J* = 11.4, 4.9 Hz, 1H), 2.49 (s, 3H), 2.31 (s, 3H), 2.23 (s, 3H), 2.12 – 2.04 (m, 1H), 1.93 – 1.88 (m, 1H), 1.39 – 1.33 (m, 5H), 1.11 (s, 3H); **^{13}C NMR (126 MHz, $CDCl_3$)** δ 138.9, 137.5, 135.4, 134.9, 131.6, 129.5, 74.3, 63.4, 50.3, 32.0, 30.4, 29.7, 24.7, 23.1, 22.6, 20.7; **HRMS (CI^+) *m/z*** $[M - OH_3]^+$ Calcd for $C_{16}H_{23}O$ 231.1743; found 231.1743.

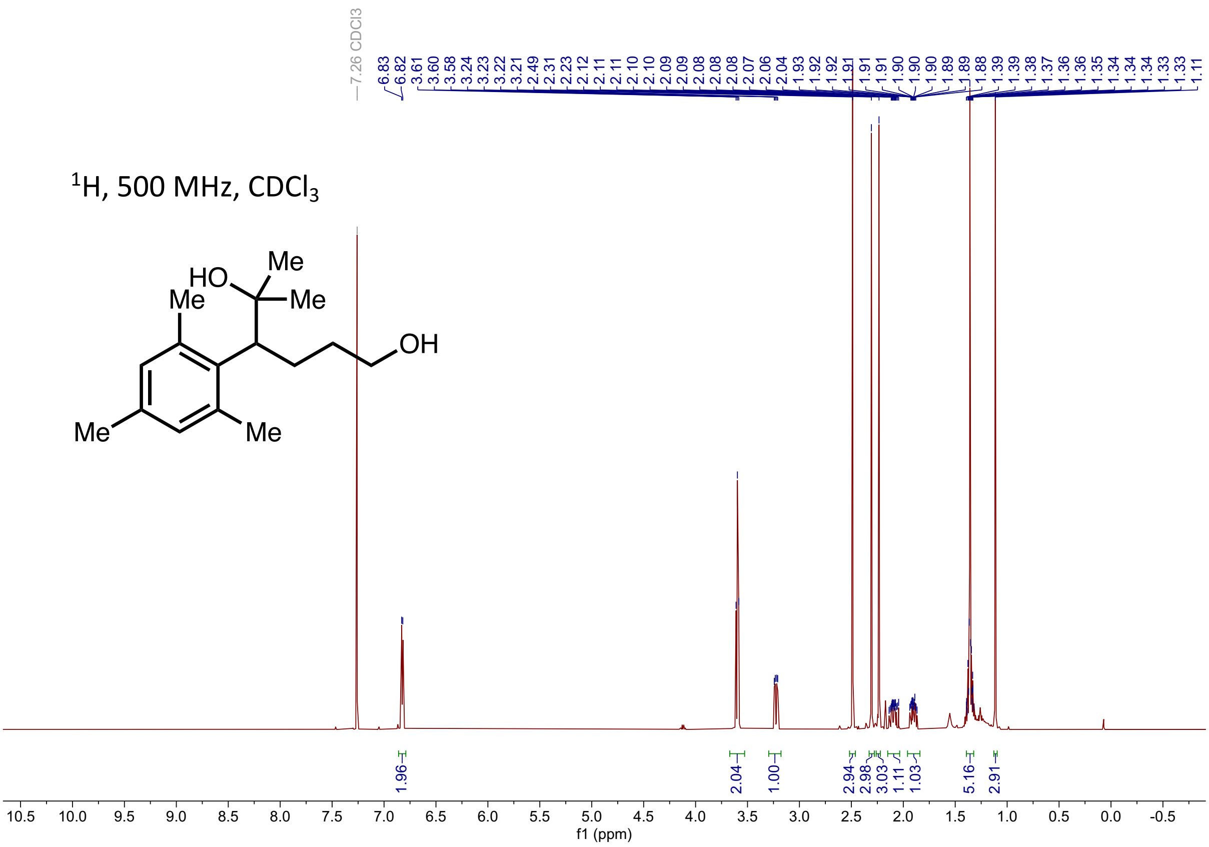

^{1}H, 500 MHz, $CDCl_3$
HO
Me
Me
Me
OH
Me
Me
7.26 CDCl3
6.83
6.82
3.61
3.60
3.58
3.24
3.23
3.22
3.21
2.49
2.31
2.23
2.12
2.11
2.11
2.10
2.10
2.09
2.09
2.08
2.08
2.08
2.07
2.06
2.04
1.93
1.92
1.92
1.91
1.91
1.91
1.91
1.90
1.90
1.90
1.89
1.89
1.88
1.39
1.39
1.38
1.37
1.36
1.36
1.35
1.34
1.34
1.34
1.33
1.33
1.11
1.96
2.04
1.00
2.94
2.98
3.03
1.11
1.03
5.16
2.91
f1 (ppm)

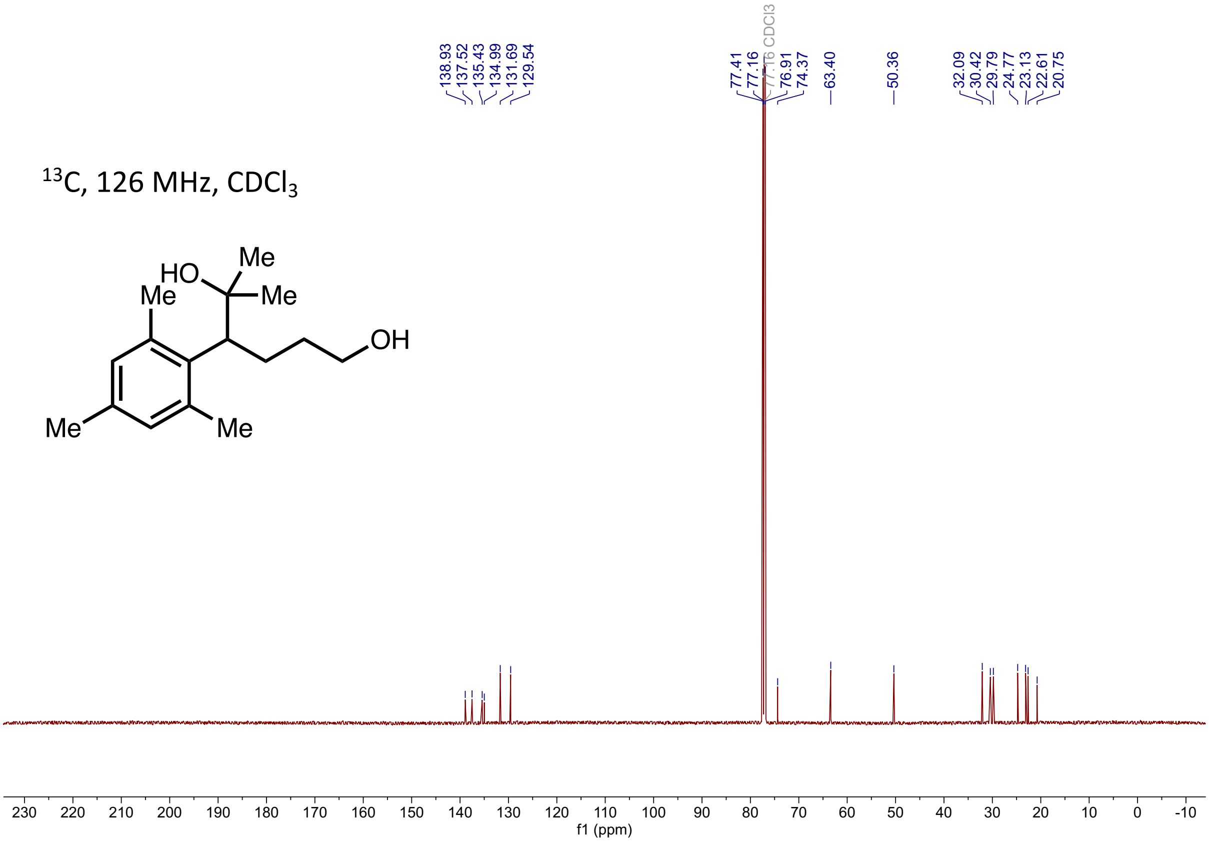

^{13}C, 126 MHz, $CDCl_3$
HO
Me
Me
Me
OH
Me
Me
138.93
137.52
135.43
134.99
131.69
129.54
77.41
77.16
77.16 CDCl3
76.91
74.37
63.40
50.36
32.09
30.42
29.79
24.77
23.13
22.61
20.75
f1 (ppm)

(S19)

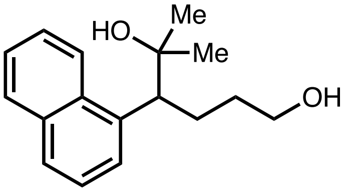

Prepared according to general procedure B, step 2 using 2-methyl-3-(naphthalene-1-yl) hex-5-en-2-ol (1.2 g, 5 mmol), THF (10 mL) and BH_3•Me_2S solution (3 mL, 6 mmol 2.0 M in hexanes). The crude residue was purified by flash column chromatography (eluent = 30 to 50% EtOAc in hexanes, silica gel) to afford product as a white solid (1.033 g, 80% yield).

Mp.: 98-102°C; $\mathbf{R_f}$ = 0.28(eluent = 50% EtOAc in Hexanes); $\mathbf{\nu_{max}}$ **/ cm^{-1}** (thin film) 3234(br), 2943, 1379, **^{1}H NMR (500 MHz, $CDCl_3$)** δ 8.23-8.21 (ddd, *J* = 8.5, 1.4, 0.7 Hz, 1H), 7.87 – 7.85 (m, 1H), 7.77 (dp, *J* = 7.9, 0.6 Hz, 1H), 7.55 – 7.26 (m, 4H), 3.73 (dd, *J* = 11.9, 3.4 Hz, 1H), 3.54 (td, *J* = 6.5, 1.0 Hz, 2H), 2.17 – 2.13 (m, 1H), 2.04 – 1.97 (m, 1H), 1.34 – 1.28 (m, 2H), 1.27 (s, 3H), 1.20 (s, 3H); **^{13}C NMR (126 MHz, $CDCl_3$)** δ 137.8, 133.9, 133.8, 128.8, 126.9, 125.8, 125.2, 125.1, 124.6, 123.7, 77.1, 77.1, 76.9, 76.6, 73.5, 62.8, 48.2, 31.1, 28.3, 28.0, 26.7; **HRMS (EI$^+$)** ***m/z*** [M - H_2O]$^+$ Calcd for $C_{17}H_{20}O$ 240.1508; found 240.1509.

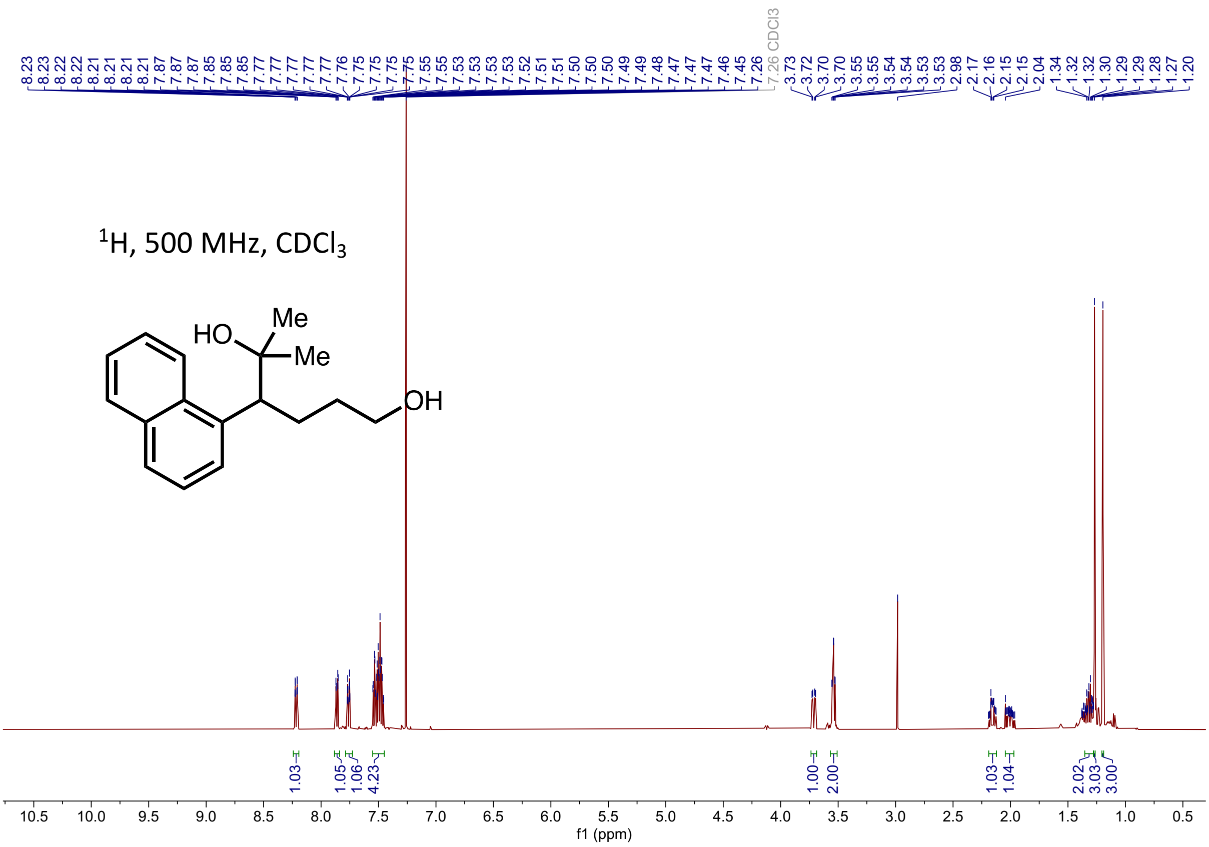

^{1}H, 500 MHz, CDCl$_3$
HO
Me
Me
OH
f1 (ppm)

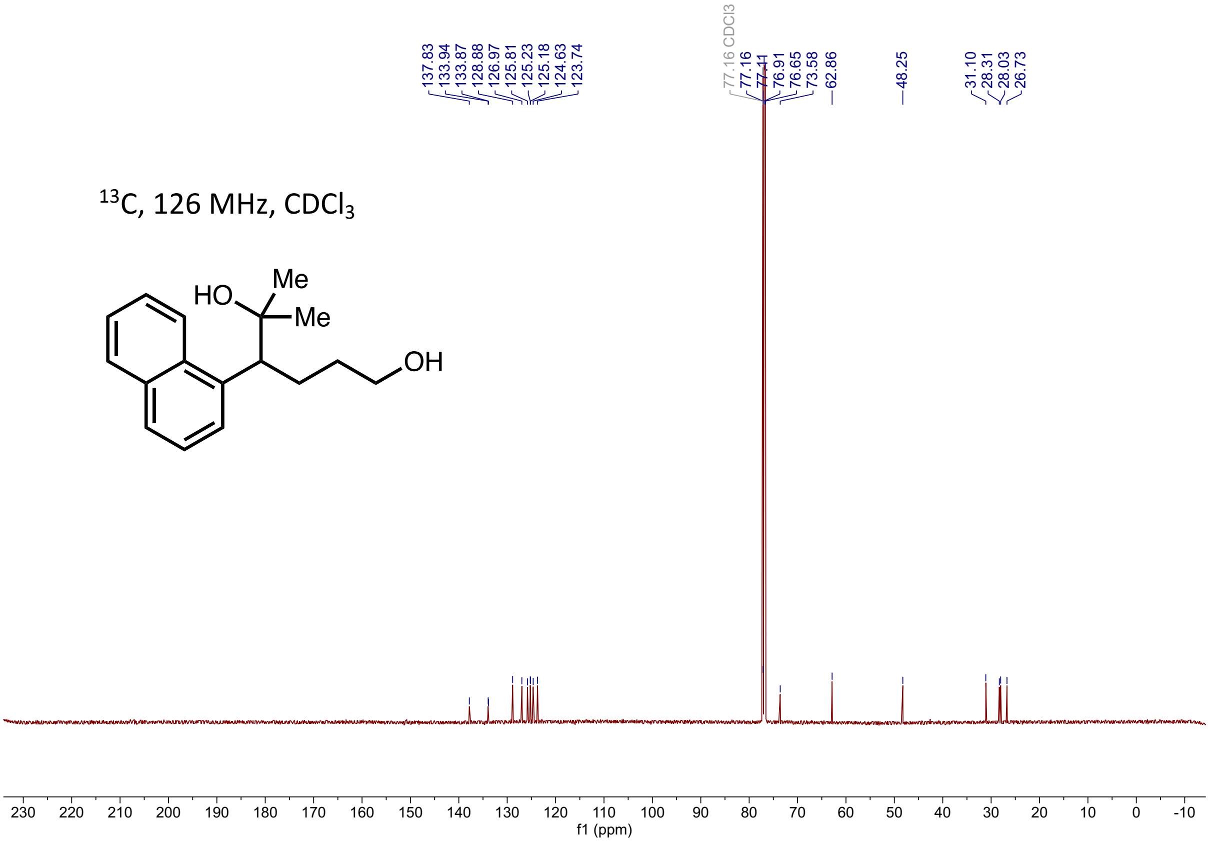

^{13}C, 126 MHz, CDCl$_3$
HO
Me
Me
OH
f1 (ppm)

(S20)

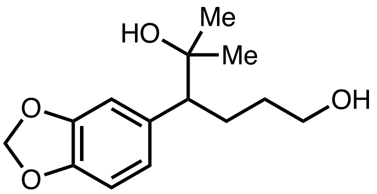

Prepared according to general procedure B, step 2 using 3-(benzo[*d*][1,3]dioxol-5-yl)-2-methylhex-5-en-2-ol (1.17 g, 5 mmol) in THF (10 mL) and BH_3•Me_2S solution (3 mL, 6 mmol, 2.0 M in hexanes). The crude residue was purified by flash column chromatography (eluent = 30 to 50% EtOAc in hexanes, silica gel) to afford product as a white solid (895.5 mg, 71% yield).

Mp.: 68-74 °C; $\mathbf{R_f}$ = 0.21 (eluent = 30% EtOAc in hexanes); $\mathbf{\nu_{max}}$ **/ cm^{-1}** (thin film) 3229 (br), 2981, 2883, 2358, 1379; **^{1}H NMR (300 MHz, $CDCl_3$)** δ 6.77 – 6.74 (m, 2H), 6.68 – 6.64 (m, 1H), 5.95 – 5.94 (m, 2H), 3.61 – 3.57 (t, *J* = 6.5 Hz, 2H), 2.54 – 2.49 (dd, *J* = 12.1, 3.2 Hz, 1H), 1.95 – 1.88 (m, 1H), 1.76 – 1.68 (m, 1H), 1.40 – 1.31 (m, 2H), 1.18 (d, 6H); **^{13}C NMR (126 MHz, $CDCl_3$)** δ 147.7, 146.4, 134.9, 122.9, 109.4, 108.1, 101.0, 72.9, 63.0, 56.7, 31.4, 28.1, 28.0, 25.8; **HRMS (EI$^+$)** ***m/z*** [M - H_2O - CH_3]$^+$ Calcd for $C_{13}H_{15}O_3$ 219.1015; found 219.1013.

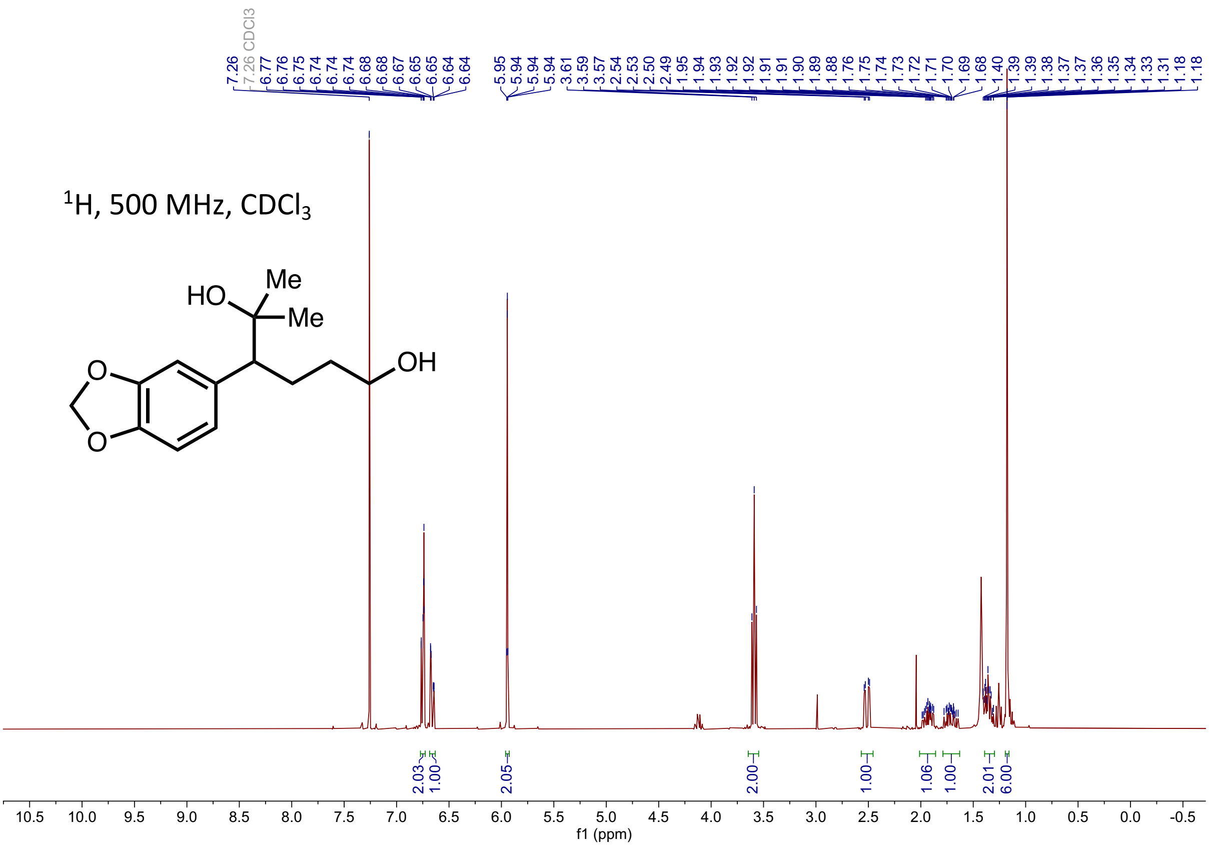

7.26
7.26 CDCl3
6.77
6.76
6.75
6.74
6.74
6.74
6.68
6.68
6.67
6.65
6.65
6.64
6.64
5.95
5.94
5.94
5.94
3.61
3.59
3.57
2.54
2.53
2.50
2.49
1.95
1.94
1.93
1.92
1.92
1.91
1.91
1.90
1.89
1.88
1.76
1.75
1.74
1.73
1.72
1.71
1.70
1.69
1.68
1.40
1.39
1.39
1.38
1.37
1.37
1.36
1.35
1.34
1.33
1.31
1.18
1.18
¹H, 500 MHz, CDCl3
HO
Me
Me
OH
O
O
2.03
1.00
2.05
2.00
1.00
1.06
1.00
2.01
6.00
f1 (ppm)

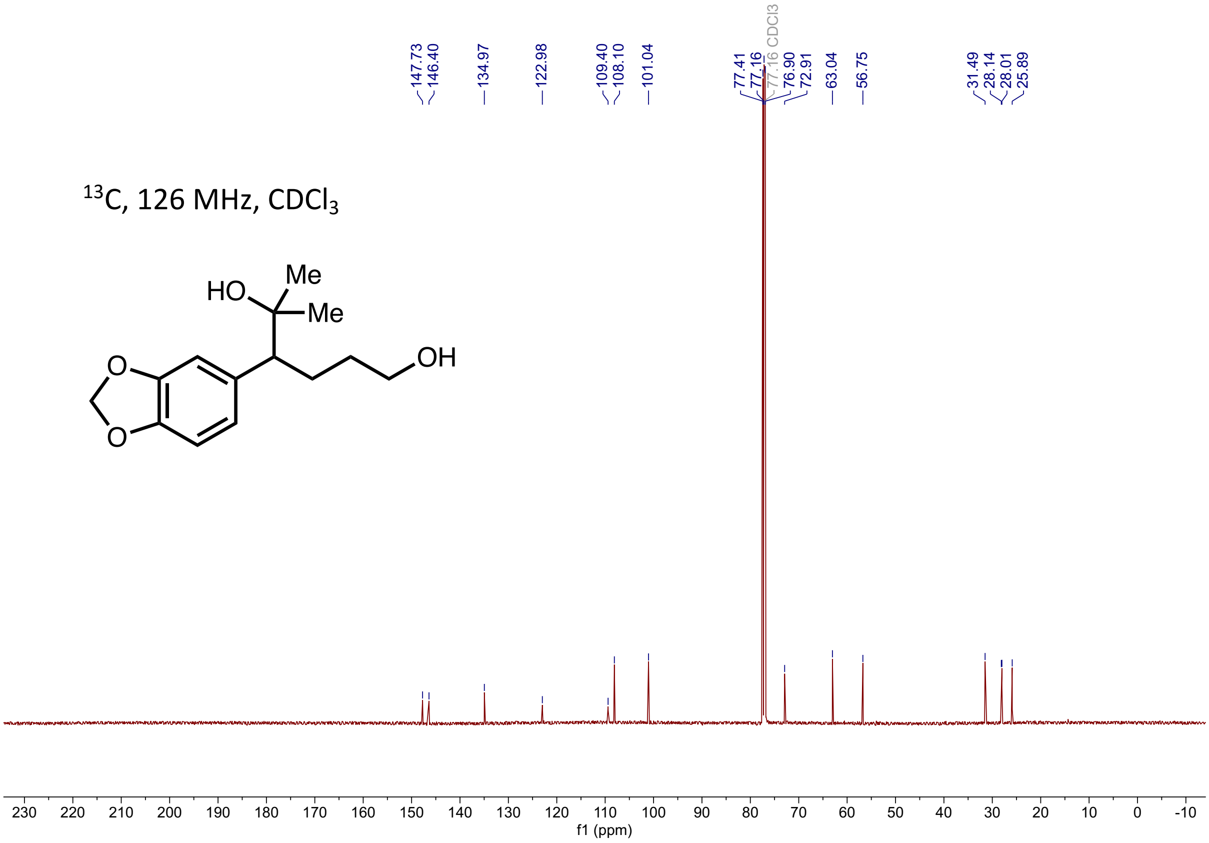

147.73
146.40
134.97
122.98
109.40
108.10
101.04
77.41
77.16
77.16 CDCl3
76.90
72.91
63.04
56.75
31.49
28.14
28.01
25.89
¹³C, 126 MHz, CDCl3
HO
Me
Me
OH
O
O
f1 (ppm)

(S21)

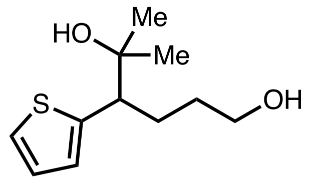

Prepared according to general procedure A, step 2 (ii) using 3-(thiophen-2-yl)tetrahydro-2*H*-pyran-2-one (1.64 g, 9 mmol), THF (18 mL) and methylmagnesium bromide solution (7.5 mL, 22.5 mmol). The crude residue was purified by flash column chromatography (eluent = 30 to 50% EtOAc in hexanes, silica gel) to afford product as a light-yellow solid (1.31 g, 68% yield).

Mp.: 57-58 °C; **R_f** = 0.13 (eluent = 50% EtOAc in hexanes); **ν_{max} / cm^{-1}** (thin film) 3246 (br), 2978, 2360, 1379; **^{1}H NMR (500 MHz, $CDCl_3$)** δ 7.21 – 7.19 (m, 1H), 6.98 – 7.96 (dd, *J* = 5.1, 3.4 Hz, 1H), 6.87 – 7.86 (m, 1H), 3.62 – 3.59 (t, *J* = 6.4 Hz, 2H), 2.93 – 2.90 (dd, *J* = 12.1, 2.9 Hz, 1H), 2.04 – 1.97 (m, 1H), 1.71 – 1.63 (m, 1H), 1.47 – 1.38 (m, 2H), 1.24 (d, *J* = 1.6 Hz, 6H); **^{13}C NMR (126 MHz, $CDCl_3$)** δ 144.5, 126.6, 126.6, 124.1, 72.5, 62.9, 52.9, 31.4, 28.0, 27.9, 27.6; **HRMS (EI$^+$) *m/z*** [M - H_2O - CH_3]$^+$ Calcd for $C_{10}H_{13}OS$ 181.0681; found 181.0679.

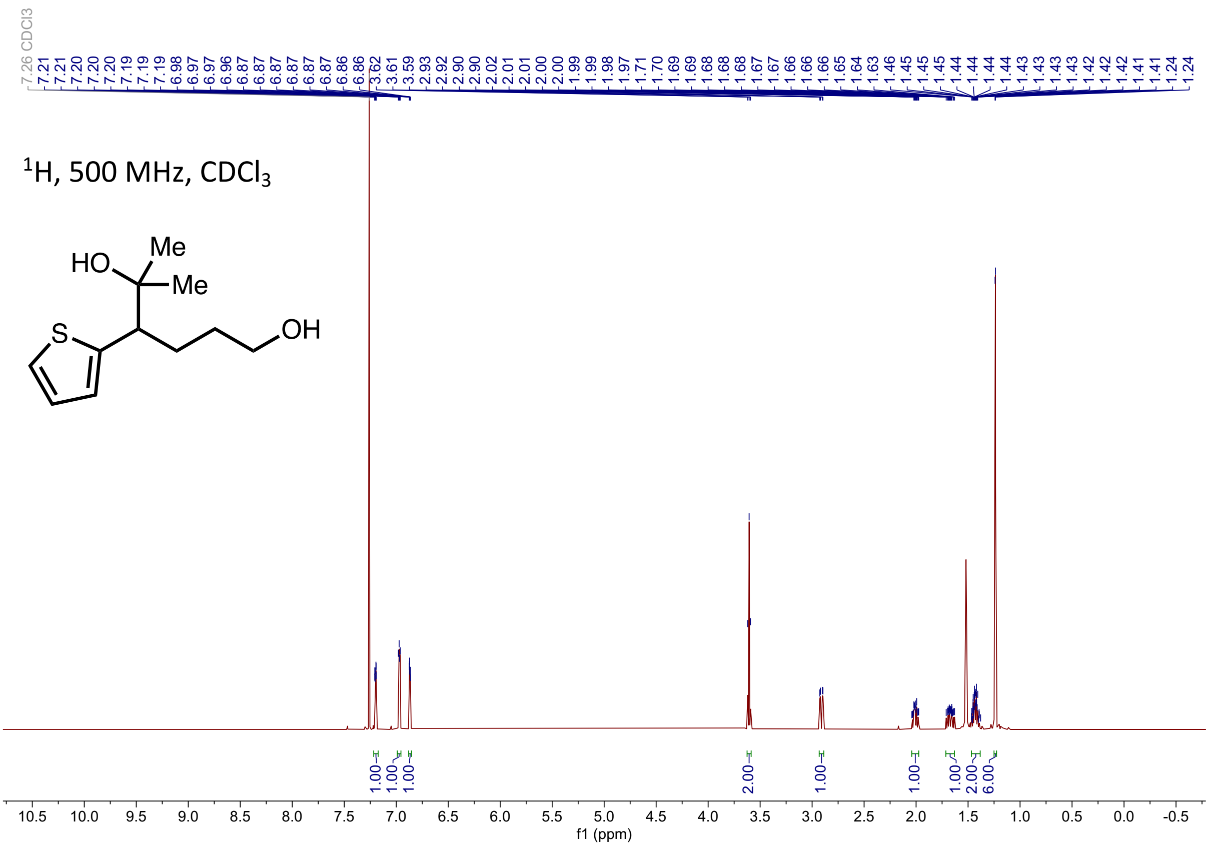
7.26 CDCl3
7.21 7.21 7.20 7.20 7.20 7.19 7.19 6.98 6.97 6.97 6.96 6.87 6.87 6.87 6.87 6.87 6.86 6.86 3.62 3.61 3.59 2.93 2.92 2.90 2.90 2.02 2.01 2.01 2.00 2.00 1.99 1.99 1.98 1.97 1.71 1.70 1.69 1.69 1.68 1.68 1.68 1.67 1.67 1.66 1.66 1.65 1.64 1.63 1.46 1.45 1.45 1.45 1.44 1.44 1.44 1.44 1.43 1.43 1.43 1.43 1.42 1.42 1.42 1.41 1.41 1.24 1.24
^{1}H, 500 MHz, $CDCl_3$
HO
Me
Me
S
OH
1.00
1.00
1.00
2.00
1.00
1.00
1.00
2.00
6.00
f1 (ppm)

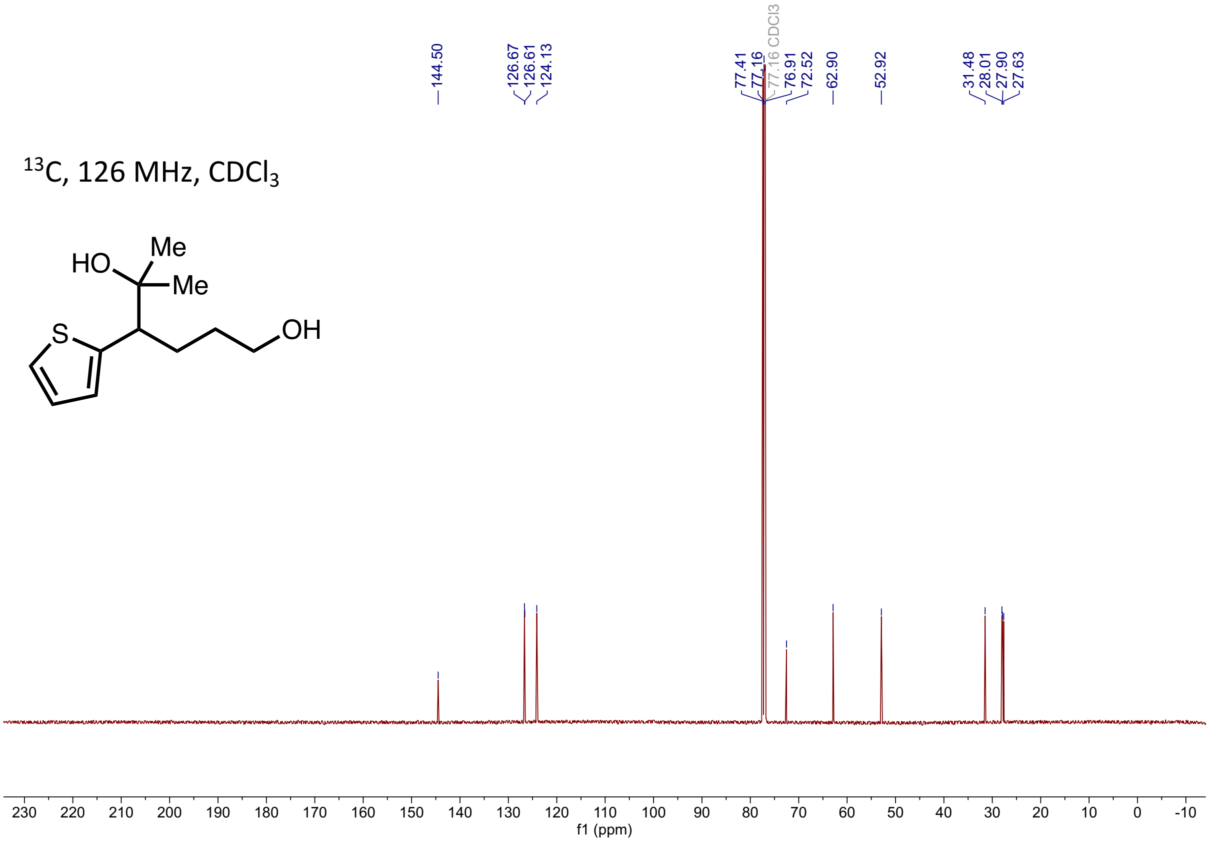
144.50
126.67 126.61 124.13
77.41 77.16 77.16 CDCl3 76.91 72.52
62.90
52.92
31.48 28.01 27.90 27.63
^{13}C, 126 MHz, $CDCl_3$
HO
Me
Me
S
OH
f1 (ppm)

(S22)

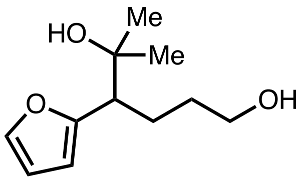

Prepared according to general procedure B, step 2 using 3-(furan-2-yl)-2-methylhex-5-en-2-ol (1.94 g, 10.8 mmol), THF (22 mL) and borane diemethylsulfide solution (6.5 mL, 13 mmol). The crude residue was purified by flash column chromatography (eluent = 30 to 80% EtOAc in hexanes, silica gel) to afford product as a pale-yellow oil (1.65 g, 77% yield).

R_f = 0.30 (eluent = 80% EtOAc in hexanes); **ν_{max} / cm^{-1}** (thin film) 3360, 2972, 2938, 2872, 1506, 1456, 1375, 1292, 1142,1011; **^{1}H NMR (500 MHz, $CDCl_3$)** δ 7.36 – 7.35 (dd, *J* = 1.8, 0.8 Hz, 1H), 6.33 – 6.32 (ddd, *J* = 3.2, 1.9, 0.3 Hz, 1H), 6.13 – 6.12 (ddd, *J* = 3.2, 0.9, 0.4 Hz, 1H), 3.61 – 3.59 (t, *J* = 6.5 Hz, 2H), 2.73 – 2.69 (dd, *J* = 11.9, 3.4 Hz, 1H), 1.89 – 1.76 (m, 2H), 1.44 – 1.38 (m, 2H), 1.20 (s, 3H), 1.19 (s, 3H); **^{13}C NMR (126 MHz, $CDCl_3$)** δ 155.6, 141.6, 110.2, 108.0, 72.7, 62.8, 50.8, 31.4, 27.8, 27.7, 24.6; **HRMS (EI$^+$) *m/z*** [M - CH_3]$^+$ Calcd for $C_{10}H_{15}O_3$ 183.1015; found 183.1011.

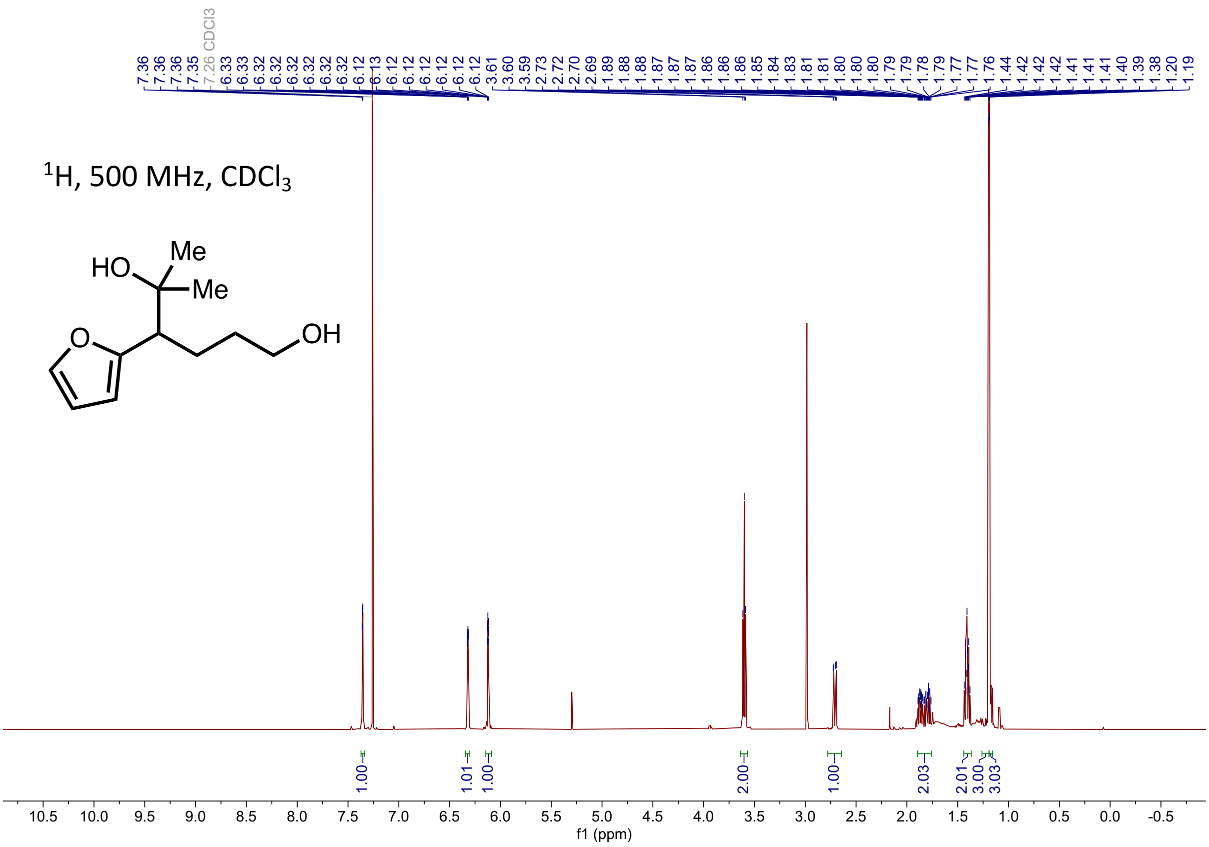
7.36 7.36 7.36 7.35 7.26 CDCl3 6.33 6.33 6.32 6.32 6.32 6.32 6.32 6.32 6.12 6.13 6.12 6.12 6.12 6.12 6.12 6.12 3.61 3.60 3.59 2.73 2.72 2.70 2.69 1.89 1.88 1.88 1.87 1.87 1.87 1.86 1.86 1.86 1.85 1.84 1.83 1.83 1.81 1.81 1.80 1.80 1.80 1.79 1.79 1.78 1.79 1.77 1.77 1.76 1.44 1.42 1.42 1.42 1.41 1.41 1.41 1.40 1.39 1.38 1.20 1.19
¹H, 500 MHz, CDCl3
HO
Me
Me
O
OH
1.00 1.01 1.00 2.00 1.00 2.03 2.01 3.00 3.03
f1 (ppm)

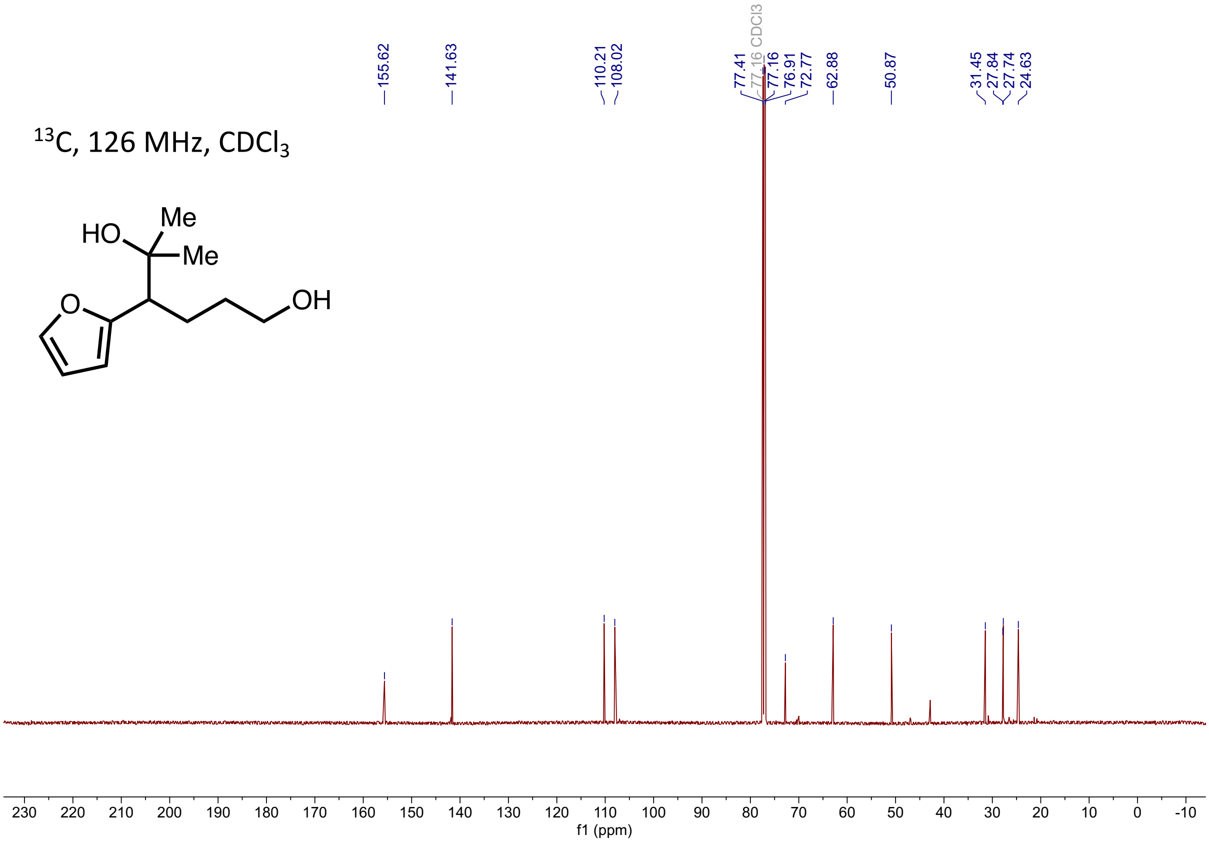
155.62 141.63 110.21 108.02 77.41 77.16 CDCl3 77.16 76.91 72.77 62.88 50.87 31.45 27.84 27.74 24.63
¹³C, 126 MHz, CDCl3
HO
Me
Me
O
OH
f1 (ppm)

(S23)

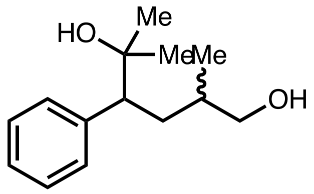

2:1 d.r.

Prepared according to general procedure B, step 2 using 2,5-dimethyl-3-phenylhex-5-en-2-ol (1.02 g, 5 mmol) in THF (10 mL) and BH_3•Me_2S solution (3 mL, 6 mmol, 2.0 M in hexanes). The crude residue was purified by flash column chromatography (eluent = 30 to 50% EtOAc in hexanes, silica gel) to afford product as a colourless oil (867 mg, 78% yield) as 2:1 mixture of diastereomers.

R_f = 0.11 (eluent = 30% EtOAc in hexanes); **ν_{max} / cm^{-1}** (thin film) 3242 (br), 2985, 1381; **HRMS (EI$^+$)** ***m/z*** [M - H_2O - CH_3]$^+$ Calcd for $C_{13}H_{17}O$ 189.1273; found 189.1273.

Selected data for major diastereomer:

^{1}H NMR (500 MHz, $CDCl_3$) δ 3.46 – 3.40 (m, 1H), 3.38 – 3.37 (m, 1H), 2.76 – 2.73 (dd, *J* = 11.9, 3.0 Hz, 1H), 1.97 – 1.92 (m, 1H), 1.72 – 1.65 (m, 1H), 1.39 – 1.32 (m, 1H), 1.18 – 1.16 (m, 6H), 0.86 – 0.84 (m, 3H); **^{13}C NMR (126 MHz, $CDCl_3$)** δ 141.7, 129.6, 128.2, 126.7, 73.5, 68.9, 54.7, 33.4, 28.7, 26.4, 18.6.

Selected data for minor diastereomer:

^{1}H NMR (500 MHz, $CDCl_3$) δ 3.51 – 3.50 (d, *J* = 5.1 Hz, 1H), 3.49 – 3.48 (d, *J* = 5.2 Hz, 1H), 2.73 – 2.70 (m, 1H), 2.05 – 1.99 (m, 1H), 1.55 – 1.49 (m, 1H), 1.30 – 1.24 (m, 1H), 1.12 (s, 6H), 0.82 – 0.80 (d, *J* = 6.7 Hz, 3H); **^{13}C NMR (126 MHz, $CDCl_3$)** δ 141.0, 129.0, 128.2, 126.7, 73.1, 66.2, 54.0, 32.7, 28.0, 27.8, 15.5.

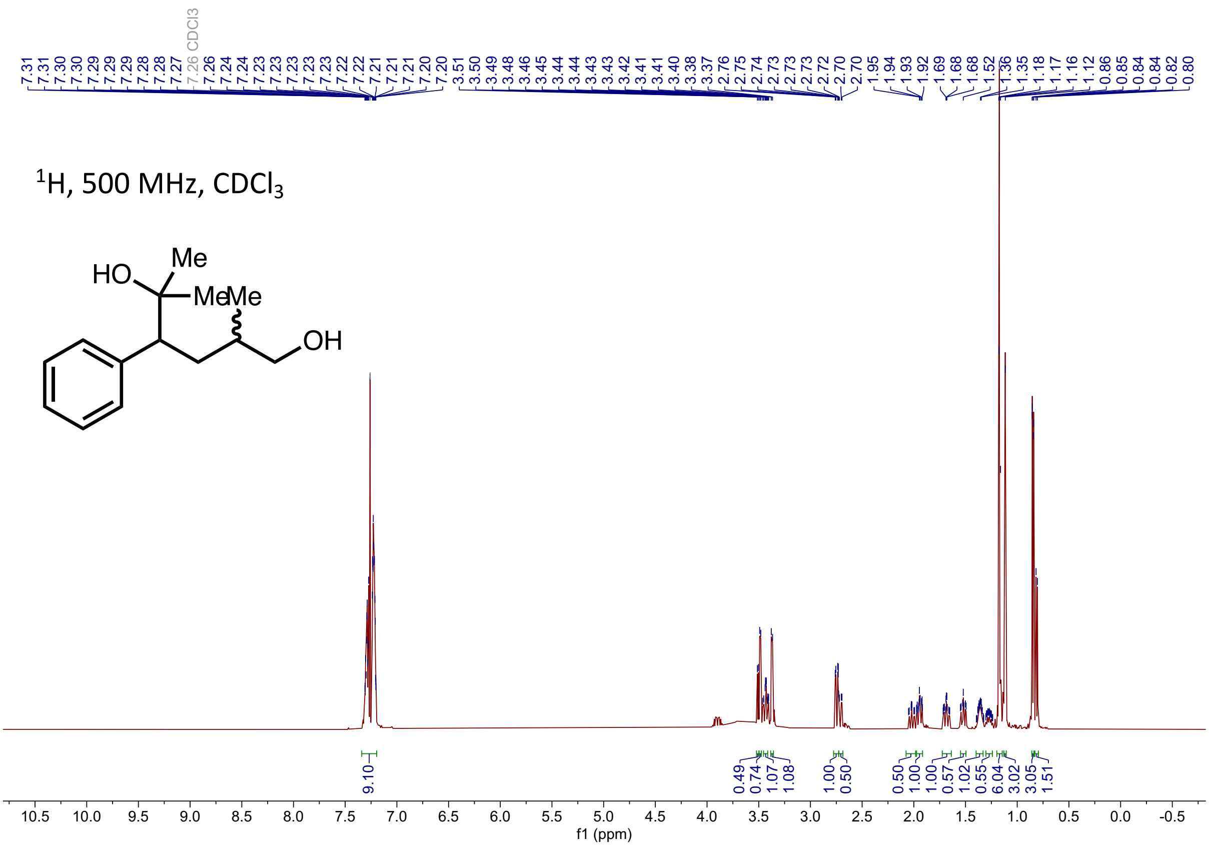

7.31 7.31 7.30 7.30 7.29 7.29 7.29 7.28 7.28 7.27 7.26 CDCl3 7.26 7.24 7.24 7.23 7.23 7.23 7.23 7.23 7.22 7.22 7.21 7.21 7.21 7.20
3.51 3.50 3.49 3.48 3.46 3.45 3.44 3.44 3.43 3.43 3.42 3.41 3.41 3.40 3.38 3.37 2.76 2.75 2.74 2.73 2.73 2.73 2.72 2.70 2.70 1.95 1.94 1.93 1.92 1.69 1.68 1.68 1.52 1.36 1.35 1.18 1.17 1.16 1.12 0.86 0.85 0.84 0.84 0.82 0.80
^{1}H, 500 MHz, CDCl$_3$
HO
Me
MeMe
OH
9.10
0.49 0.74 1.07 1.08
1.00 0.50
0.50 1.00 1.00 0.57 1.02 0.55 6.04 3.02 3.05 1.51
10.5 10.0 9.5 9.0 8.5 8.0 7.5 7.0 6.5 6.0 5.5 5.0 4.5 4.0 3.5 3.0 2.5 2.0 1.5 1.0 0.5 0.0 -0.5
f1 (ppm)

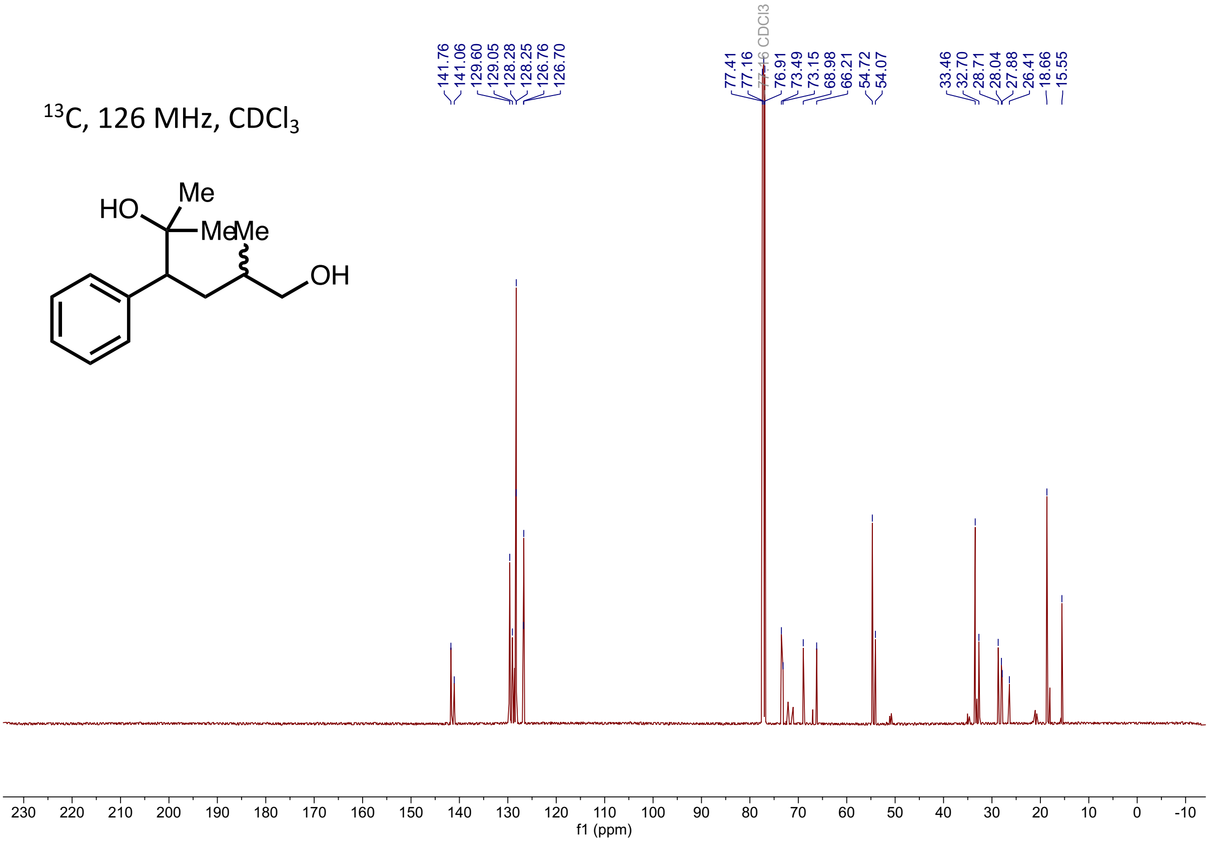

141.76 141.06 129.60 129.05 128.28 128.25 126.76 126.70
77.41 77.16 77.16 CDCl3 76.91 73.49 73.15 68.98 66.21 54.72 54.07
33.46 32.70 28.71 28.04 27.88 26.41 18.66 15.55
^{13}C, 126 MHz, CDCl$_3$
HO
Me
MeMe
OH
230 220 210 200 190 180 170 160 150 140 130 120 110 100 90 80 70 60 50 40 30 20 10 0 -10
f1 (ppm)

(S24)

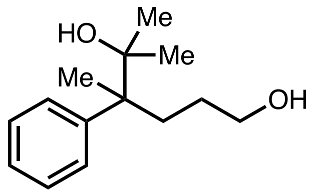

Prepared according to general procedure A, step 2 (ii) using 3-methyl- 3-phenyltetrahydro-2*H*-pyran-2-one (0.71g, 3.7mmol), THF (7.4 mL) and methylmagnesium bromide (2,7mL, 8.14 mmol). The crude residue was purified by flash column chromatography (eluent = 30 to 50% EtOAc in hexanes, silica gel) to afford product as a white solid (0.49 g, 70% yield).

Mp.: 72-75 °C; **R_f** = 0.42 (eluent = 50% EtOAc in Hexanes); **v_{max} / cm^{-1}** (thin film) 3261 (br), 2981, 1384, **^{1}H NMR (500 MHz, $CDCl_3$)** δ 7.38 – 7.34 (m, 2H), 7.33 – 7.30 (m, 2H), 7.23 (ddt, *J* = 7.7, 6.6, 1.3 Hz, 1H), 3.62 (t, *J* = 6.5 Hz, 2H), 2.33 – 2.32 (m, 1H), 1.67 (ddd, *J* = 13.6, 12.1, 5.0 Hz, 1H), 1.44 – 1.41 (m, 1H), 1.40 (d, *J* = 0.8 Hz, 3H), 1.25 – 1.19 (m, 1H), 1.18 (s, 3H), 1.09 (s, 3H); **^{13}C NMR (126 MHz, $CDCl_3$)** δ 143.3, 128.8, 127.9, 126.1, 76.9, 75.0, 63.8, 48.5, 31.0, 28.3, 25.9, 25.7, 21.1; **HRMS (EI$^+$)** ***m/z*** [M - H_2O - CH_3]$^+$ Calcd for $C_{13}H_{17}O$ 189.1273; found 189.1272.

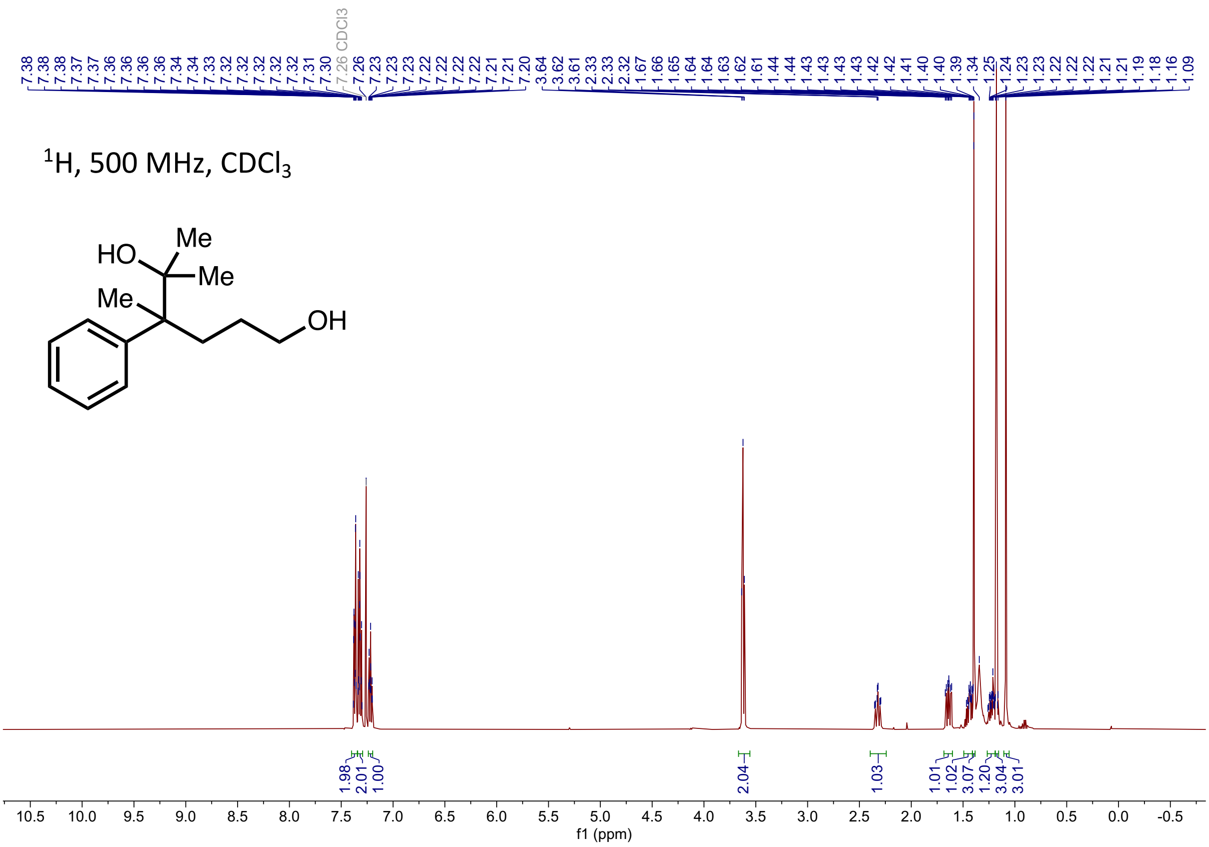
1H, 500 MHz, CDCl3
HO
Me
Me
Me
OH
f1 (ppm)

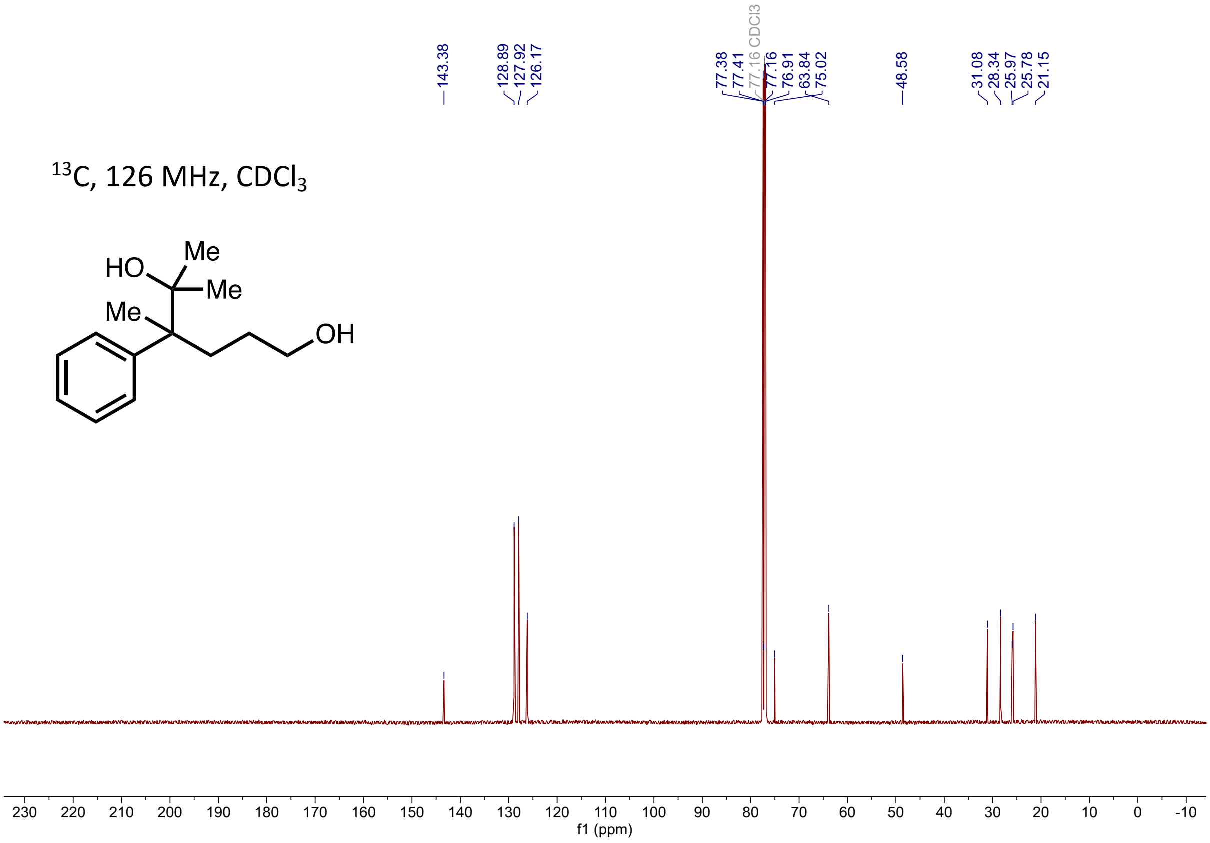
143.38
128.89
127.92
126.17
77.38
77.41
77.16 CDCl3
77.16
76.91
63.84
75.02
48.58
31.08
28.34
25.97
25.78
21.15
13C, 126 MHz, CDCl3
HO
Me
Me
Me
OH
f1 (ppm)

(S25)

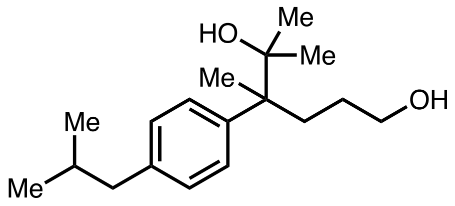

Prepared according to general procedure B, step 2 using 3-(4-isobutylphenyl)-2,3-dimethylhex-5-en-2-ol (1 g, 3.8mmol), THF (8 mL) and BH_3•Me_2S solution (2.3 mL, 4.6 mmol, 2.0 M). The crude residue was purified by flash column chromatography (eluent = 30 to 50% EtOAc in hexanes, silica gel) to afford product as white solid (700 mg, 65% yield).

Mp.: 75-78 °C; **R_f** = 0.47(eluent = 50% EtOAc in hexanes); **ν_{max} / cm^{-1}** (thin film) 3242 (br), 2980, 1381; **^{1}H NMR (300 MHz, $CDCl_3$)** δ 7.26 – 7.07 (m, 4H), 3.65 (t, *J* = 6.5 Hz, 2H), 2.43 (d, *J* = 7.2 Hz, 2H), 2.29 (td, *J* = 12.9, 3.8 Hz, 1H), 1.87 – 1.83 (m, 1H), 1.62 – 1.56 (m, 2H), 1.44 – 1.40(m, 1H), 1.37 (d, *J* = 0.7 Hz, 3H), 1.17 (s, 3H), 1.08 (s, 3H), 0.91 (s, 3H), 0.88 (s, 3H) ; **^{13}C NMR (75 MHz, $CDCl_3$)** δ 140.3, 139.5, 129.3, 128.6, 128.5, 127.0, 75.0, 63.9, 48.2, 45.0, 31.0, 30.2, 28.3, 25.9, 25.7, 22.6, 22.5, 21.1; **HRMS (EI^+) *m/z*** $[M - H_2O - CH_3]^+$ Calcd for $C_{17}H_{25}O$ 245.1899; found 245.1895.

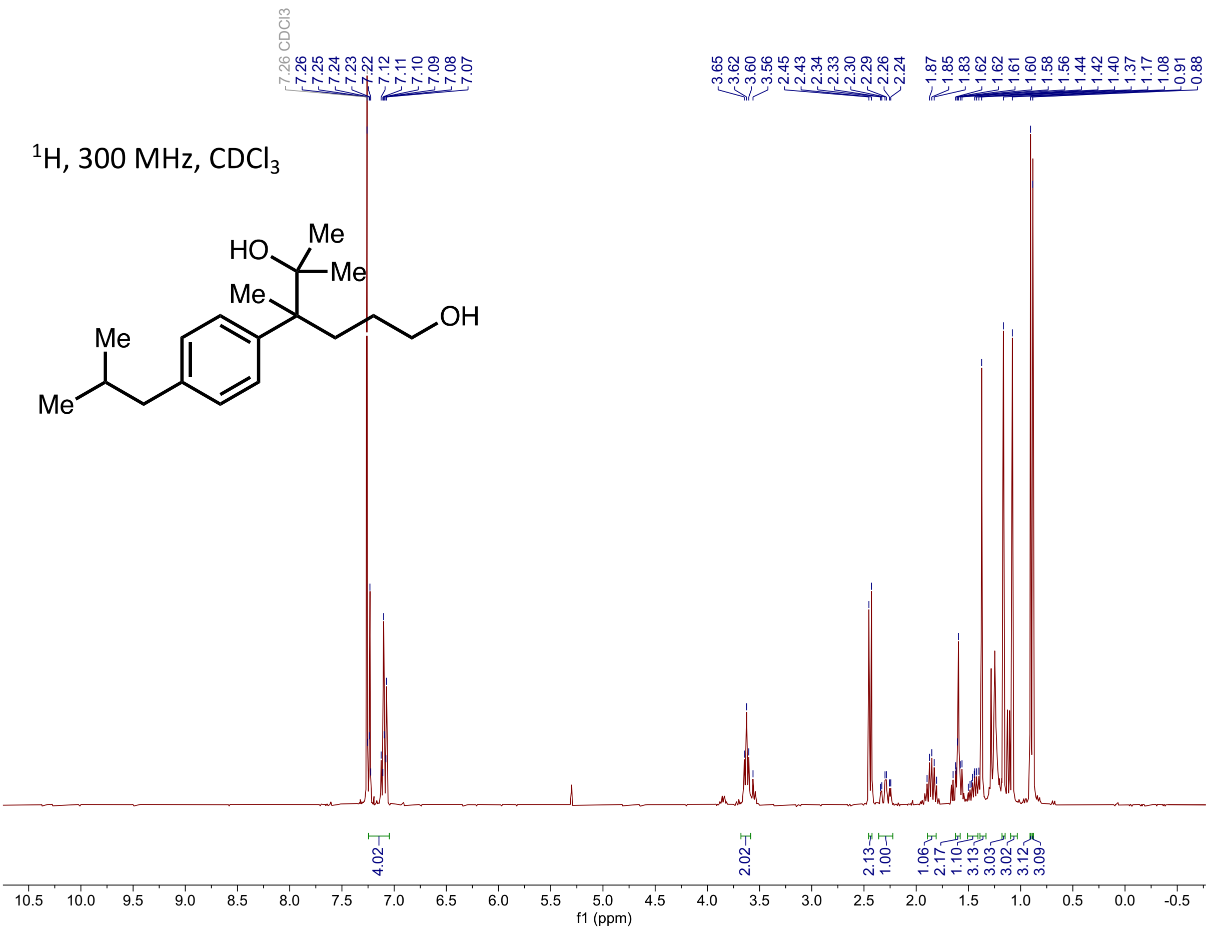
^{1}H, 300 MHz, $CDCl_3$
HO
Me
Me
Me
OH
Me
Me
f1 (ppm)

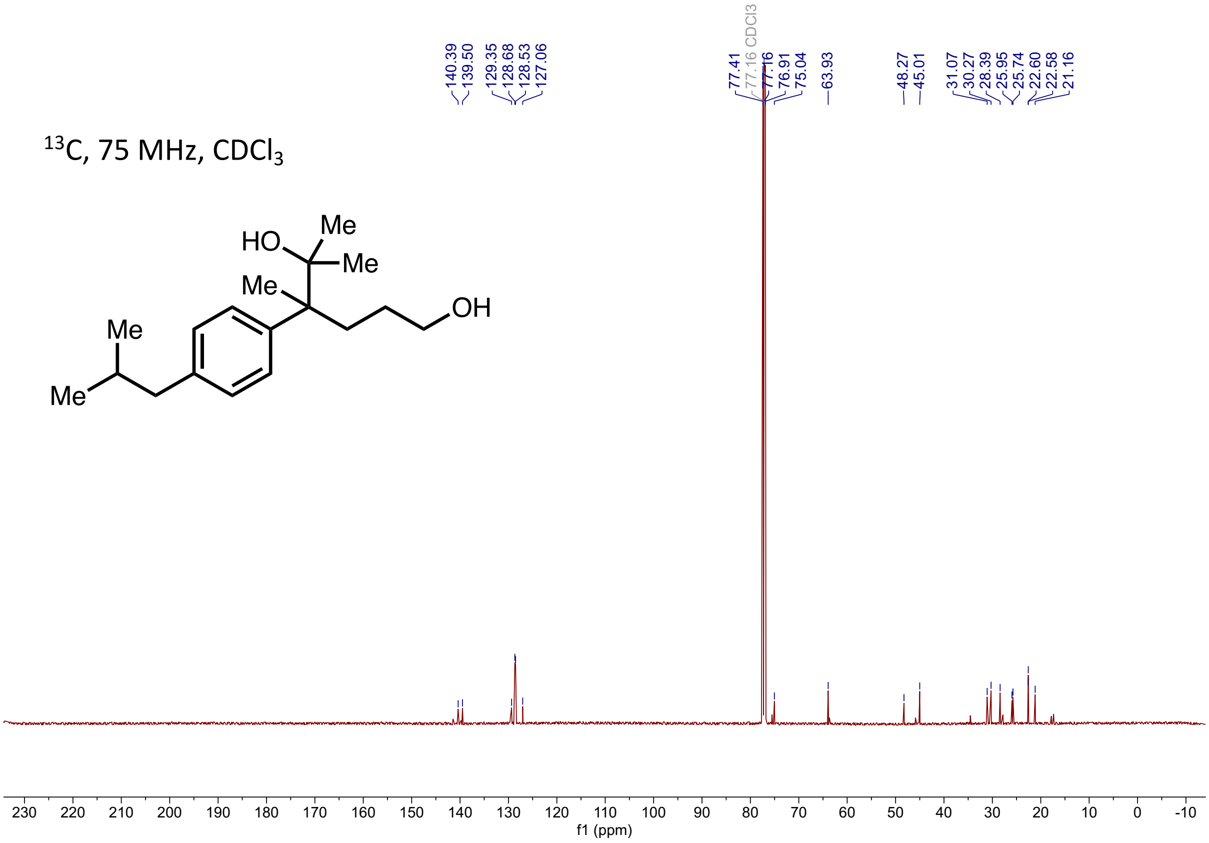
^{13}C, 75 MHz, $CDCl_3$
HO
Me
Me
Me
OH
Me
Me
f1 (ppm)

(S26)

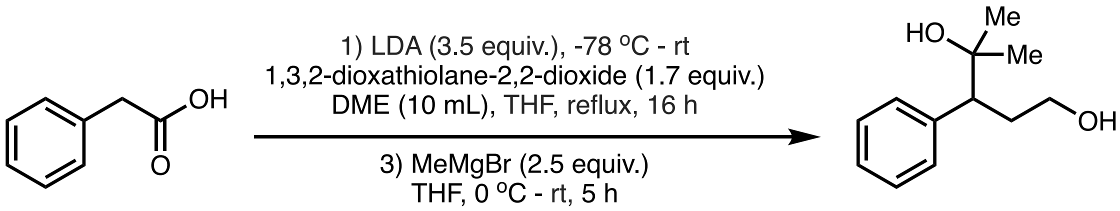

To an *in situ* preprepared solution of Lithium diisopropylamide solution (17.5 mmol) in THF (5 mL) at -78 °C was added a solution of the 2-phenylacetic acid (681 mg, 5 mmol) in THF (3 mL) dropwise. The mixture was stirred for 20 min and warmed to room temperature and left stirring for an additional 45 minutes. A solution of 1,3,2-dioxathiolane-2,2-dioxide (1.05 mg, 8.5 mmol) in THF (5 mL) was added to the mixture followed by DME (10 mL) and the mixture was refluxed for 16 h. after that, the mixture was cooled down to room temperature, quenched with water and extracted with DCM (x 3). The organics were combined, washed with $NaHCO_3$, brine, dried over $MgSO_4$, filtered and concentrated *in vacuo* yielding crude lactone. The crude residue was used for the next step without further purification following general procedure A, step 2 (ii) using methyl magnesium bromide solution (4.2 mL, 12.5 mmol) The crude residue was purified by flash column chromatography (eluent = 30 to 50% EtOAc in hexanes, silica gel) to afford product as white solid (525 mg, 54% yield).

Mp.: 66-70 °C; **R_f** = 0.19 (eluent = 30% EtOAc in hexanes); **ν_{max} / cm^{-1}** (thin film) 3224 (br), 2981, 1377; **^{1}H NMR (500 MHz, $CDCl_3$)** δ 7.32 – 7.29 (m, 2H), 7.26 – 7.22 (m, 3H), 3.59 – 3.54 (m, 1H), 3.43 – 3.38 (m, 1H), 2.79 – 2.76 (dd, *J* = 11.0, 3.9 Hz, 1H), 2.22 – 2.16 (m, 1H), 2.05 – 1.98 (m, 1H), 1.19 (d, *J* = 2.5 Hz, 6H**)**; **^{13}C NMR (126 MHz, $CDCl_3$)** δ 141.1, 129.4, 128.3, 126.8, 72.7, 61.7, 53.7, 32.7, 28.4, 27.4; **HRMS (EI^+) *m/z*** [M - H_2O - CH_3]$^+$ Calcd for $C_{11}H_{13}O$ 161.0960; found 161.0959.

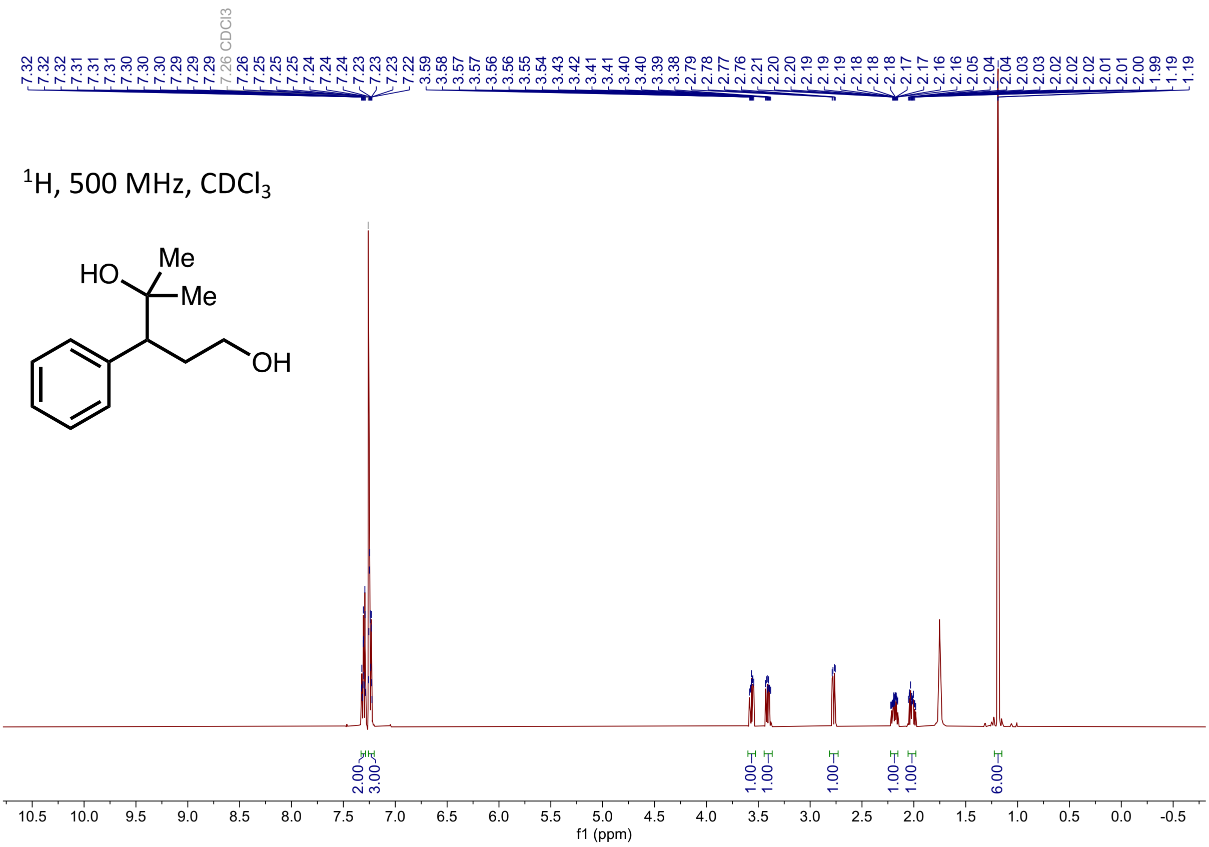
7.32
7.32
7.32
7.31
7.31
7.31
7.30
7.30
7.30
7.29
7.29
7.29
7.26 CDCl3
7.26
7.25
7.25
7.25
7.24
7.24
7.24
7.23
7.23
7.23
7.22
3.59
3.58
3.57
3.56
3.56
3.55
3.54
3.43
3.42
3.41
3.41
3.40
3.40
3.39
3.38
2.79
2.78
2.77
2.76
2.21
2.21
2.20
2.20
2.19
2.19
2.19
2.18
2.18
2.18
2.17
2.17
2.16
2.16
2.05
2.04
2.04
2.03
2.03
2.02
2.02
2.02
2.01
2.01
2.00
1.99
1.19
1.19
1H, 500 MHz, CDCl3
HO
Me
Me
OH
2.00
3.00
1.00
1.00
1.00
1.00
1.00
6.00
10.5
10.0
9.5
9.0
8.5
8.0
7.5
7.0
6.5
6.0
5.5
5.0
4.5
4.0
3.5
3.0
2.5
2.0
1.5
1.0
0.5
0.0
-0.5
f1 (ppm)

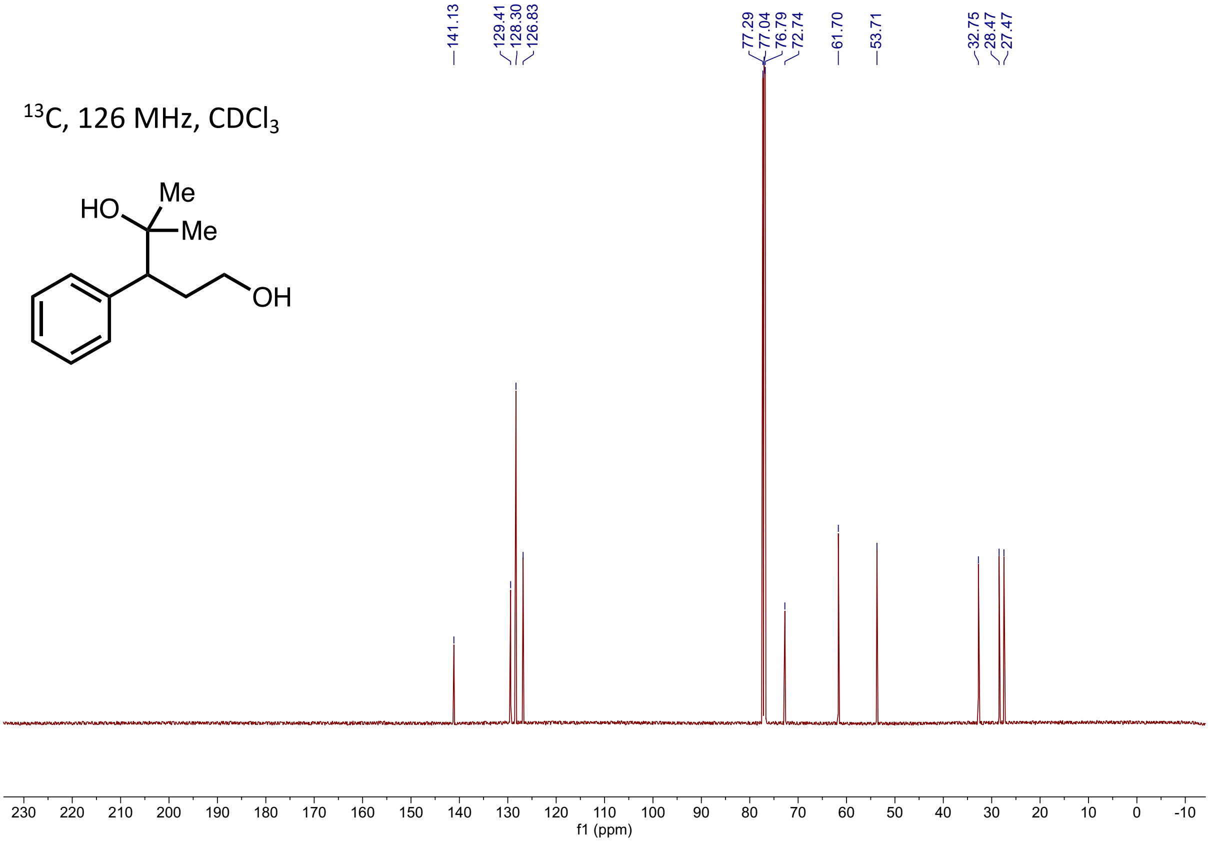
141.13
129.41
128.30
126.83
77.29
77.04
76.79
72.74
61.70
53.71
32.75
28.47
27.47
13C, 126 MHz, CDCl3
HO
Me
Me
OH
230
220
210
200
190
180
170
160
150
140
130
120
110
100
90
80
70
60
50
40
30
20
10
0
-10
f1 (ppm)

(S27)

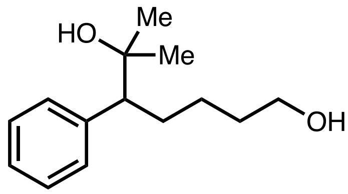

Prepared according to General Procedure B, step 2 using 2-methyl-3-phenylhept-6-en-2-ol (1.02 g, 5 mmol) in THF (10 mL) and BH_3•Me_2S solution (3 mL, 6 mmol, 2.0 M in hexanes). The crude residue was purified by flash column chromatography (eluent = 30 to 50% EtOAc in hexanes, silica gel) to afford product as viscous colourless oil (1.03 g, 93% yield)

R_f = 0.08 (eluent = 30% EtOAc in hexanes); **ν_{max} / cm^{-1}** (thin film) 32548 (br), 2978, 1381; **^1H NMR (500 MHz, $CDCl_3$)** δ 7.33 – 7.27 (m, 2H), 7.25 – 7.19 (m, 3H), 3.56 – 3.51 (m, 2H), 2.60 – 2.55 (dd, *J* = 11.8, 3.4 Hz, 1H), 1.86 – 1.81 (m, 2H), 1.57 – 1.46 (m, 2H), 1.17 – 1.15 (d, *J* = 3.6 Hz, 6H), 1.12 – 1.09 (m, 2H); **^{13}C NMR (126 MHz, $CDCl_3$)** δ 141.2, 129.6, 128.9, 128.7, 128.3, 126.7, 72.9, 62.9, 57.3, 32.9, 29.2, 28.0, 27.9, 24.5.; **HRMS (ES^+) *m/z*** $[M + Na]^+$ Calcd for $C_{14}H_{22}O_2Na$ 245.1517; found 245.1516.

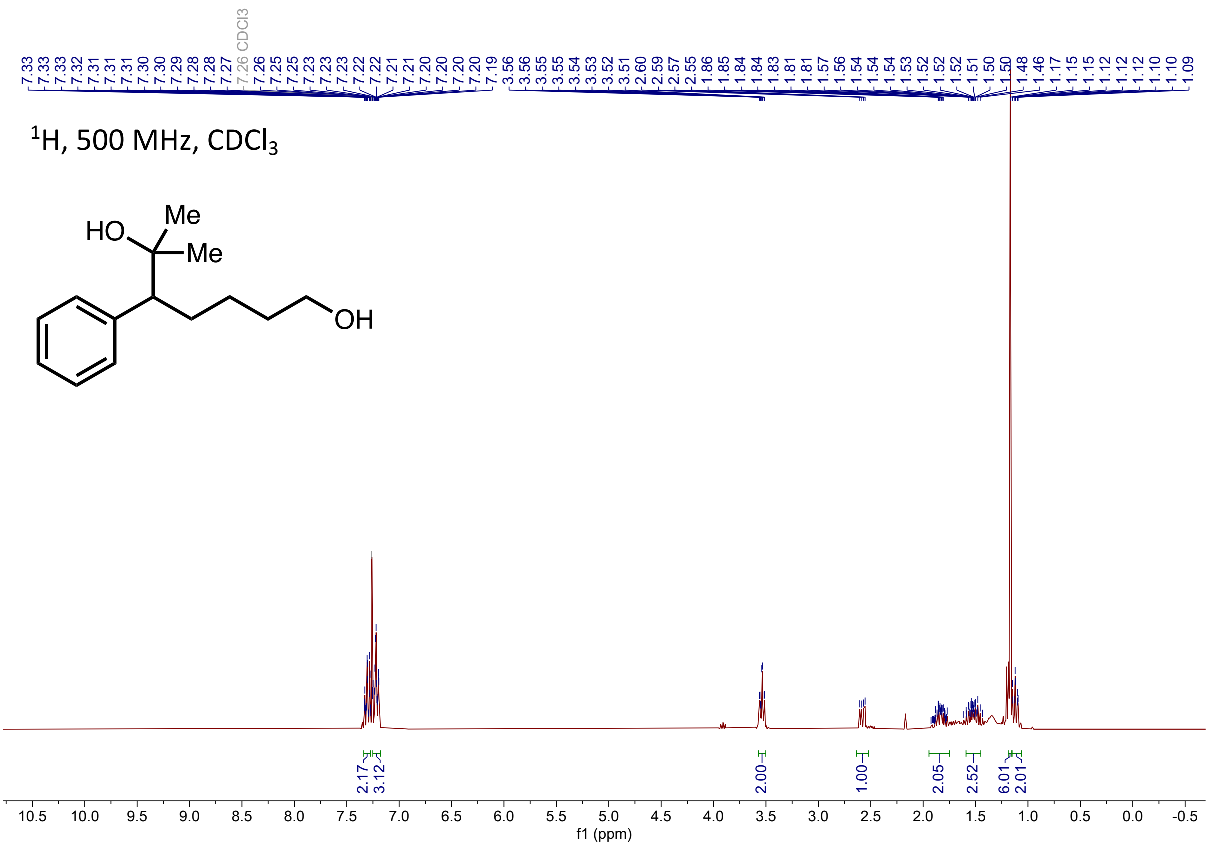
7.33 7.33 7.32 7.31 7.31 7.31 7.30 7.30 7.29 7.28 7.28 7.27 7.26 CDCl3 7.26 7.25 7.25 7.23 7.23 7.23 7.22 7.22 7.21 7.21 7.20 7.20 7.20 7.20 7.19 3.56 3.56 3.55 3.55 3.54 3.53 3.52 3.51 2.60 2.59 2.57 2.55 1.86 1.85 1.84 1.84 1.83 1.81 1.81 1.57 1.56 1.54 1.54 1.54 1.53 1.52 1.52 1.52 1.51 1.50 1.50 1.48 1.46 1.17 1.15 1.15 1.12 1.12 1.12 1.10 1.09
1H, 500 MHz, CDCl3
HO
Me
Me
OH
2.17
3.12
2.00
1.00
2.05
2.52
6.01
2.01
10.5 10.0 9.5 9.0 8.5 8.0 7.5 7.0 6.5 6.0 5.5 5.0 4.5 4.0 3.5 3.0 2.5 2.0 1.5 1.0 0.5 0.0 -0.5
f1 (ppm)

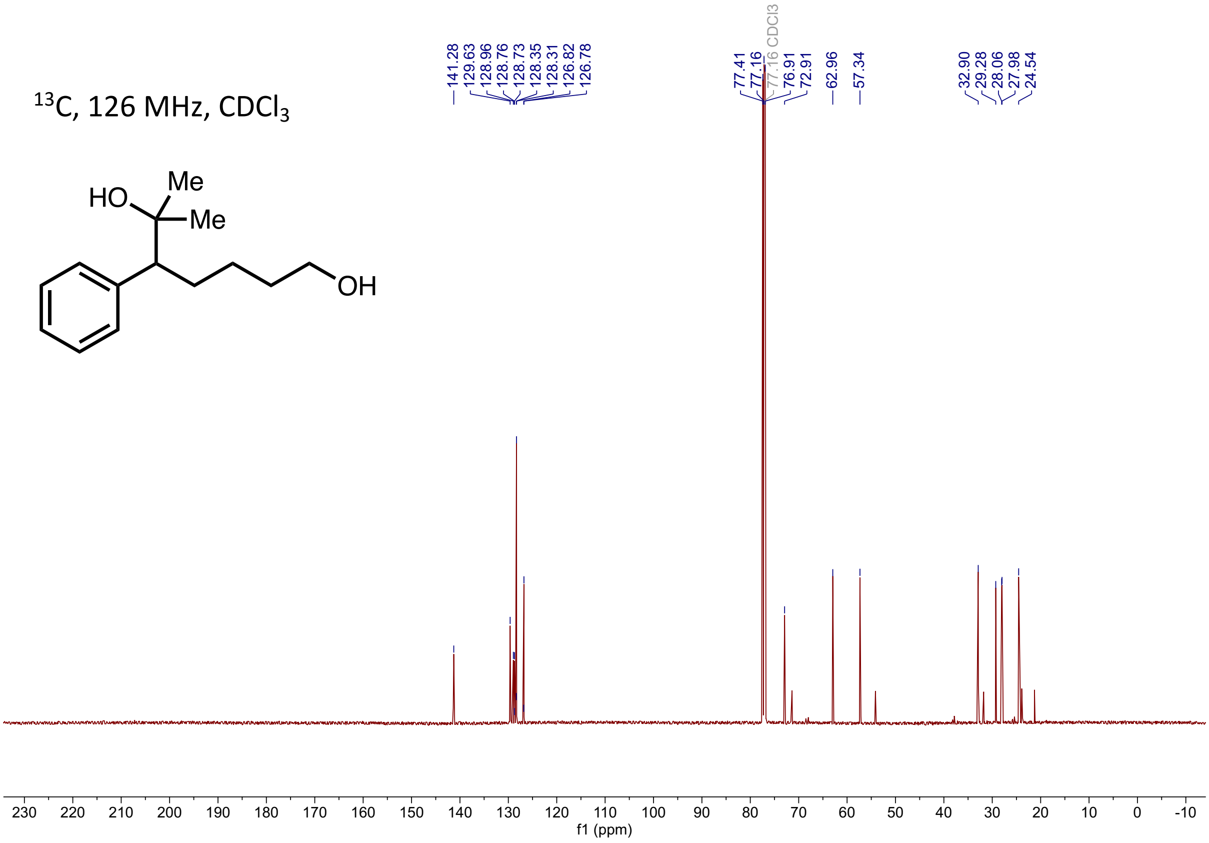
141.28 129.63 128.96 128.76 128.73 128.35 128.31 126.82 126.78
77.41 77.16 77.16 CDCl3 76.91 72.91 62.96 57.34
32.90 29.28 28.06 27.98 24.54
13C, 126 MHz, CDCl3
HO
Me
Me
OH
230 220 210 200 190 180 170 160 150 140 130 120 110 100 90 80 70 60 50 40 30 20 10 0 -10
f1 (ppm)

(S28)

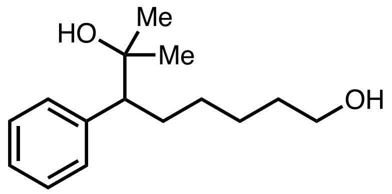

Prepared according to General Procedure B, step 2 using 2-methyl-3-phenyloct-7-en-2-ol (2.18 g, 10 mmol), THF (20 mL) and BH_3•Me_2S solution (6 mL, 12 mmol). The crude residue was purified by flash column chromatography (eluent = 30 to 50% EtOAc in hexanes, silica gel) to afford product as viscous colourless oil (1.39 g, 59% yield).

R_f = 0.21 (eluent = 20% EtOAc in hexanes); **ν_{max} / cm^{-1}** (thin film) 3242 (br), 2985, 2887, 1381; **^{1}H NMR (500 MHz, $CDCl_3$)** δ 7.32 – 7.29 (m, 2H), 7.25 – 7.20 (m, 3H), 3.57 – 3.54 (t, *J* = 6.6 Hz, 2H), 2.59 – 2.56 (dd, *J* = 11.6, 3.6 Hz, 1H), 1.83 – 1.80 (m, 2H), 1.50 – 1.45 (m, 2H), 1.34 – 1.26 (m, 4H), 1.17 (d, *J* = 2.5 Hz, 6H), 1.12 – 1.07 (m, 2H); **^{13}C NMR (126 MHz, $CDCl_3$)** δ 141.3, 129.6, 128.2, 126.7, 72.9, 63.0, 57.3, 32.6, 29.4, 28.1, 28.0, 27.9, 25.8; **HRMS (CI$^+$) *m/z*** [M - CH_3]$^+$ Calcd for $C_{14}H_{21}O_2$ 221.1536; found 221.1536.

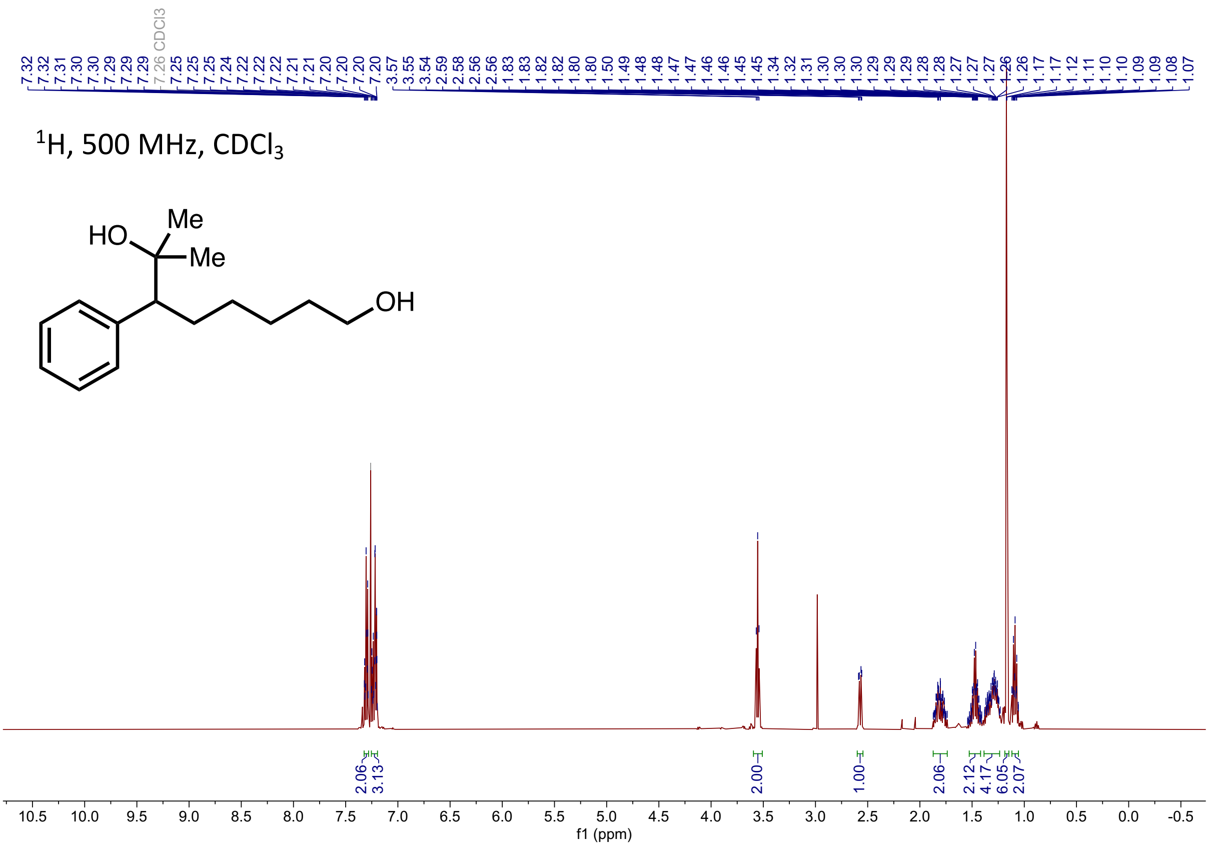

^{1}H, 500 MHz, CDCl$_3$
HO
Me
Me
OH
f1 (ppm)

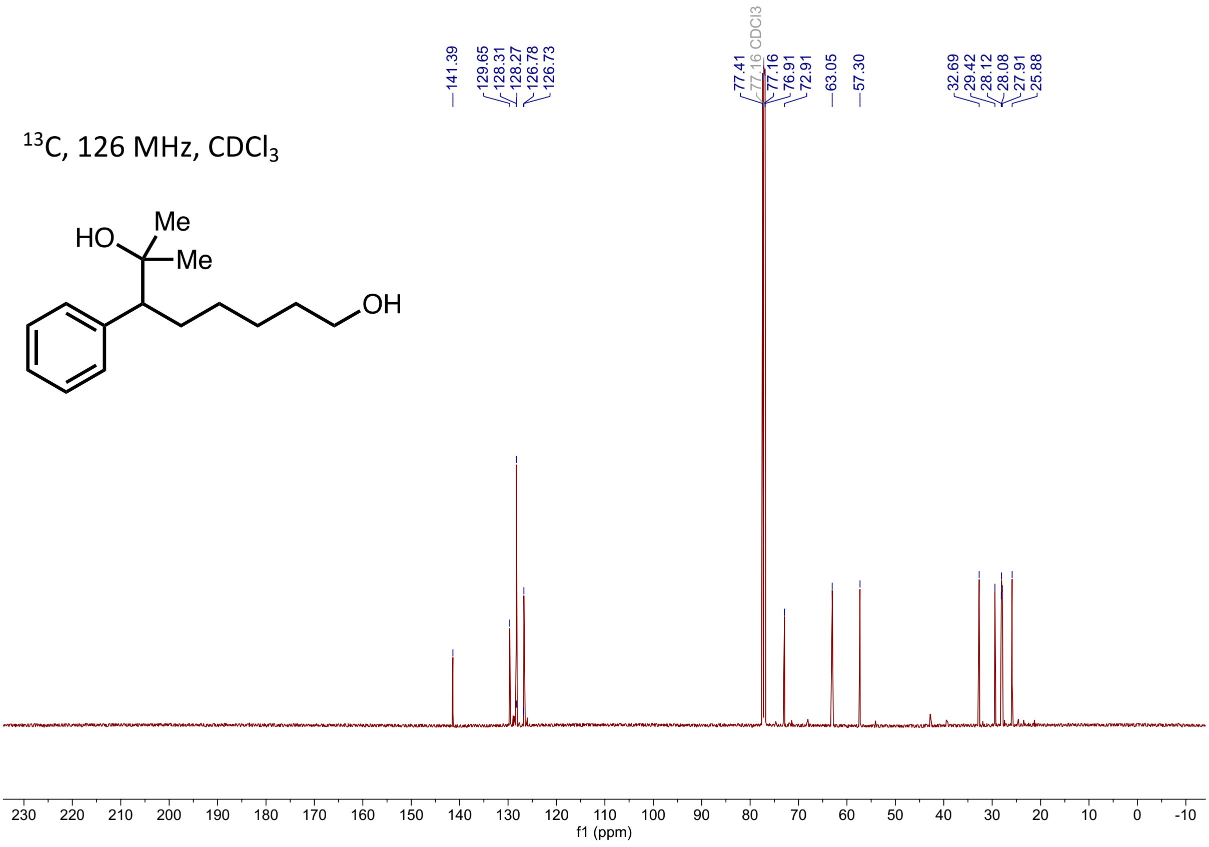

^{13}C, 126 MHz, CDCl$_3$
HO
Me
Me
OH
f1 (ppm)

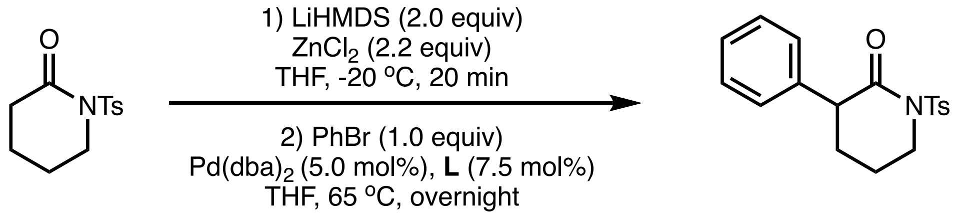

Crude 3-phenyl-1-tosylpiperidin-2-one was prepared following literature method[4] using 1-tosylpiperidin-2-one. To a stirred solution of 1-tosylpiperidin-2-one (2.79 g, 11 mmol) in THF (20 mL) at -20 °C was added a solution of LiHMDS (10 mL). After 20 min at -20 °C, a solution of $ZnCl_2$ (1.5 g, 11 mmol) in THF (10 mL) was added and left to stir for an additional 20 min. After that, the solution was cannulated into a stirred solution of 2-dicyclohexylphosphino-2'-(*N*,*N*-dimethylamino)-biphenyl **L** (148 mg, 0.375 mmol), $Pd(dba)_2$ (144 mg, 0.25 mmol) and bromobenzene (0.53 mL, 5 mmol) in THF (10 mL). The reaction mixture was warmed up to 65 °C and left to stir overnight. The mixture was cooled down, quenched with saturated NH_4Cl solution and extracted with Et_2O (x 2). The organics were combined, dried over $MgSO_4$, filtered and concentrated *in vacuo* yielding crude 3-phenyl-1-tosylpiperidin-2-one. The crude residue was used for the next step without further purification.

(S29)

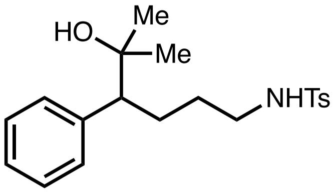

Prepared according to general procedure A, step 2 (ii) using crude 3-phenyl-1-tosylpiperidin-2-one, THF (10 mL) and methyl magnesium bromide solution (4.2 mL, 12.5 mmol). The crude residue was purified by flash column chromatography (eluent = 40 to 50% EtOAc in hexanes, silica gel) to afford product as brown solid (650 mg, 36% yield).

Mp.: 46-49 °C; **R_f** = 0.17 (eluent = 40% EtOAc in hexanes); **ν_{max} / cm^{-1}** (thin film) 3446, 2985, 1386; **^{1}H NMR (500 MHz, $CDCl_3$)** δ 7.70 – 7.67 (m, 2H), 7.30 – 7.21 (m, 5H), 7.15 – 7.13 (m, 2H), 4.31 – 4.30 (m, 1H), 2.89 – 2.85 (q, *J* = 6.7 Hz, 3H), 2.50 – 2.47 (dd, *J* = 12.1, 3.2 Hz, 1H), 2.42 (s, 3H), 1.89 – 1.82 (m, 1H), 1.76 – 1.68 (m, 1H), 1.26 – 1.19 (m, 2H), 1.13 – 1.12 (d, *J* = 4.7 Hz, 6H); **^{13}C NMR (126 MHz, $CDCl_3$)** δ 143.4, 140.8, 137.0, 129.8, 129.4, 128.4, 127.2, 126.9, 72.8, 56.7, 43.2, 28.2, 27.7, 26.3, 21.6; **HRMS (ESN$^+$)** ***m/z*** [M - H]$^+$ Calcd for $C_{20}H_{26}NO_3S$ 360.1633; found 360.1618.

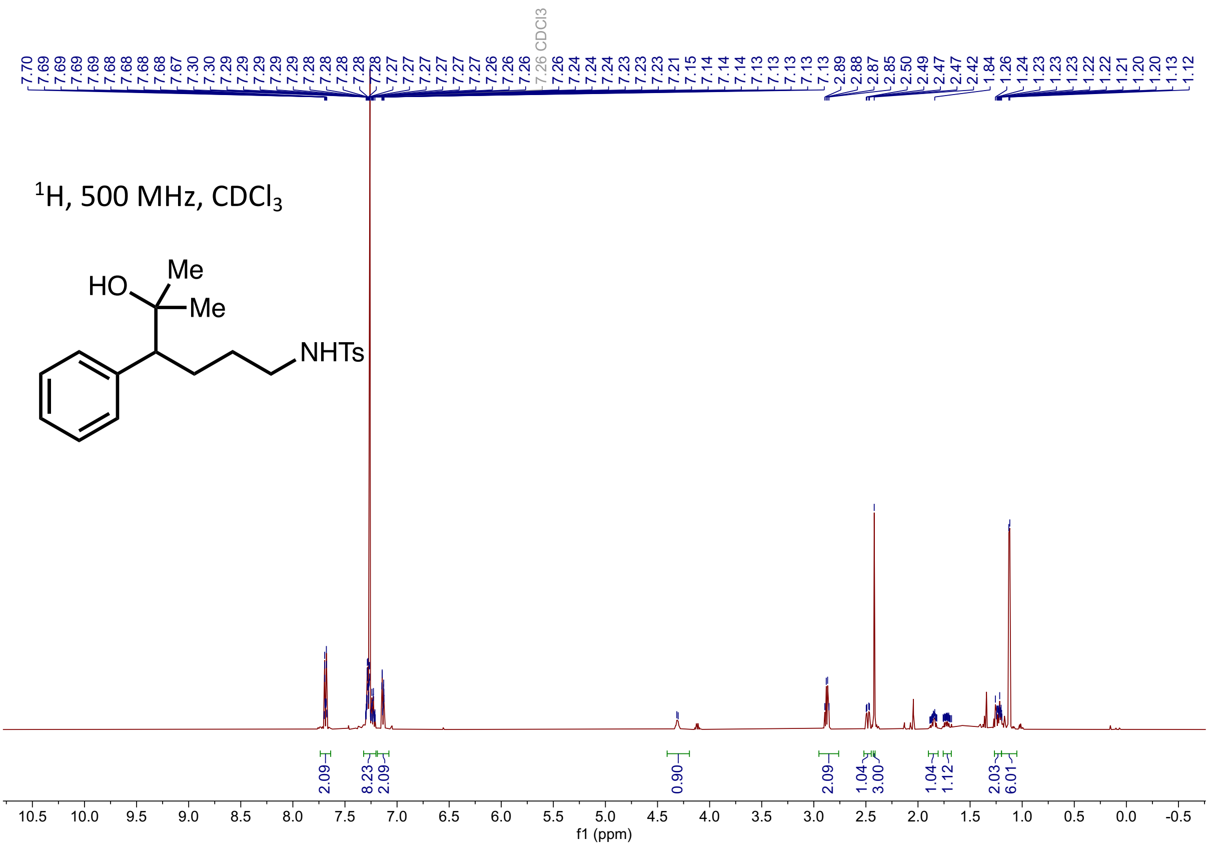

7.70 7.69 7.69 7.69 7.69 7.68 7.68 7.68 7.68 7.67 7.30 7.29 7.29 7.29 7.29 7.29 7.28 7.28 7.28 7.28 7.28 7.27 7.27 7.27 7.27 7.27 7.26 7.26 7.26 CDCl3 7.26 7.24 7.24 7.24 7.23 7.23 7.23 7.23 7.21 7.15 7.14 7.14 7.14 7.13 7.13 7.13 7.13 7.13 2.89 2.88 2.87 2.85 2.50 2.49 2.47 2.47 2.42 1.84 1.26 1.24 1.23 1.23 1.23 1.22 1.22 1.21 1.20 1.20 1.13 1.12
1H, 500 MHz, CDCl3
HO
Me
Me
NHTs
2.09 8.23 2.09 0.90 2.09 1.04 3.00 1.04 1.12 2.03 6.01
10.5 10.0 9.5 9.0 8.5 8.0 7.5 7.0 6.5 6.0 5.5 5.0 4.5 4.0 3.5 3.0 2.5 2.0 1.5 1.0 0.5 0.0 -0.5
f1 (ppm)

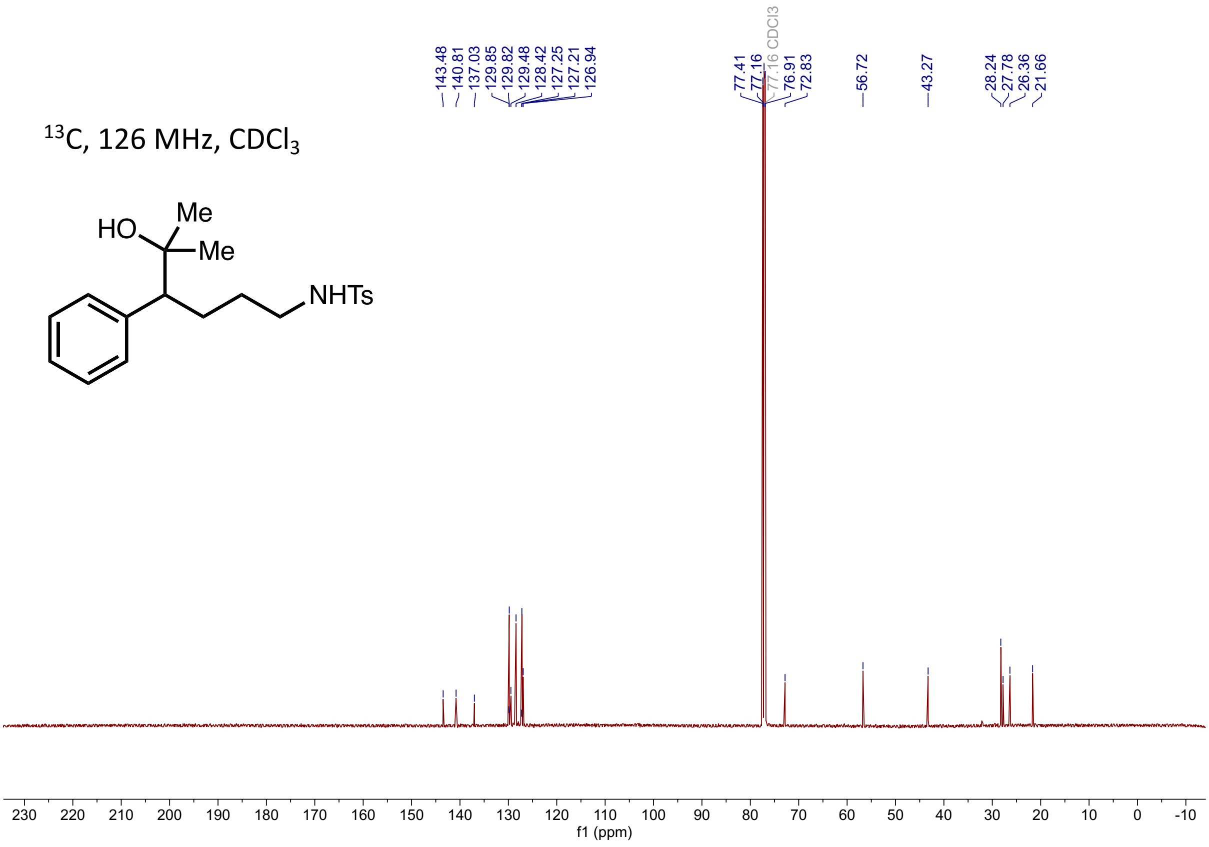

143.48 140.81 137.03 129.85 129.82 129.48 128.42 127.25 127.21 126.94
77.41 77.16 77.16 CDCl3 76.91 72.83 56.72 43.27 28.24 27.78 26.36 21.66
13C, 126 MHz, CDCl3
HO
Me
Me
NHTs
230 220 210 200 190 180 170 160 150 140 130 120 110 100 90 80 70 60 50 40 30 20 10 0 -10
f1 (ppm)

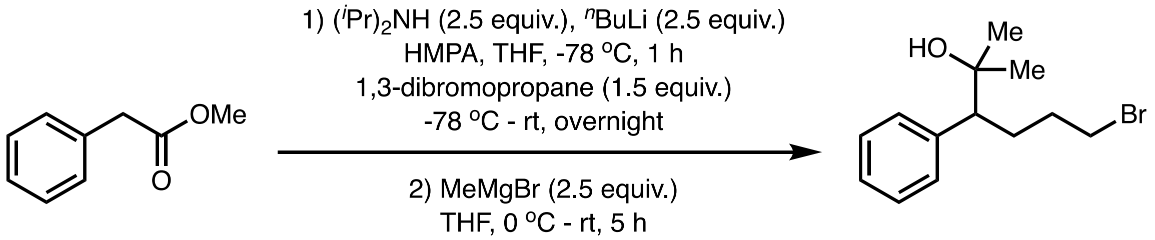

Crude 6-bromo-2-methyl-3-phenylhexan-2-ol was made following modified general procedure B, step 1 using Lithium diisopropylamide solution (12 mmol), 2-phenylacetate (1.5 g, 10 mmol) and 1,3-dibromopropane (3.23 g, 16 mmol). To the resulted crude, in THF (20 mL), was added methyl magnesium bromide solution (8.4 mL, 25 mmol) following general procedure A, step 2 (ii). The resulted crude residue was used for the next step without further purification.

(S30)

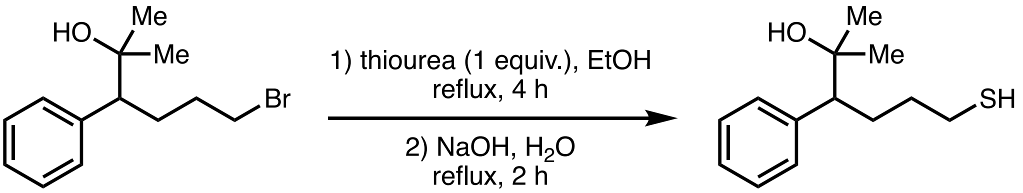

To a solution of thiourea (762 mg, 10 mmol) in EtOH (20 mL) was added crude 6-bromo-2-methyl-3-phenylhexan-2-ol and the mixture was heated to reflux for 4 h. The mixture was cooled down to room temperature and concentrated *in vacuo* to remove EtOH. A solution of NaOH (560 mg) in water (10 mL) was added to the resulted residue and the mixture was heated to reflux for 2 h. The mixture was cooled down to room temperature, acidified with saturated NH_4Cl solution and extracted with Et_2O (x 3). The organics were washed with H_2O, combined, dried over $MgSO_4$, filtered and concentrated *in vacuo* yielding crude residue. The crude residues were purified by flash column chromatography (eluent = 40 to 50% EtOAc in hexanes, silica gel) to afford the product as colourless oil (942 mg, 42% yield).

R_f = 0.11 (eluent = 30% EtOAc in hexanes); **^{1}H NMR (500 MHz, $CDCl_3$)** δ 7.33 – 7.29 (m, 2H), 7.25 – 7.20 (m, 3H), 2.58 – 2.55 (dd, *J* = 12.1, 3.2 Hz, 1H), 2.50 – 2.42 (m, 2H), 1.99 – 1.95 (m, 1H), 1.87 – 1.85 (m, 1H), 1.43 – 1.37 (m, 2H), 1.26 (t, *J* = 7.8 Hz, 1H), 1.18 (d, *J* = 3.0 Hz, 5H); **^{13}C NMR (126 MHz, $CDCl_3$)** δ 140.9, 129.5, 128.3, 126.9, 72.8, 56.8, 32.6, 28.1, 28.0, 24.8.

Data consistent with the literature.[5]

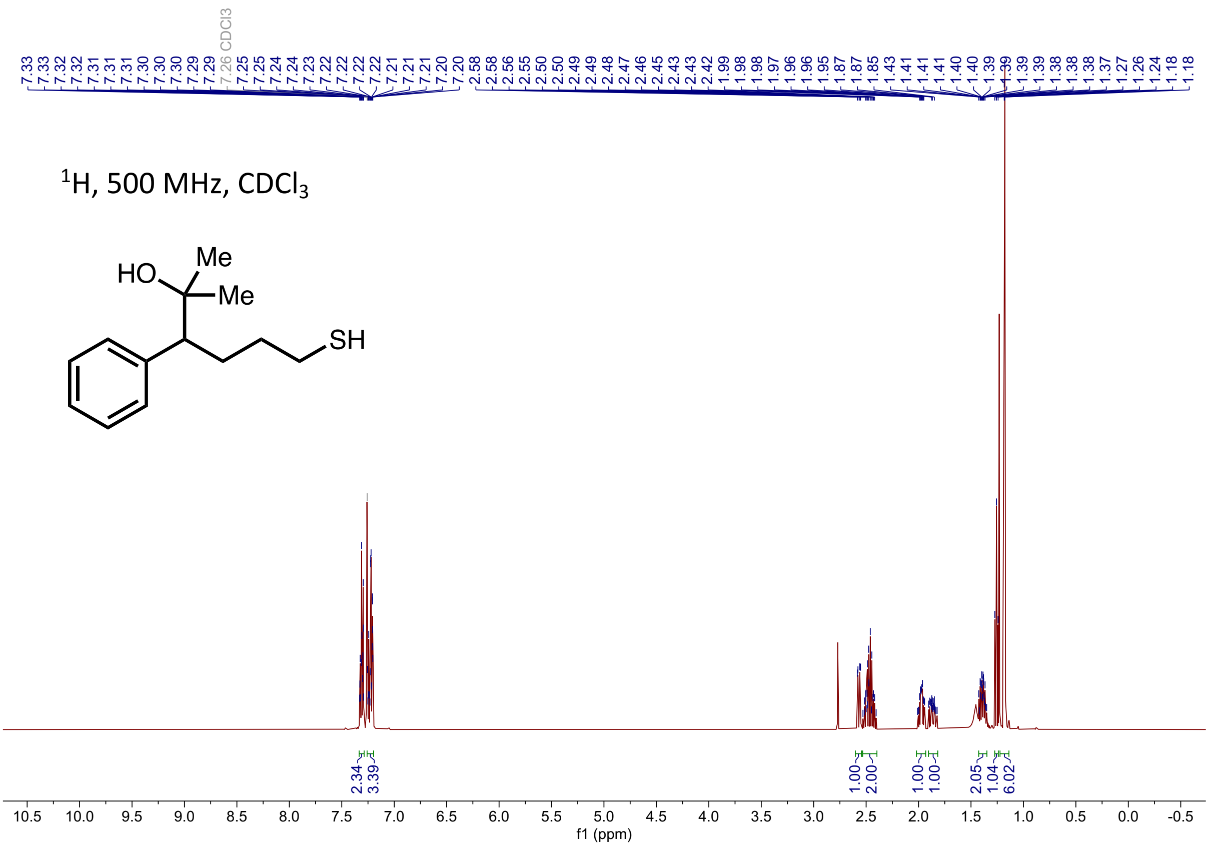

^1H, 500 MHz, $CDCl_3$
HO
Me
Me
SH
2.34
3.39
1.00
2.00
1.00
1.00
2.05
1.04
6.02
f1 (ppm)

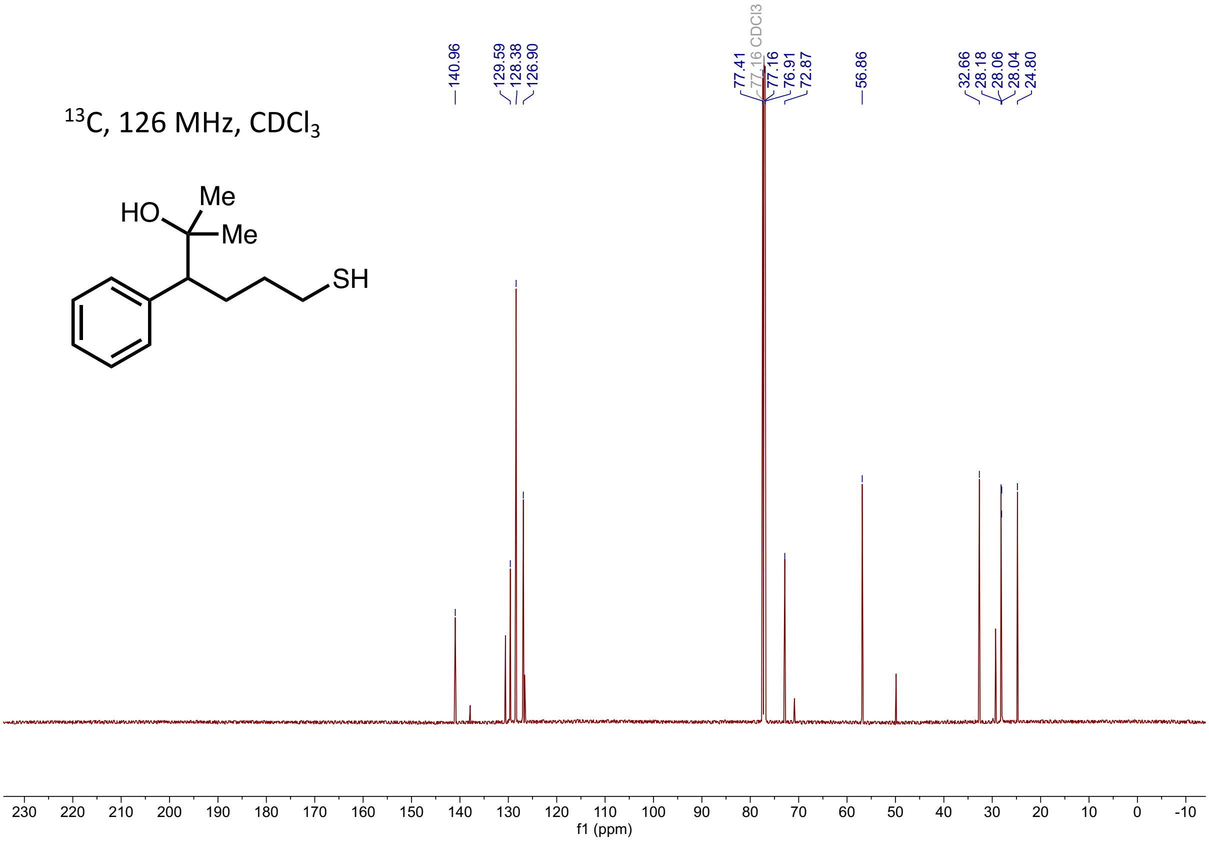

^{13}C, 126 MHz, $CDCl_3$
140.96
129.59
128.38
126.90
77.41
77.16 CDCl3
77.16
76.91
72.87
56.86
32.66
28.18
28.06
28.04
24.80
HO
Me
Me
SH
f1 (ppm)

(S31)

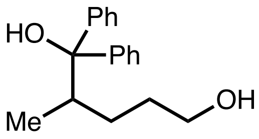

Prepared according to general procedure C using 3-methyltetrahydro-2*H*-pyran-2-one (570 mg, 5 mmol), THF (10 mL) and phenylmagnesium bromide solution (4.2 mL, 12.5 mmol). The crude residue was purified by flash column chromatography (eluent = 30 to 50% EtOAc in hexanes, silica gel) to afford product as colourless oil (1.23 g, 91% yield).

R_f = 0.28 (eluent = 30% EtOAc in hexanes); **ν_{max} / cm^{-1}** (thin film) 3280 (br), 2981, 2360, 1384;**^{1}H NMR (500 MHz, $CDCl_3$)** δ 7.50 – 7.47 (m, 4H), 7.31 – 7.26 (m, 4H), 7.19 – 7.15 (m, 2H), 3.59 – 3.55 (m, 2H), 2.66 (m, 1H), 1.74 – 1.70 (m, 1H), 1.54 – 1.49 (m, 2H), 1.16 – 1.10 (m, 1H), 0.92 – 0.90 (d, *J* = 6.7 Hz, 3H); **^{13}C NMR (126 MHz, $CDCl_3$)** δ 146.68, 128.37, 128.26, 126.63, 126.52, 125.79, 81.25, 63.17, 40.36, 31.23, 27.57, 14.34; **HRMS (EI$^+$)** ***m/z*** [M - H_2O]$^+$ Calcd for $C_{18}H_{20}O$ 252.1508; found 252.1509.

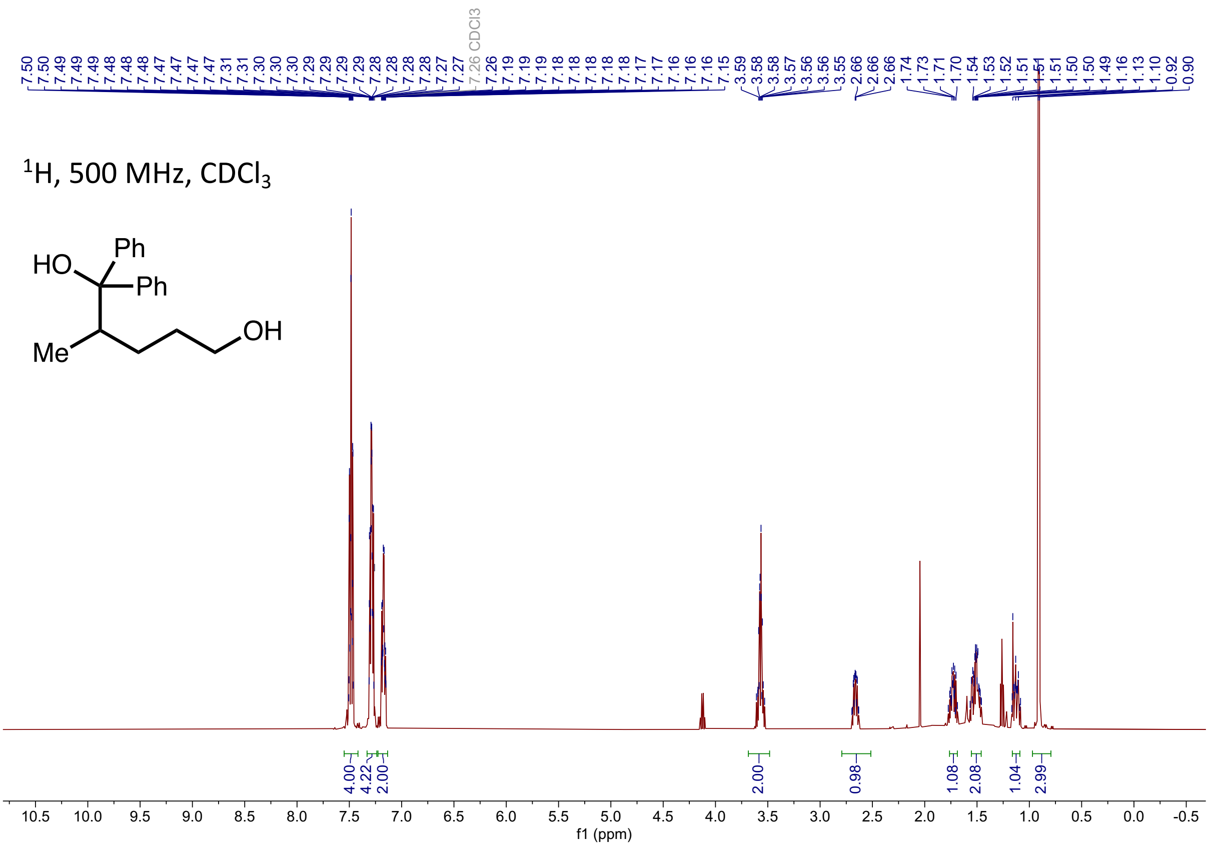
^{1}H, 500 MHz, $CDCl_3$
HO
Ph
Ph
Me
OH
f1 (ppm)

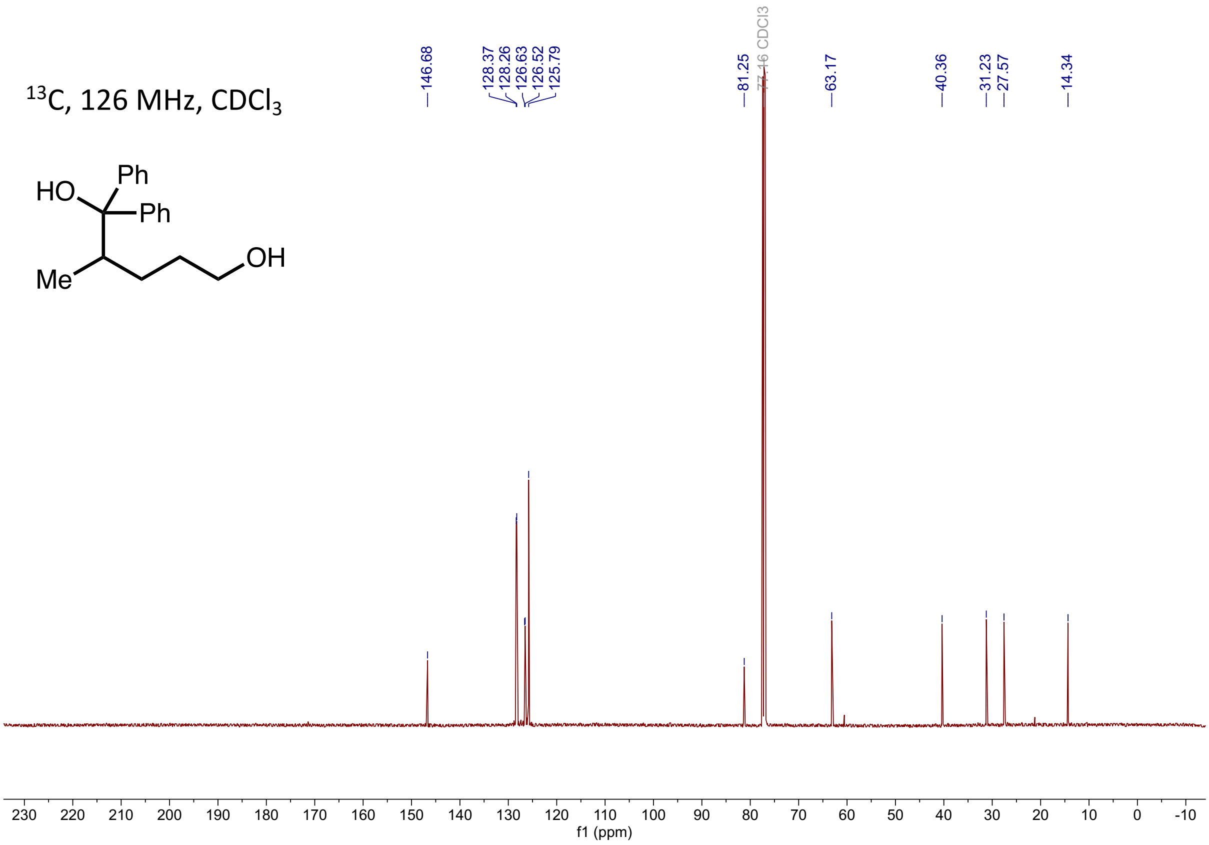
^{13}C, 126 MHz, $CDCl_3$
146.68
128.37
128.26
126.63
126.52
125.79
81.25
77.16 CDCl3
63.17
40.36
31.23
27.57
14.34
HO
Ph
Ph
Me
OH
f1 (ppm)

(S32)

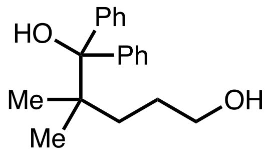

Prepared according to general procedure C using 3,3-dimethyltetrahydro-2*H*-pyran-2-one (641 mg, 5 mmol), THF (10 mL) and phenylmagnesium bromide solution (4.2 mL, 12.5 mmol). The crude residue was purified by flash column chromatography (eluent = 30 to 50% EtOAc in hexanes, silica gel) to afford product as colourless oil (1.33 g, 94% yield).

R_f = 0.25 (eluent = 30% EtOAc in hexanes); **ν_{max} / cm^{-1}** (thin film) 3269 (br), 2981, 2361, 1384; **^{1}H NMR (500 MHz, $CDCl_3$)** δ 7.52 – 7.50 (m, 4H), 7.29 – 7.25 (m, 4H), 7.22 – 7.19 (m, 2H), 3.59 – 3.56 (m, 2H), 1.60 – 1.59 (m, 4H), 1.15 (s, 6H); **^{13}C NMR (126 MHz, $CDCl_3$)** δ 146.1, 128.8, 127.4, 126.6, 83.7, 63.9, 41.6, 33.9, 28.1, 23.8; **HRMS (EI^+)** ***m/z*** $[M - H_2O - CH_3]^+$ Calcd for $C_{18}H_{19}O$ 251.1430; found 251.1431.

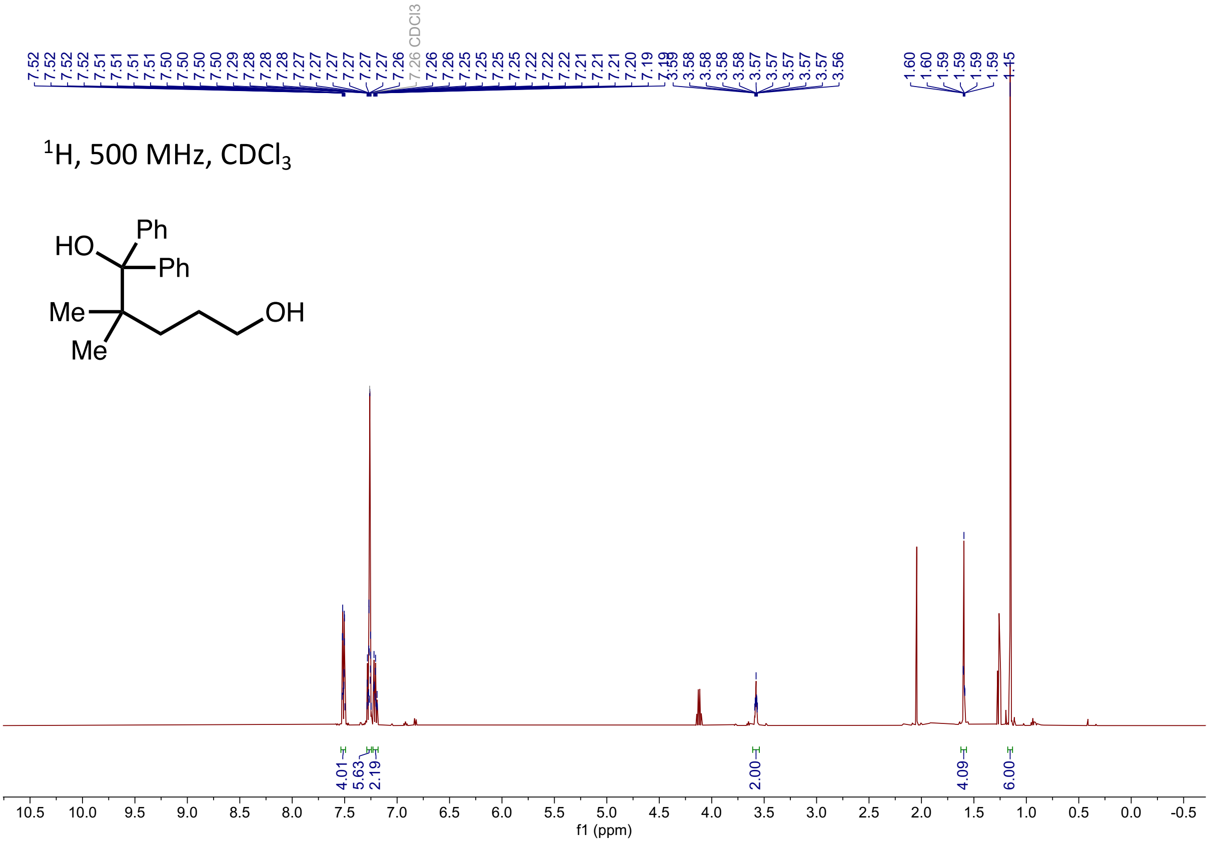

7.52 7.52 7.52 7.52 7.51 7.51 7.51 7.50 7.50 7.50 7.50 7.29 7.28 7.28 7.28 7.27 7.27 7.27 7.27 7.27 7.26 7.26 CDCl3 7.26 7.26 7.25 7.25 7.25 7.25 7.22 7.22 7.22 7.21 7.21 7.21 7.20 7.19 7.19 3.59 3.58 3.58 3.58 3.58 3.57 3.57 3.57 3.57 3.56
1.60 1.60 1.59 1.59 1.59 1.59 1.15
^{1}H, 500 MHz, CDCl$_3$
HO
Ph
Ph
Me
Me
OH
4.01
5.63
2.19
2.00
4.09
6.00
f1 (ppm)

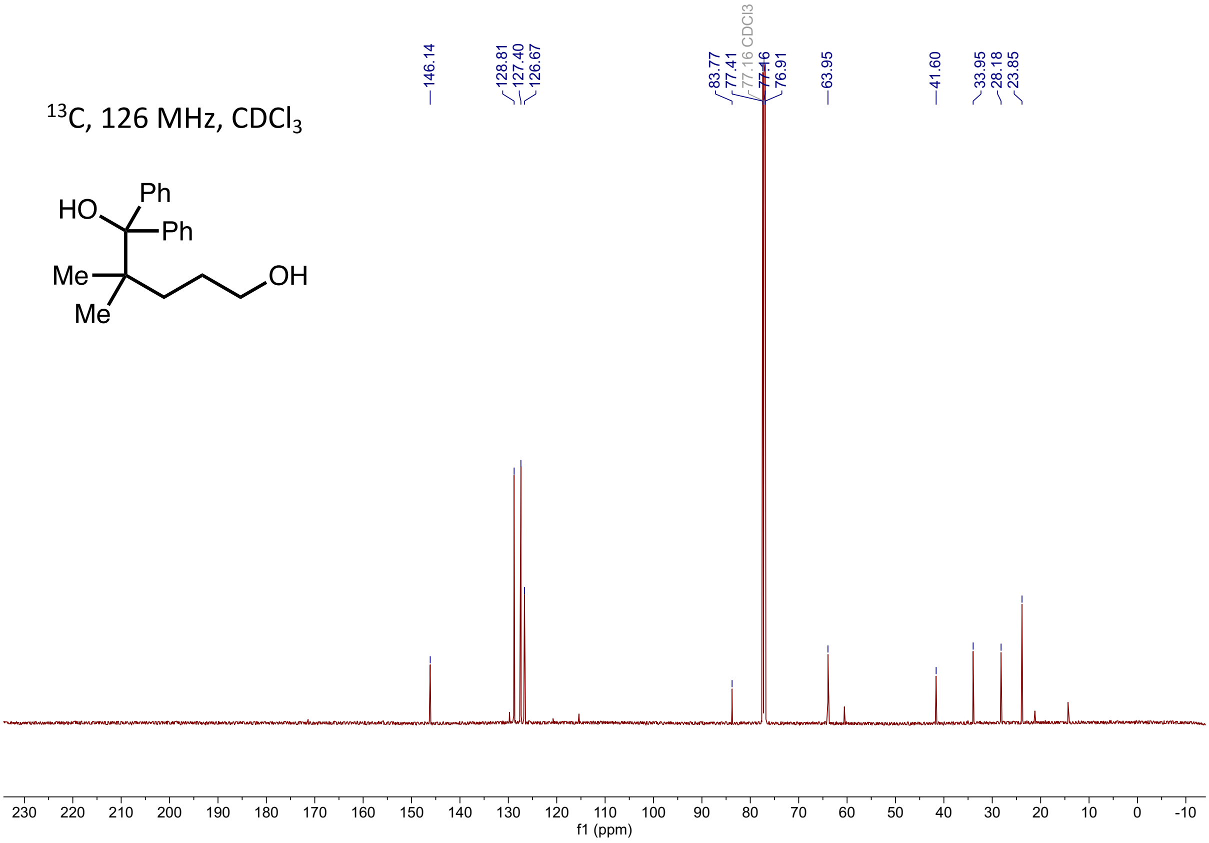

146.14
128.81 127.40 126.67
83.77 77.41 77.16 CDCl3 77.16 76.91
63.95
41.60
33.95 28.18 23.85
^{13}C, 126 MHz, CDCl$_3$
HO
Ph
Ph
Me
Me
OH
f1 (ppm)

(S33)

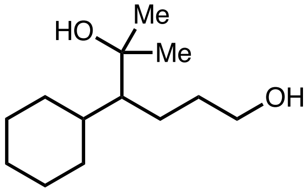

Prepared according to modified general procedure B, step 2 using 3-cyclohexyl-2-methylhex-5-en-2-ol (982 g, 5 mmol) in THF (10 mL) and BH_3•Me_2S solution (3 mL, 6 mmol, 2.0 M in hexanes). The crude residue was purified by flash column chromatography (eluent = 30 to 50% EtOAc in hexanes, silica gel) to afford product as pale-yellow oil (686 mg, 64% yield).

R_f = 0.26 (eluent = 30% EtOAc in hexanes); **ν_{max} / cm^{-1}** (thin film) 3365 (br), 2985, 2358, 1388; **^{1}H NMR (500 MHz, $CDCl_3$)** δ 3.67 – 3.64 (m, 2H), 1.75 – 1.69 (m, 3H), 1.68 – 1.62 (m, 2H), 1.61 – 1.59 (m, 1H), 1.58 – 1.55 (m, 3H), 1.54 – 1.50 (m, 2H), 1.44 – 1.35 (m, 1H), 1.29 – 1.25 (m, 2H), 1.23 (s, 3H), 1.20 (s, 3H), 1.16 – 1.13 (m, 2H); **^{13}C NMR (126 MHz, $CDCl_3$)** δ 74.8, 63.1, 54.5, 39.1, 35.1, 34.3, 29.2, 29.0, 27.8, 27.7, 27.0, 26.6, 22.1; **HRMS (ESP^+) *m/z*** $[M + Li]^+$ Calcd for $C_{13}H_{26}O_2Li$ 221.2093; found 221.2091.

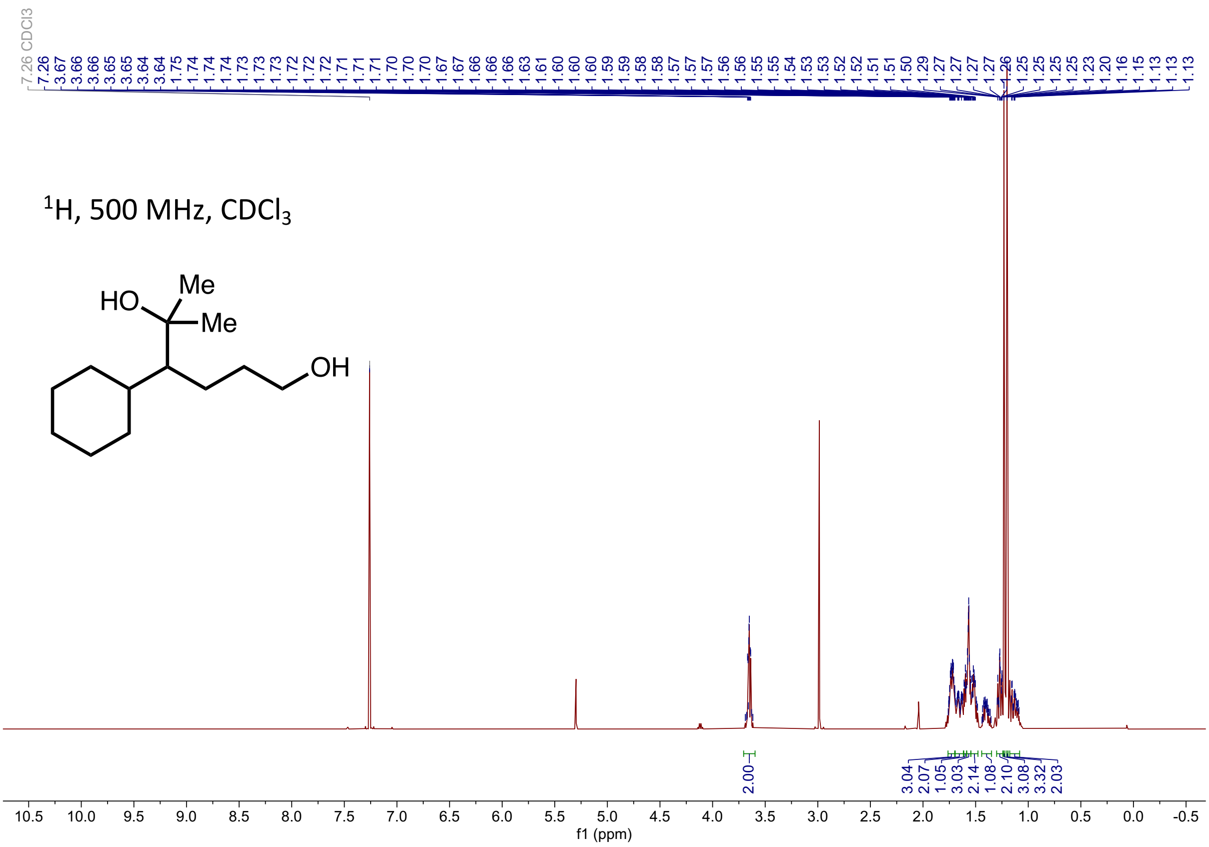
^{1}H, 500 MHz, CDCl$_3$
HO
Me
Me
OH
7.26 CDCl3
f1 (ppm)

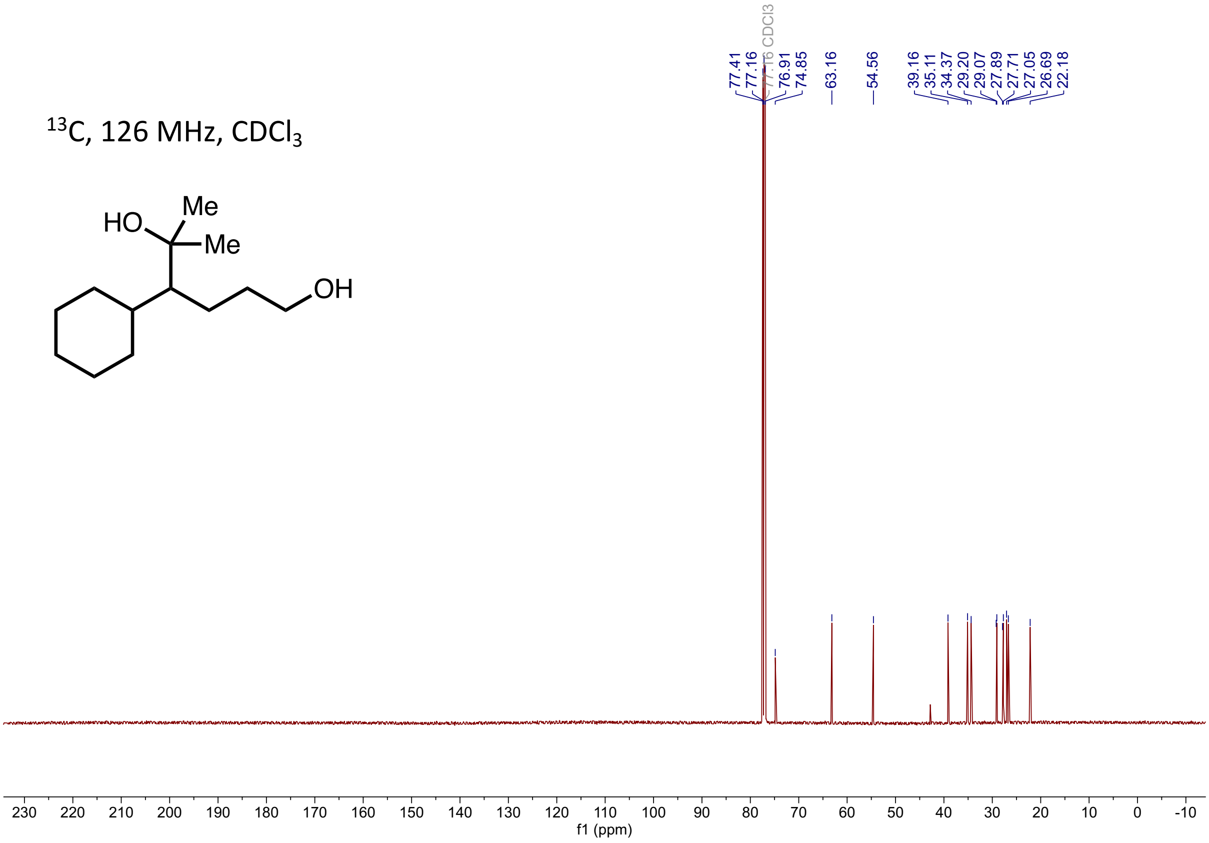
^{13}C, 126 MHz, CDCl$_3$
HO
Me
Me
OH
77.41
77.16
77.16 CDCl3
76.91
74.85
63.16
54.56
39.16
35.11
34.37
29.20
29.07
27.89
27.71
27.05
26.69
22.18
f1 (ppm)

(S34)

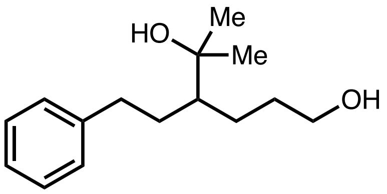

Prepared according to modified general procedure B, step 2 using 2-methyl-3-phenethylhex-5-en-2-ol (1.09 g, 5 mmol) in THF (10 mL) and $BH_3 \bullet Me_2S$ solution (3 mL, 6 mmol, 2.0 M in hexanes). The crude residue was purified by flash column chromatography (eluent = 30 to 50% EtOAc in hexanes, silica gel) to afford product as yellow oil (445 mg, 38% yield).

R_f = 0.19 (eluent = 30% EtOAc in hexanes); **ν_{max} / cm^{-1}** (thin film) 3377 (br), 2985, 2361, 1386; **^{1}H NMR (500 MHz, $CDCl_3$)** δ 7.30 – 7.27 (m, 2H), 7.21 – 7.17 (m, 3H), 3.70 – 3.63 (m, 2H), 2.78 – 2.72 (m, 1H), 2.64 – 2.58 (m, 1H), 1.79 – 1.68 (m, 3H), 1.61 – 1.57 (m, 1H), 1.48 – 1.41 (m, 1H), 1.39 – 1.35 (m, 1H), 1.33 – 1.25 (m, 1H), 1.22 (s, 3H), 1.18 (s, 3H); **^{13}C NMR (126 MHz, $CDCl_3$)** δ 142.7, 128.5, 128.5, 125.9, 74.3, 62.9, 48.6, 35.6, 34.0, 32.4, 28.2, 26.9, 26.6; **HRMS (EI$^+$)** ***m/z*** [M - H_2O - CH_3]$^+$ Calcd for $C_{14}H_{19}O$ 203.1430; found 203.1431.

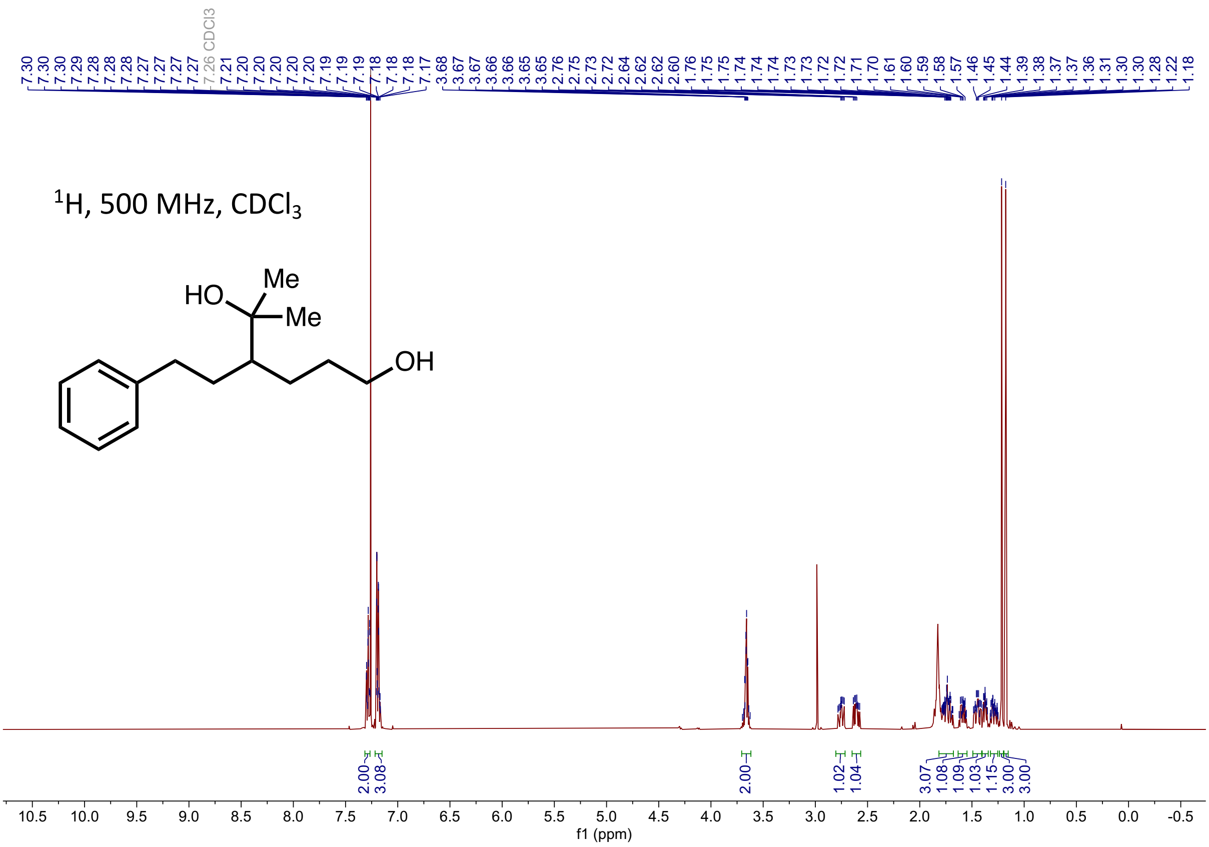
1H, 500 MHz, CDCl3
HO
Me
Me
OH
7.30 7.30 7.30 7.29 7.28 7.28 7.28 7.27 7.27 7.27 7.27 7.26 CDCl3 7.21 7.20 7.20 7.20 7.20 7.20 7.19 7.19 7.19 7.18 7.18 7.17 3.68 3.67 3.66 3.66 3.65 3.65 2.76 2.75 2.73 2.72 2.64 2.62 2.62 2.60 1.76 1.75 1.75 1.74 1.74 1.74 1.73 1.73 1.72 1.72 1.71 1.70 1.61 1.60 1.59 1.58 1.57 1.46 1.45 1.44 1.39 1.38 1.37 1.37 1.36 1.31 1.30 1.30 1.28 1.22 1.18
2.00 3.08 2.00 1.02 1.04 3.07 1.08 1.09 1.03 1.15 3.00 3.00
f1 (ppm)

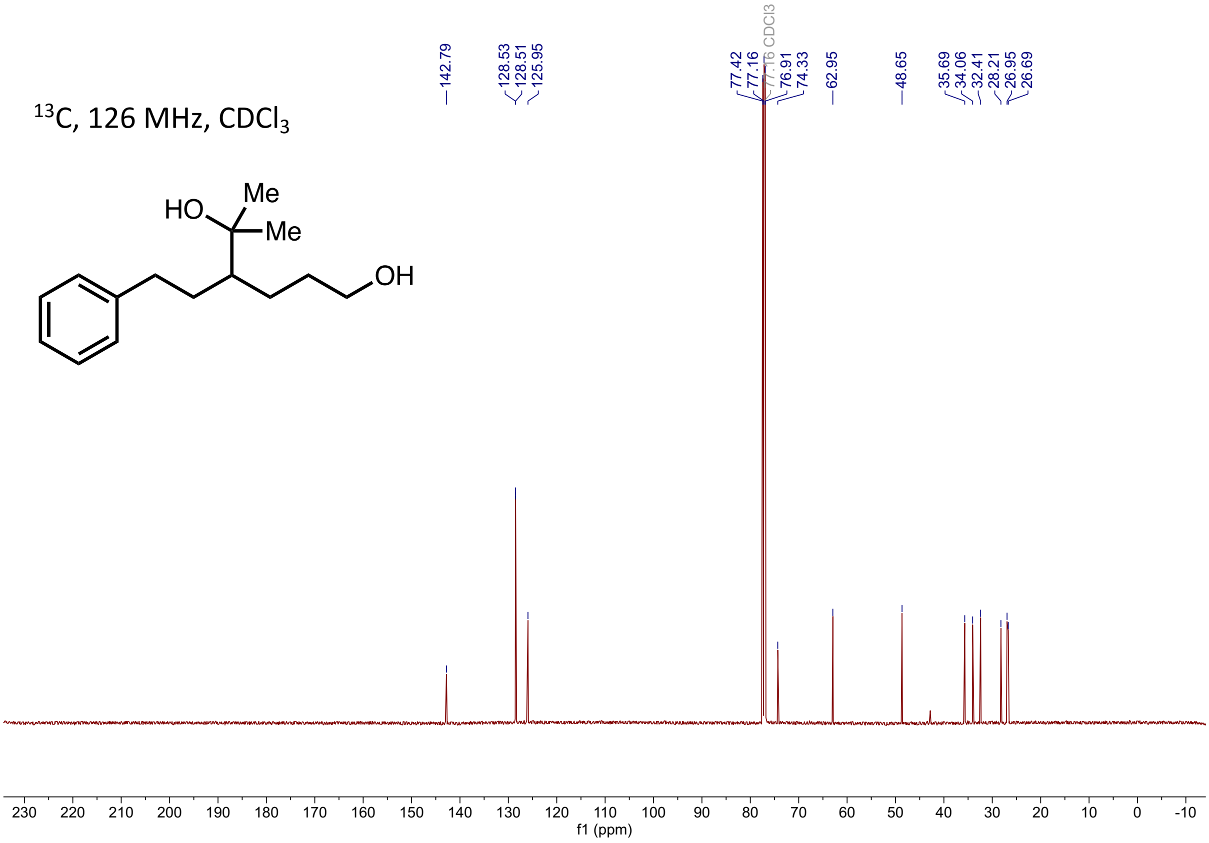
13C, 126 MHz, CDCl3
HO
Me
Me
OH
142.79 128.53 128.51 125.95 77.42 77.16 77.16 CDCl3 76.91 74.33 62.95 48.65 35.69 34.06 32.41 28.21 26.95 26.69
f1 (ppm)

(S37) and (S39) Secured commercially.

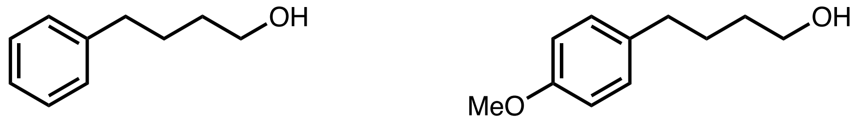

(S38) and (S40)

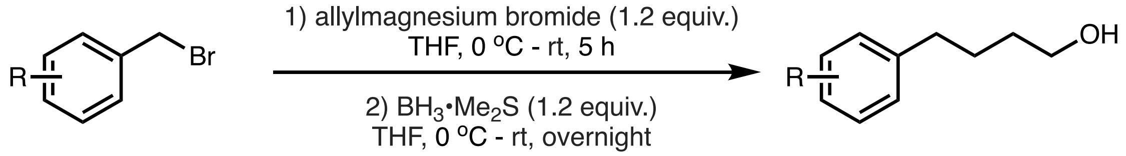

To a solution of alkyl bromide (5 mmol, 1 equiv.) in THF (15 mL) in a flame-dried 100 mL round-bottomed flask under N_2 was added allylmagnesium bromide (7.5 mmol, 1 M in diethylether) dropwise at 0°C. The reaction was monitored by TLC and after consumption of all starting material (around 4 hours for both substrates) the reaction was quenched with saturated NH_4Cl solution and then extracted with DCM (x 3). The organics were combined, dried over $MgSO_4$, filtered and concentrated *in vacuo* yielding crude residue. The crude residue was used for the next step without further purification following general procedure B, Step 2 using crude residues, THF (10 mL) and BH_3•Me_2S solution (3 mL, 6 mmol, 2 M in THF). The crude residues were purified by flash column chromatography (eluent = 5 to 15% EtOAc in hexanes, silica gel) to afford both products as colourless oil (**39** = 513 mg, 61% yield, **41** = 447 mg, 41% yield).

(S38)

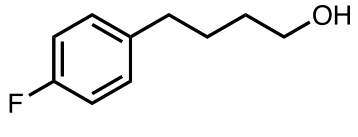

R_f = 0.58 (eluent = 10% EtOAc in hexanes); **^{1}H NMR (500 MHz, $CDCl_3$)** δ 7.14 – 7.11 (m, 2H), 6.98 – 6.93 (m, 2H), 3.67 – 3.62 (t, *J* = 6.4 Hz, 2H), 2.63 – 2.60 (t, *J* = 7.5 Hz, 2H), 1.71 – 1.64 (m, 2H), 1.62 – 1.56 (m, 2H), 1.25 (s, 1H); **^{13}C NMR (126 MHz, $CDCl_3$)** δ 161.3 (d, *J* = 243.2 Hz), 138.0 (d, *J* = 3.2 Hz), 129.8, 115.1 (d, *J* = 21.0 Hz), 62.9, 34.9, 32.3, 27.8; **^{19}F NMR (471 MHz, $CDCl_3$)** δ -117.94.

Data consistent with the literature.[6]

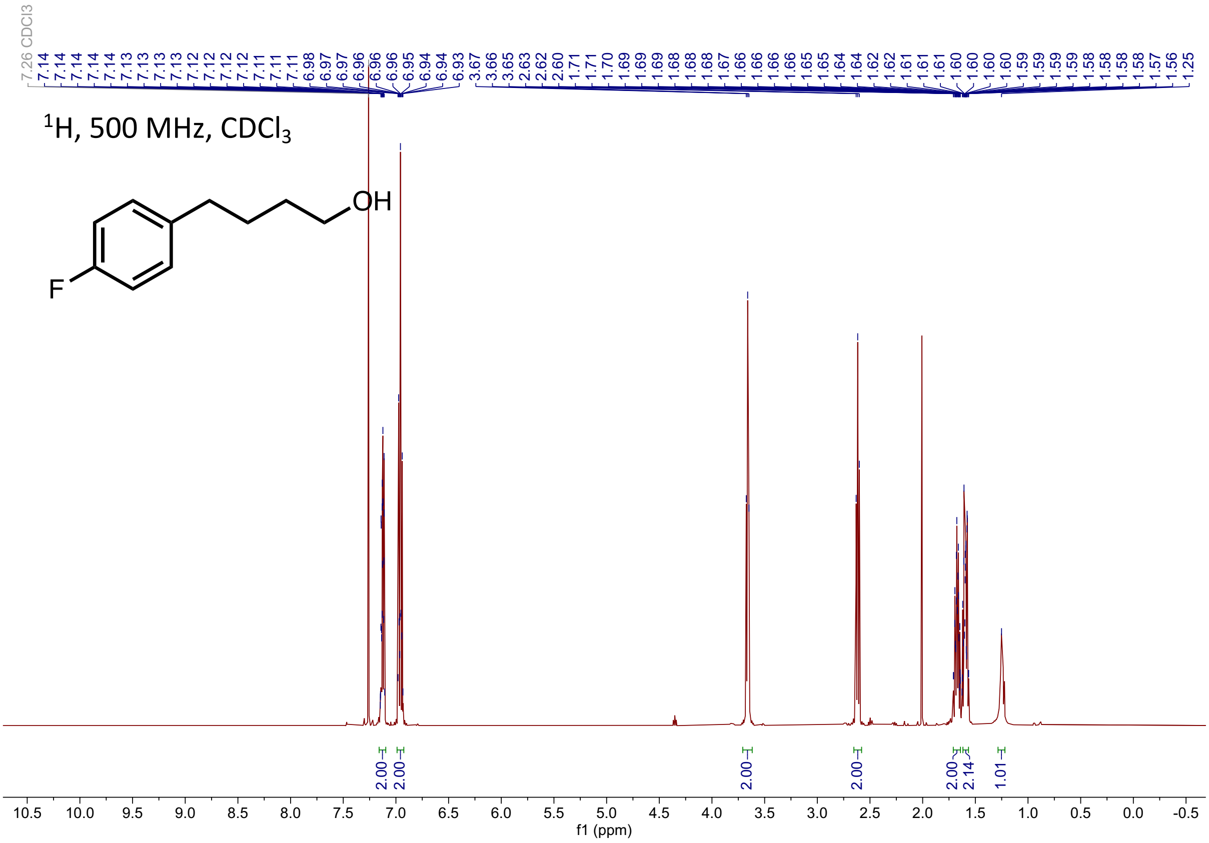
7.26 CDCl3
7.14 7.14 7.14 7.14 7.13 7.13 7.13 7.12 7.12 7.12 7.11 7.11 7.11 6.98 6.97 6.97 6.96 6.96 6.96 6.95 6.94 6.94 6.93 3.67 3.66 3.65 2.63 2.62 2.60 1.71 1.71 1.70 1.69 1.69 1.68 1.68 1.67 1.66 1.66 1.66 1.65 1.65 1.64 1.64 1.62 1.62 1.61 1.61 1.61 1.60 1.60 1.60 1.60 1.59 1.59 1.59 1.59 1.58 1.58 1.58 1.57 1.56 1.25
1H, 500 MHz, CDCl3
OH
F
2.00 2.00 2.00 2.00 2.00 2.14 1.01
10.5 10.0 9.5 9.0 8.5 8.0 7.5 7.0 6.5 6.0 5.5 5.0 4.5 4.0 3.5 3.0 2.5 2.0 1.5 1.0 0.5 0.0 -0.5
f1 (ppm)

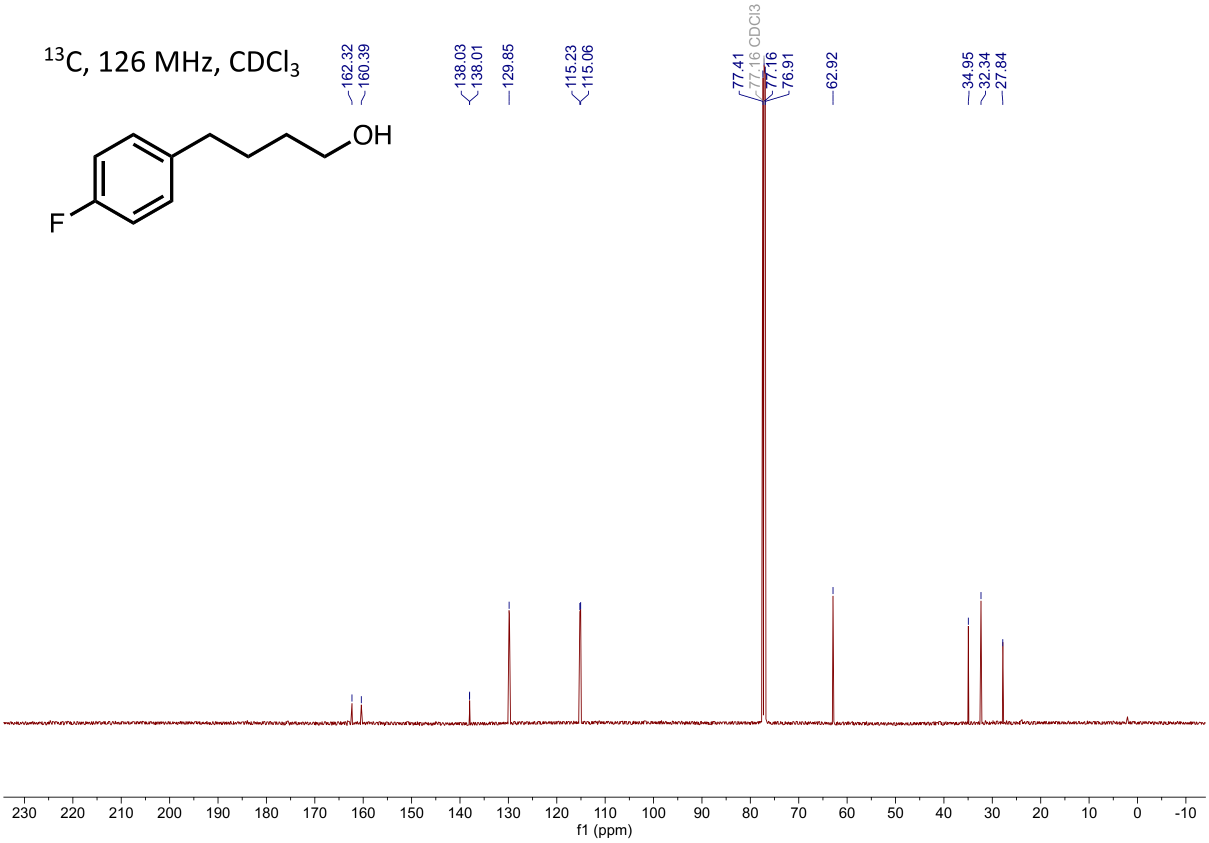
13C, 126 MHz, CDCl3
162.32 160.39 138.03 138.01 129.85 115.23 115.06 77.41 77.16 CDCl3 77.16 76.91 62.92 34.95 32.34 27.84
OH
F
230 220 210 200 190 180 170 160 150 140 130 120 110 100 90 80 70 60 50 40 30 20 10 0 -10
f1 (ppm)

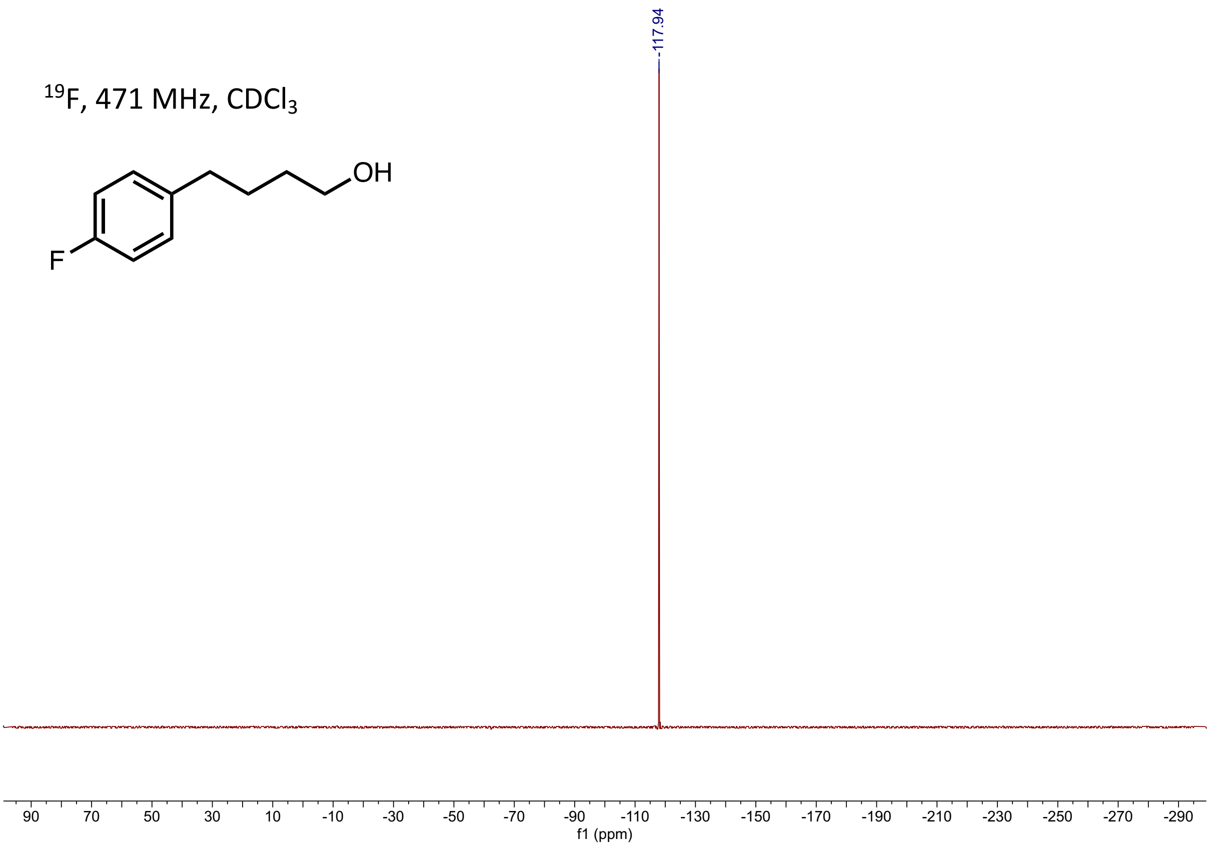
-117.94
^{19}F, 471 MHz, $CDCl_3$
OH
F
90 70 50 30 10 -10 -30 -50 -70 -90 -110 -130 -150 -170 -190 -210 -230 -250 -270 -290
f1 (ppm)

(S40)

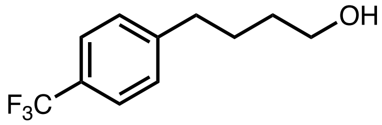

R$_f$ = 0.51 (eluent = 10% EtOAc in hexanes); **^{1}H NMR (500 MHz, CDCl$_3$)** δ 7.54 – 7.52 (m, 2H), 7.30 – 7.28 (m, 2H), 3.68 – 3.66 (t, *J* = 6.5, 0.6 Hz, 2H), 2.72 – 2.69 (t, *J* = 7.6 Hz, 2H), 1.75 – 1.69 (m, 2H), 1.63 – 1.58 (m, 2H); **^{13}C NMR (126 MHz, CDCl$_3$)** δ 146.5 (d, *J* = 1.6 Hz), 128.8, 125.3 (q, *J* = 3.8 Hz), 62.7, 35.6, 32.2, 27.4; **^{19}F NMR (471 MHz, CDCl$_3$)** δ -62.28.

Data consistent with the literature.[7]

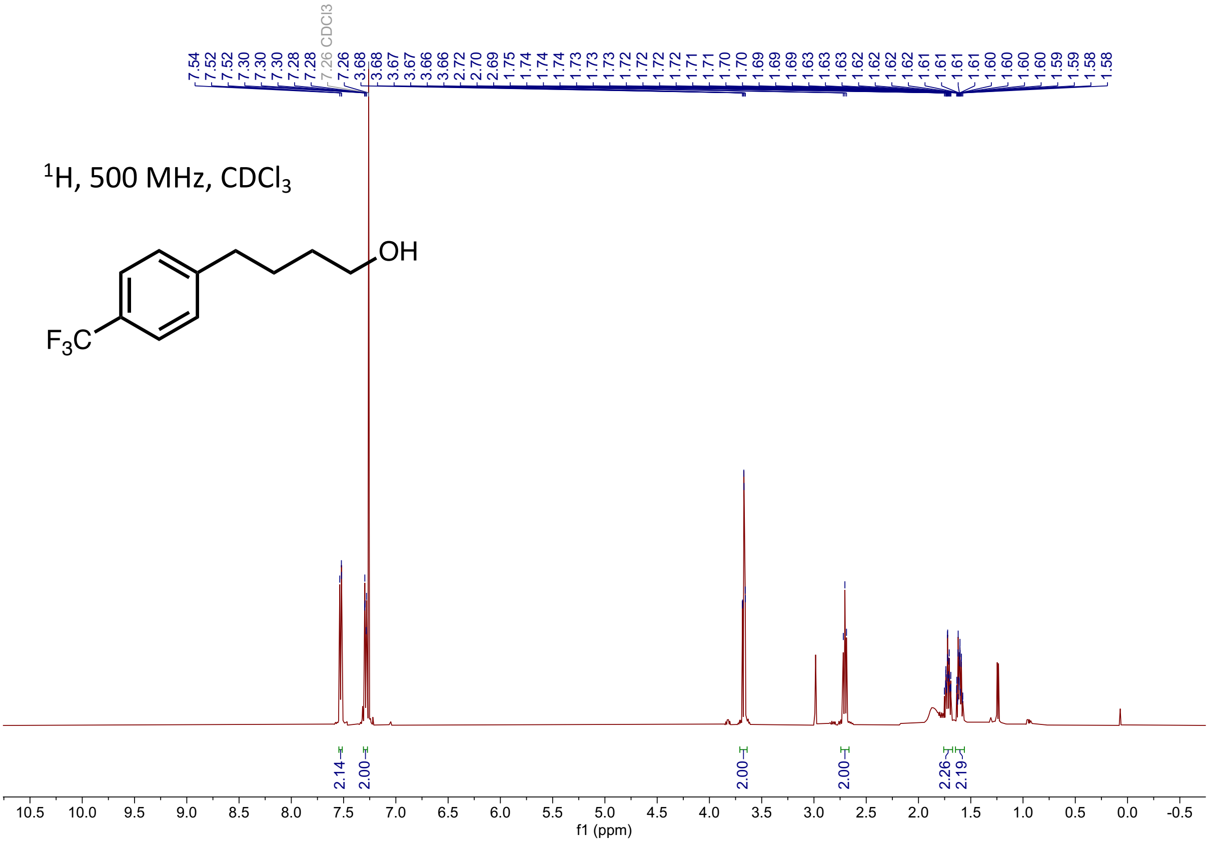

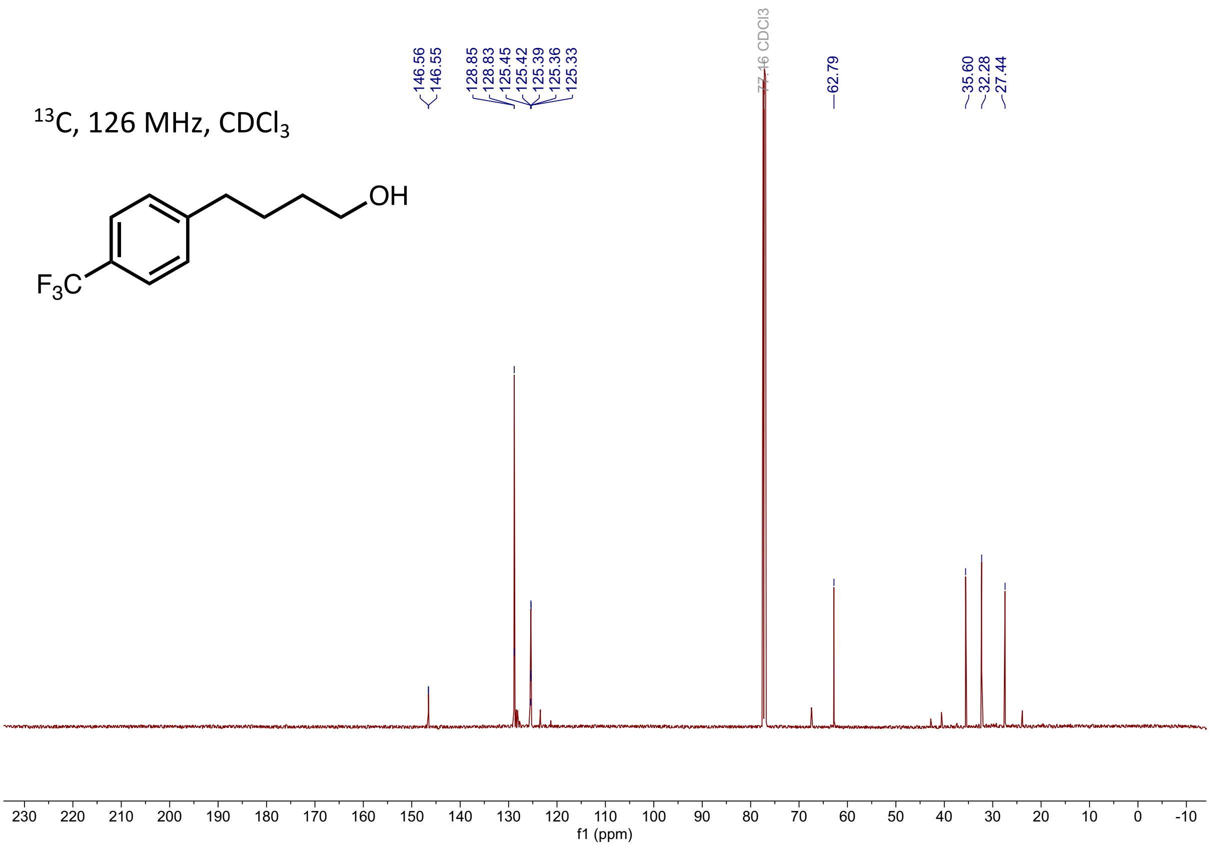

^{13}C, 126 MHz, $CDCl_3$
OH
F_3C
146.56
146.55
128.85
128.83
125.45
125.42
125.39
125.36
125.33
77.16 CDCl3
62.79
35.60
32.28
27.44
230 220 210 200 190 180 170 160 150 140 130 120 110 100 90 80 70 60 50 40 30 20 10 0 -10
f1 (ppm)

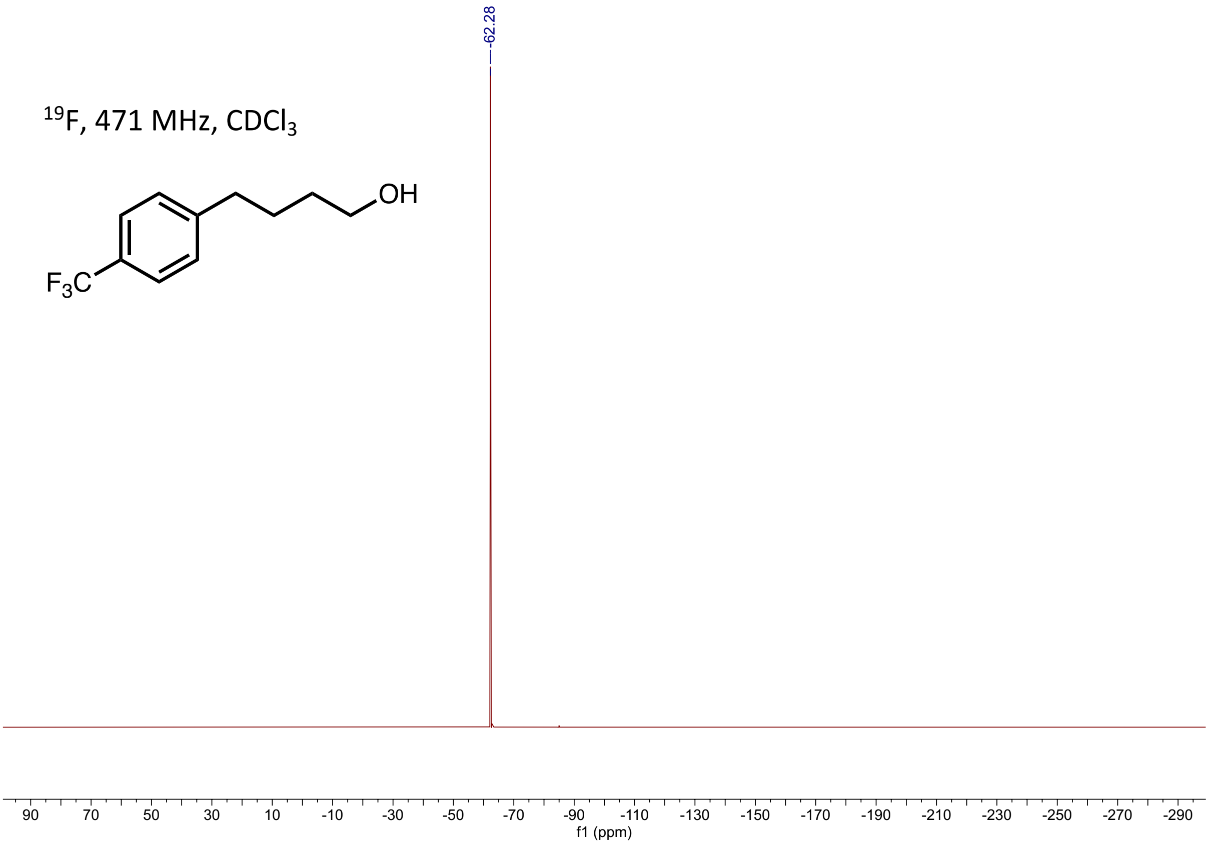

^{19}F, 471 MHz, $CDCl_3$
OH
F_3C
-62.28
90 70 50 30 10 -10 -30 -50 -70 -90 -110 -130 -150 -170 -190 -210 -230 -250 -270 -290
f1 (ppm)

Characterisation of Products

Electrochemical General Procedure:

To an oven-dried 10 mL ElectraSyn vial equipped with a magnetic stirrer bar, was added substrate (0.30 mmol) and *n*-Bu_4NClO_4 (103 mg, 0.30 mmol). The threaded glass of the vial was wrapped with PTFE tape and connected to the ElectraSyn cap, which was fitted with a graphite anode and a platinum cathode. The vial was purged with N_2 gas via evacuate-refill cycles (× 3). Dichloromethane (5.7 mL) was added, followed by TFE (0.3 mL) and the mixture was stirred to facilitate dissolution. The mixture was then purged *via* bubbling with N_2 gas for 3 minutes. The vial was then connected to an ElectraSyn. Electrolysis at 7.5 mA was conducted until 2.0 F of charge had been passed, under N_2 with continuous stirring. After electrolysis was complete mesitylene internal standard (42 µL, 0.30 mmol, 1 equiv.) was added to the reaction mixture followed by sampling for crude ^{1}H NMR analysis. The mixture was then concentrated *in vacuo* and the crude residue was purified by flash column chromatography on silica gel.

$$\%\ \text{NMR Yield of the Products} = \left(\frac{\text{Compound Integral}}{\text{Standard Integral}}\right) \times \left(\frac{\text{Standard Proton}}{\text{Compound Proton}}\right) \times 100$$

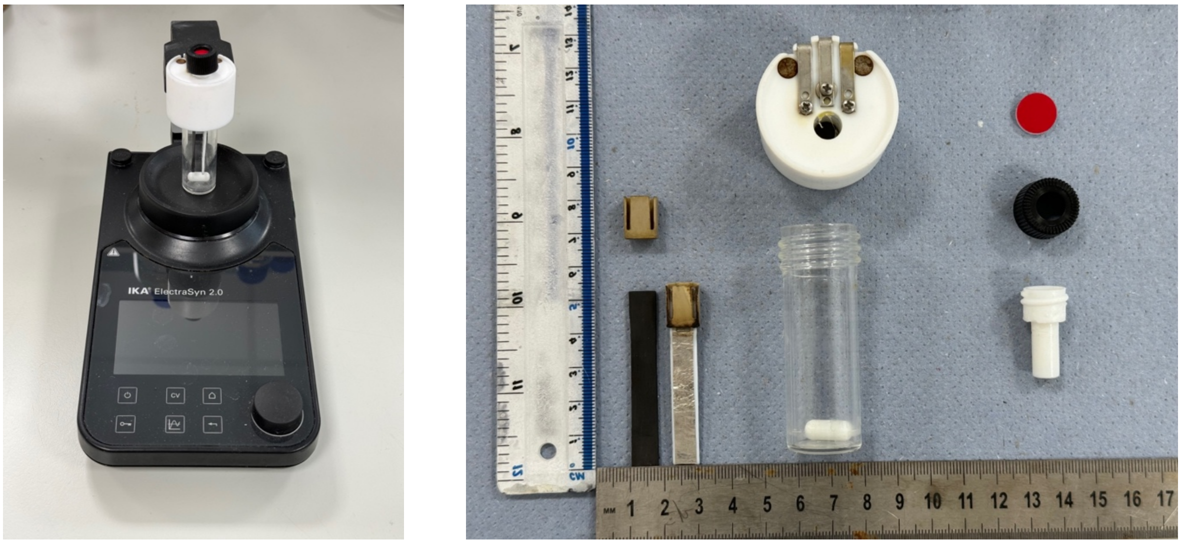

Figure S3. *Reaction set up - IKA ElectraSyn 2.0 fitted with assembled reaction cell (Left); Disassembled reaction cell (10 mL vial) with exemplar electrodes - graphite anode and platinum cathode (Right).*

Faradaic Efficiency:

$$\% \text{ Faradaic Efficiency} = \frac{\text{Actual moles of product}}{\text{Theoretical moles of product}} \times 100$$

$$\text{Theoretical moles of product (based on charge passed)} = \frac{Q}{n \times F}$$

Where:

Q = the total charge passed (in Coulombs) = current (I) x reaction time (t).

n = the number of moles of electrons required per mole of product.

F = Faraday's constant (96,485 C/mol).

Example (Faradaic Efficiency of model product **2**):

Q = 0.0075 A x 7720 s = 57.9 C

$$\text{Theoretical moles of product} = \frac{57.9 \text{ C}}{2 \times 96{,}485 \text{ C/mol}} = 0.0003 \text{ mol}$$

$$\% \text{ Faradaic Efficiency} = \frac{0.00027 \text{ mol (90\% nmr yield)}}{0.0003 \text{ mol}} \times 100 = 90\%$$

(2)

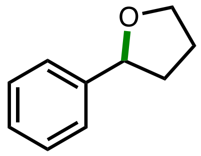

Prepared according to electrochemical general procedure using **1** and **(*S*)-1** (62.5 mg, 0.30 mmol, 1 equiv.), *n*-Bu_4ClO_4 (103 mg, 0.30 mmol, 1 equiv.), dichloromethane (5.7 mL) and TFE (0.3 mL). The yield was determined by crude ^{1}H NMR using 1,3,5-trimethylbenzene (42 µL, 1 equiv.) as an internal standard: 90%. Purification by flash column chromatography (eluent = 5 to 15% EtOAc in hexanes, silica gel) to afford product as colourless oil (38.7 mg, 87% yield) as a racemate with both substrates.

R_f = 0.32 (eluent = 10% EtOAc in hexanes); **^{1}H NMR (500 MHz, $CDCl_3$)** δ 7.35 – 7.33 (m, 4H), 7.27 – 7.24 (m, 1H), 4.92 – 4.89 (t, *J* = 7.2 Hz, 1H), 4.13 – 4.08 (m, 1H), 3.97 – 3.92 (m, 1H), 2.35 – 2.31 (m, 1H), 2.05 – 1.99 (m, 2H), 1.84 – 1.79 (m, 1H); **^{13}C NMR (126 MHz, CDCl3)** δ 143.5, 128.4, 127.2, 125.7, 80.8, 68.8, 34.7, 26.1.

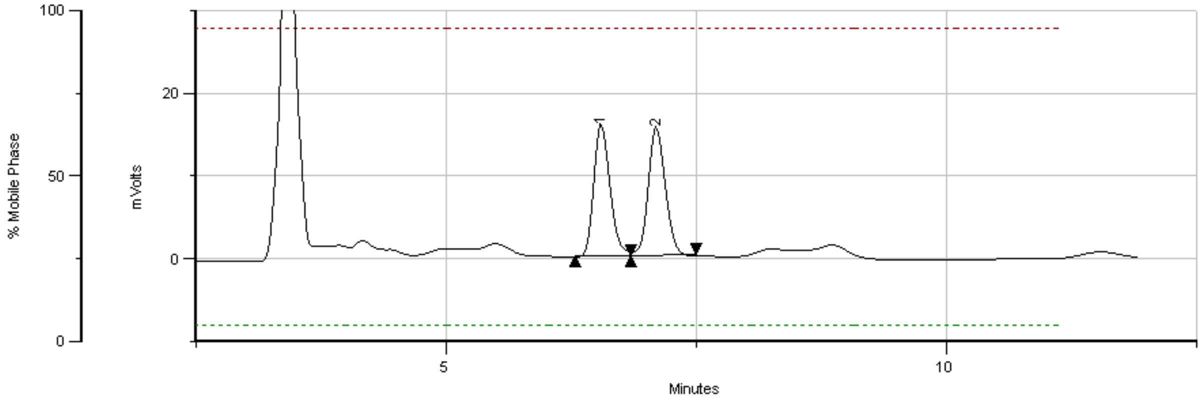

	Inj. Number	Peak Name	R. Time	Area	Area %
1	1.00	*1	6.55	303620.28	49.36
2	1.00	2	7.10	311470.97	50.64

Figure S4. *HPLC trace for racemic* **2**. *HPLC Chiralpak IC column, 211 nm, 25 °C, 95:5 Hexane/IPA, flow rate 1 mL/min, enantiomer 1 = 6.5 min, enantiomer 2 = 7.1 min.*

Data consistent with the literature.[8]

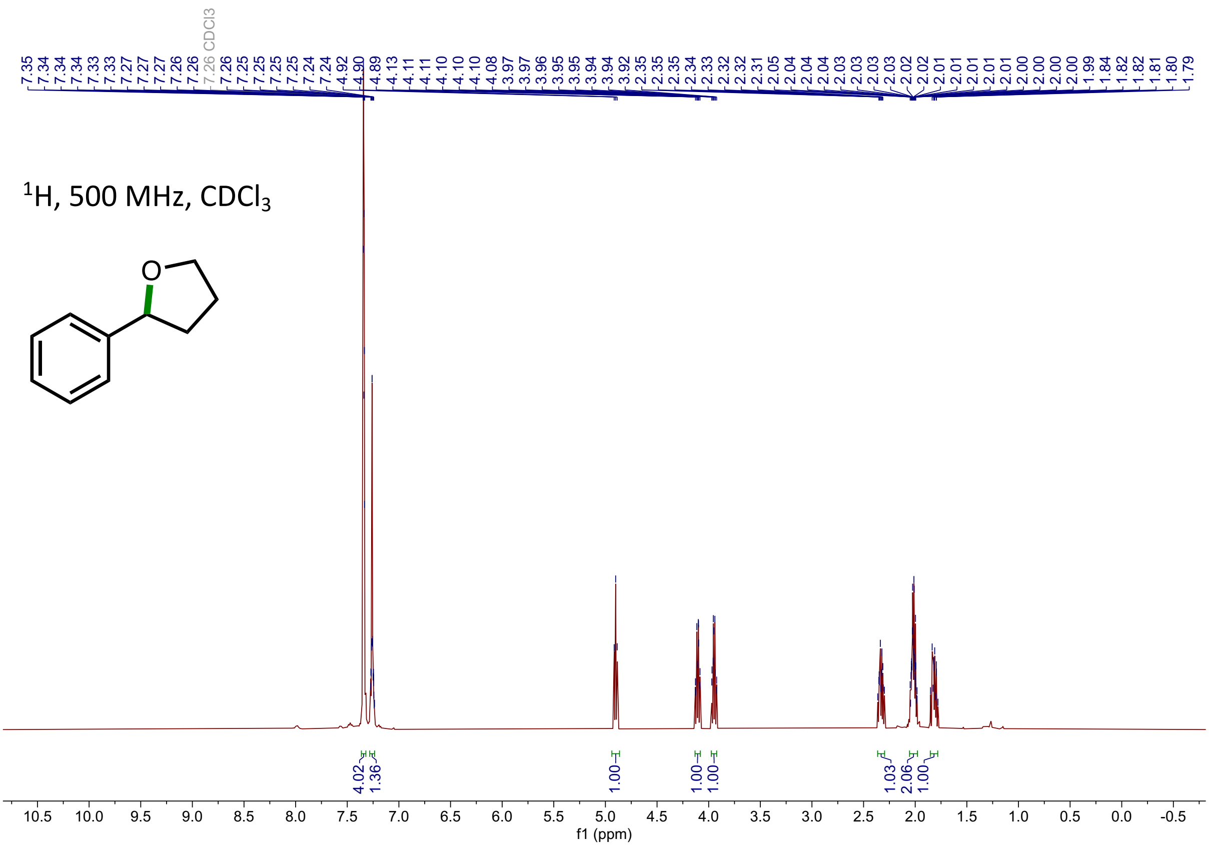
7.35 7.34 7.34 7.33 7.33 7.27 7.27 7.27 7.26 7.26 CDCl3 7.26 7.25 7.25 7.25 7.25 7.24 7.24 4.92 4.90 4.89 4.13 4.11 4.11 4.10 4.10 4.08 3.97 3.97 3.96 3.95 3.95 3.94 3.94 3.92 2.35 2.35 2.35 2.34 2.33 2.32 2.32 2.31 2.05 2.04 2.04 2.04 2.03 2.03 2.03 2.03 2.02 2.02 2.01 2.01 2.01 2.01 2.01 2.00 2.00 2.00 2.00 1.99 1.84 1.82 1.82 1.81 1.80 1.79
^{1}H, 500 MHz, CDCl$_3$
O
4.02 1.36 1.00 1.00 1.00 1.03 2.06 1.00
f1 (ppm)

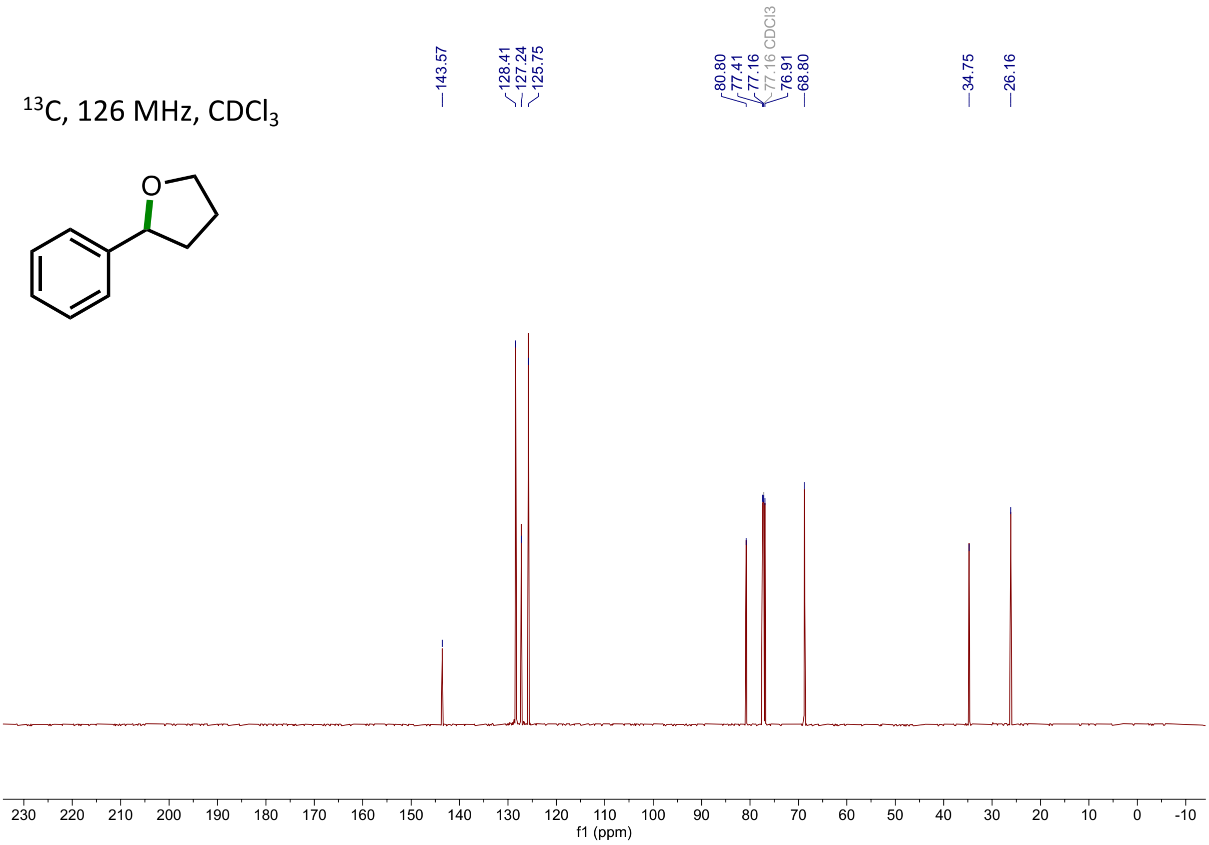
143.57 128.41 127.24 125.75 80.80 77.41 77.16 77.16 CDCl3 76.91 68.80 34.75 26.16
^{13}C, 126 MHz, CDCl$_3$
O
f1 (ppm)

(P4)

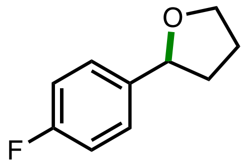

Prepared according to electrochemical general procedure using **S4** (67.9 mg, 0.30 mmol, 1 equiv.), *n*-Bu_4ClO_4 (103 mg, 0.30 mmol, 1 equiv.), dichloromethane (5.7 mL) and TFE (0.3 mL). The yield was determined by crude ^{1}H NMR using 1,3,5-trimethylbenzene (42 µL, 1 equiv.) as an internal standard: 66%. Purification by flash column chromatography (eluent = 5 to 15% EtOAc in hexanes, silica gel) to afford product as pale-yellow oil (31 mg, 62% yield).

R_f = 0.51 (eluent = 10% EtOAc in hexanes); **^{1}H NMR (500 MHz, $CDCl_3$)** δ 7.32 – 7.28 (m, 2H), 7.03 – 6.99 (m, 2H), 4.87 – 4.84 (t, *J* = 7.2 Hz, 1H), 4.11 – 4.06 (m, 1H), 3.95 – 3.90 (m, 1H), 2.34 – 2.27 (m, 1H), 2.04 – 1.98 (m, 2H), 1.80 – 1.73 (m, 1H); **^{13}C NMR (126 MHz, CDCl3)** δ 162.1 (d, *J* = 244.5 Hz), 139.2, 127.4 (d, *J* = 8.0 Hz), 115.2 (d, *J* = 21.3 Hz), 80.2, 68.7, 34.8, 26.1; **^{19}F NMR (471 MHz, $CDCl_3$)** δ -115.93.

Data consistent with the literature.[9]

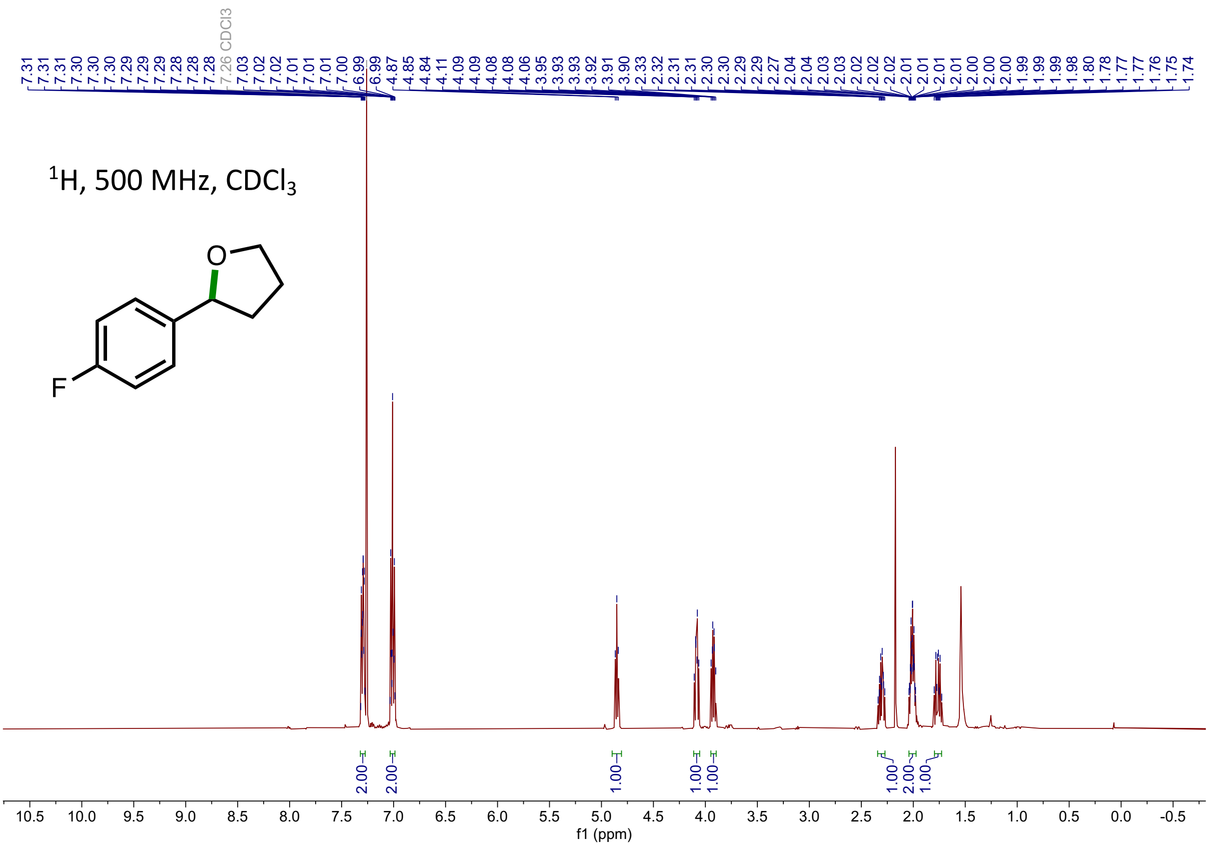

7.31 7.31 7.31 7.30 7.30 7.29 7.29 7.29 7.28 7.28 7.28 7.26 CDCl3 7.03 7.02 7.02 7.01 7.01 7.01 7.00 6.99 6.99 4.87 4.85 4.84 4.11 4.09 4.09 4.08 4.08 4.06 3.95 3.93 3.93 3.92 3.91 3.90 2.33 2.32 2.31 2.31 2.30 2.30 2.29 2.29 2.27 2.04 2.03 2.03 2.02 2.02 2.02 2.01 2.01 2.01 2.01 2.00 2.00 2.00 1.99 1.99 1.99 1.98 1.80 1.78 1.77 1.77 1.76 1.75 1.74
^{1}H, 500 MHz, CDCl$_3$
O
F
2.00 2.00 1.00 1.00 1.00 1.00 2.00 1.00
10.5 10.0 9.5 9.0 8.5 8.0 7.5 7.0 6.5 6.0 5.5 5.0 4.5 4.0 3.5 3.0 2.5 2.0 1.5 1.0 0.5 0.0 -0.5
f1 (ppm)

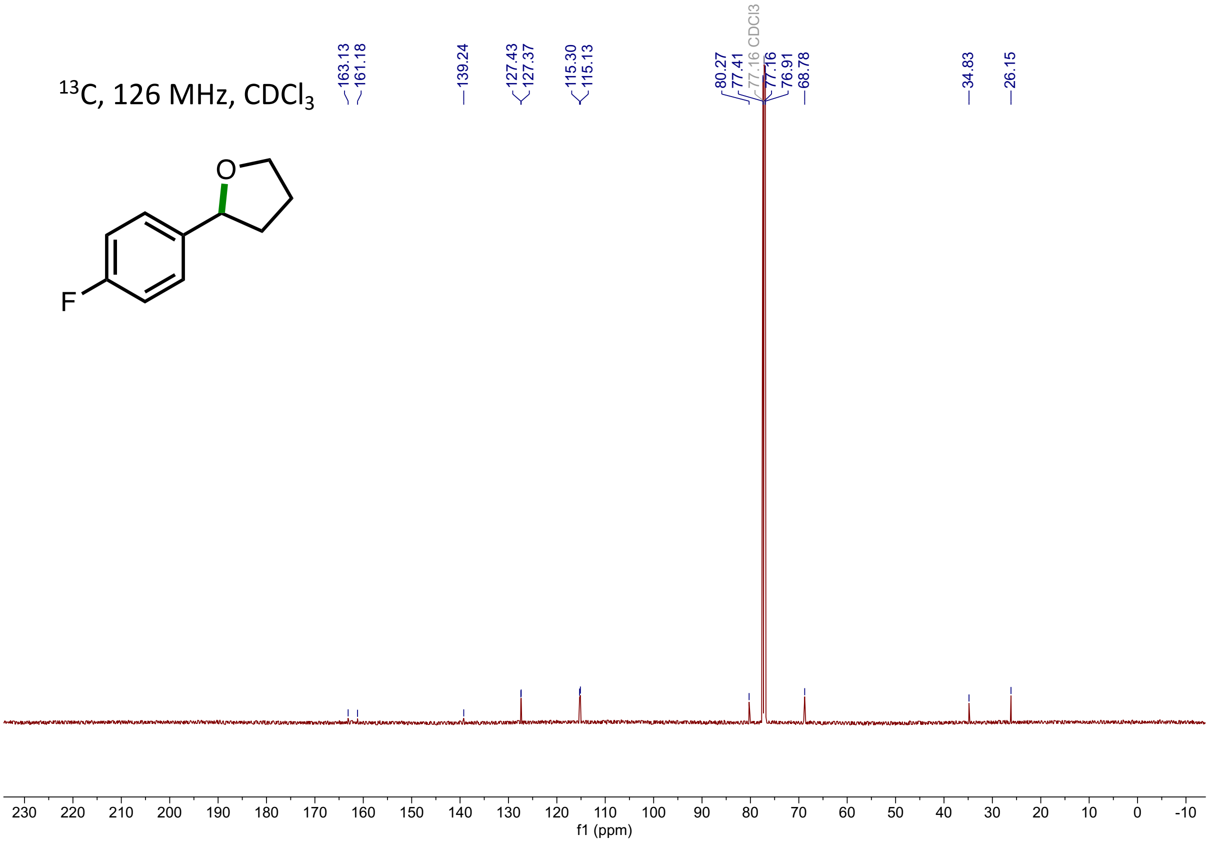

163.13 161.18 139.24 127.43 127.37 115.30 115.13 80.27 77.41 77.16 CDCl3 77.16 76.91 68.78 34.83 26.15
^{13}C, 126 MHz, CDCl$_3$
O
F
230 220 210 200 190 180 170 160 150 140 130 120 110 100 90 80 70 60 50 40 30 20 10 0 -10
f1 (ppm)

^{19}F, 471 MHz, $CDCl_3$

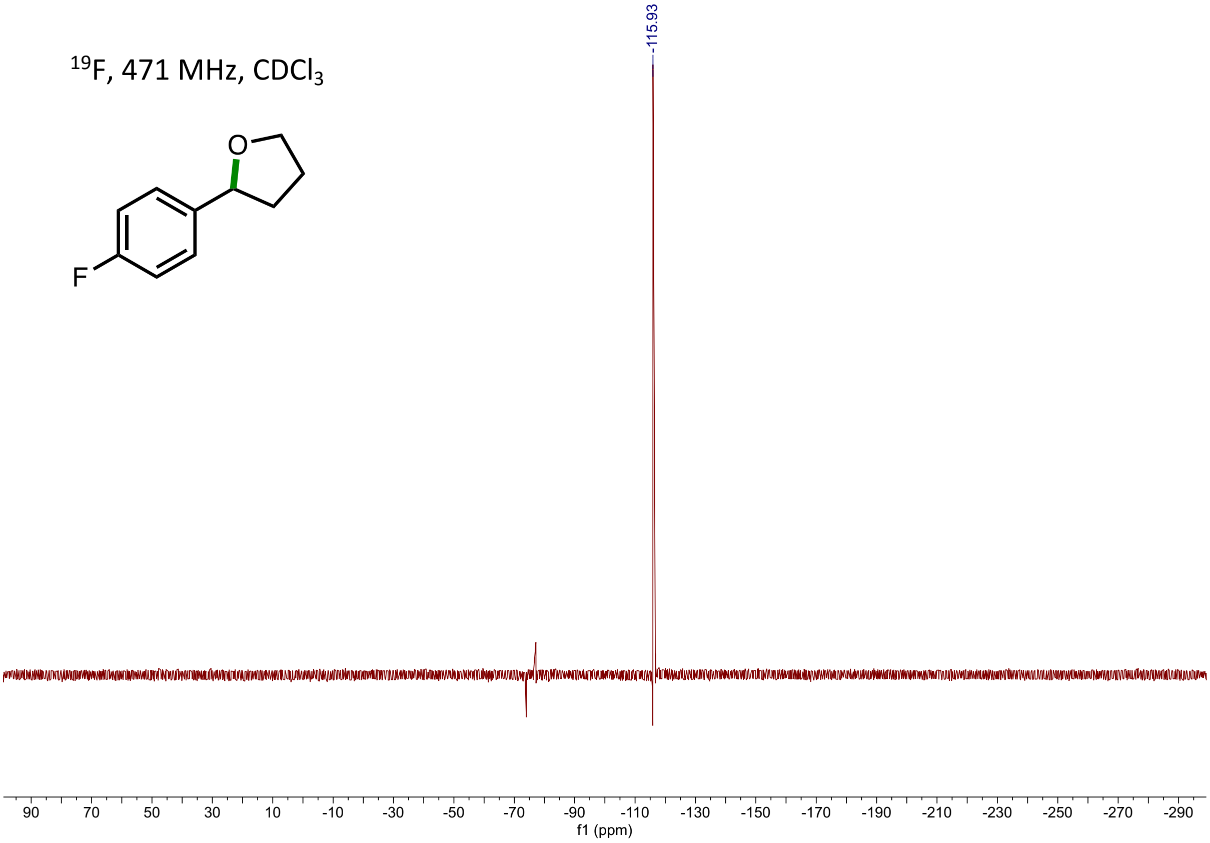

(P5)

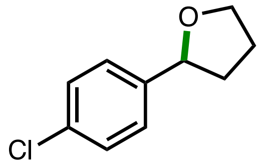

Prepared according to electrochemical general procedure using **S5** (72.8 mg, 0.30 mmol, 1 equiv.), *n*-Bu_4ClO_4 (103 mg, 0.30 mmol, 1 equiv.), dichloromethane (5.7 mL) and TFE (0.3 mL). The yield was determined by crude ^{1}H NMR using 1,3,5-trimethylbenzene (42 µL, 1 equiv.) as an internal standard: 74%. Purification by flash column chromatography (eluent = 5 to 15% EtOAc in hexanes, silica gel) to afford product as pale-yellow oil (38.3 mg, 70% yield).

R_f = 0.48 (eluent = 10% EtOAc in hexanes); **^{1}H NMR (500 MHz, $CDCl_3$)** δ 7.28 – 7.22 (m, 4H), 4.84 – 4.82 (t, *J* = 7.2 Hz, 1H), 4.08 – 4.03 (m, 1H), 3.92 – 3.88 (m, 1H), 2.32 – 2.25 (m, 1H), 2.02 – 1.94 (m, 2H), 1.74 – 1.70 (m, 1H); **^{13}C NMR (126 MHz, CDCl3)** δ 142.1, 132.8, 128.5, 127.1, 80.1, 68.8, 34.8, 26.1.

Data consistent with the literature.[10]

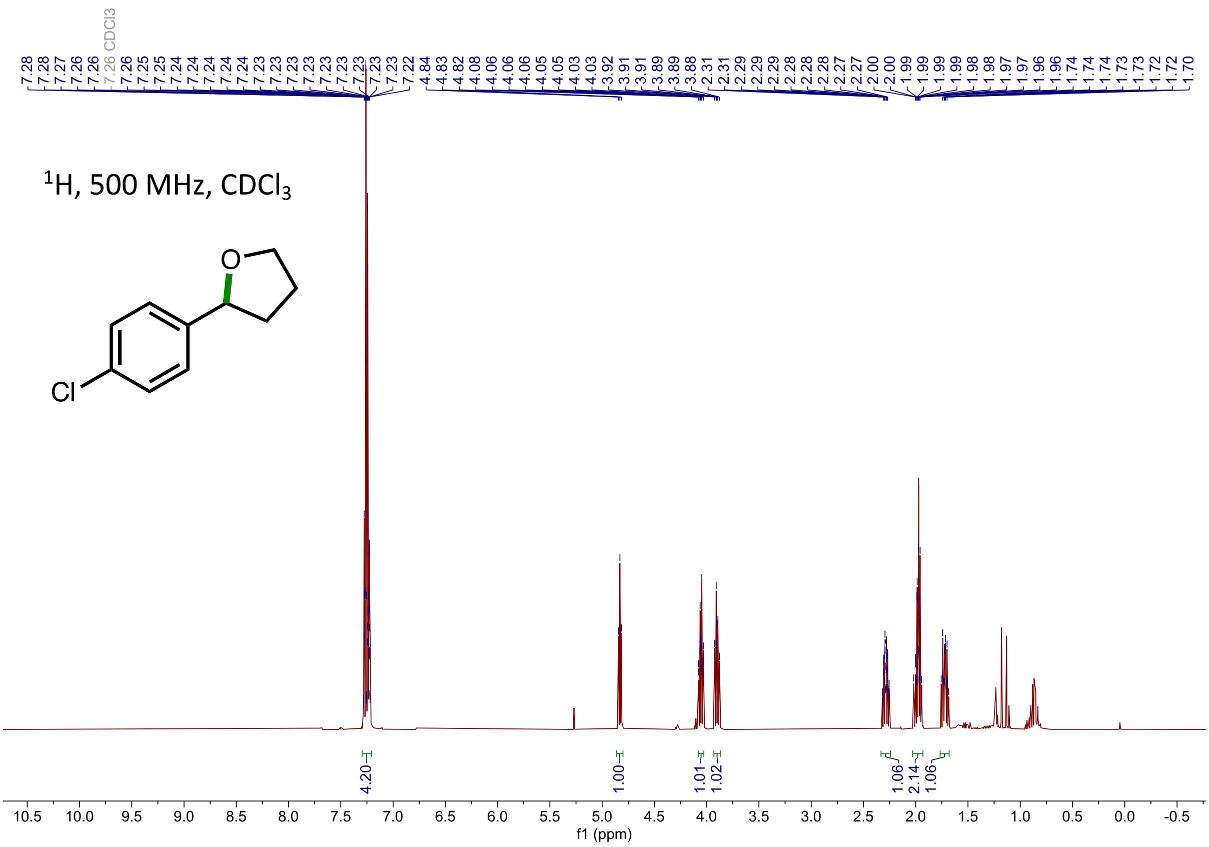

^{1}H, 500 MHz, CDCl$_3$
O
Cl
4.20
1.00
1.01
1.02
1.06
2.14
1.06
f1 (ppm)

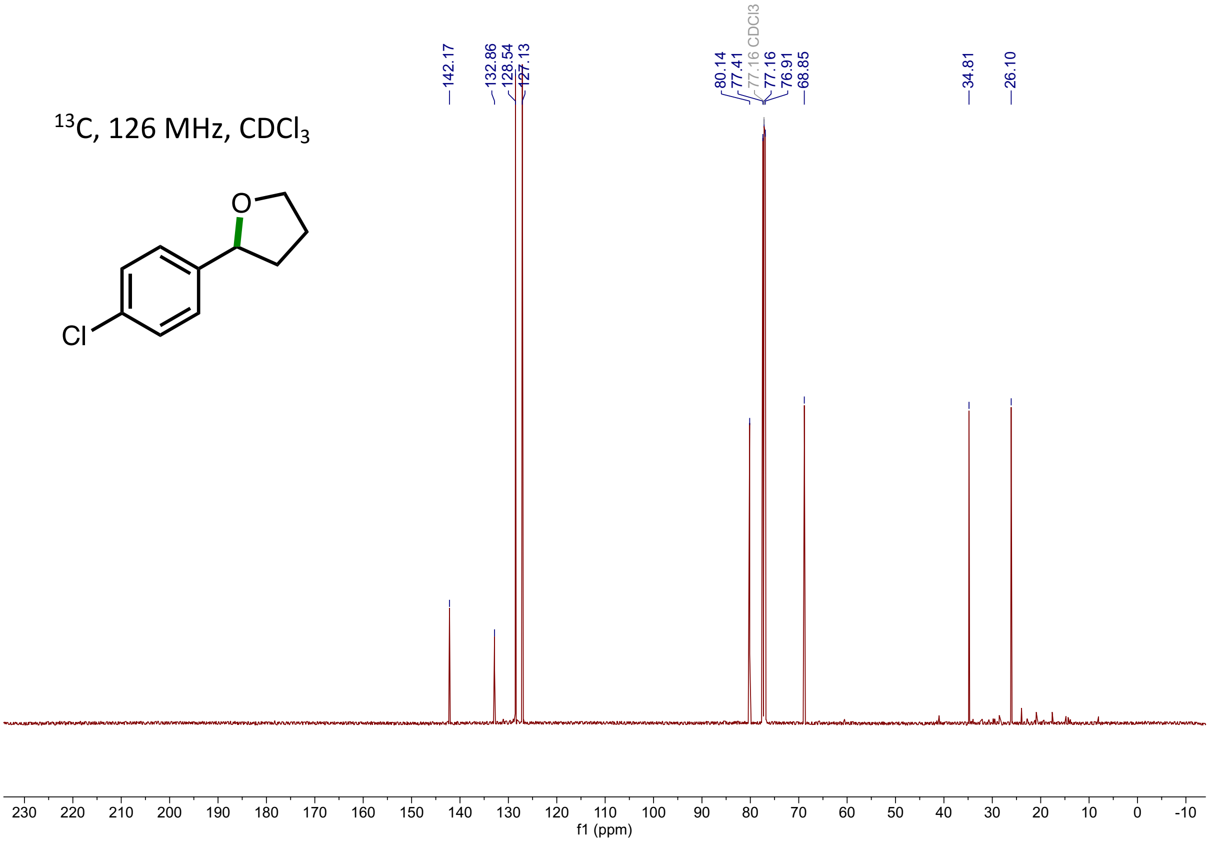

^{13}C, 126 MHz, CDCl$_3$
142.17
132.86
128.54
127.13
80.14
77.41
77.16 CDCl3
77.16
76.91
68.85
34.81
26.10
O
Cl
f1 (ppm)

(P6)

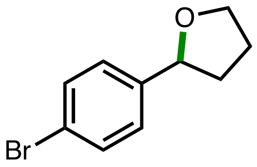

Prepared according to electrochemical general procedure using **S6** (86.2 mg, 0.30 mmol, 1 equiv.), *n*-Bu_4ClO_4 (103 mg, 0.30 mmol, 1 equiv.), dichloromethane (5.7 mL) and TFE (0.3 mL). The yield was determined by crude ^{1}H NMR using 1,3,5-trimethylbenzene (42 µL, 1 equiv.) as an internal standard: 68%. Purification by flash column chromatography (eluent = 5 to 15% EtOAc in hexanes, silica gel) to afford product as colourless oil (41 mg, 60% yield).

R_f = 0.60 (eluent = 10% EtOAc in hexanes); **^{1}H NMR (500 MHz, $CDCl_3$)** δ 7.46 – 7.43 (m, 2H), 7.22 – 7.20 (m, 2H), 4.86 – 4.83 (t, *J* = 7.2 Hz, 1H), 4.10 – 4.06 (m, 1H), 3.95 – 3.90 (m, 1H), 2.34 – 2.30 (m, 1H), 2.03 – 1.97 (m, 2H), 1.77 – 1.72 (m, 1H); **^{13}C NMR (126 MHz, CDCl3)** δ 142.7, 131.4, 127.4, 120.9, 80.1, 68.8, 34.7, 26.1.

Data consistent with the literature.[11]

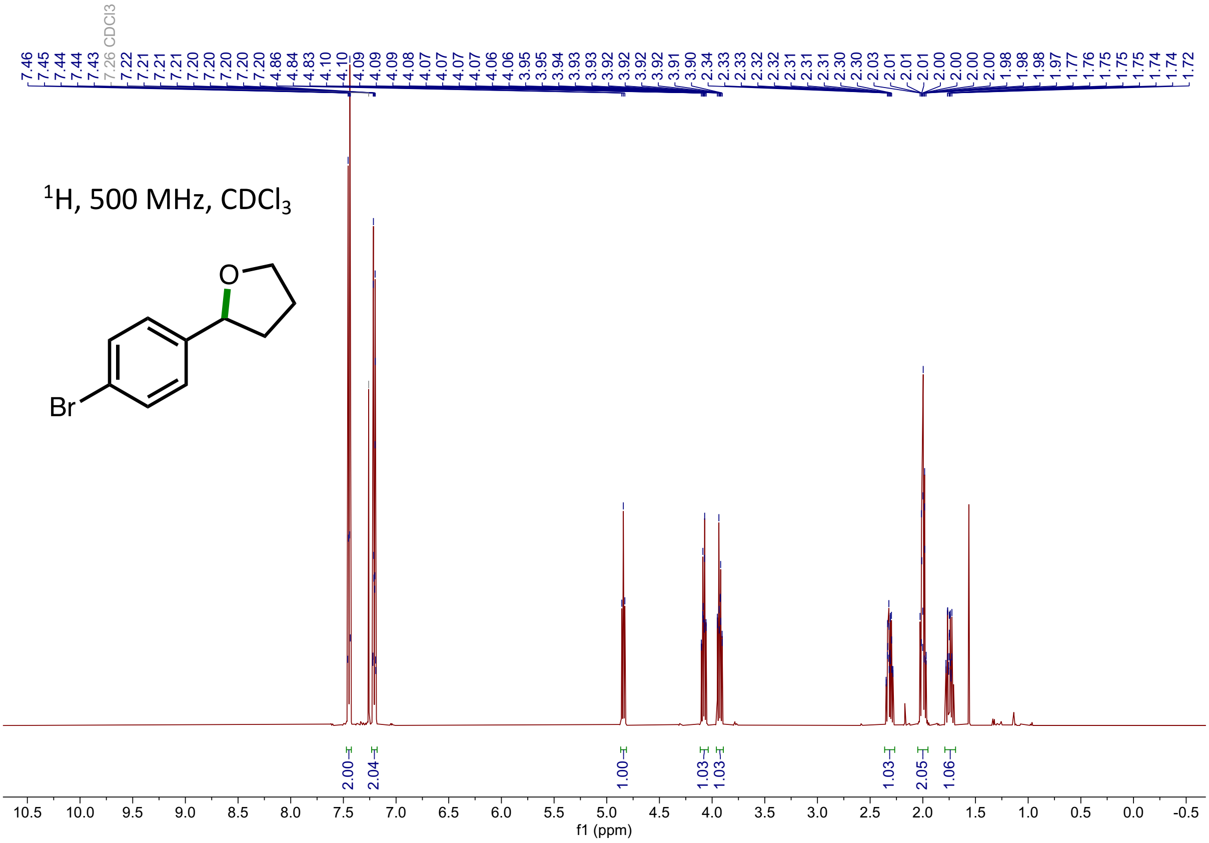

^{1}H, 500 MHz, CDCl$_3$
O
Br
7.46 7.45 7.44 7.43 7.26 CDCl3 7.22 7.21 7.21 7.21 7.20 7.20 7.20 7.20 4.86 4.84 4.83 4.10 4.10 4.09 4.09 4.09 4.08 4.07 4.07 4.07 4.06 4.06 3.95 3.95 3.94 3.93 3.93 3.92 3.92 3.92 3.92 3.91 3.90 2.34 2.33 2.33 2.32 2.32 2.31 2.31 2.30 2.30 2.03 2.01 2.01 2.01 2.00 2.00 2.00 1.98 1.98 1.97 1.77 1.76 1.75 1.75 1.74 1.74 1.72
2.00 2.04 1.00 1.03 1.03 1.03 2.05 1.06
f1 (ppm)

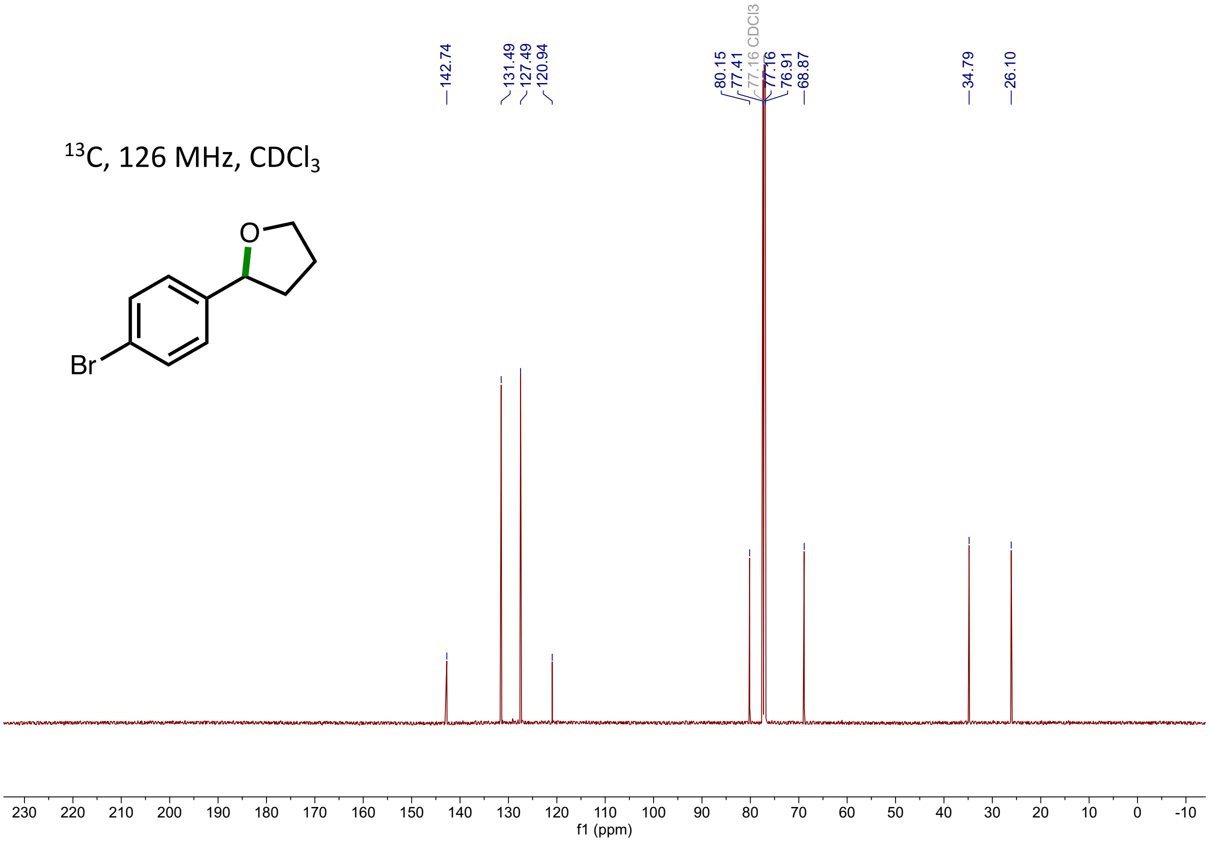

^{13}C, 126 MHz, CDCl$_3$
O
Br
142.74
131.49 127.49 120.94
80.15 77.41 77.16 CDCl3 76.91 68.87
34.79
26.10
f1 (ppm)

(P7)

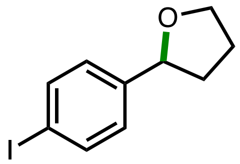

Prepared according to electrochemical general procedure using **S7** (100.3 mg, 0.30 mmol, 1 equiv.), *n*-Bu_4ClO_4 (103 mg, 0.30 mmol, 1 equiv.), dichloromethane (5.7 mL) and TFE (0.3 mL). The yield was determined by crude ^{1}H NMR using 1,3,5-trimethylbenzene (42 µL, 1 equiv.) as an internal standard: 67%. Purification by flash column chromatography (eluent = 5 to 15% EtOAc in hexanes, silica gel) to afford product as pale-yellow oil (52.0 mg, 63% yield).

R_f = 0.50 (eluent = 10% EtOAc in hexanes); **ν_{max} / cm^{-1}** (thin film) 2983, 2938, 2864, 2359, 2344, 1485, 1063, 1001, 814; **^{1}H NMR (500 MHz, $CDCl_3$)** δ 7.66 – 7.64 (m, 2H), 7.09 – 7.07 (m, 2H), 4.85 – 4.82 (t, *J* = 7.2 Hz, 1H), 4.10 – 4.05 (m, 1H), 3.95 – 3.90 (m, 1H), 2.33 – 2.29 (m, 1H), 2.02 – 1.96 (m, 2H), 1.76 – 1.72 (m, 1H); **^{13}C NMR (126 MHz, CDCl3)** δ 143.4, 137.4, 127.7, 92.4, 80.1, 68.8, 34.7, 26.0; **HRMS (EI^+) *m/z*** $[M - H]^+$ Calcd for $C_{10}H_{10}OI$ 272.9770; found 272.9768.

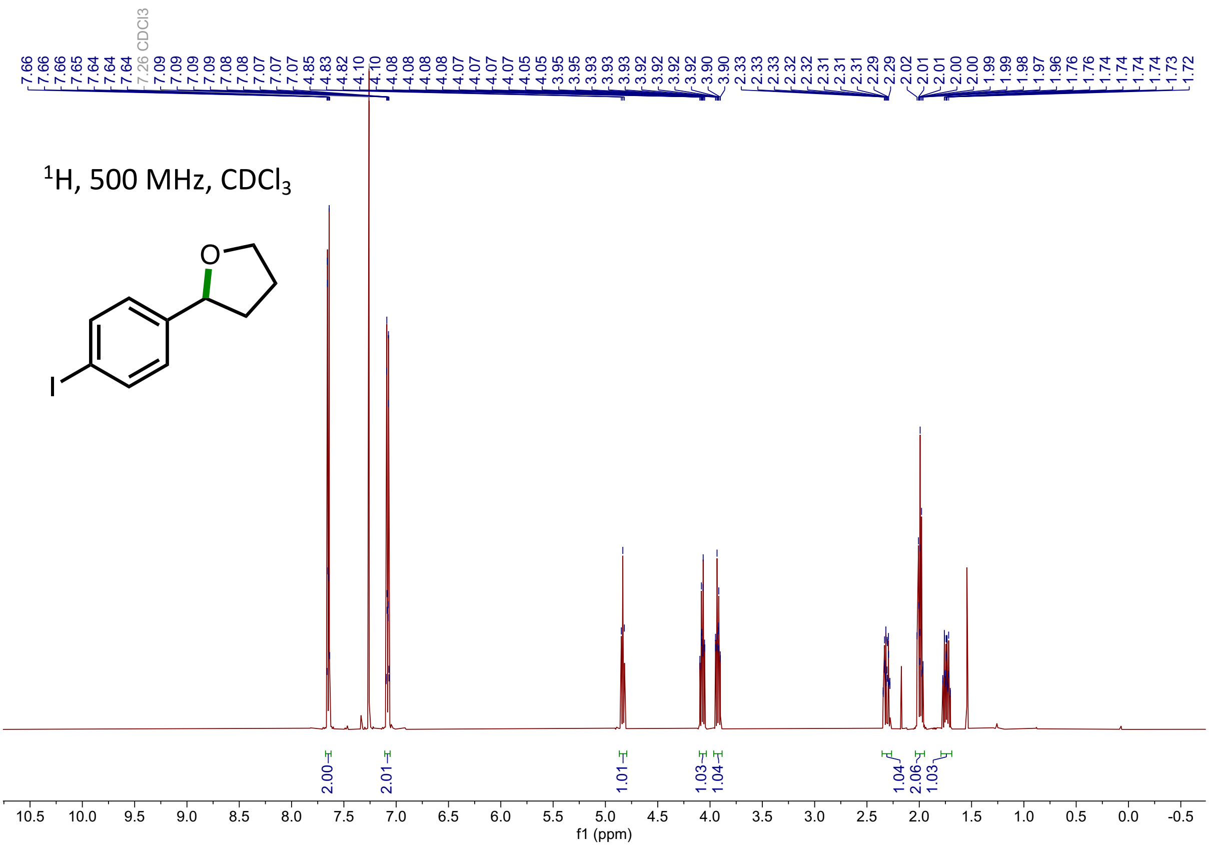
7.66
7.66
7.65
7.64
7.64
7.26 CDCl3
7.09
7.09
7.09
7.09
7.08
7.08
7.07
7.07
7.07
4.85
4.83
4.82
4.10
4.08
4.08
4.08
4.07
4.07
4.07
4.07
4.05
4.05
3.95
3.95
3.93
3.93
3.93
3.92
3.92
3.92
3.92
3.90
3.90
2.33
2.33
2.33
2.32
2.32
2.31
2.31
2.31
2.29
2.29
2.02
2.01
2.01
2.00
2.00
1.99
1.99
1.98
1.97
1.96
1.76
1.74
1.74
1.74
1.74
1.73
1.72
1H, 500 MHz, CDCl3
O
I
2.00
2.01
1.01
1.03
1.04
1.04
2.06
1.03
10.5 10.0 9.5 9.0 8.5 8.0 7.5 7.0 6.5 6.0 5.5 5.0 4.5 4.0 3.5 3.0 2.5 2.0 1.5 1.0 0.5 0.0 -0.5
f1 (ppm)

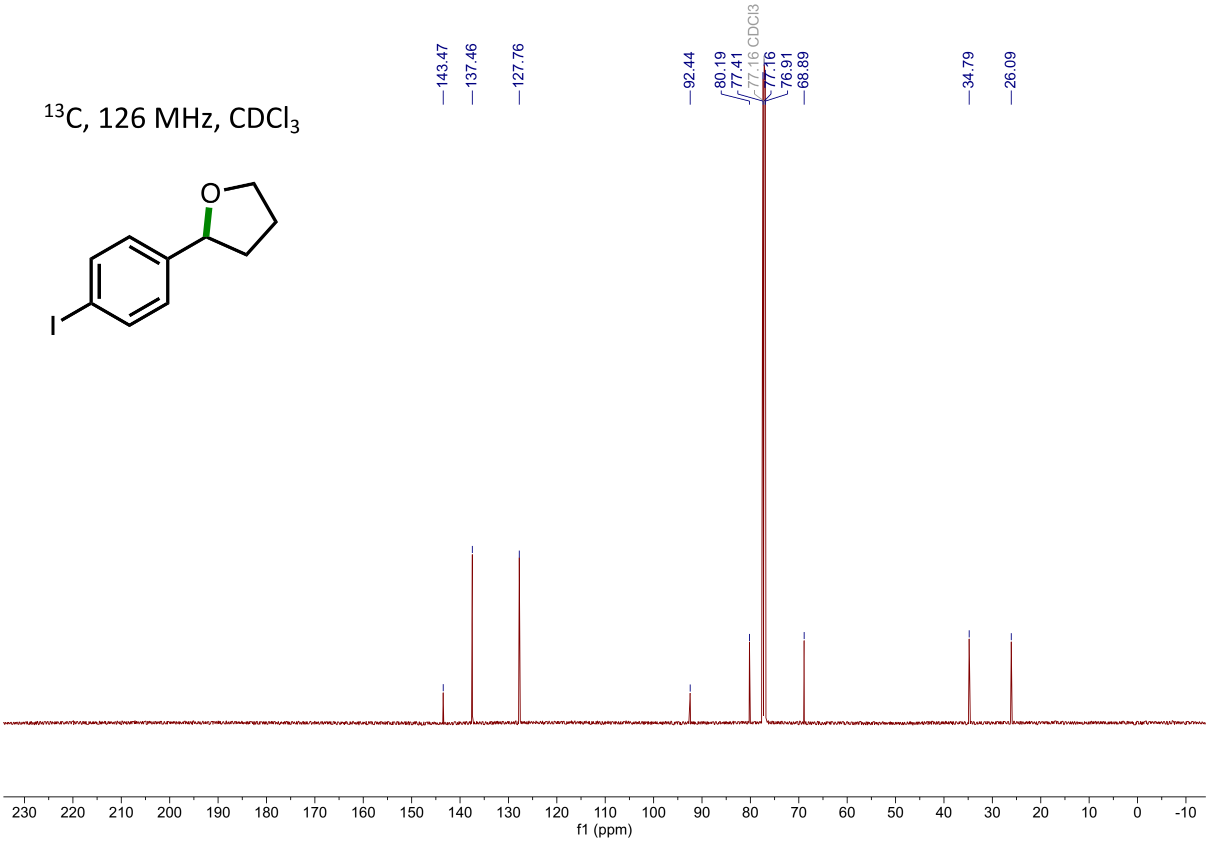
143.47
137.46
127.76
92.44
80.19
77.41
77.16 CDCl3
76.91
68.89
34.79
26.09
13C, 126 MHz, CDCl3
O
I
230 220 210 200 190 180 170 160 150 140 130 120 110 100 90 80 70 60 50 40 30 20 10 0 -10
f1 (ppm)

(8)

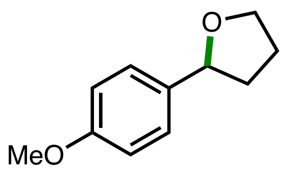

Prepared according to electrochemical general procedure using **S8** (71.5 mg, 0.30 mmol, 1 equiv.), *n*-Bu_4ClO_4 (103 mg, 0.30 mmol, 1 equiv.), dichloromethane (5.7 mL) and TFE (0.3 mL). The yield was determined by crude ^{1}H NMR using 1,3,5-trimethylbenzene (42 µL, 1 equiv.) as an internal standard: 96%. Purification by flash column chromatography (eluent = 5 to 15% EtOAc in hexanes, silica gel) to afford product as colourless oil (48.1 mg, 90% yield).

R_f = 0.61 (eluent = 10% EtOAc in hexanes); **^{1}H NMR (500 MHz, $CDCl_3$)** δ 7.28 – 7.25 (m, 2H), 6.88 – 6.86 (m, 2H), 4.84 – 4.81 (t, *J* = 7.7, 6.7 Hz, 1H), 4.10 – 4.06 (m, 1H), 3.93 – 3.88 (m, 1H), 3.80 (s, 3H), 2.31 – 2.24 (m, 1H), 2.06 – 1.95 (m, 2H), 1.83 – 1.77 (m, 1H); **^{13}C NMR (126 MHz, CDCl3)** δ 158.9, 135.4, 127.1, 113.3, 80.5, 68.6, 55.4, 34.6, 26.2.

Data consistent with the literature.[12]

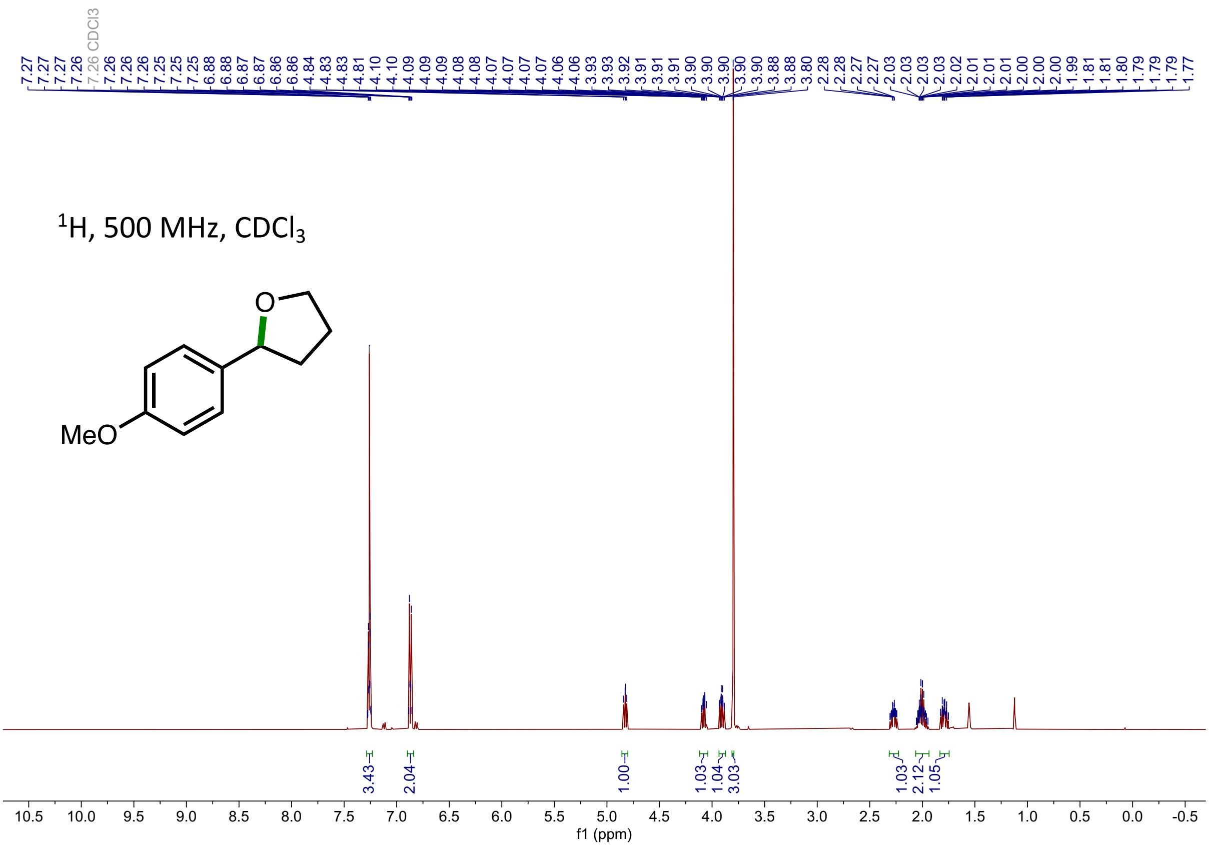

^{1}H, 500 MHz, CDCl$_3$
MeO
O
f1 (ppm)

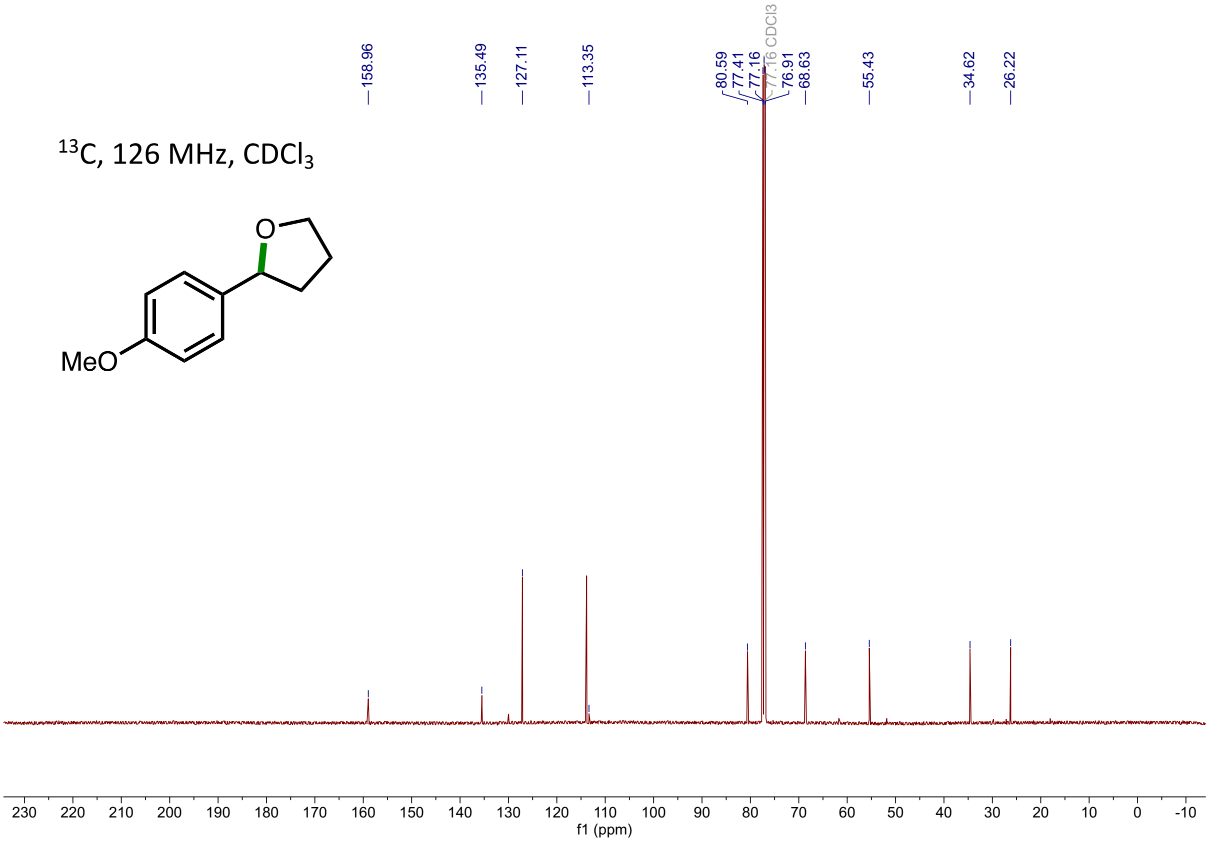

158.96
135.49
127.11
113.35
80.59
77.41
77.16
77.16 CDCl3
76.91
68.63
55.43
34.62
26.22
^{13}C, 126 MHz, CDCl$_3$
MeO
O
f1 (ppm)

(P9)

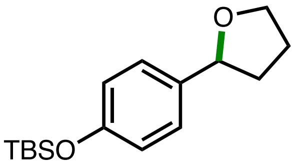

Prepared according to electrochemical general procedure using **S9** (101.5 mg, 0.30 mmol, 1 equiv.), *n*-Bu_4ClO_4 (103 mg, 0.30 mmol, 1 equiv.), dichloromethane (5.7 mL) and TFE (0.3 mL). The yield was determined by crude ^{1}H NMR using 1,3,5-trimethylbenzene (42 µL, 1 equiv.) as an internal standard: 70%. Purification by flash column chromatography (eluent = 5 to 15% EtOAc in hexanes, silica gel) to afford product as yellow oil (49.3 mg, 59% yield).

R_f = 0.75 (eluent = 10% EtOAc in hexanes); **ν_{max} / cm^{-1}** (thin film) 3726, 3221, 2967, 2361, 2342, 1246, 1061, 914, 827; **^{1}H NMR (500 MHz, $CDCl_3$)** δ 7.20 – 7.18 (m, 2H), 6.80 – 6.78 (m, 2H), 4.81– 4.79 (t, *J* = 6.3 Hz, 1H), 4.10 – 4.05 (m, 1H), 3.93 – 3.87 (m,1H), 2.28 – 2.25 (m, 1H), 2.03 – 1.98 (m, 2H), 1.83 – 1.75 (m, 1H), 0.98 (s, 9H), 0.18 (s, 6H); **^{13}C NMR (126 MHz, CDCl3)** δ 154.9, 136.0, 127.0, 120.0, 80.7, 68.6, 34.5, 26.2, 25.8, 18.3, -4.2; **HRMS (ESP$^+$)** ***m/z*** [M - OH]$^+$ Calcd for $C_{16}H_{25}OSi$ 261.1675; found 261.1676.

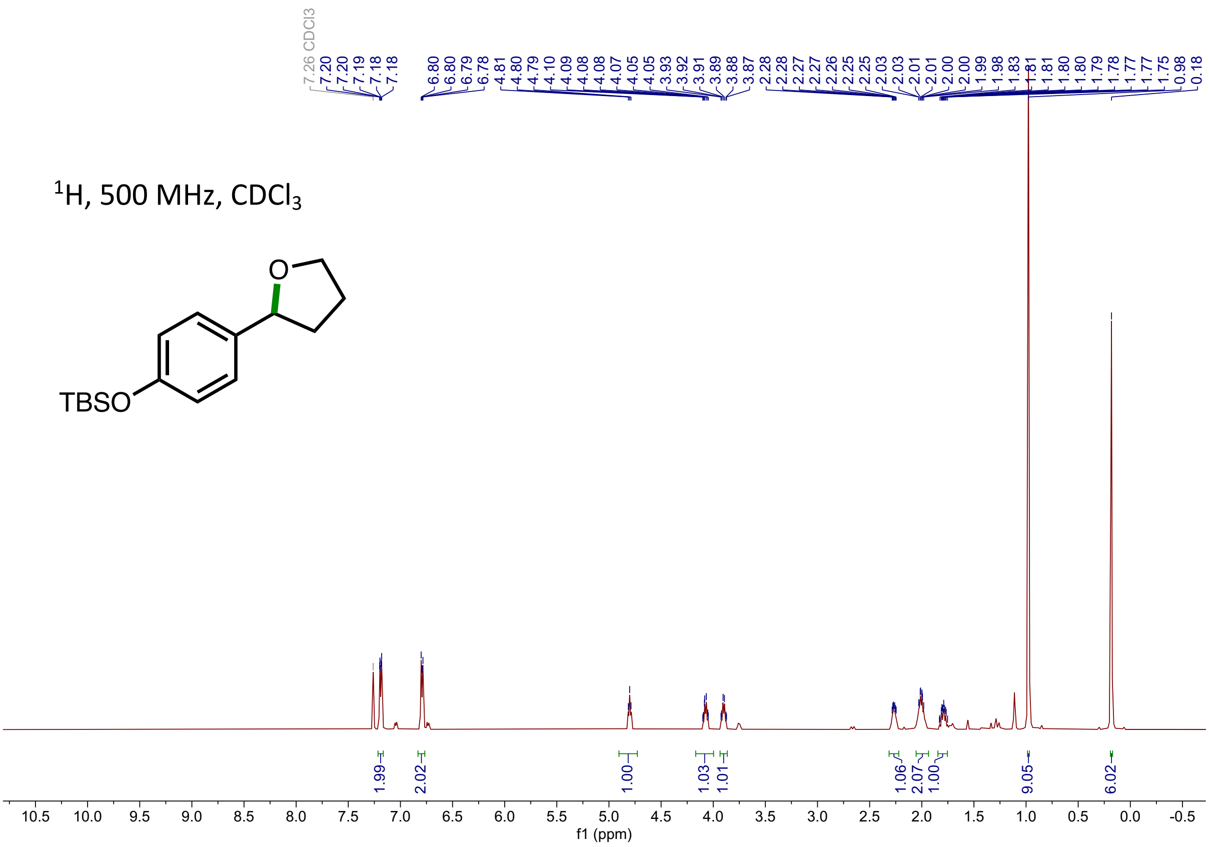

1H, 500 MHz, CDCl3
TBSO
O
7.26 CDCl3
7.20
7.20
7.19
7.18
7.18
6.80
6.80
6.79
6.78
4.81
4.80
4.79
4.10
4.09
4.08
4.08
4.07
4.05
4.05
3.93
3.92
3.91
3.89
3.88
3.87
2.28
2.28
2.27
2.27
2.26
2.25
2.25
2.03
2.03
2.01
2.01
2.00
2.00
1.99
1.98
1.83
1.81
1.81
1.80
1.80
1.79
1.78
1.77
1.75
0.98
0.18
1.99
2.02
1.00
1.03
1.01
1.06
2.07
1.00
9.05
6.02
10.5
10.0
9.5
9.0
8.5
8.0
7.5
7.0
6.5
6.0
5.5
5.0
4.5
4.0
3.5
3.0
2.5
2.0
1.5
1.0
0.5
0.0
-0.5
f1 (ppm)

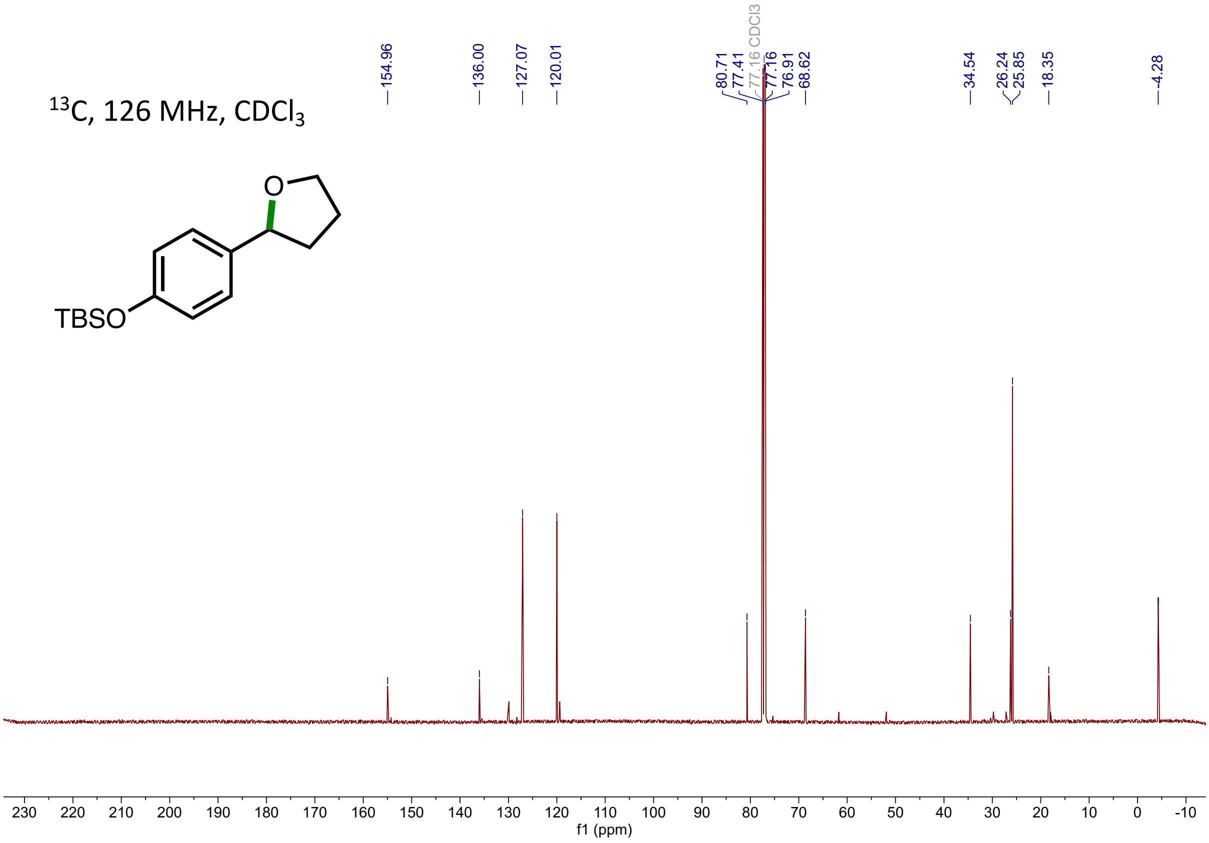

13C, 126 MHz, CDCl3
TBSO
O
154.96
136.00
127.07
120.01
80.71
77.41
77.16 CDCl3
77.16
76.91
68.62
34.54
26.24
25.85
18.35
-4.28
230
220
210
200
190
180
170
160
150
140
130
120
110
100
90
80
70
60
50
40
30
20
10
0
-10
f1 (ppm)

(P10)

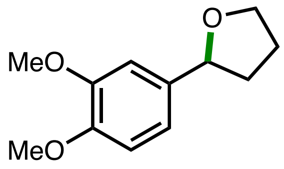

Prepared according to electrochemical general procedure using **S10** (80.0 mg, 0.30 mmol, 1 equiv.), *n*-Bu_4ClO_4 (103 mg, 0.30 mmol, 1 equiv.), dichloromethane (5.7 mL) and TFE (0.3 mL). The yield was determined by crude ^{1}H NMR using 1,3,5-trimethylbenzene (42 µL, 1 equiv.) as an internal standard: 79%. Purification by flash column chromatography (eluent = 5 to 15% EtOAc in hexanes, silica gel) to afford product as pale-yellow oil (46.0 mg, 75% yield).

R_f = 0.16 (eluent = 5% EtOAc in hexanes); **^{1}H NMR (500 MHz, $CDCl_3$)** δ 6.90 (d, *J* = 1.9 Hz, 1H), 6.88 – 6.84 (m, 2H), 4.82 (dd, *J* = 7.8, 6.6 Hz, 1H), 4.11 – 4.07 (m, 1H), 3.94 – 3.90 (m, 1H), 3.89 (s, 3H), 3.86 (s, 3H), 2.31 – 2.25 (m, 1H), 2.05 – 1.98 (m, 2H), 1.82 – 1.77 (ddt, *J* = 12.2, 8.6, 7.8 Hz, 1H); **^{13}C NMR (126 MHz, CDCl3)** δ 149.0, 148.2, 135.9, 118.0, 111.0, 109.0, 80.7, 68.6, 56.0, 55.9, 34.6, 26.1.

Data consistent with the literature.[13]

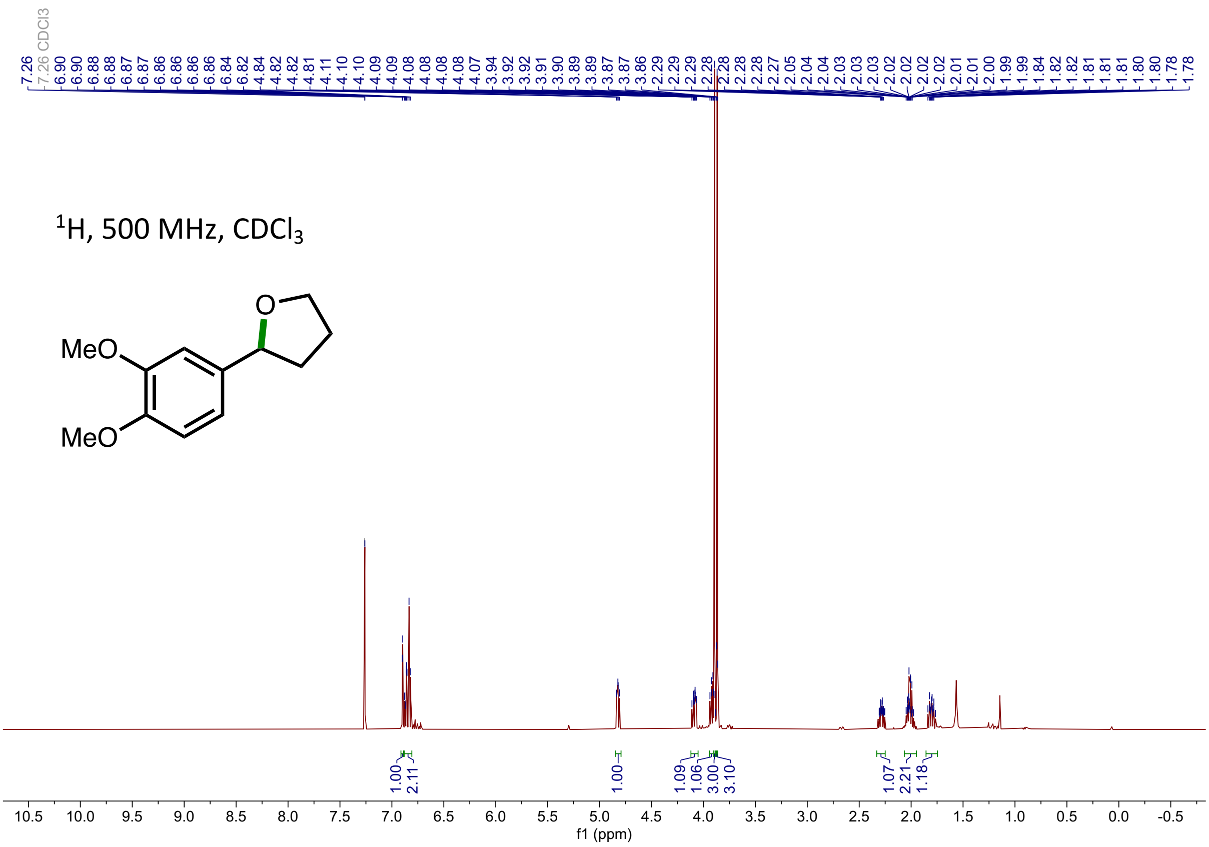
7.26
7.26 CDCl3
6.90
6.90
6.88
6.88
6.87
6.87
6.86
6.86
6.86
6.86
6.84
6.82
4.84
4.82
4.82
4.81
4.11
4.10
4.09
4.09
4.08
4.08
4.08
4.08
4.07
3.94
3.92
3.92
3.91
3.90
3.89
3.89
3.87
3.87
3.86
2.29
2.29
2.29
2.28
2.28
2.28
2.28
2.27
2.05
2.04
2.04
2.03
2.03
2.03
2.02
2.02
2.02
2.02
2.01
2.01
2.00
1.99
1.99
1.84
1.82
1.82
1.81
1.81
1.81
1.80
1.80
1.78
1.78
¹H, 500 MHz, CDCl3
MeO
MeO
O
1.00
2.11
1.00
1.09
1.06
3.00
3.10
1.07
2.21
1.18
10.5 10.0 9.5 9.0 8.5 8.0 7.5 7.0 6.5 6.0 5.5 5.0 4.5 4.0 3.5 3.0 2.5 2.0 1.5 1.0 0.5 0.0 -0.5
f1 (ppm)

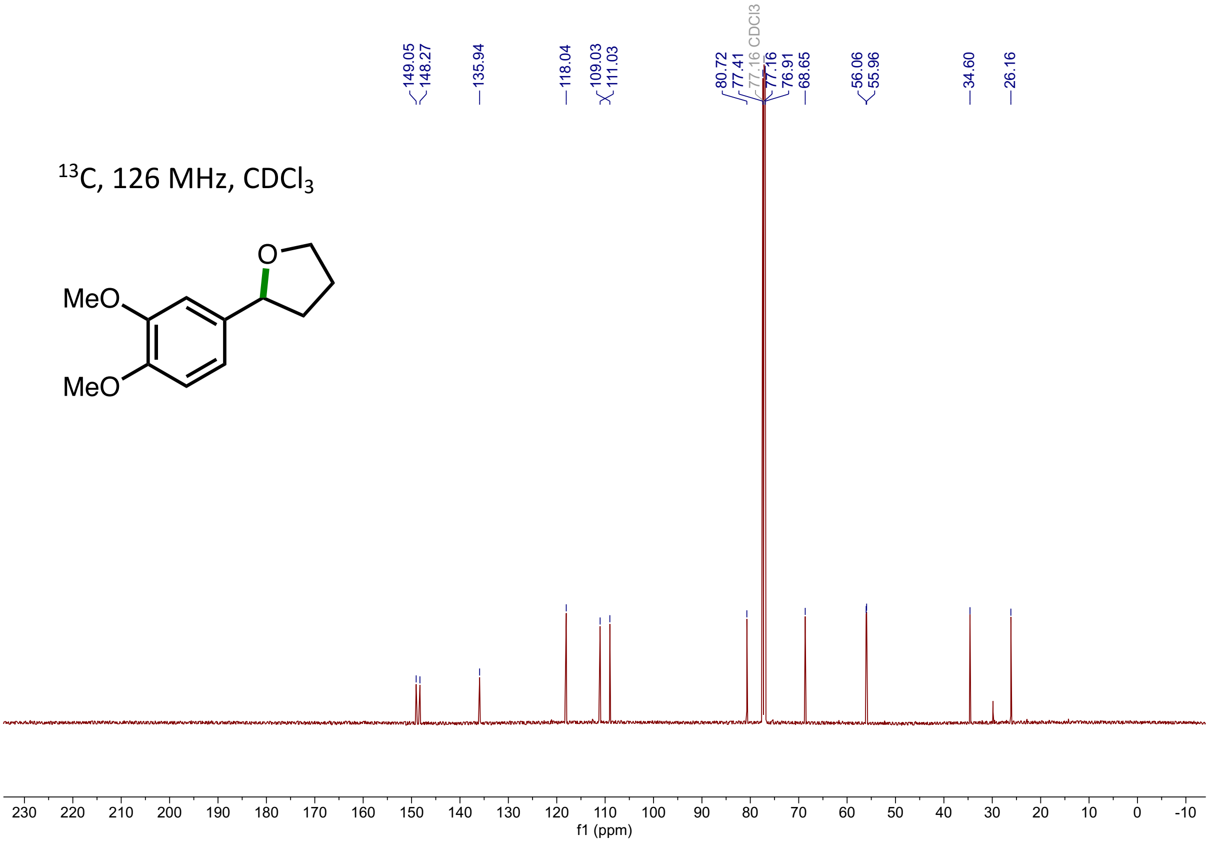
149.05
148.27
135.94
118.04
109.03
111.03
80.72
77.41
77.16 CDCl3
77.16
76.91
68.65
56.06
55.96
34.60
26.16
¹³C, 126 MHz, CDCl3
MeO
MeO
O
230 220 210 200 190 180 170 160 150 140 130 120 110 100 90 80 70 60 50 40 30 20 10 0 -10
f1 (ppm)

(P12)

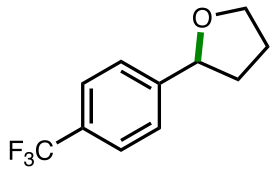

Prepared according to electrochemical general procedure using **S12** (82.9 mg, 0.30 mmol, 1 equiv.), *n*-Bu_4ClO_4 (103 mg, 0.30 mmol, 1 equiv.), dichloromethane (5.7 mL) and TFE (0.3 mL). The yield was determined by crude ^{1}H NMR using 1,3,5-trimethylbenzene (42 µL, 1 equiv.) as an internal standard: 34%. Purification by flash column chromatography (eluent = 5 to 15% EtOAc in hexanes, silica gel) to afford product as colourless oil (21.4 mg, 33% yield).

R_f = 0.57 (eluent = 10% EtOAc in hexanes); **^{1}H NMR (500 MHz, $CDCl_3$)** δ 7.59 – 7.57 (d, *J* = 8.1 Hz, 2H), 7.45 – 7.43 (d, *J* = 8.0 Hz, 2H), 4.96 – 4.93 (t, *J* = 7.2 Hz, 1H), 4.13 – 4.08 (m, 1H), 3.98 – 3.94 (m, 1H), 2.42 – 2.32 (m, 1H), 2.04 – 1.99 (m, 2H), 1.81 – 1.74 (m, 1H); **^{13}C NMR (126 MHz, CDCl3)** δ 147.8 (d, *J* = 1.4 Hz), 129.4 (q, *J* = 32.4 Hz), 125.9, 125.3 (q, *J* = 3.8 Hz), 80.1, 69.0, 34.8, 26.0; **^{19}F NMR (471 MHz, $CDCl_3$)** δ -62.40.

Data consistent with the literature.[14]

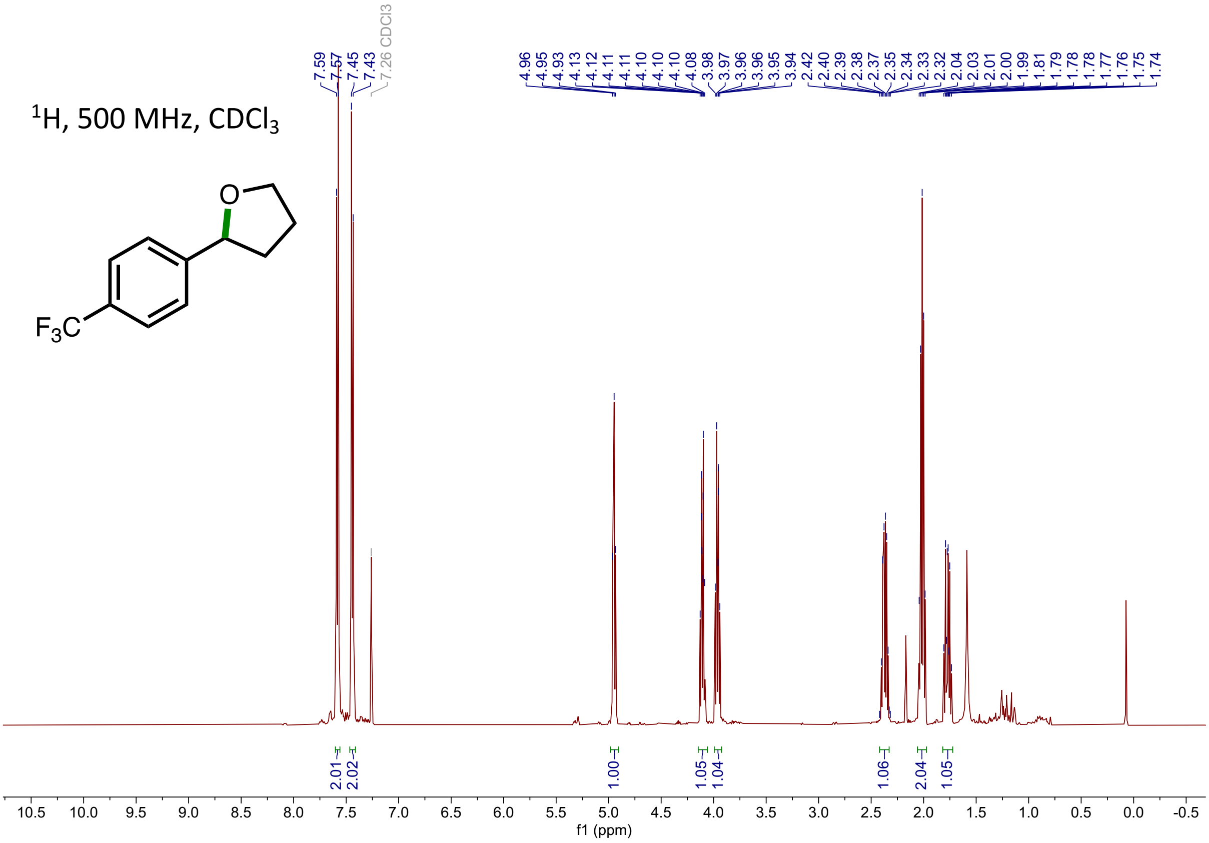

^{1}H, 500 MHz, CDCl$_3$
F$_3$C
O
7.59
7.57
7.45
7.43
7.26 CDCl3
4.96
4.95
4.93
4.13
4.12
4.11
4.11
4.10
4.10
4.10
4.08
3.98
3.97
3.96
3.96
3.95
3.94
2.42
2.40
2.39
2.38
2.37
2.35
2.34
2.33
2.32
2.04
2.03
2.01
2.00
1.99
1.81
1.79
1.78
1.78
1.77
1.76
1.75
1.74
2.01
2.02
1.00
1.05
1.04
1.06
2.04
1.05
10.5 10.0 9.5 9.0 8.5 8.0 7.5 7.0 6.5 6.0 5.5 5.0 4.5 4.0 3.5 3.0 2.5 2.0 1.5 1.0 0.5 0.0 -0.5
f1 (ppm)

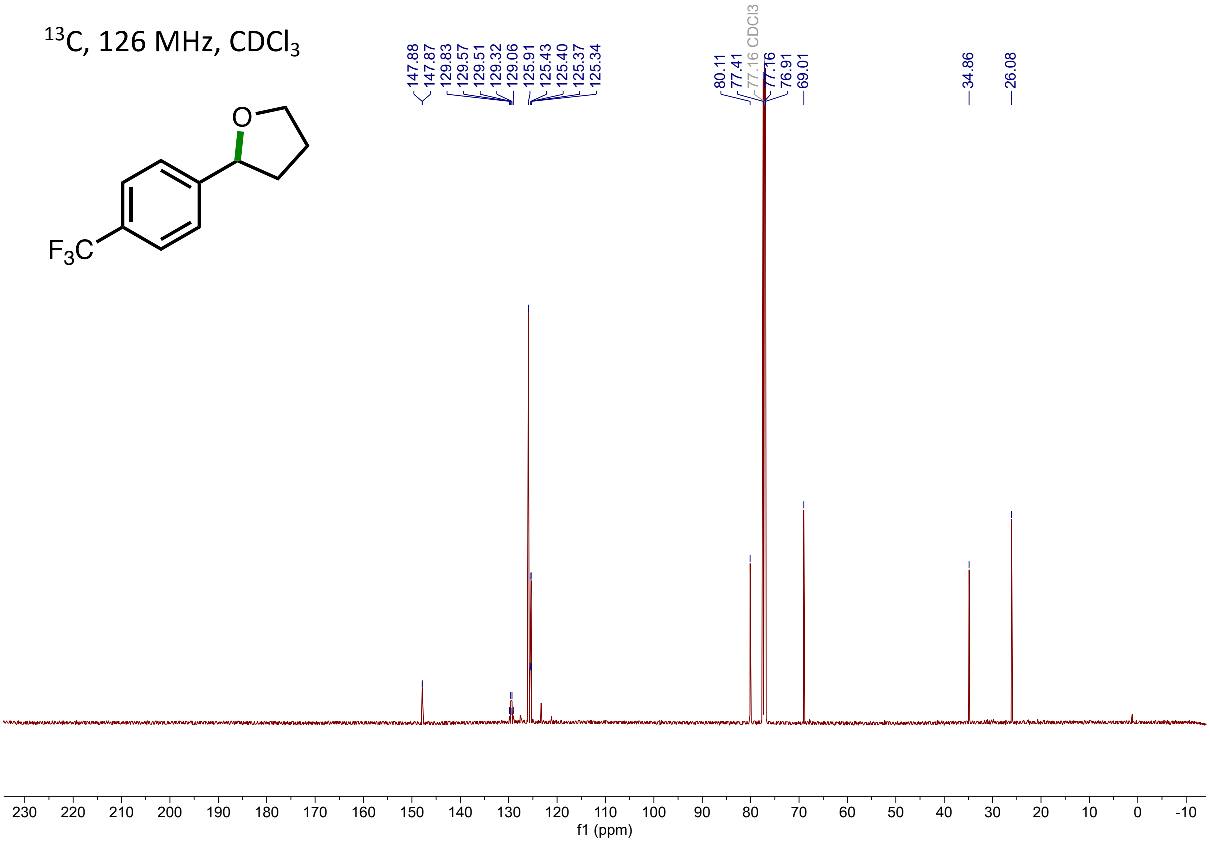

^{13}C, 126 MHz, CDCl$_3$
F$_3$C
O
147.88
147.87
129.83
129.57
129.51
129.32
129.06
125.91
125.43
125.40
125.37
125.34
80.11
77.41
77.16 CDCl3
77.16
76.91
69.01
34.86
26.08
230 220 210 200 190 180 170 160 150 140 130 120 110 100 90 80 70 60 50 40 30 20 10 0 -10
f1 (ppm)

^{19}F, 471 MHz, $CDCl_3$

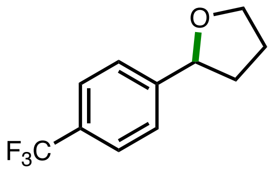

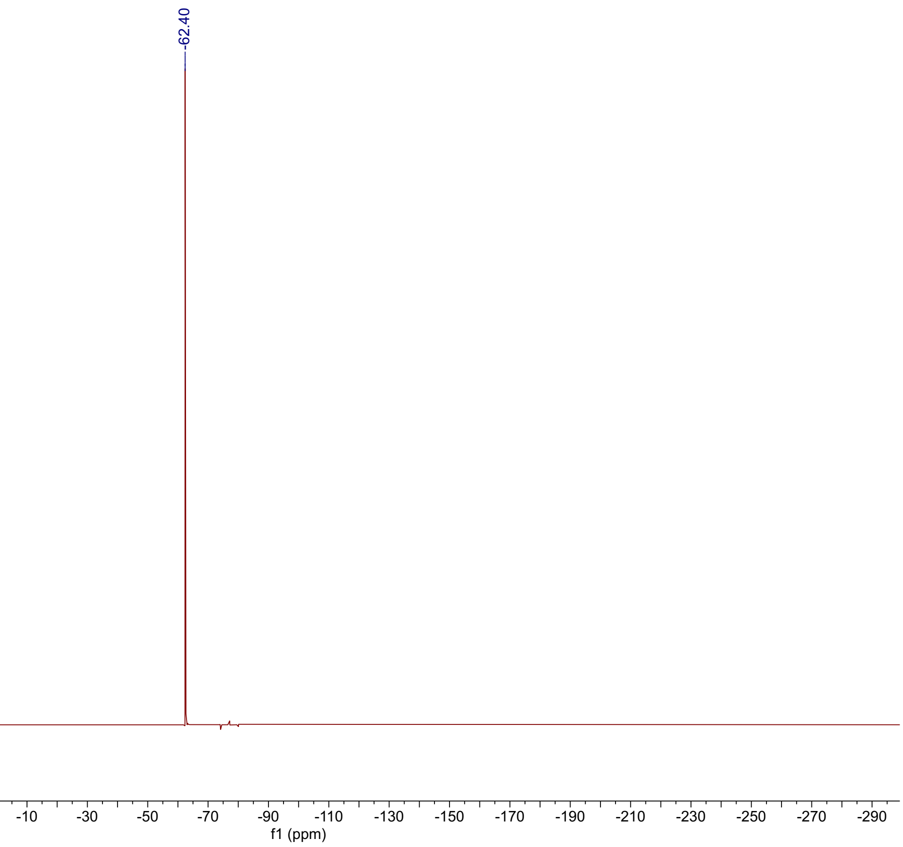

(P13)

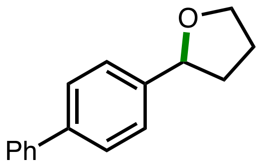

Prepared according to electrochemical general procedure using **S13** (85.32 mg, 0.30 mmol, 1 equiv.), *n*-Bu_4ClO_4 (103 mg, 0.30 mmol, 1 equiv.), dichloromethane (5.7 mL) and TFE (0.3 mL). The yield was determined by crude ^{1}H NMR using 1,3,5-trimethylbenzene (42 µL, 1 equiv.) as an internal standard: 62%. Purification by flash column chromatography (eluent = 5 to 15% EtOAc in hexanes, silica gel) to afford product as colourless oil (35.2mg, 52% yield).

R_f = 0.55 (eluent = 10% EtOAc in hexanes); **^{1}H NMR (500 MHz, $CDCl_3$)** δ 7.60 – 7.56 (m, 4H), 7.45 – 7.40 (m, 4H), 7.36 – 7.32 (m, 1H), 4.96 – 4.93 (t, *J* = 7.2 Hz, 1H), 4.15 – 4.11 (m, 1H), 3.99 – 3.94 (m, 1H), 2.40 – 2.33 (m, 1H), 2.08 – 2.00 (m, 2H), 1.90 – 1.82 (m, 1H) **^{13}C NMR (126 MHz, CDCl3)** δ 142.6, 141.1, 140.2, 128.8, 127.3, 127.2 (d, *J* = 1.4 Hz), 126.2, 80.6, 68.8, 34.7, 26.2.

Data consistent with the literature.[15]

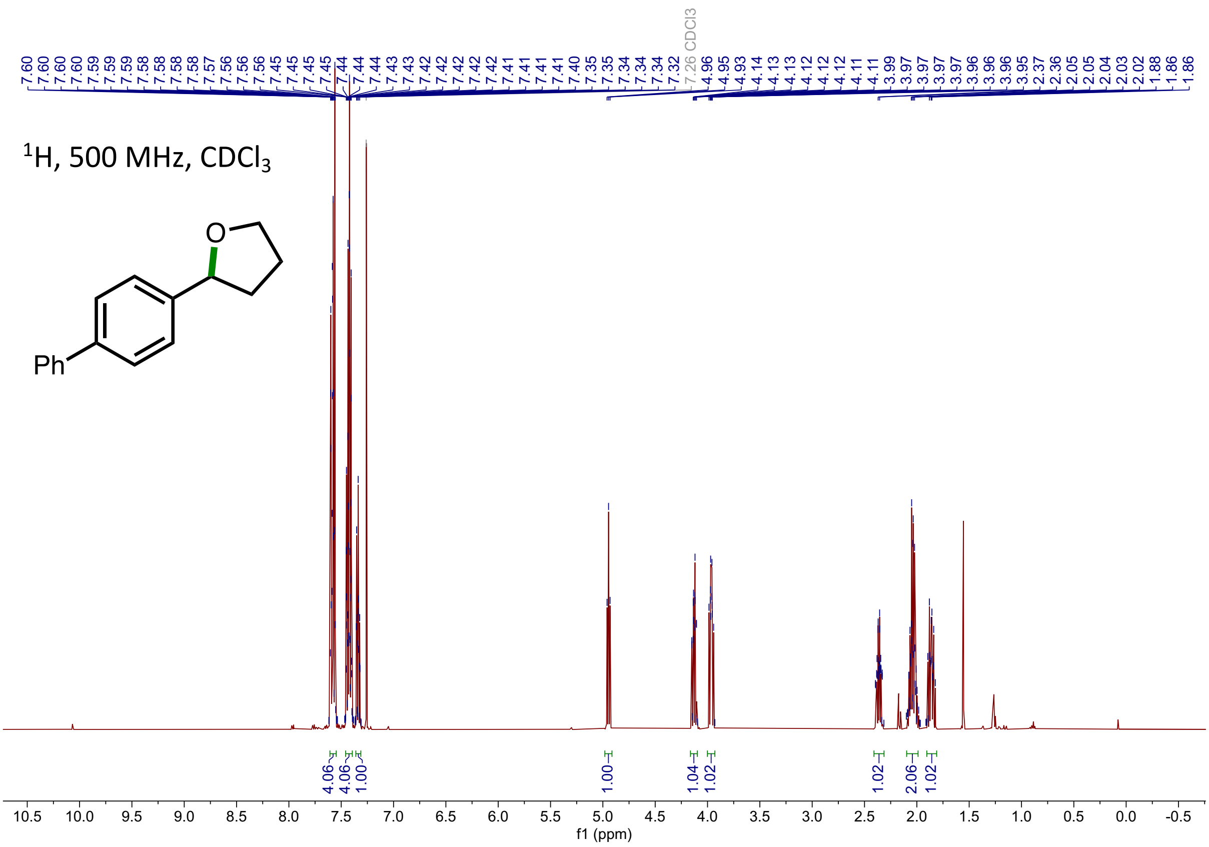
7.60 7.60 7.60 7.59 7.59 7.59 7.58 7.58 7.58 7.58 7.57 7.56 7.56 7.56 7.45 7.45 7.45 7.45 7.44 7.44 7.44 7.43 7.43 7.42 7.42 7.42 7.42 7.41 7.41 7.41 7.41 7.40 7.35 7.35 7.34 7.34 7.34 7.32 7.26 CDCl3 4.96 4.95 4.93 4.14 4.13 4.13 4.12 4.12 4.12 4.11 4.11 3.99 3.97 3.97 3.97 3.97 3.96 3.96 3.96 3.95 2.37 2.36 2.05 2.05 2.04 2.03 2.02 1.88 1.86 1.86
^{1}H, 500 MHz, CDCl$_3$
O
Ph
4.06
4.06
1.00
1.00
1.04
1.02
1.02
2.06
1.02
10.5 10.0 9.5 9.0 8.5 8.0 7.5 7.0 6.5 6.0 5.5 5.0 4.5 4.0 3.5 3.0 2.5 2.0 1.5 1.0 0.5 0.0 -0.5
f1 (ppm)

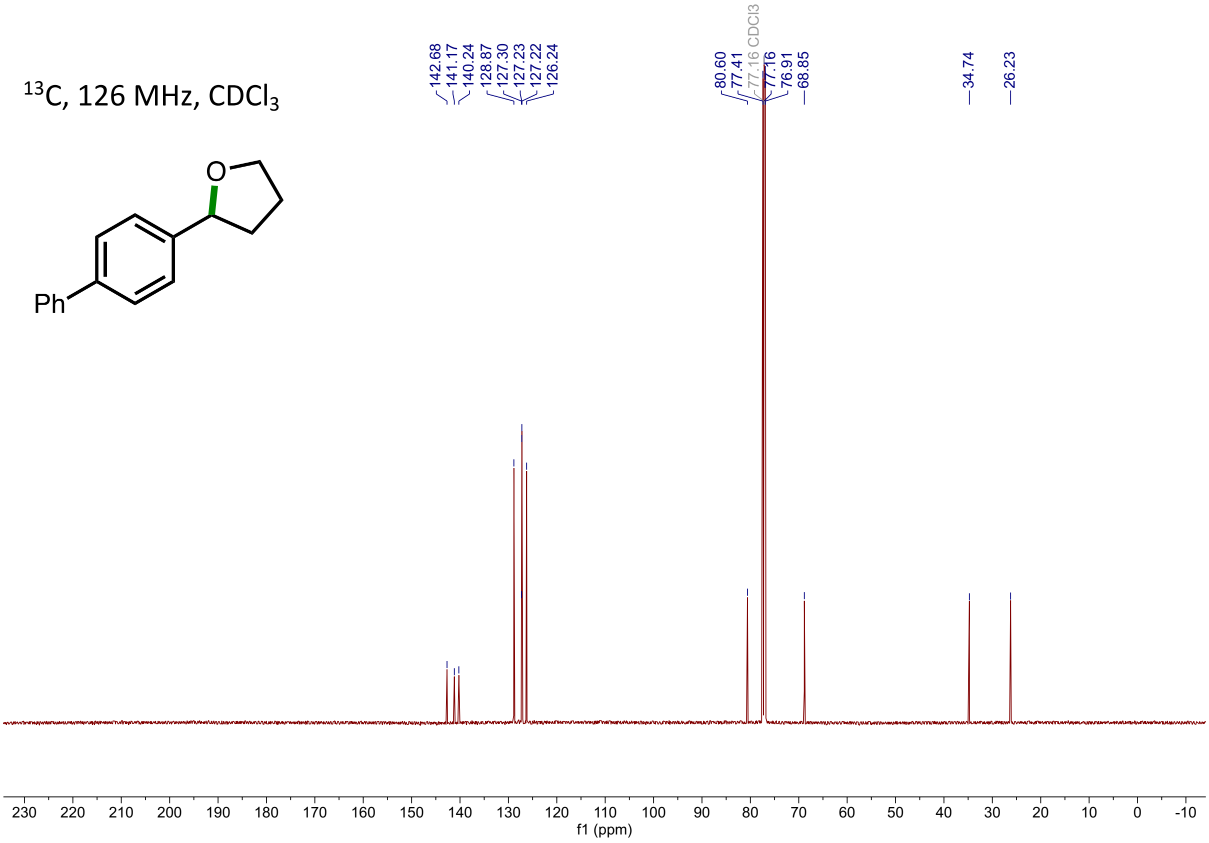
142.68 141.17 140.24 128.87 127.30 127.23 127.22 126.24
80.60 77.41 77.16 CDCl3 77.16 76.91 68.85
34.74
26.23
^{13}C, 126 MHz, CDCl$_3$
O
Ph
230 220 210 200 190 180 170 160 150 140 130 120 110 100 90 80 70 60 50 40 30 20 10 0 -10
f1 (ppm)

(P14)

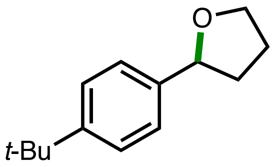

Prepared according to electrochemical general procedure using **S14** (79.3 mg, 0.30 mmol, 1 equiv.), *n*-Bu_4ClO_4 (103 mg, 0.30 mmol, 1 equiv.), dichloromethane (5.7 mL) and TFE (0.3 mL). The yield was determined by crude ^{1}H NMR using 1,3,5-trimethylbenzene (42 µL, 1 equiv.) as an internal standard: 90%. Purification by flash column chromatography (eluent = 5 to 15% EtOAc in hexanes, silica gel) to afford product as colourless oil (52.9 mg, 86% yield).

R_f = 0.61 (eluent = 10% EtOAc in hexanes); **$ν_{max}$ / cm^{-1}** (thin film) 2965, 2868, 2363, 2344, 1458, 1362, 1063, 828; **^{1}H NMR (500 MHz, $CDCl_3$)** δ 7.28 – 7.26 (m, 2H), 7.19 – 7.17 (m, 2H), 4.79 – 4.76 (t, *J* = 7.2 Hz, 1H), 4.02 – 3.97 (m, 1H), 3.86 – 3.81 (m, 1H), 2.22 – 2.21 (m, 1H), 1.94 – 1.90 (m, 2H), 1.78 – 1.73 (m, 1H), 1.23 (s, 9H); **^{13}C NMR (126 MHz, CDCl3)** δ 150.1, 140.4, 125.5, 125.3, 80.6, 68.7, 34.6, 34.4, 31.5, 26.2; **HRMS (EI$^+$)** ***m/z*** [M - H]$^+$ Calcd for $C_{14}H_{19}O$ 203.1430; found 203.1427.

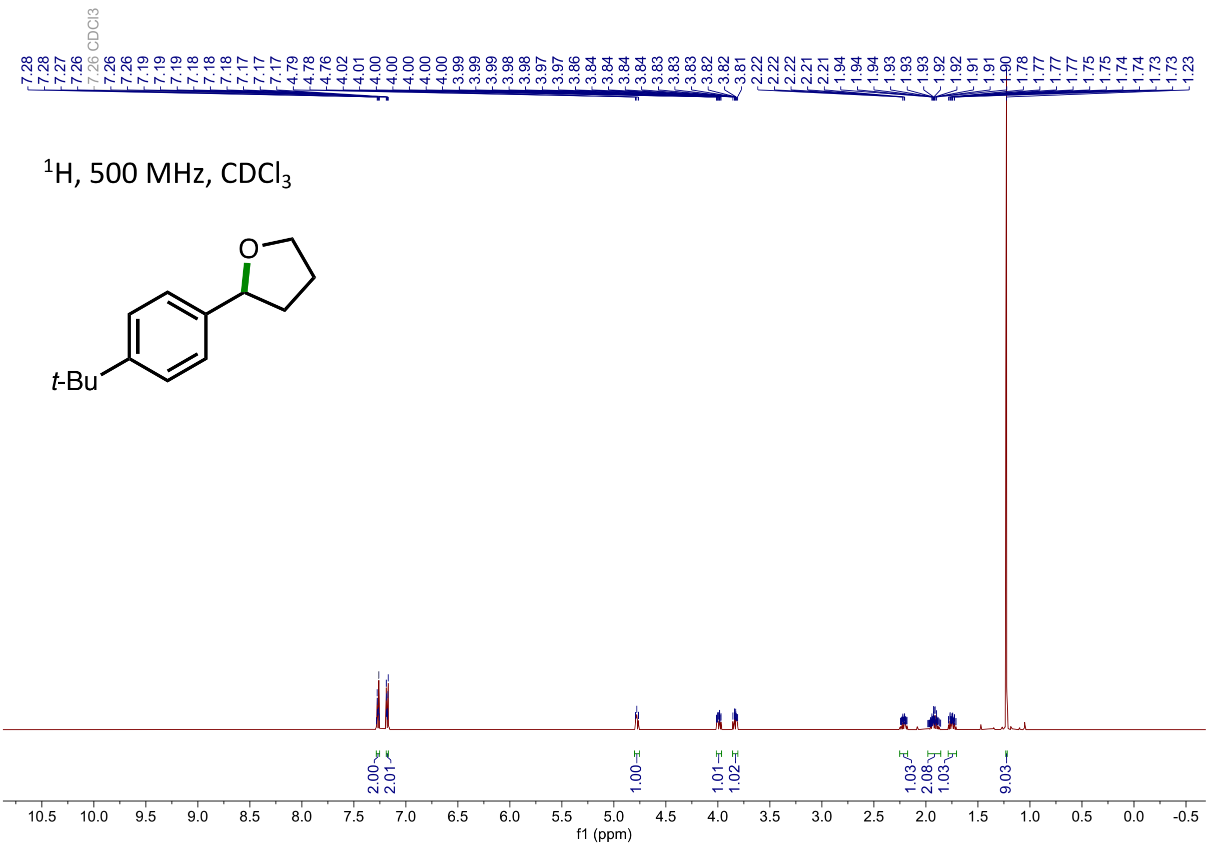

^{1}H, 500 MHz, CDCl$_3$
t-Bu
O
f1 (ppm)

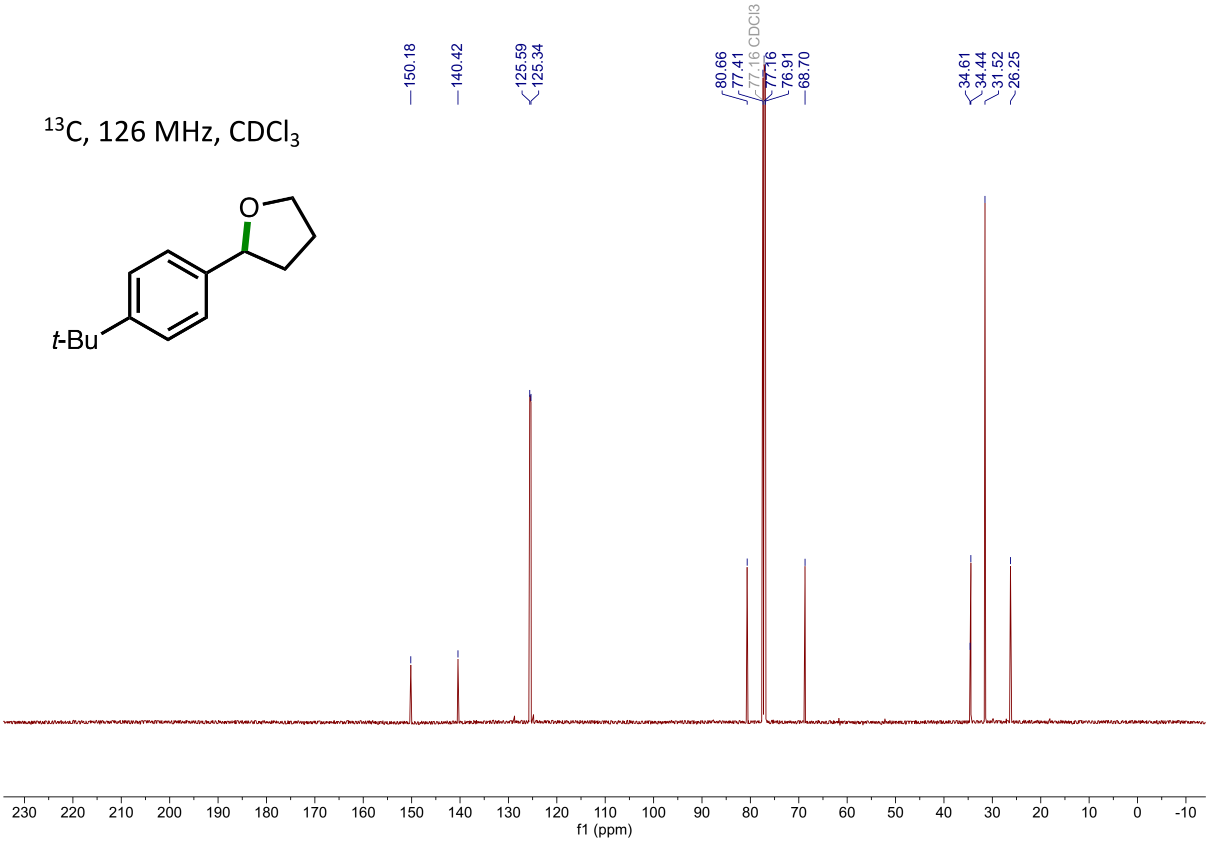

^{13}C, 126 MHz, CDCl$_3$
150.18
140.42
125.59
125.34
80.66
77.41
77.16 CDCl3
76.91
68.70
34.61
34.44
31.52
26.25
t-Bu
O
f1 (ppm)

(P15)

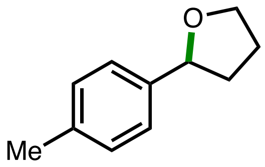

Prepared according to electrochemical general procedure using **S15** (66.7 mg, 0.30 mmol, 1 equiv.), *n*-Bu_4ClO_4 (103 mg, 0.30 mmol, 1 equiv.), dichloromethane (5.7 mL) and TFE (0.3 mL). The yield was determined by crude ^{1}H NMR using 1,3,5-trimethylbenzene (42 µL, 1 equiv.) as an internal standard: 88%. Purification by flash column chromatography (eluent = 5 to 15% EtOAc in hexanes, silica gel) to afford product as yellow oil (39.2 mg, 80% yield).

R_f = 0.60 (eluent = 10% EtOAc in hexanes); **^{1}H NMR (500 MHz, $CDCl_3$)** 7.26 – 7.22 (m, 2H), 7.15 – 7.13 (m, 2H), 4.87 – 4.85 (t, *J* = 7.2 Hz, 1H), 4.11 – 4.07 (dddd, *J* = 8.3, 7.1, 6.4, 0.5 Hz, 1H), 3.95 – 3.90 (m, 1H), 2.38 (s, 3H), 2.34 – 2.29 (m, 1H), 2.03 – 1.98 (m, 2H), 1.82 – 1.78 (m, 1H); **^{13}C NMR (126 MHz, CDCl3)** δ 140.5, 136.8, 129.1, 125.7, 80.7, 68.7, 34.7, 26.1, 21.2.

Data consistent with the literature.[15]

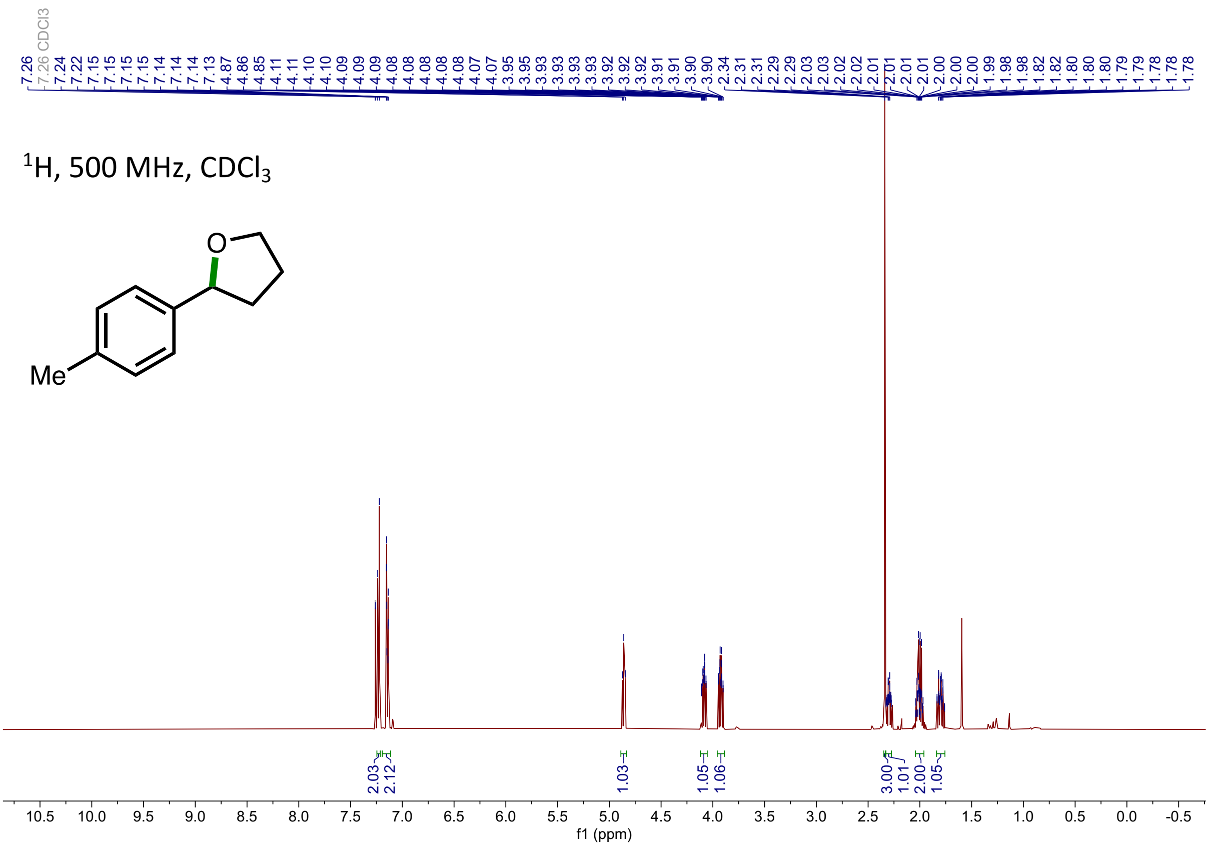
7.26
7.26 CDCl3
7.24
7.22
7.15
7.15
7.15
7.14
7.14
7.13
4.87
4.86
4.85
4.11
4.11
4.10
4.10
4.09
4.09
4.09
4.08
4.08
4.08
4.08
4.07
4.07
3.95
3.95
3.93
3.93
3.93
3.93
3.92
3.92
3.92
3.91
3.91
3.90
3.90
2.34
2.31
2.31
2.29
2.29
2.03
2.03
2.02
2.02
2.01
2.01
2.01
2.01
2.00
2.00
2.00
1.99
1.98
1.98
1.82
1.82
1.80
1.80
1.80
1.79
1.78
1.78
1.78
^{1}H, 500 MHz, $CDCl_3$
O
Me
2.03
2.12
1.03
1.05
1.06
3.00
1.01
2.00
1.05
10.5 10.0 9.5 9.0 8.5 8.0 7.5 7.0 6.5 6.0 5.5 5.0 4.5 4.0 3.5 3.0 2.5 2.0 1.5 1.0 0.5 0.0 -0.5
f1 (ppm)

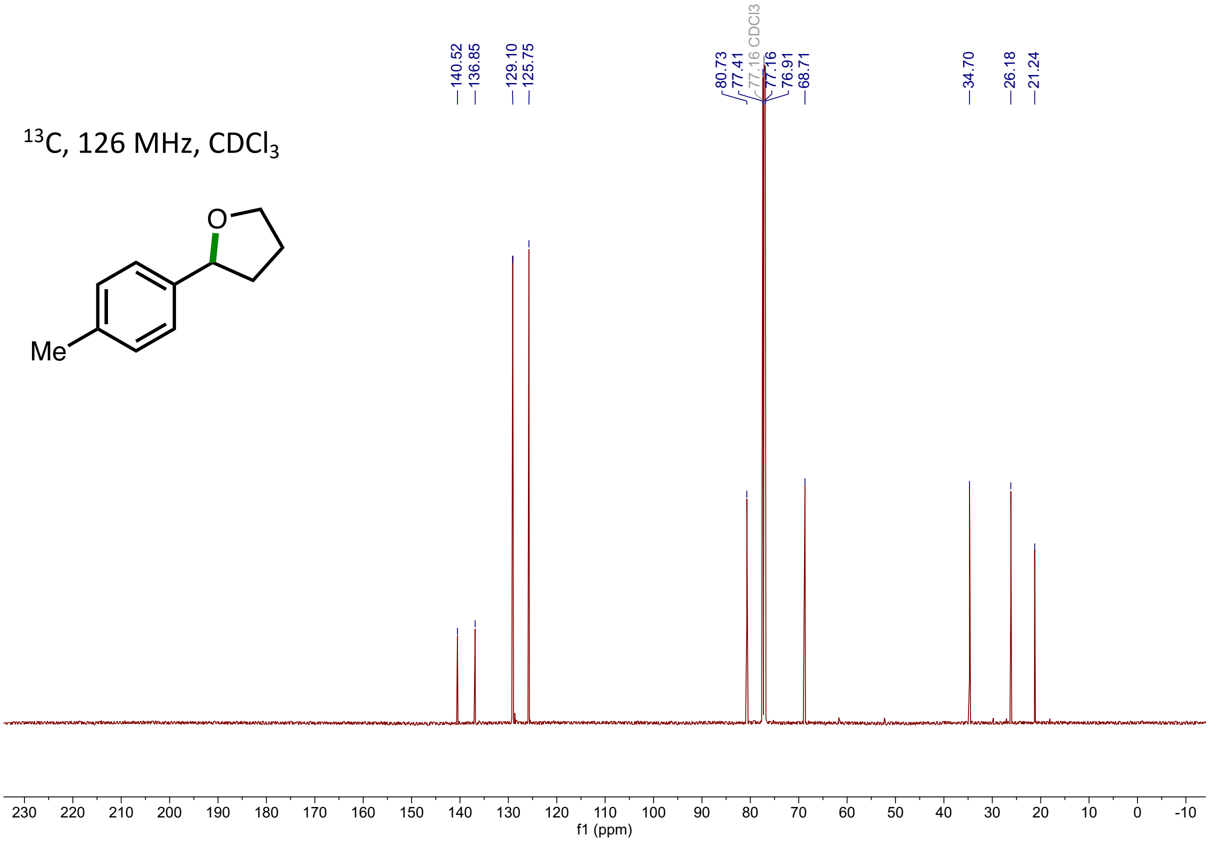
140.52
136.85
129.10
125.75
80.73
77.41
77.16 CDCl3
77.16
76.91
68.71
34.70
26.18
21.24
^{13}C, 126 MHz, $CDCl_3$
O
Me
230 220 210 200 190 180 170 160 150 140 130 120 110 100 90 80 70 60 50 40 30 20 10 0 -10
f1 (ppm)

(P16)

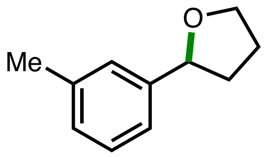

Prepared according to electrochemical general procedure using **S16** (66.7 mg, 0.30 mmol, 1 equiv.), *n*-Bu_4ClO_4 (103 mg, 0.30 mmol, 1 equiv.), dichloromethane (5.7 mL) and TFE (0.3 mL). The yield was determined by crude ^{1}H NMR using 1,3,5-trimethylbenzene (42 µL, 1 equiv.) as an internal standard: 75%. Purification by flash column chromatography (eluent = 5 to 15% EtOAc in hexanes, silica gel) to afford product as colourless oil (35.2 mg, 72% yield).

R_f = 0.61 (eluent = 10% EtOAc in hexanes); **^{1}H NMR (500 MHz, $CDCl_3$)** 7.24 – 7.21 (m, 1H), 7.17 – 7.12 (m, 2H), 7.08 – 7.06 (ddd, *J* = 7.4, 1.2, 0.6 Hz, 1H), 4.87 – 4.84 (t, *J* = 7.2 Hz, 1H), 4.11 – 4.08 (m, 1H), 3.96 – 3.92 (m, 1H), 2.36 (s, 3H), 2.32 – 2.30 (m, 1H), 2.03 – 1.99 (m, 2H), 1.83 – 1.79 (m, 1H); **^{13}C NMR (126 MHz, CDCl3)** δ 143.5, 138.0, 128.3, 128.0, 126.4, 122.8, 80.85, 68.7, 34.6, 26.1, 21.6.

Data consistent with the literature.[15]

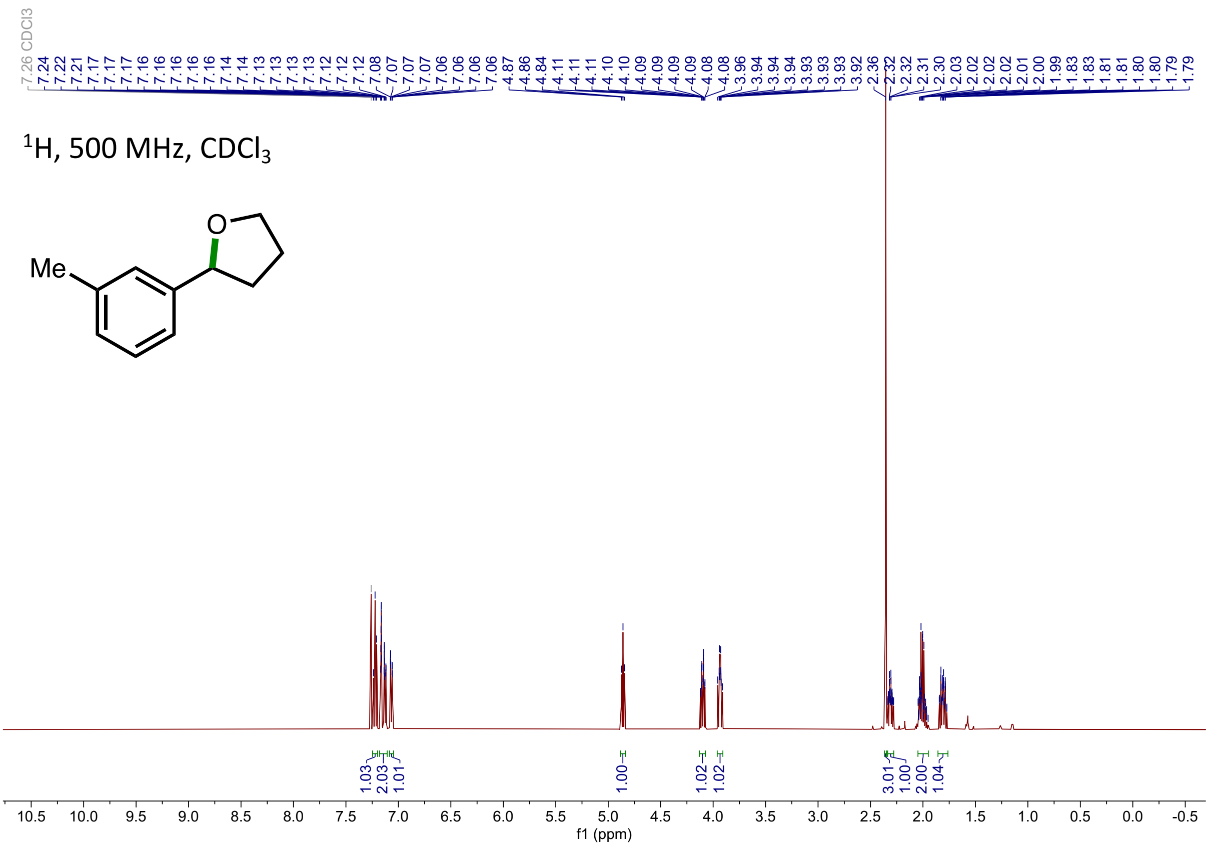
7.26 CDCl3
7.24 7.22 7.21 7.17 7.17 7.17 7.16 7.16 7.16 7.16 7.14 7.14 7.13 7.13 7.13 7.13 7.12 7.12 7.12 7.08 7.07 7.07 7.06 7.06 7.06 7.06 4.87 4.86 4.84 4.11 4.11 4.11 4.10 4.10 4.09 4.09 4.09 4.09 4.09 4.08 4.08 3.96 3.94 3.94 3.94 3.93 3.93 3.93 3.92 2.36 2.32 2.32 2.31 2.30 2.03 2.02 2.02 2.02 2.01 2.00 1.99 1.83 1.83 1.81 1.81 1.80 1.80 1.79 1.79
^{1}H, 500 MHz, CDCl$_3$
Me
O
1.03 2.03 1.01 1.00 1.02 1.02 3.01 1.00 2.00 1.04
10.5 10.0 9.5 9.0 8.5 8.0 7.5 7.0 6.5 6.0 5.5 5.0 4.5 4.0 3.5 3.0 2.5 2.0 1.5 1.0 0.5 0.0 -0.5
f1 (ppm)

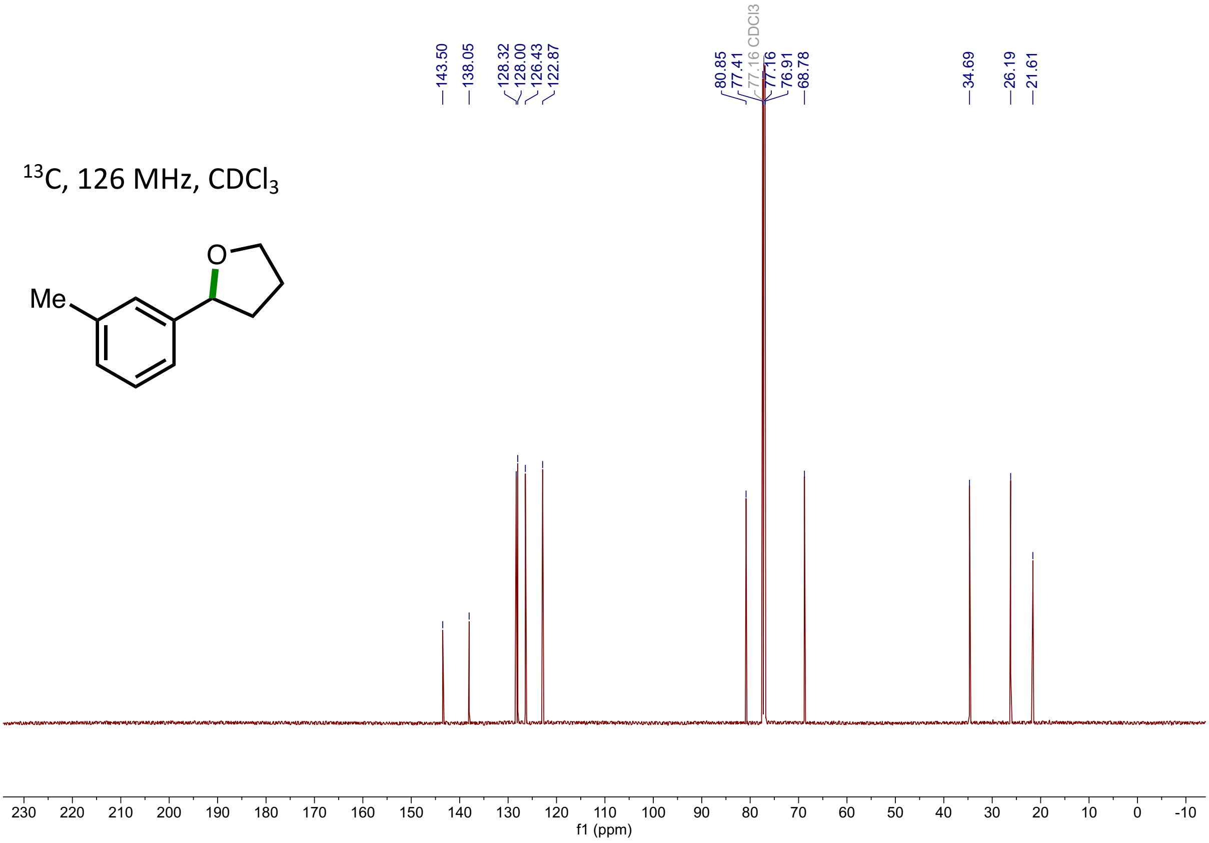
143.50 138.05 128.32 128.00 126.43 122.87 80.85 77.41 77.16 CDCl3 77.16 76.91 68.78 34.69 26.19 21.61
^{13}C, 126 MHz, CDCl$_3$
Me
O
230 220 210 200 190 180 170 160 150 140 130 120 110 100 90 80 70 60 50 40 30 20 10 0 -10
f1 (ppm)

(P17)

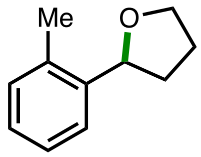

Prepared according to electrochemical general procedure using **S17** (66.7 mg, 0.30 mmol, 1 equiv.), *n*-Bu_4ClO_4 (103 mg, 0.30 mmol, 1 equiv.), dichloromethane (5.7 mL) and TFE (0.3 mL). The yield was determined by crude ^{1}H NMR using 1,3,5-trimethylbenzene (42 µL, 1 equiv.) as an internal standard: 83%. Purification by flash column chromatography (eluent = 5 to 15% EtOAc in hexanes, silica gel) to afford product as colourless oil (36.1 mg, 74% yield).

R_f = 0.53 (eluent = 10% EtOAc in hexanes); **^{1}H NMR (500 MHz, $CDCl_3$)** 7.45 – 7.43 (m, 1H), 7.20 – 7.11 (m, 3H), 5.08 – 5.05 (t, *J* = 7.2 Hz, 1H), 4.16 – 4.13 (m, 1H), 3.96 – 3.91 (m, 1H), 2.38 – 2.34 (m, 1H), 2.31 (s, 3H), 2.03 – 1.99 (m, 2H), 1.71 – 1.67 (m, 1H); **^{13}C NMR (126 MHz, CDCl3)** δ 141.9, 134.3, 130.2, 126.9, 126.1, 124.6, 78.1, 68.7, 33.3, 26.1, 19.3.

Data consistent with the literature.[15]

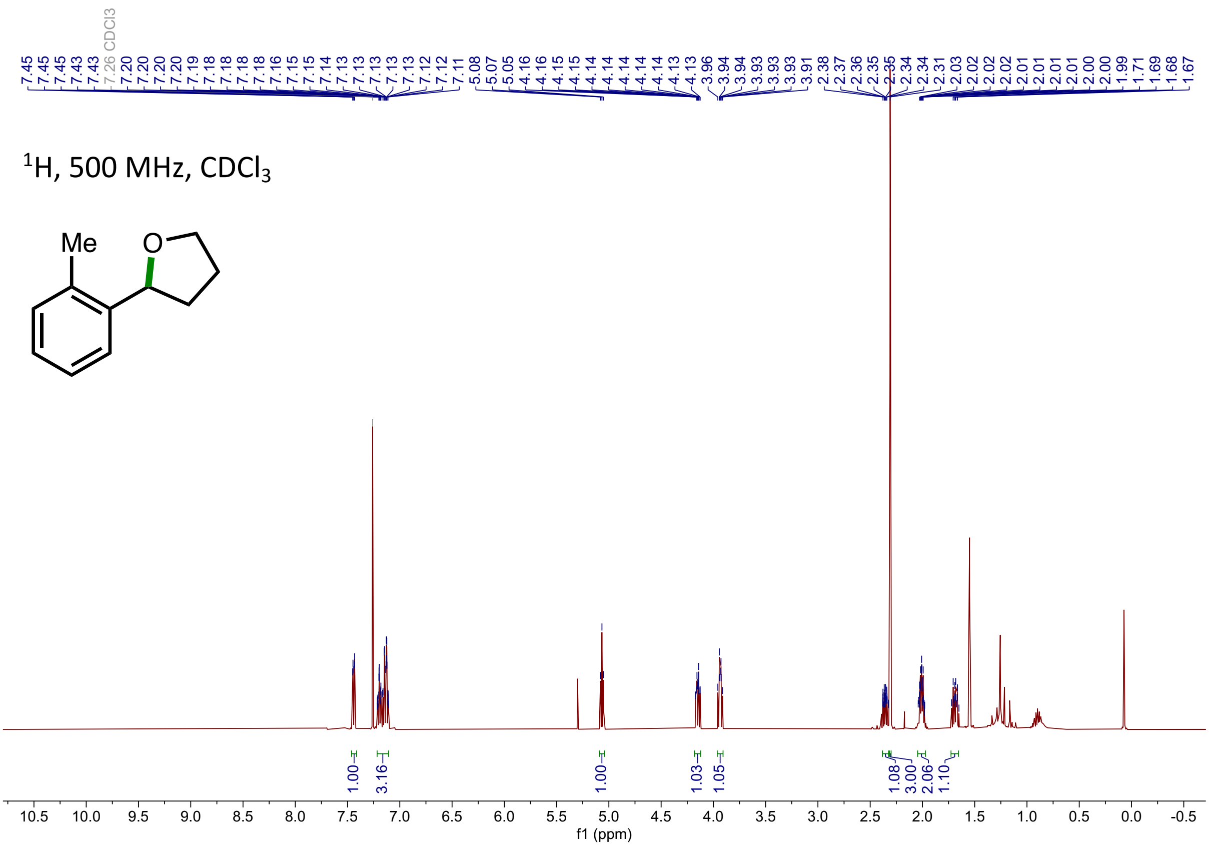
7.45
7.45
7.43
7.43
7.26 CDCl3
7.20
7.20
7.20
7.20
7.19
7.18
7.18
7.18
7.16
7.15
7.15
7.14
7.13
7.13
7.13
7.13
7.12
7.11
5.08
5.07
5.05
4.16
4.16
4.15
4.15
4.14
4.14
4.14
4.14
4.14
4.13
4.13
3.96
3.94
3.94
3.93
3.93
3.93
3.91
2.38
2.37
2.36
2.35
2.35
2.34
2.34
2.31
2.03
2.02
2.02
2.02
2.01
2.01
2.01
2.01
2.00
1.99
1.71
1.69
1.68
1.67
¹H, 500 MHz, CDCl3
Me
O
1.00
3.16
1.00
1.03
1.05
1.08
3.00
2.06
1.10
f1 (ppm)

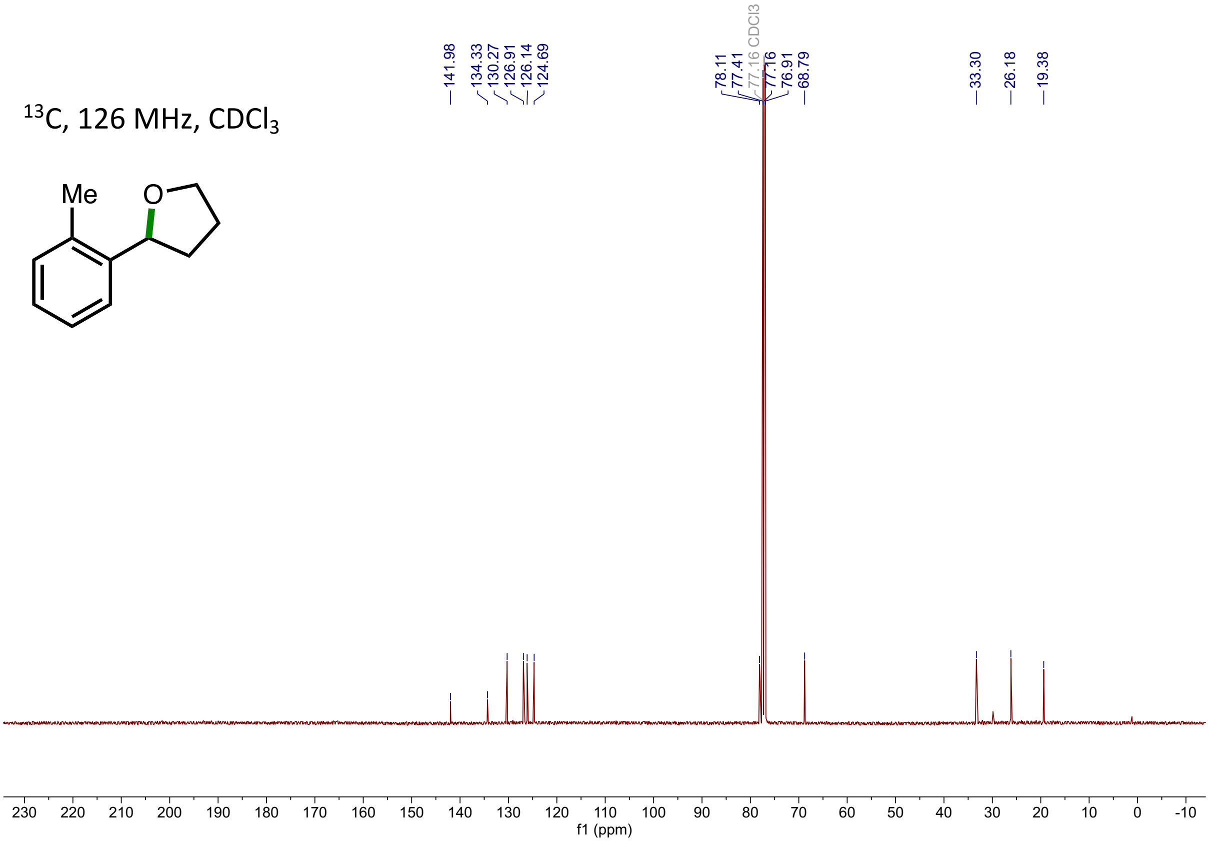
141.98
134.33
130.27
126.91
126.14
124.69
78.11
77.41
77.16 CDCl3
77.16
76.91
68.79
33.30
26.18
19.38
¹³C, 126 MHz, CDCl3
Me
O
f1 (ppm)

(P18)

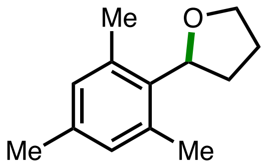

Prepared according to electrochemical general procedure using **S18** (75.1 mg, 0.30 mmol, 1 equiv.), *n*-Bu_4ClO_4 (103 mg, 0.30 mmol, 1 equiv.), dichloromethane (5.7 mL) and TFE (0.3 mL). The yield was determined by crude ^{1}H NMR using 1,3,5-trimethylbenzene (42 µL, 1 equiv.) as an internal standard: 62%. Purification by flash column chromatography (eluent = 5 to 15% EtOAc in hexanes, silica gel) to afford product as yellow oil (31.5 mg, 55% yield).

R_f = 0.63 (eluent = 10% EtOAc in hexanes); **^{1}H NMR (500 MHz, $CDCl_3$)** 6.81 (s, 2H), 5.16 – 5.13 (dd, *J* = 10.1, 6.6 Hz, 1H), 4.15 – 4.10 (m, 1H), 3.88 – 3.83 (m, 1H), 2.34 (s, 6H), 2.24 (s, 3H), 2.19 – 2.03 (m, 3H), 1.92 – 1.85 (m, 1H); **^{13}C NMR (126 MHz, CDCl3)** δ 136.7, 136.6, 134.5, 130.4, 78.9, 68.2, 31.4, 27.1, 21.1, 20.9.

Data consistent with the literature.[16]

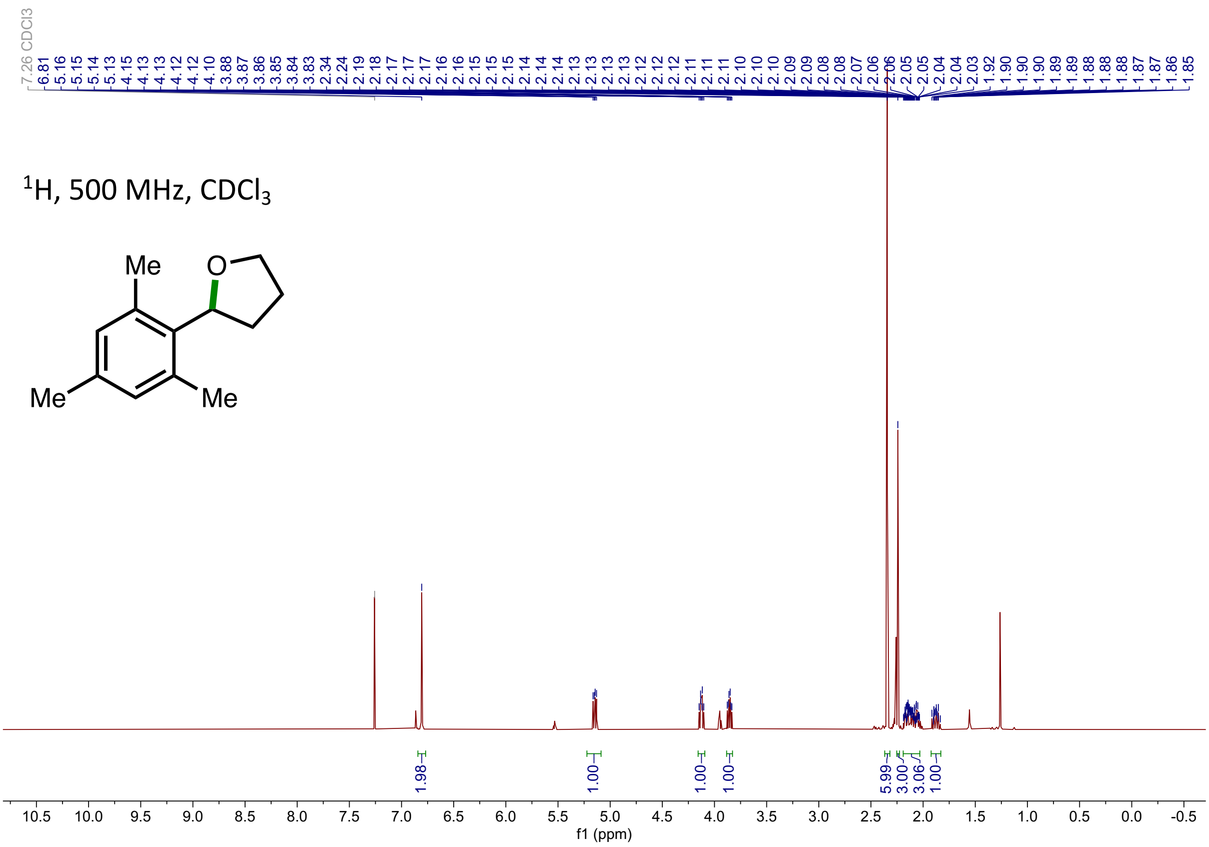
7.26 CDCl3
6.81
5.16
5.15
5.14
5.13
4.15
4.13
4.13
4.12
4.12
4.10
3.88
3.87
3.86
3.85
3.84
3.83
2.34
2.24
2.19
2.18
2.17
2.17
2.17
2.16
2.16
2.15
2.15
2.15
2.14
2.14
2.14
2.13
2.13
2.13
2.12
2.12
2.12
2.11
2.11
2.11
2.10
2.10
2.09
2.09
2.08
2.08
2.07
2.06
2.06
2.05
2.05
2.04
2.04
2.03
1.92
1.90
1.90
1.89
1.89
1.88
1.88
1.87
1.87
1.86
1.85
1H, 500 MHz, CDCl3
Me
O
Me
Me
1.98
1.00
1.00
1.00
5.99
3.00
3.06
1.00
10.5
10.0
9.5
9.0
8.5
8.0
7.5
7.0
6.5
6.0
5.5
5.0
4.5
4.0
3.5
3.0
2.5
2.0
1.5
1.0
0.5
0.0
-0.5
f1 (ppm)

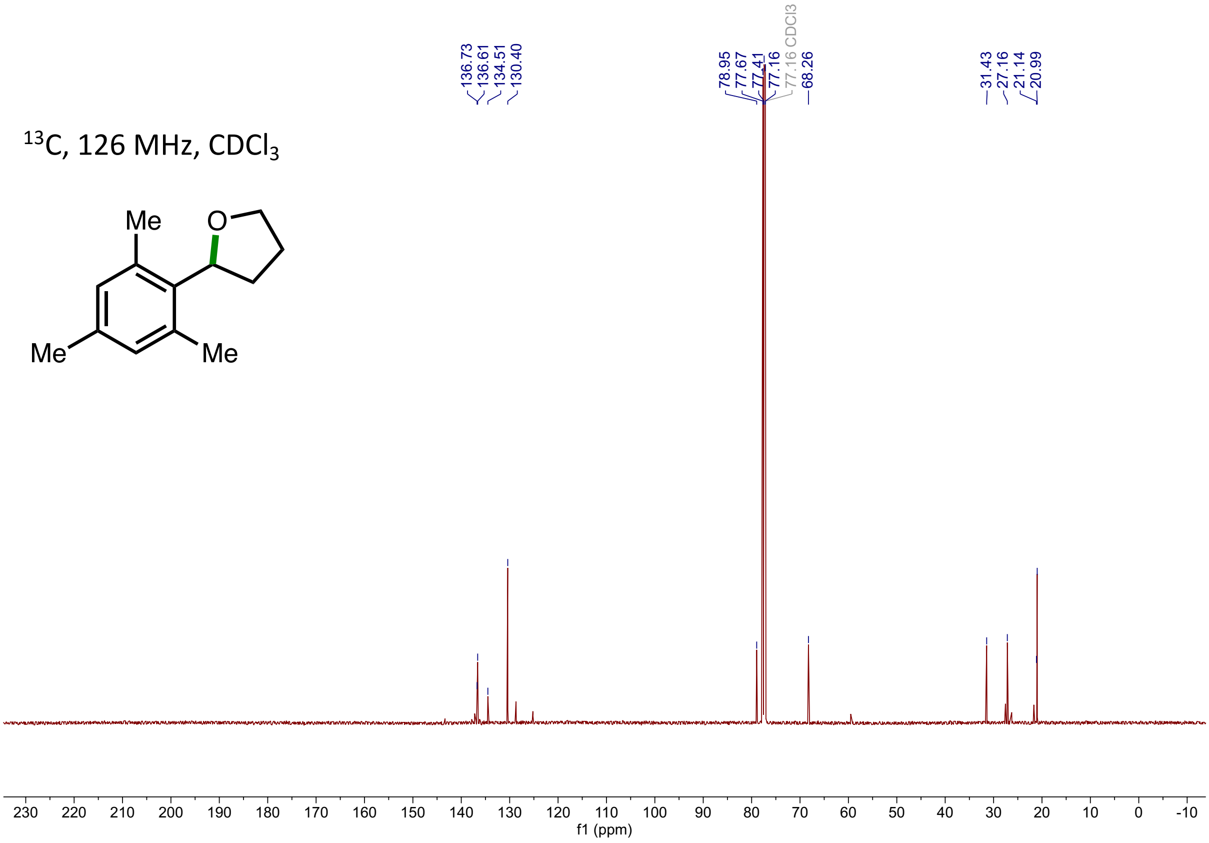
136.73
136.61
134.51
130.40
78.95
77.67
77.41
77.16
77.16 CDCl3
68.26
31.43
27.16
21.14
20.99
13C, 126 MHz, CDCl3
Me
O
Me
Me
230
220
210
200
190
180
170
160
150
140
130
120
110
100
90
80
70
60
50
40
30
20
10
0
-10
f1 (ppm)

(P19)

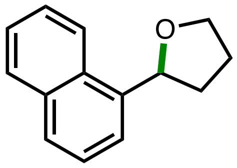

Prepared according to electrochemical general procedure using **S19** (77.50 mg, 0.30 mmol, 1 equiv.), *n*-Bu_4ClO_4 (103 mg, 0.30 mmol, 1 equiv.), dichloromethane (5.7 mL) and TFE (0.3 mL). The yield was determined by crude ^{1}H NMR using 1,3,5-trimethylbenzene (42 µL, 1 equiv.) as an internal standard: 86%. Purification by flash column chromatography (eluent = 5 to 15% EtOAc in hexanes, silica gel) to afford product as pale-yellow oil (47.0 mg, 83% yield).

R_f = 0.46 (eluent = 5% EtOAc in hexanes); **^{1}H NMR (500 MHz, $CDCl_3$)** 7.99 – 7.96 (m, 1H), 7.87 (dd, *J* = 7.4, 2.2 Hz, 1H), 7.75 (d, *J* = 9.5 Hz, 1H), 7.64 (dt, *J* = 7.2, 1.1 Hz, 1H), 7.53 – 7.45 (m, 3H), 5.65 (t, *J* = 7.0 Hz, 1H), 4.26 – 4.22 (m, 1H), 4.06 – 4.02 (m, 1H), 2.59 – 2.55 (m, 1H), 2.09 – 2.0 (m, 2H), 1.95 – 1.89 (m, 1H); **^{13}C NMR (126 MHz, CDCl3)** δ 139.3, 133.7, 130.3, 128.8, 127.4, 125.7, 125.5, 125.3, 123.4, 121.8, 77.9, 68.7, 33.8, 25.9.

Data consistent with the literature.[17]

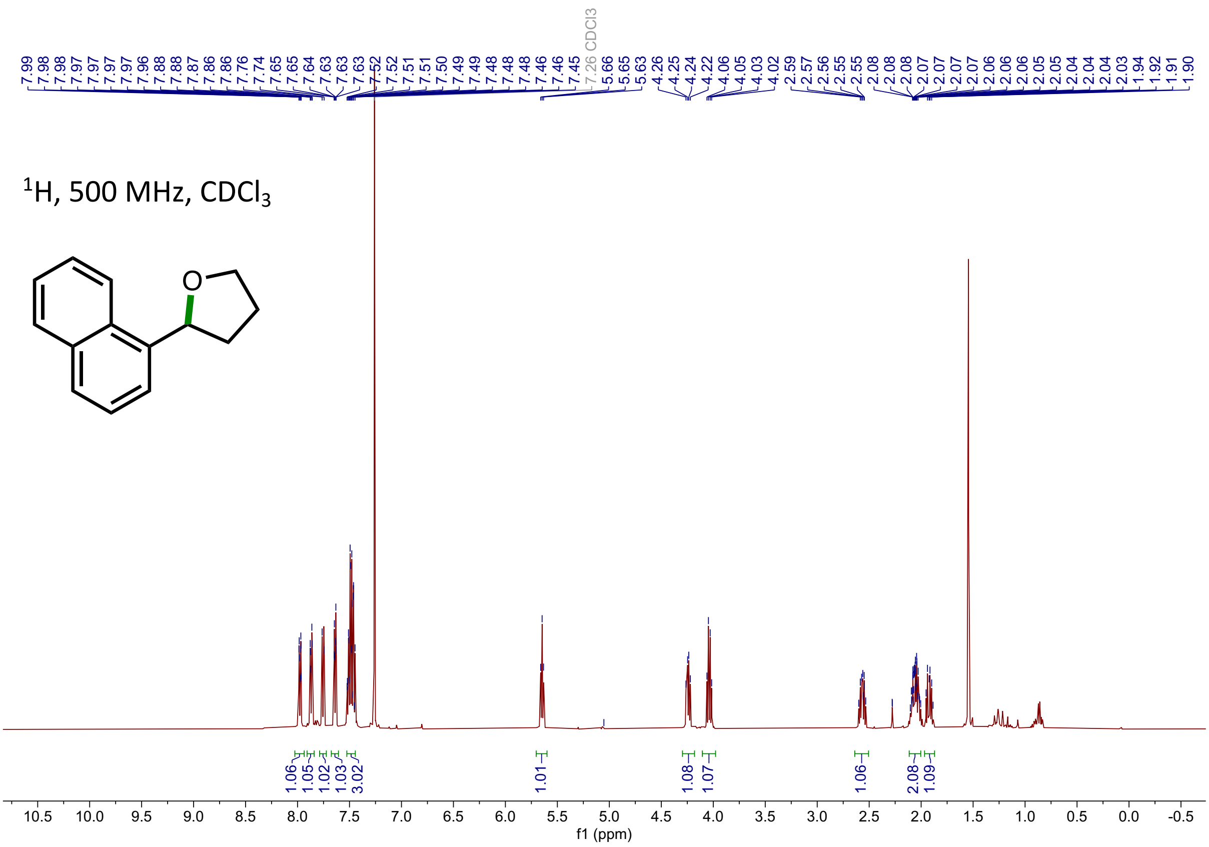

7.99 7.98 7.97 7.97 7.97 7.97 7.96 7.88 7.88 7.87 7.86 7.86 7.76 7.74 7.65 7.64 7.63 7.63 7.63 7.52 7.52 7.51 7.50 7.49 7.49 7.48 7.48 7.46 7.46 7.45
7.26 CDCl3
5.66 5.65 5.63 4.26 4.25 4.24 4.22 4.06 4.05 4.03 4.02 2.59 2.57 2.56 2.55 2.55 2.08 2.08 2.08 2.07 2.07 2.07 2.06 2.06 2.06 2.05 2.04 2.04 2.04 2.03 1.94 1.92 1.91 1.90
^{1}H, 500 MHz, CDCl$_3$
O
1.06 1.05 1.02 1.03 3.02 1.01 1.08 1.07 1.06 2.08 1.09
10.5 10.0 9.5 9.0 8.5 8.0 7.5 7.0 6.5 6.0 5.5 5.0 4.5 4.0 3.5 3.0 2.5 2.0 1.5 1.0 0.5 0.0 -0.5
f1 (ppm)

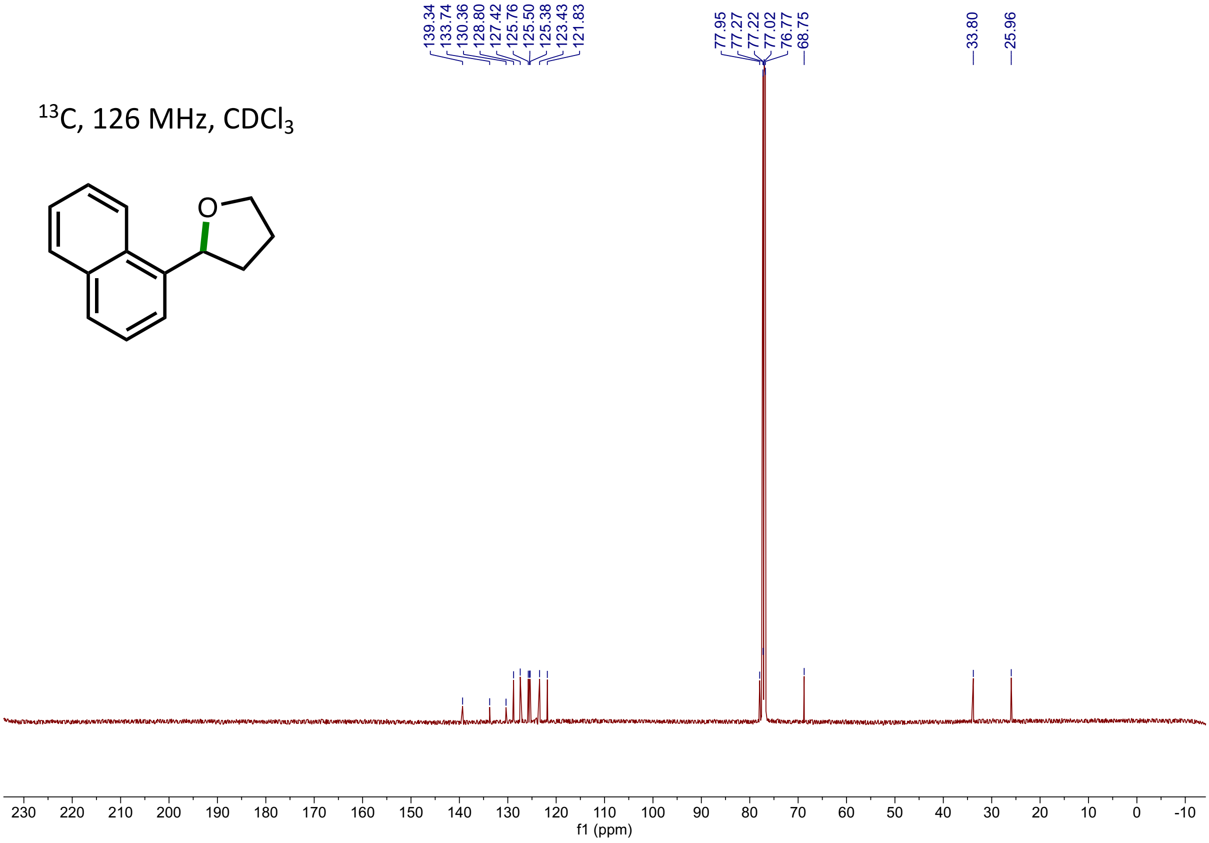

139.34 133.74 130.36 128.80 127.42 125.76 125.50 125.38 123.43 121.83
77.95 77.27 77.22 77.02 76.77 68.75
33.80 25.96
^{13}C, 126 MHz, CDCl$_3$
O
230 220 210 200 190 180 170 160 150 140 130 120 110 100 90 80 70 60 50 40 30 20 10 0 -10
f1 (ppm)

(P20)

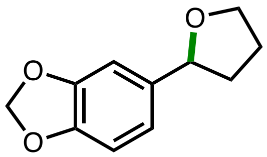

Prepared according to electrochemical general procedure using **S20** (75.7 mg, 0.30 mmol, 1 equiv.), *n*-Bu_4ClO_4 (103 mg, 0.30 mmol, 1 equiv.), dichloromethane (5.7 mL) and TFE (0.3 mL). The yield was determined by crude ^{1}H NMR using 1,3,5-trimethylbenzene (42 µL, 1 equiv.) as an internal standard: 38%. Purification by flash column chromatography (eluent = 5 to 15% EtOAc in hexanes, silica gel) to afford product as colourless oil (17.9 mg, 31% yield).

R_f = 0.55 (eluent = 10% EtOAc in hexanes); **^{1}H NMR (500 MHz, $CDCl_3$)** 6.84 (m, 1H), 6.81 – 6.78 (m, 2H), 5.93 (s, 1H), 4.81 – 4.78 (t, 1H), 4.11 – 4.02 (m, 1H), 3.95 – 3.87 (m, 1H), 2.30 – 2.23 (m, 1H), 2.04 – 1.93 (m, 2H), 1.80 – 1.74 (m, 1H); **^{13}C NMR (126 MHz, CDCl3)** δ 147.7, 146.7, 137.5, 119.1, 108.1, 106.4, 101.0, 80.7, 68.7, 34.7, 26.1.

Data consistent with the literature.[13]

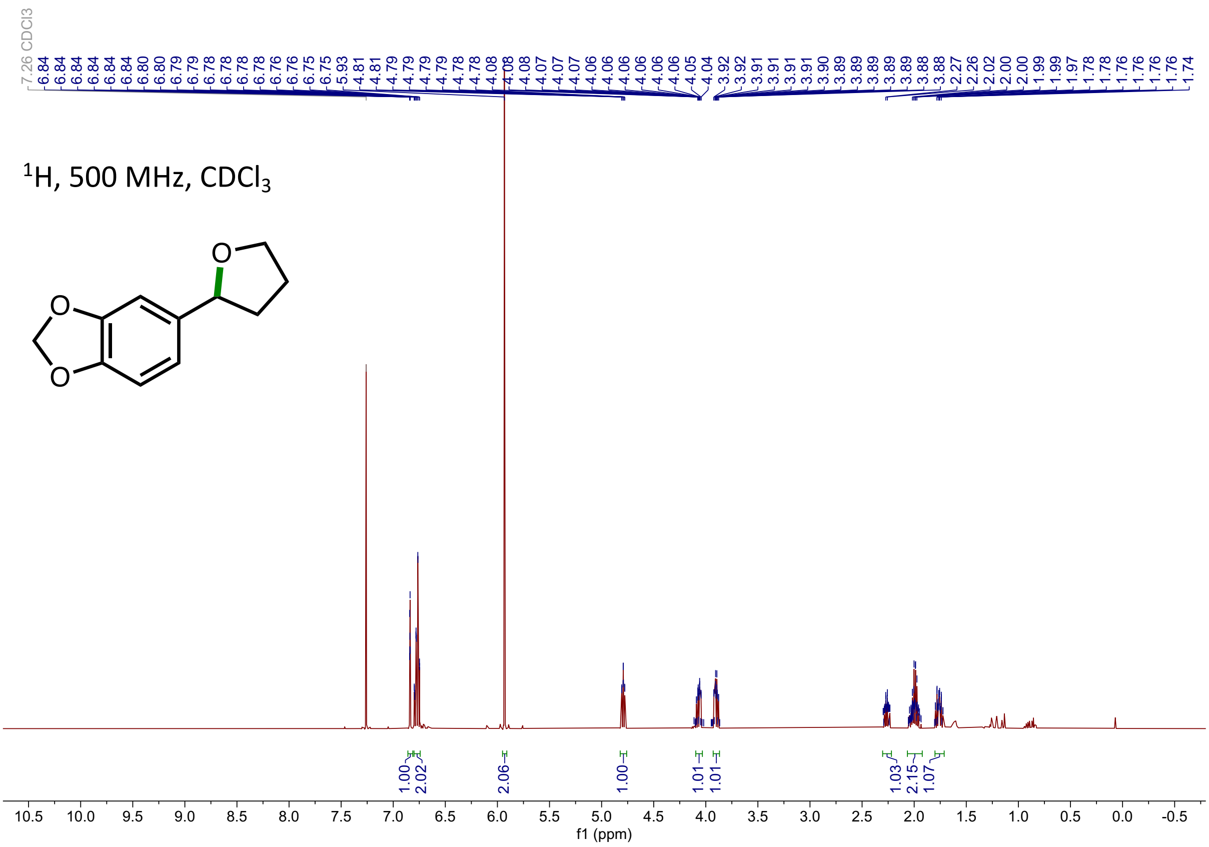

7.26 CDCl3
6.84 6.84 6.84 6.84 6.84 6.80 6.80 6.79 6.79 6.78 6.78 6.78 6.78 6.76 6.76 6.75 6.75 5.93 4.81 4.81 4.79 4.79 4.79 4.79 4.78 4.78 4.08 4.08 4.08 4.07 4.07 4.07 4.06 4.06 4.06 4.06 4.06 4.06 4.05 4.04 3.92 3.92 3.91 3.91 3.91 3.91 3.90 3.89 3.89 3.89 3.89 3.89 3.88 3.88 2.27 2.26 2.02 2.00 2.00 1.99 1.99 1.97 1.78 1.78 1.76 1.76 1.76 1.74
^{1}H, 500 MHz, CDCl$_3$
O
O
O
1.00
2.02
2.06
1.00
1.01
1.01
1.03
2.15
1.07
10.5 10.0 9.5 9.0 8.5 8.0 7.5 7.0 6.5 6.0 5.5 5.0 4.5 4.0 3.5 3.0 2.5 2.0 1.5 1.0 0.5 0.0 -0.5
f1 (ppm)

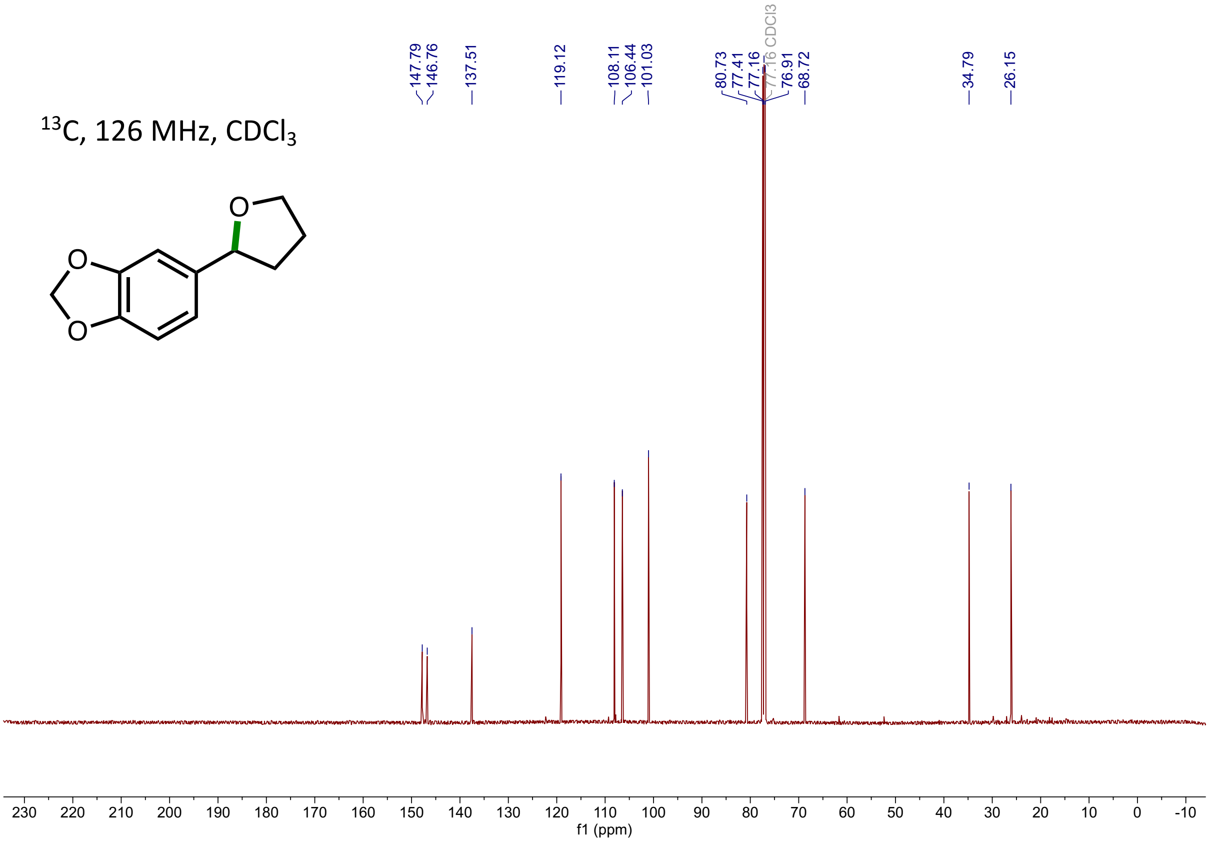

147.79
146.76
137.51
119.12
108.11
106.44
101.03
80.73
77.41
77.16 CDCl3
76.91
68.72
34.79
26.15
^{13}C, 126 MHz, CDCl$_3$
O
O
O
230 220 210 200 190 180 170 160 150 140 130 120 110 100 90 80 70 60 50 40 30 20 10 0 -10
f1 (ppm)

(P21)

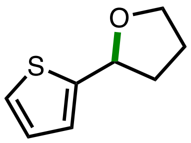

Prepared according to electrochemical general procedure using **S21** (64.3 mg, 0.30 mmol, 1 equiv.), *n*-Bu_4ClO_4 (103 mg, 0.30 mmol, 1 equiv.), dichloromethane (5.7 mL) and TFE (0.3 mL). The yield was determined by crude ^{1}H NMR using 1,3,5-trimethylbenzene (42 µL, 1 equiv.) as an internal standard: 50%. Purification by flash column chromatography (eluent = 5 to 15% EtOAc in hexanes, silica gel) to afford product as colourless oil (20.3 mg, 44% yield).

R_f = 0.41 (eluent = 10% EtOAc in hexanes); **^{1}H NMR (500 MHz, $CDCl_3$)** 7.23 – 7.22 (m, 1H), 6.96 – 6.95 (m, 2H), 5.16 – 5.14 (t, 1H), 4.08 – 4.04 (m, 1H), 3.92 – 3.87 (m, 1H), 2.35 – 2.32 (m, 1H), 2.07 – 2.01 (m, 1H), 2.01 – 1.96 (m, 2H); **^{13}C NMR (126 MHz, CDCl3)** δ 147.2, 126.7, 124.5, 123.8, 76.8, 68.4, 34.7, 26.0.

Data consistent with the literature.[12]

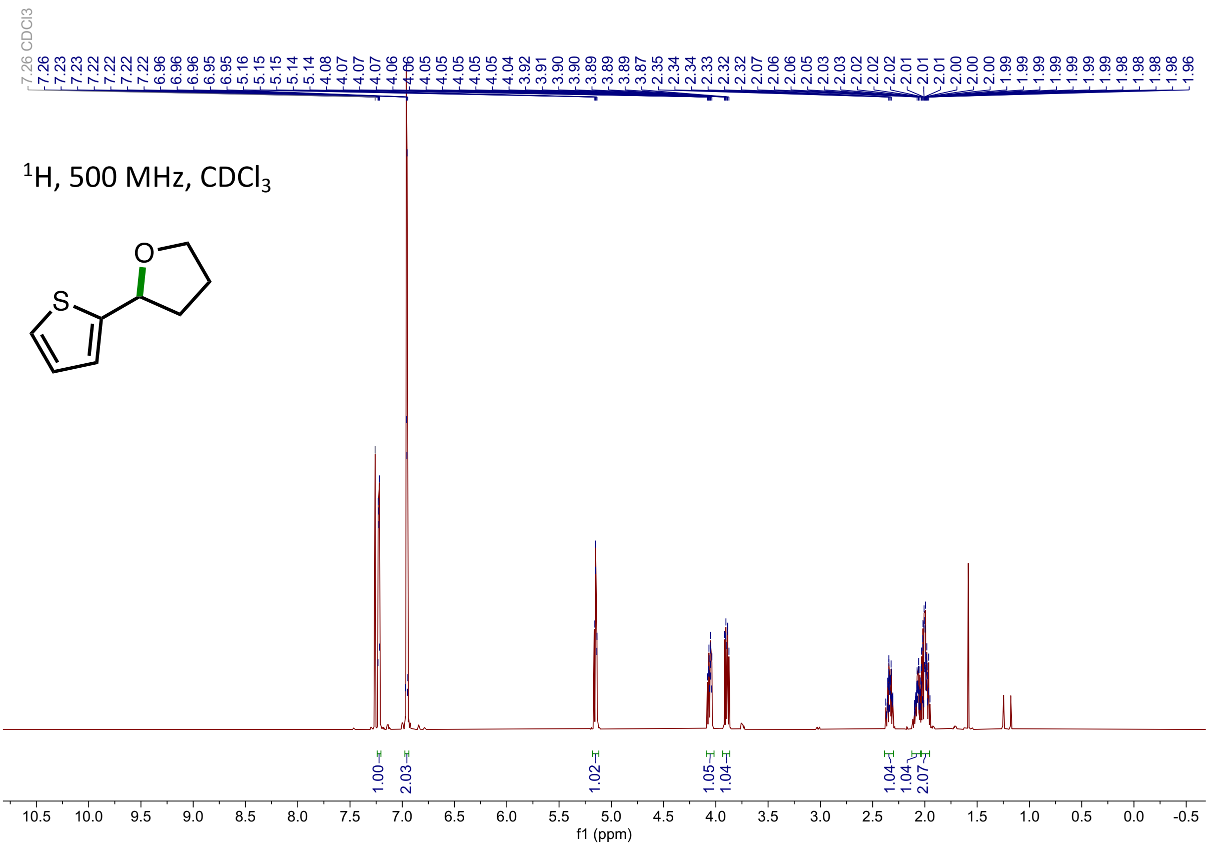
1H, 500 MHz, CDCl3
7.26 CDCl3
7.26 7.23 7.23 7.22 7.22 7.22 6.96 6.96 6.96 6.95 6.95 5.16 5.15 5.15 5.14 4.08 4.07 4.07 4.06 4.06 4.05 4.05 4.05 4.05 4.04 3.92 3.91 3.90 3.90 3.89 3.89 3.89 3.87 2.35 2.34 2.34 2.33 2.32 2.32 2.07 2.06 2.06 2.05 2.03 2.03 2.02 2.02 2.02 2.01 2.01 2.00 2.00 2.00 1.99 1.99 1.99 1.99 1.99 1.99 1.98 1.98 1.98 1.96
S
O
1.00 2.03 1.02 1.05 1.04 1.04 1.04 2.07
f1 (ppm)

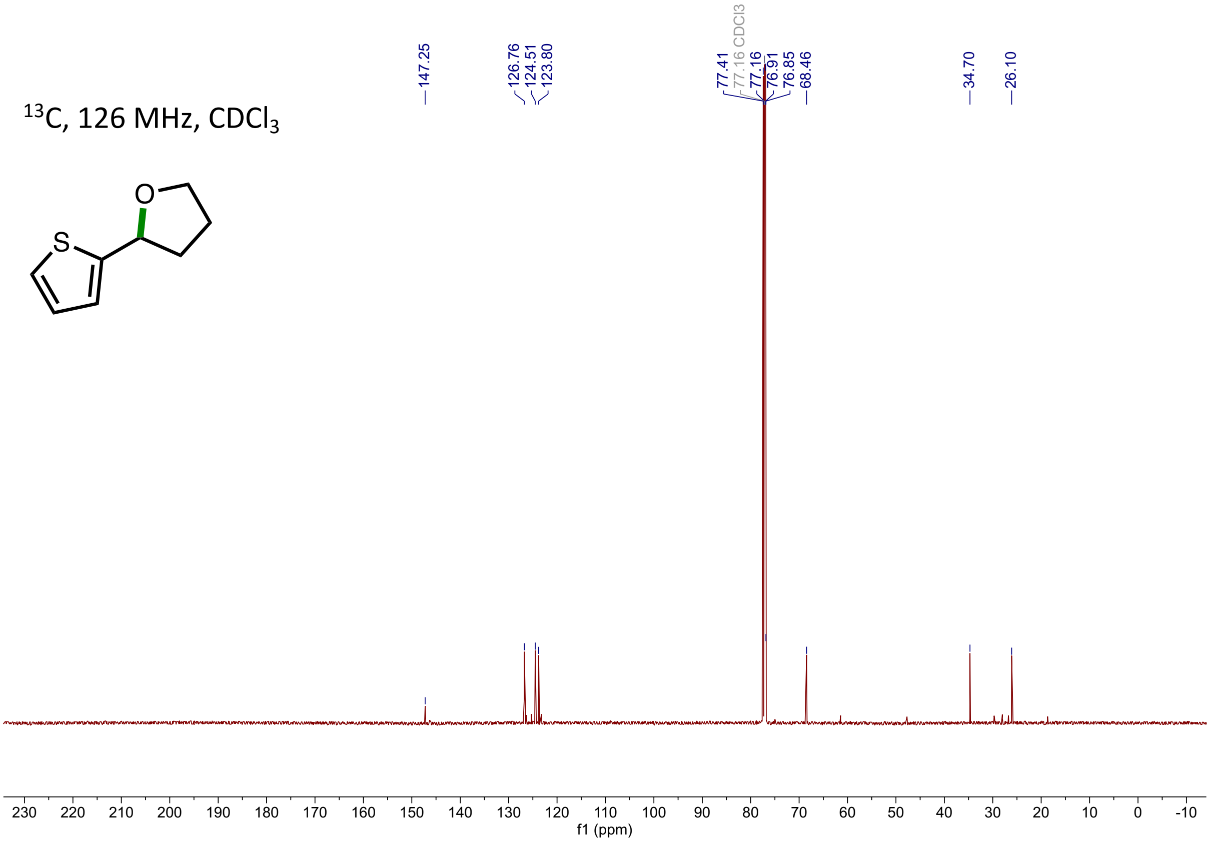
13C, 126 MHz, CDCl3
147.25
126.76 124.51 123.80
77.41 77.16 CDCl3 77.16 76.91 76.85 68.46
34.70
26.10
S
O
f1 (ppm)

(P22)

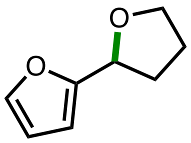

Prepared according to electrochemical general procedure using **S22** (59.5 mg, 0.30 mmol, 1 equiv.), *n*-Bu_4ClO_4 (103 mg, 0.30 mmol, 1 equiv.), dichloromethane (5.7 mL) and TFE (0.3 mL). The yield was determined by crude ^{1}H NMR using 1,3,5-trimethylbenzene (42 µL, 1 equiv.) as an internal standard: 53%. Purification by flash column chromatography (eluent = 5 to 15% EtOAc in hexanes, silica gel) to afford product as yellow oil (12.6 mg, 30% yield).

R_f = 0.42 (eluent = 10% Et_2O in hexanes); **^{1}H NMR (500 MHz, $CDCl_3$)** 7.38 (dd, *J* = 1.8, 0.9 Hz, 1H), 6.32 – 6.31 (dd, *J* = 3.2, 1.8 Hz, 1H), 6.26 – 6.25 (dt, *J* = 3.3, 0.7 Hz, 1H), 4.93 – 4.90 (t, *J* = 6.8 Hz, 1H), 4.01 – 3.96 (m, 1H), 3.90 – 3.86 (m, 1H), 2.20 – 1.98 (m, 4H); **^{13}C NMR (126 MHz, CDCl3)** δ 155.1, 142.4, 110.1, 106.7, 73.9, 68.4, 30.4, 26.1.

Data consistent with the literature.[18]

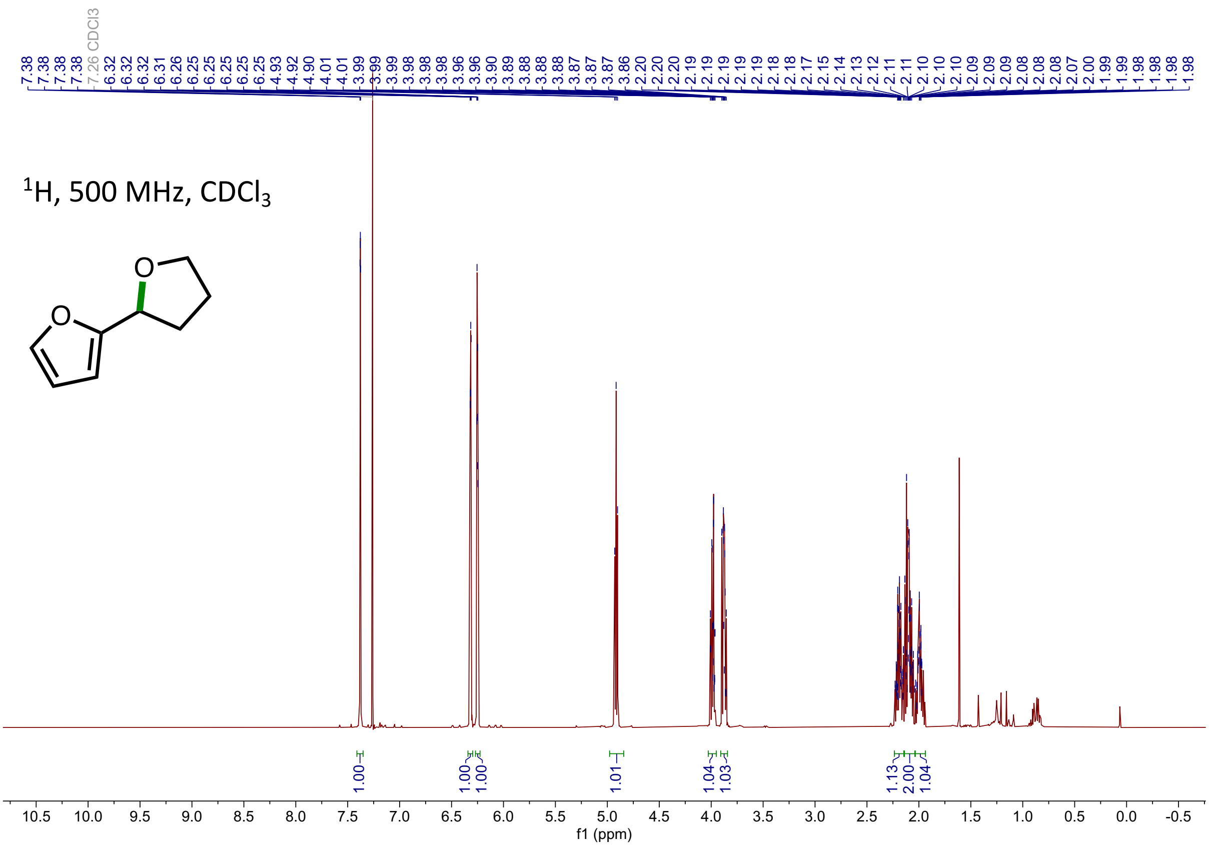

7.38 7.38 7.38 7.38 7.26 CDCl3 6.32 6.32 6.32 6.31 6.26 6.25 6.25 6.25 6.25 6.25 4.93 4.92 4.90 4.01 4.01 3.99 3.99 3.99 3.98 3.98 3.98 3.96 3.96 3.90 3.89 3.88 3.88 3.88 3.87 3.87 3.86 2.20 2.20 2.20 2.19 2.19 2.19 2.19 2.18 2.18 2.17 2.15 2.14 2.13 2.12 2.11 2.11 2.10 2.10 2.10 2.09 2.09 2.09 2.08 2.08 2.08 2.07 2.00 1.99 1.99 1.98 1.98 1.98 1.98
¹H, 500 MHz, CDCl3
O
O
1.00
1.00
1.00
1.01
1.04
1.03
1.13
2.00
1.04
10.5 10.0 9.5 9.0 8.5 8.0 7.5 7.0 6.5 6.0 5.5 5.0 4.5 4.0 3.5 3.0 2.5 2.0 1.5 1.0 0.5 0.0 -0.5
f1 (ppm)

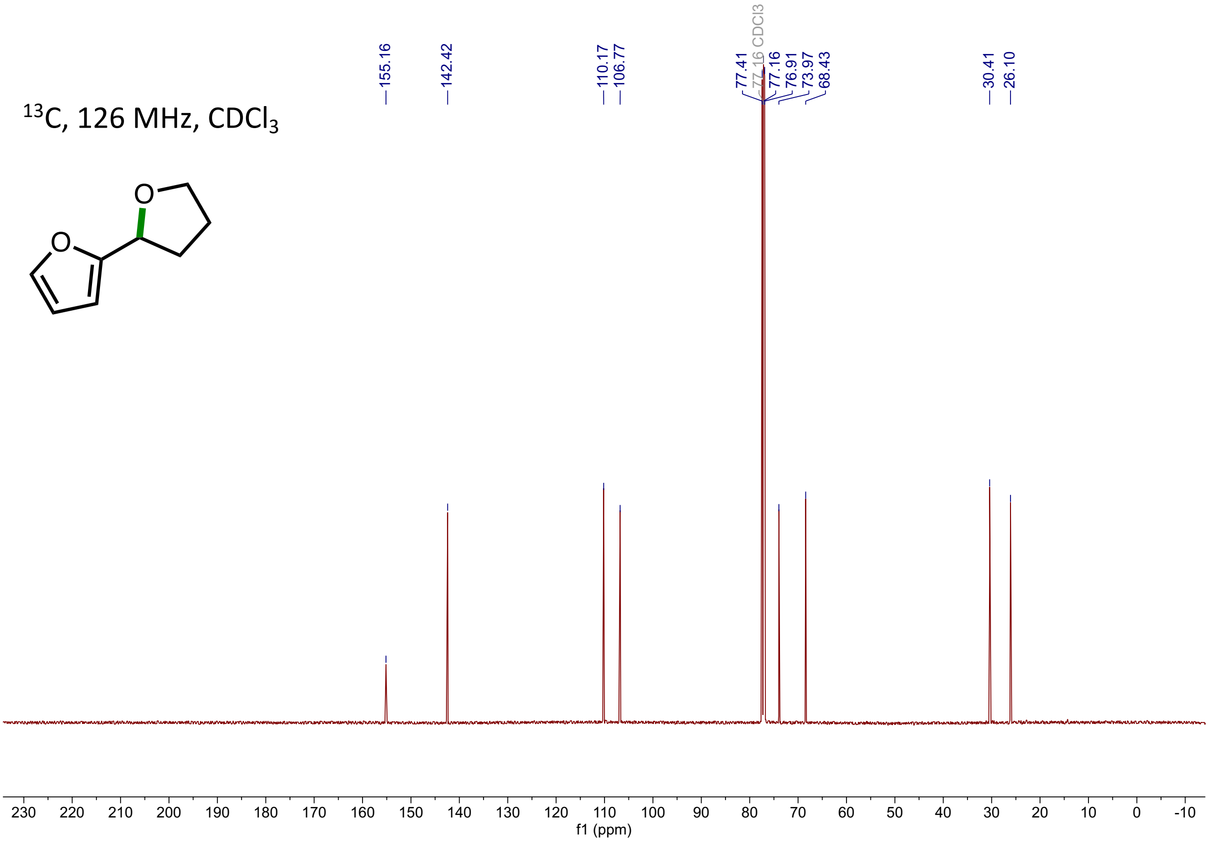

155.16
142.42
110.17
106.77
77.41
77.16 CDCl3
77.16
76.91
73.97
68.43
30.41
26.10
¹³C, 126 MHz, CDCl3
O
O
230 220 210 200 190 180 170 160 150 140 130 120 110 100 90 80 70 60 50 40 30 20 10 0 -10
f1 (ppm)

(P23)

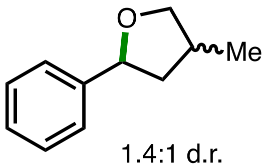

Prepared according to electrochemical general procedure using **S23** (66.7 mg, 0.30 mmol, 1 equiv.), *n*-Bu_4ClO_4 (103 mg, 0.30 mmol, 1 equiv.), dichloromethane (5.7 mL) and TFE (0.3 mL). The yield was determined by crude ^{1}H NMR using 1,3,5-trimethylbenzene (42 µL, 1 equiv.) as an internal standard: 90% as 1.4:1 mixture of diastereomers. Purification by flash column chromatography (eluent = 5 to 15% EtOAc in hexanes, silica gel) to afford product as colourless oil (39.4 mg, 81% yield) as 1:1 mixture of diastereomers.

R_f = 0.42 (eluent = 10% EtOAc in hexanes).

Selected data for diastereomer 1:

^{1}H NMR (500 MHz, $CDCl_3$) δ 5.01 – 4.95 (m, 1H), 4.17 (dd, *J* = 8.2, 6.9 Hz, 1H), 3.42 (dd, *J* = 8.2, 7.0 Hz, 1H); **^{13}C NMR (126 MHz, $CDCl_3$)** δ 144.0, 128.3, 127.1, 125.6, 80.2, 75.8, 42.8, 33.3, 17.8.

Selected data for diastereomer 2:

^{1}H NMR (500 MHz, $CDCl_3$) δ 4.86 (dd, *J* = 9.9, 5.7 Hz, 1H), 4.04 (t, *J* = 7.9 Hz, 1H), 3.53 (t, *J* = 8.0 Hz, 1H); **^{13}C NMR (126 MHz, $CDCl_3$)** δ 143.5, 128.4, 127.2, 125.7, 81.7, 75.6, 44.0, 35.1, 17.5.

Data consistent with the literature.[19]

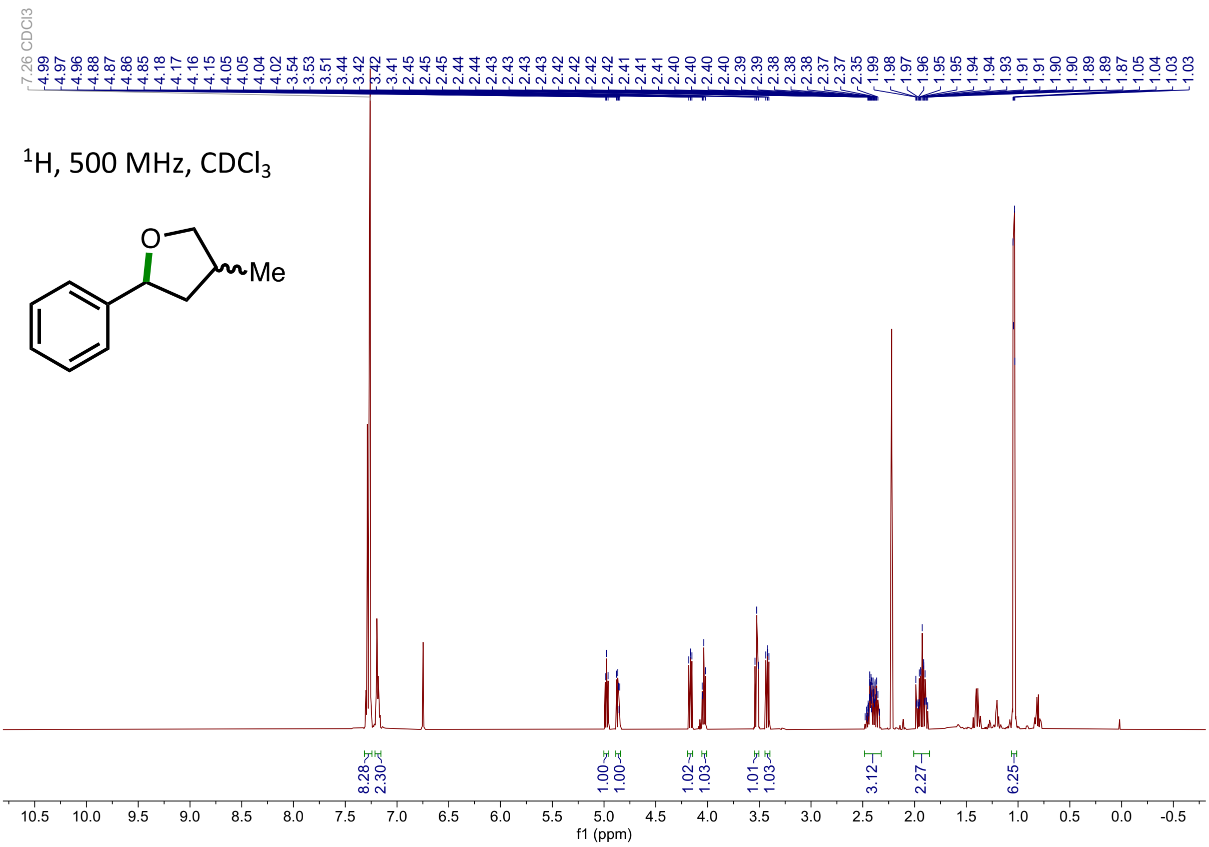

7.26 CDCl3
4.99 4.97 4.96 4.88 4.87 4.86 4.85 4.18 4.17 4.16 4.15 4.05 4.05 4.04 4.02 3.54 3.53 3.51 3.44 3.42 3.42 3.41 2.45 2.45 2.45 2.44 2.44 2.43 2.43 2.43 2.43 2.42 2.42 2.42 2.42 2.41 2.41 2.41 2.40 2.40 2.40 2.40 2.39 2.39 2.38 2.38 2.38 2.37 2.35 1.99 1.98 1.97 1.96 1.95 1.95 1.94 1.94 1.93 1.91 1.91 1.90 1.89 1.89 1.87 1.05 1.04 1.03 1.03
¹H, 500 MHz, CDCl₃
O
Me
8.28 2.30 1.00 1.00 1.02 1.03 1.01 1.03 3.12 2.27 6.25
10.5 10.0 9.5 9.0 8.5 8.0 7.5 7.0 6.5 6.0 5.5 5.0 4.5 4.0 3.5 3.0 2.5 2.0 1.5 1.0 0.5 0.0 -0.5
f1 (ppm)

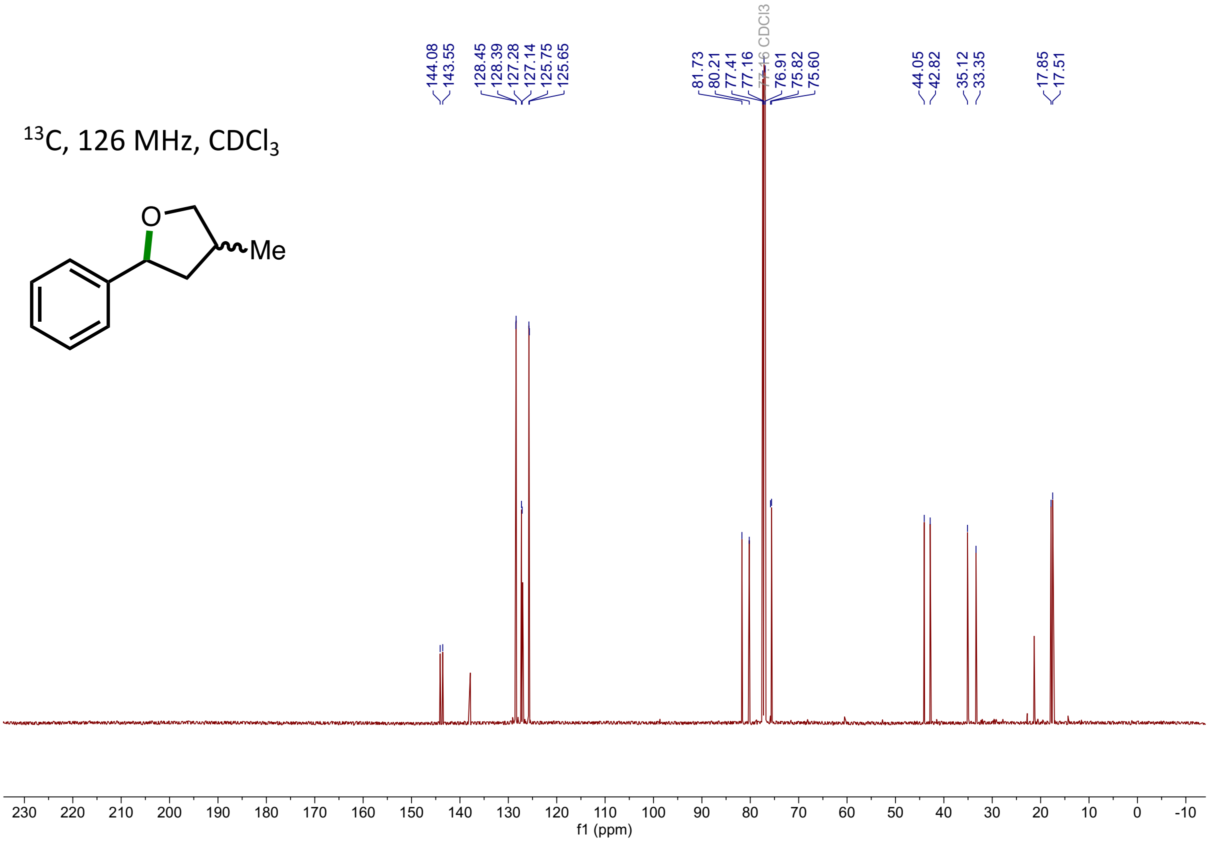

144.08 143.55 128.45 128.39 127.28 127.14 125.75 125.65
81.73 80.21 77.41 77.16 77.16 CDCl3 76.91 75.82 75.60
44.05 42.82 35.12 33.35 17.85 17.51
¹³C, 126 MHz, CDCl₃
O
Me
230 220 210 200 190 180 170 160 150 140 130 120 110 100 90 80 70 60 50 40 30 20 10 0 -10
f1 (ppm)

(P24)

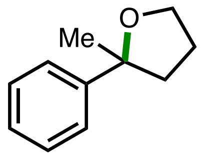

Prepared according to electrochemical general procedure using **S24** (66.7 mg, 0.30 mmol, 1 equiv.), *n*-Bu_4ClO_4 (103 mg, 0.30 mmol, 1 equiv.), dichloromethane (5.7 mL) and TFE (0.3 mL). The yield was determined by crude ^{1}H NMR using 1,3,5-trimethylbenzene (42 µL, 1 equiv.) as an internal standard: 85%. Purification by flash column chromatography (eluent = 5 to 15% EtOAc in hexanes, silica gel) to afford product as pale-yellow oil (26.0 mg, 53% yield).

R_f = 0.5 (eluent = 5% EtOAc in hexanes); **^{1}H NMR (500 MHz, $CDCl_3$)** δ 7.41 – 7.39 (m, 2H), 7.34 – 7.31 (m, 2H), 7.26 – 7.20 (m, 1H), 4.03 – 4.01 (m, 1H), 3.91 (td, *J* = 8.1, 5.5 Hz, 1H), 2.22 – 2.19 (m, 1H), 2.05 – 1.97 (m, 2H), 1.83 – 1.78 (m, 1H), 1.53 (s, 3H); **^{13}C NMR (126 MHz, $CDCl_3$)** δ 148.3, 128.2, 126.4, 124.8, 84.4, 67.6, 39.6, 29.8, 25.9.

Data consistent with the literature.[20]

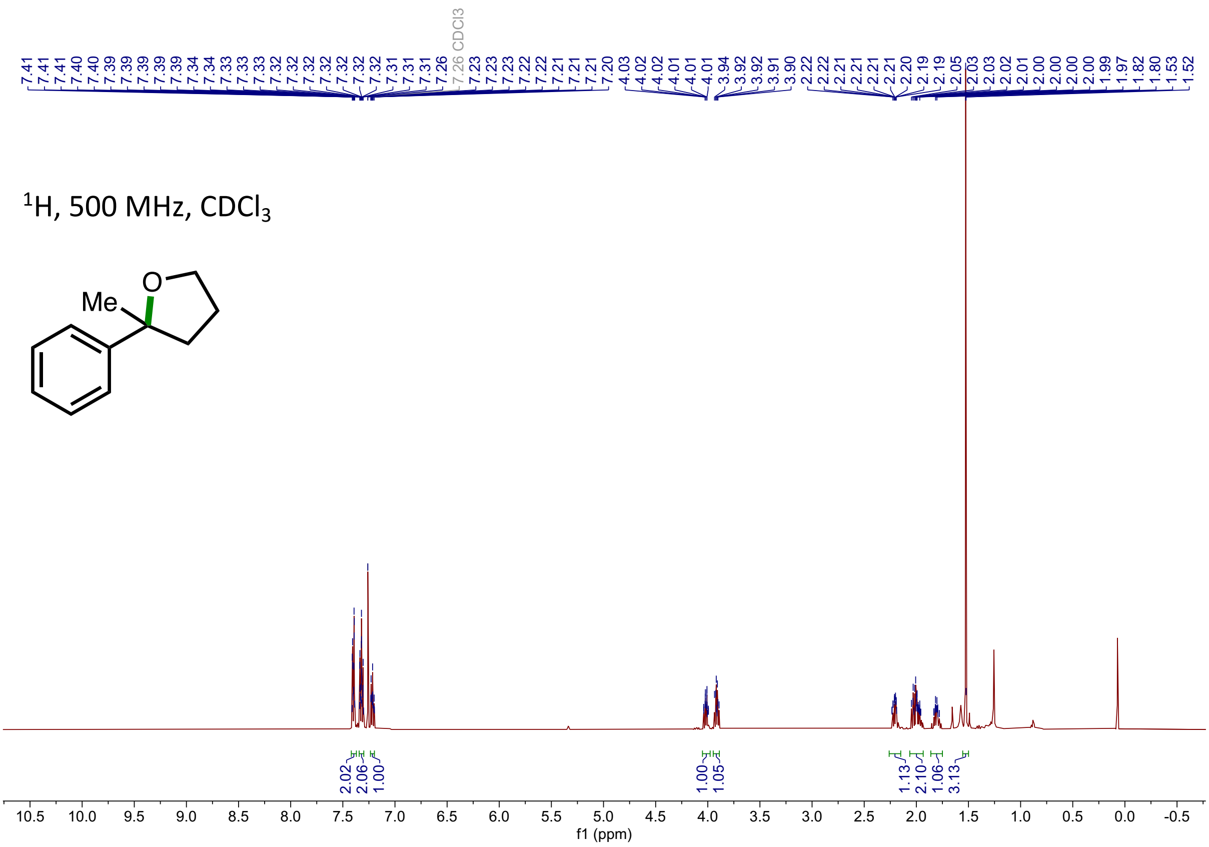
7.41 7.41 7.41 7.40 7.39 7.39 7.39 7.39 7.39 7.34 7.33 7.33 7.33 7.32 7.32 7.32 7.32 7.32 7.32 7.31 7.31 7.31 7.26 CDCl3 7.23 7.23 7.23 7.22 7.22 7.21 7.21 7.21 7.20 4.03 4.02 4.02 4.01 4.01 4.01 3.94 3.92 3.92 3.91 3.90 2.22 2.22 2.21 2.21 2.21 2.20 2.19 2.19 2.05 2.03 2.03 2.02 2.01 2.00 2.00 2.00 2.00 1.99 1.97 1.82 1.80 1.53 1.52
^{1}H, 500 MHz, CDCl$_3$
Me
O
2.02
2.06
1.00
1.00
1.05
1.13
2.10
1.06
3.13
10.5 10.0 9.5 9.0 8.5 8.0 7.5 7.0 6.5 6.0 5.5 5.0 4.5 4.0 3.5 3.0 2.5 2.0 1.5 1.0 0.5 0.0 -0.5
f1 (ppm)

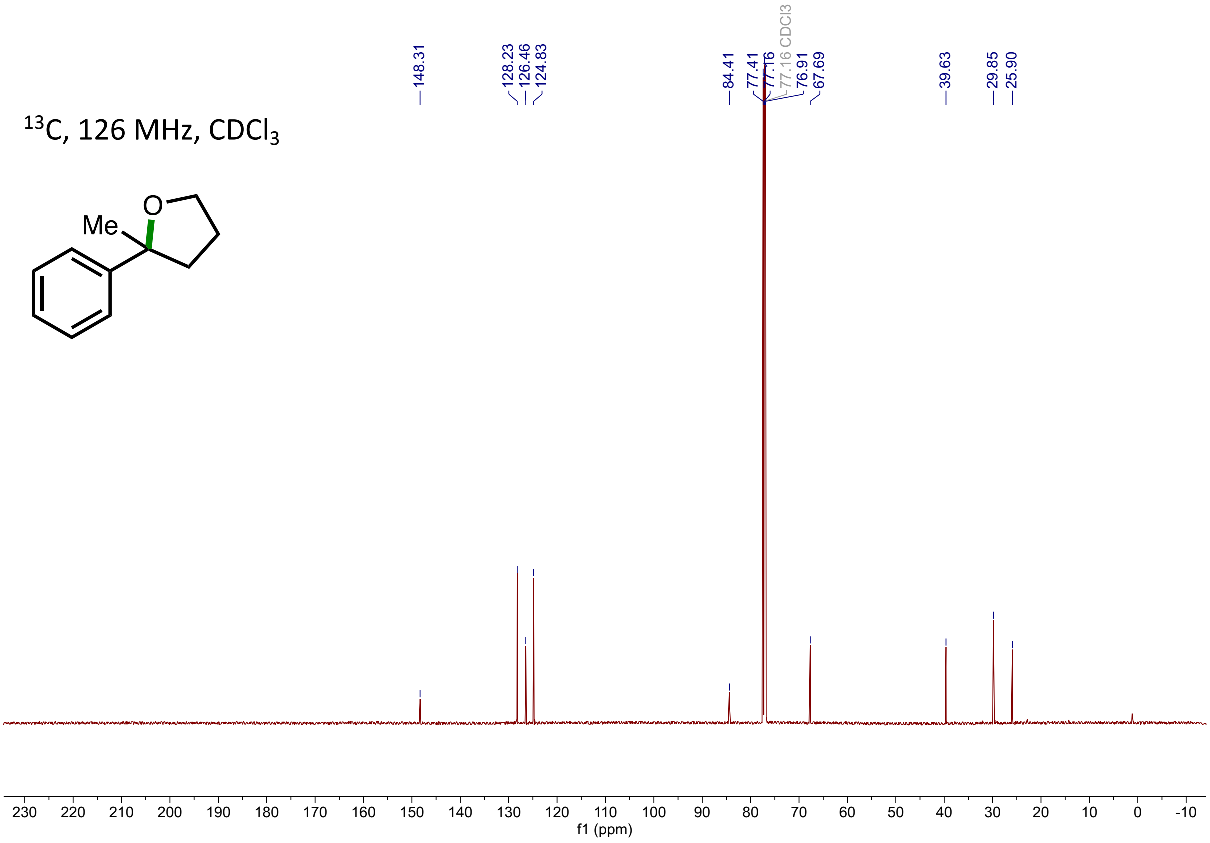
148.31
128.23 126.46 124.83
84.41 77.41 77.16 77.16 CDCl3 76.91 67.69
39.63 29.85 25.90
^{13}C, 126 MHz, CDCl$_3$
Me
O
230 220 210 200 190 180 170 160 150 140 130 120 110 100 90 80 70 60 50 40 30 20 10 0 -10
f1 (ppm)

(P25)

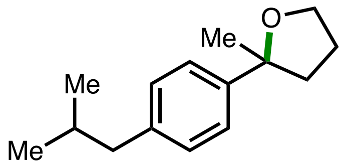

Prepared according to electrochemical general procedure using **S25** (79.32 mg, 0.30 mmol, 1 equiv.), *n*-Bu_4ClO_4 (103 mg, 0.30 mmol, 1 equiv.), dichloromethane (5.7 mL) and TFE (0.3 mL). The yield was determined by crude ^{1}H NMR using 1,3,5-trimethylbenzene (42 µL, 1 equiv.) as an internal standard: 71%. Purification by flash column chromatography (eluent = 5 to 15% EtOAc in hexanes, silica gel) to afford product as pale-yellow oil (5.4 mg, 8% yield).

R_f = 0.62 (eluent = 10% EtOAc in hexanes); **ν_{max} / cm^{-1}** (thin film) 2960, 1718,1508; **^{1}H NMR (400 MHz, $CDCl_3$)** δ 7.29 – 7.25 (m, 2H), 7.12 – 7.03 (m, 2H), 4.10 – 3.68 (m, 2H), 2.43 (d, *J* = 7.2 Hz, 2H), 2.23 – 2.10 (m, 1H), 2.05 – 1.91 (m, 2H), 1.86 1.74 (m, 2H), 1.50 (s, 3H), 0.90 (s, 3H), 0.87 (s, 3H); **^{13}C NMR (400 MHz, $CDCl_3$)** δ 145.39, 139.66, 128.82, 124.44, 84.22, 77.36, 77.24, 77.04, 76.72, 67.52, 45.03, 39.47, 30.24, 29.73, 25.80, 22.45; **HRMS (EI$^+$)** ***m/z*** [M - CH_3] Calcd for $C_{14}H_{19}O$ 203.1431; found 203.1400.

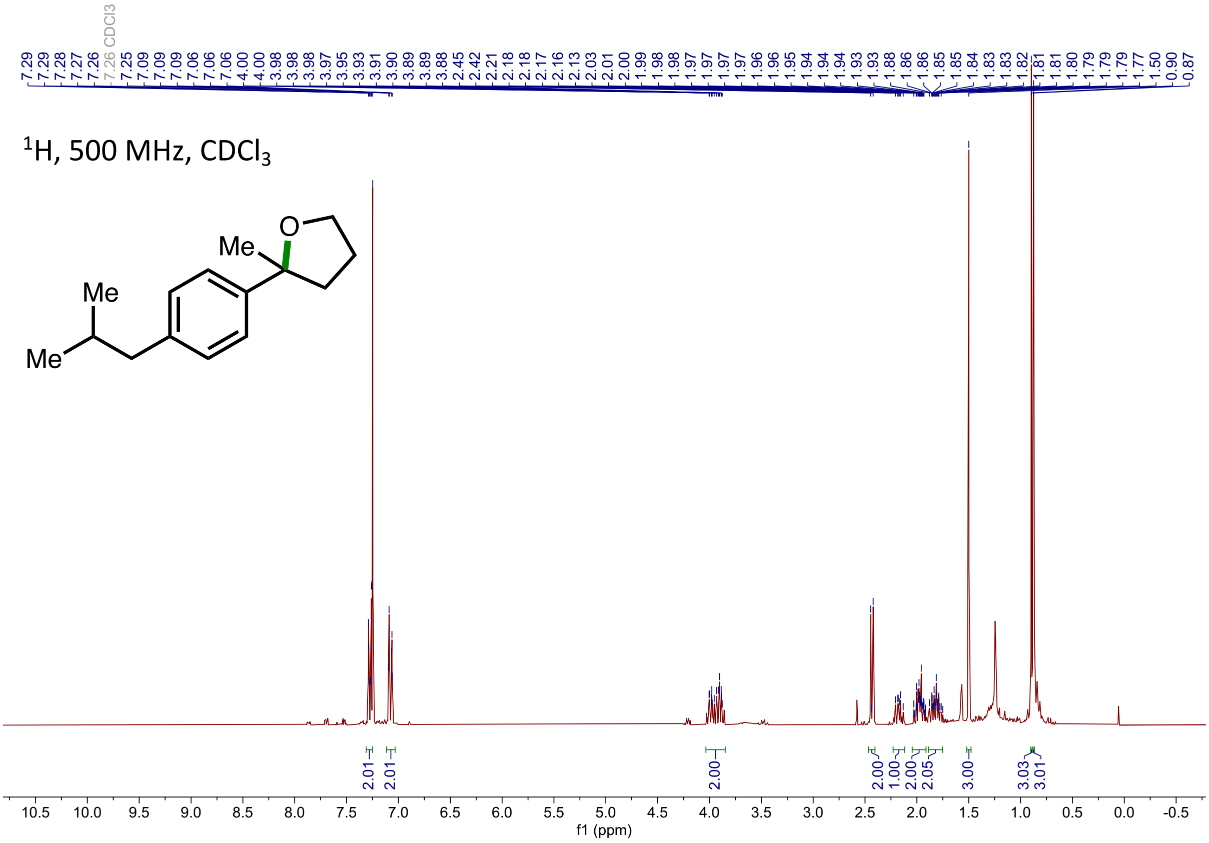

7.29 7.29 7.28 7.27 7.26 7.26 CDCl3 7.25 7.09 7.09 7.09 7.06 7.06 7.06 4.00 3.98 3.98 3.98 3.97 3.95 3.93 3.91 3.90 3.89 3.89 3.88 2.45 2.42 2.21 2.18 2.18 2.17 2.16 2.13 2.03 2.01 2.00 1.99 1.98 1.98 1.97 1.97 1.97 1.97 1.96 1.96 1.95 1.94 1.94 1.94 1.93 1.93 1.88 1.86 1.86 1.85 1.85 1.84 1.83 1.83 1.82 1.81 1.81 1.80 1.79 1.79 1.77 1.50 0.90 0.87
^{1}H, 500 MHz, CDCl$_3$
O
Me
Me
Me
2.01 2.01 2.00 2.00 1.00 2.00 2.05 3.00 3.03 3.01
10.5 10.0 9.5 9.0 8.5 8.0 7.5 7.0 6.5 6.0 5.5 5.0 4.5 4.0 3.5 3.0 2.5 2.0 1.5 1.0 0.5 0.0 -0.5
f1 (ppm)

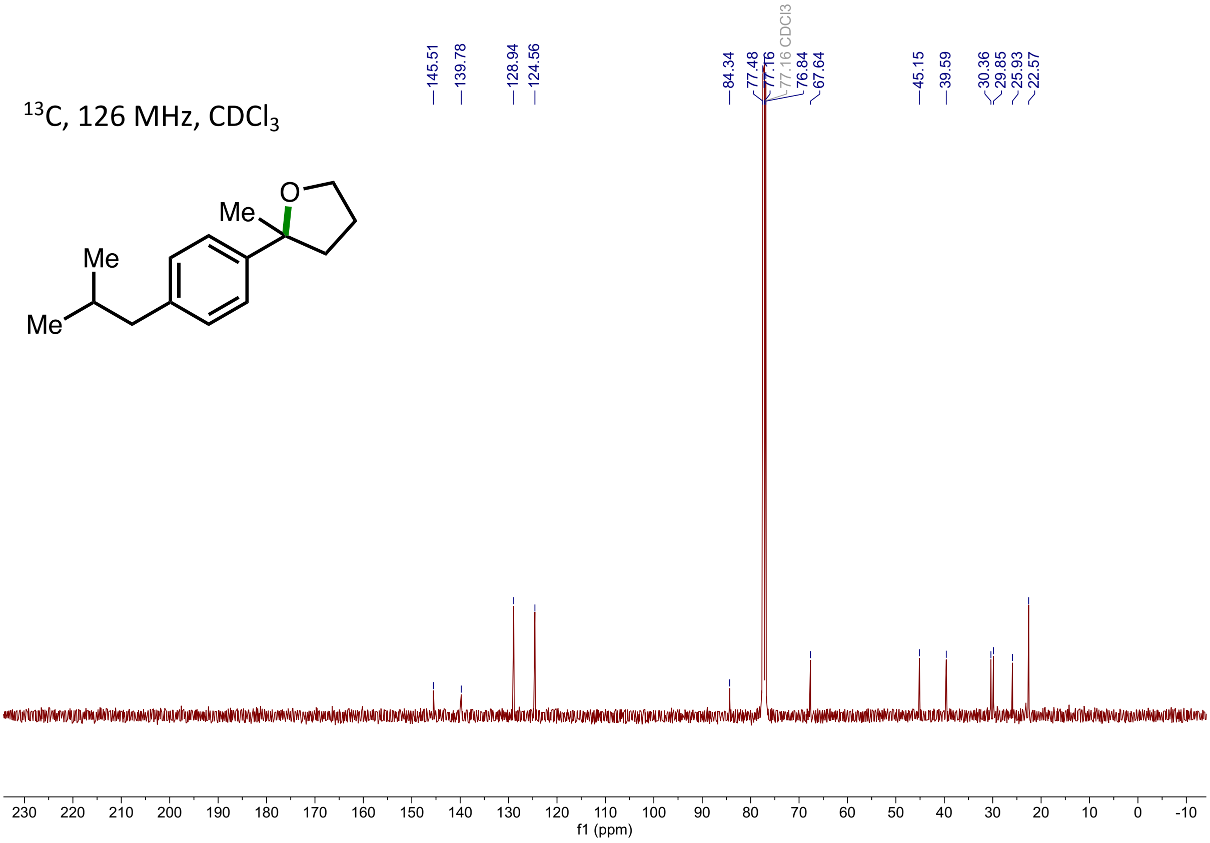

145.51 139.78 128.94 124.56 84.34 77.48 77.16 77.16 CDCl3 76.84 67.64 45.15 39.59 30.36 29.85 25.93 22.57
^{13}C, 126 MHz, CDCl$_3$
O
Me
Me
Me
230 220 210 200 190 180 170 160 150 140 130 120 110 100 90 80 70 60 50 40 30 20 10 0 -10
f1 (ppm)

(P27)

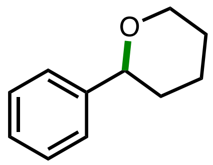

Prepared according to electrochemical general procedure using **S27** (66.7 mg, 0.30 mmol, 1 equiv.), *n*-Bu_4ClO_4 (103 mg, 0.30 mmol, 1 equiv.), dichloromethane (5.7 mL) and TFE (0.3 mL). The yield was determined by crude ^{1}H NMR using 1,3,5-trimethylbenzene (42 µL, 1 equiv.) as an internal standard: 49%. Purification by flash column chromatography (eluent = 5 to 15% EtOAc in hexanes, silica gel) to afford product as colourless oil (20 mg, 41% yield).

R_f = 0.45 (eluent = 10% EtOAc in hexanes); **^{1}H NMR (500 MHz, $CDCl_3$)** 7.36 – 7.30 (m, 4H), 7.27 – 7.25 (m, 1H), 4.33 – 4.31 (dd, *J* = 10.7, 2.3 Hz, 1H), 4.16 – 4.10 (m, 2H), 3.65 – 3.59 (m, 1H), 1.96 – 1.93 (m, 1H), 1.85 – 1.81 (m, 1H), 1.72 – 1.66 (m, 2H), 1.60 – 1.57 (m, 1H); **^{13}C NMR (126 MHz, CDCl3)** δ 143.46, 128.43, 127.43, 125.99, 80.31, 69.16, 34.16, 26.04, 24.16.

Data consistent with the literature.[21]

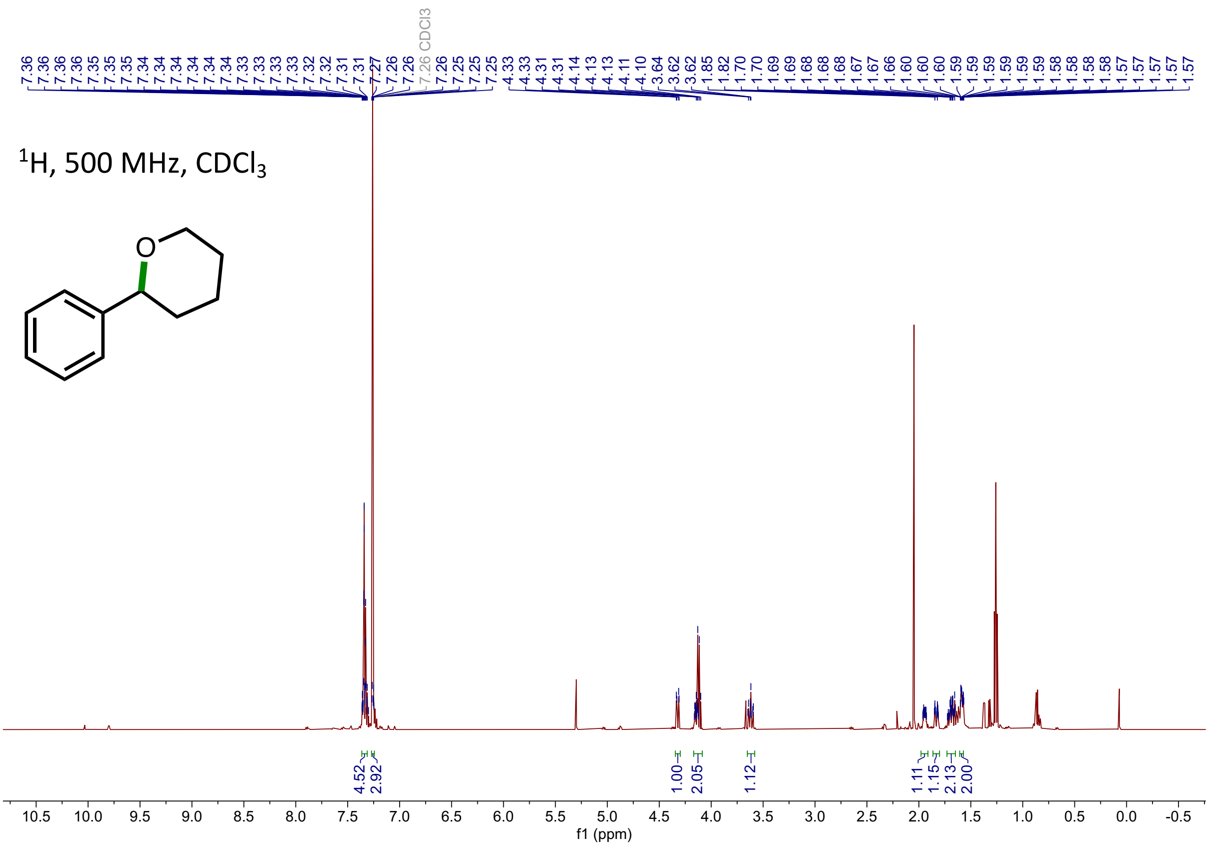
1H, 500 MHz, CDCl3
7.26 CDCl3
O
4.52
2.92
1.00
2.05
1.12
1.11
1.15
2.13
2.00
f1 (ppm)

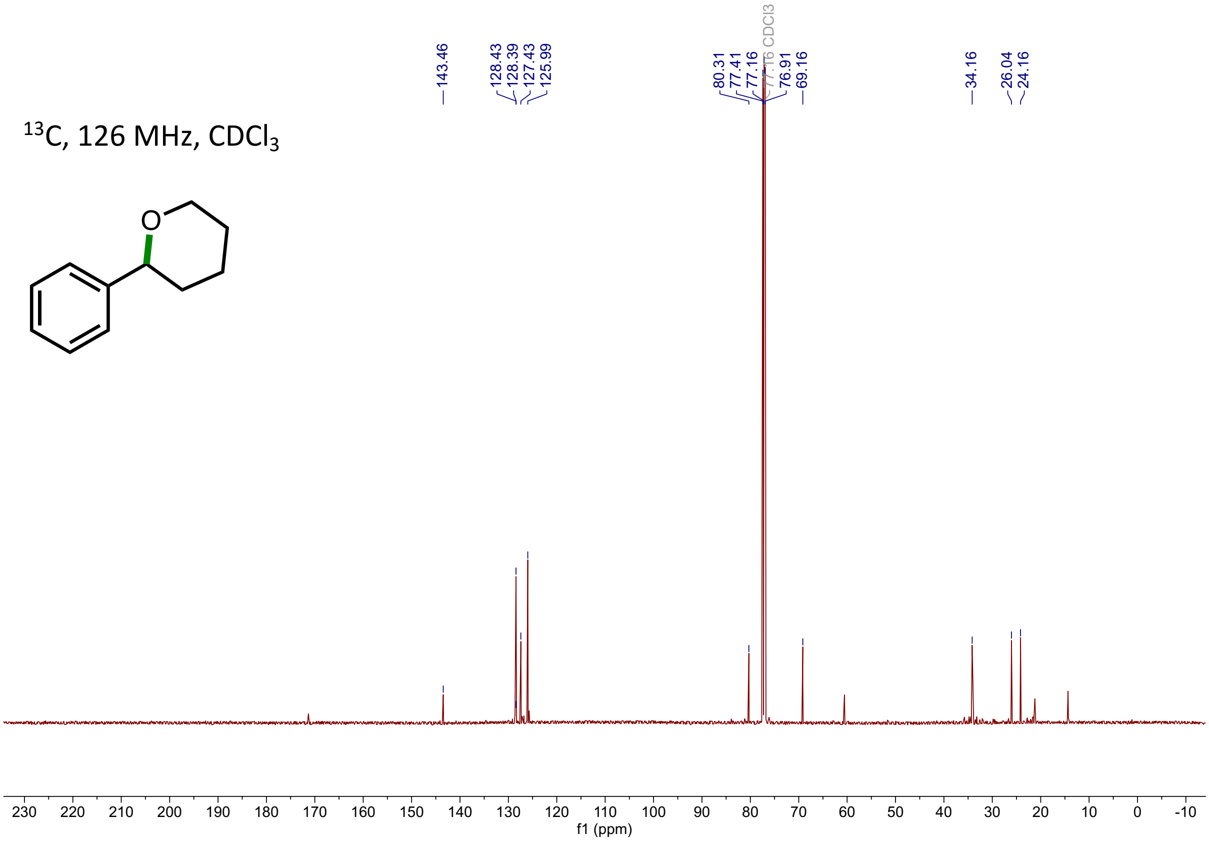
13C, 126 MHz, CDCl3
143.46
128.43
128.39
127.43
125.99
80.31
77.41
77.16
77.16 CDCl3
76.91
69.16
34.16
26.04
24.16
O
f1 (ppm)

(P29)

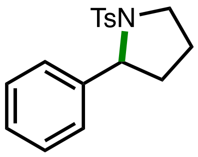

Prepared according to electrochemical general procedure using **S29** (108.5 mg, 0.30 mmol, 1 equiv.), *n*-Bu_4ClO_4 (103 mg, 0.30 mmol, 1 equiv.), dichloromethane (5.7 mL) and TFE (0.3 mL). The yield was determined by crude ^{1}H NMR using 1,3,5-trimethylbenzene (42 µL, 1 equiv.) as an internal standard: 55%. Purification by flash column chromatography (eluent = 5 to 15% EtOAc in hexanes, silica gel) to afford product as white solid (47.9 mg, 53% yield).

Mp.: 58-61 °C; $\mathbf{R_f}$ = 0.37 (eluent = 20% EtOAc in hexanes); **^{1}H NMR (500 MHz, $CDCl_3$)** 7.68 – 7.66 (m, 2H), 7.31 – 7.22 (m, 7H), 4.80 – 4.77 (dd, *J* = 8.1, 3.8 Hz, 1H), 3.66 – 3.57 (m, 1H), 3.45 – 3.38 (m, 1H), 2.43 (s, 3H), 2.01 – 1.95 (m, 1H), 1.90 – 1.79 (m, 2H), 1.69 – 1.65 (m, 1H); **^{13}C NMR (126 MHz, CDCl3)** δ 143.4, 143.1, 135.2, 129.7, 128.4, 127.6, 127.1, 126.2, 63.3, 49.5, 35.9, 24.1, 21.6.

Data consistent with the literature.[22]

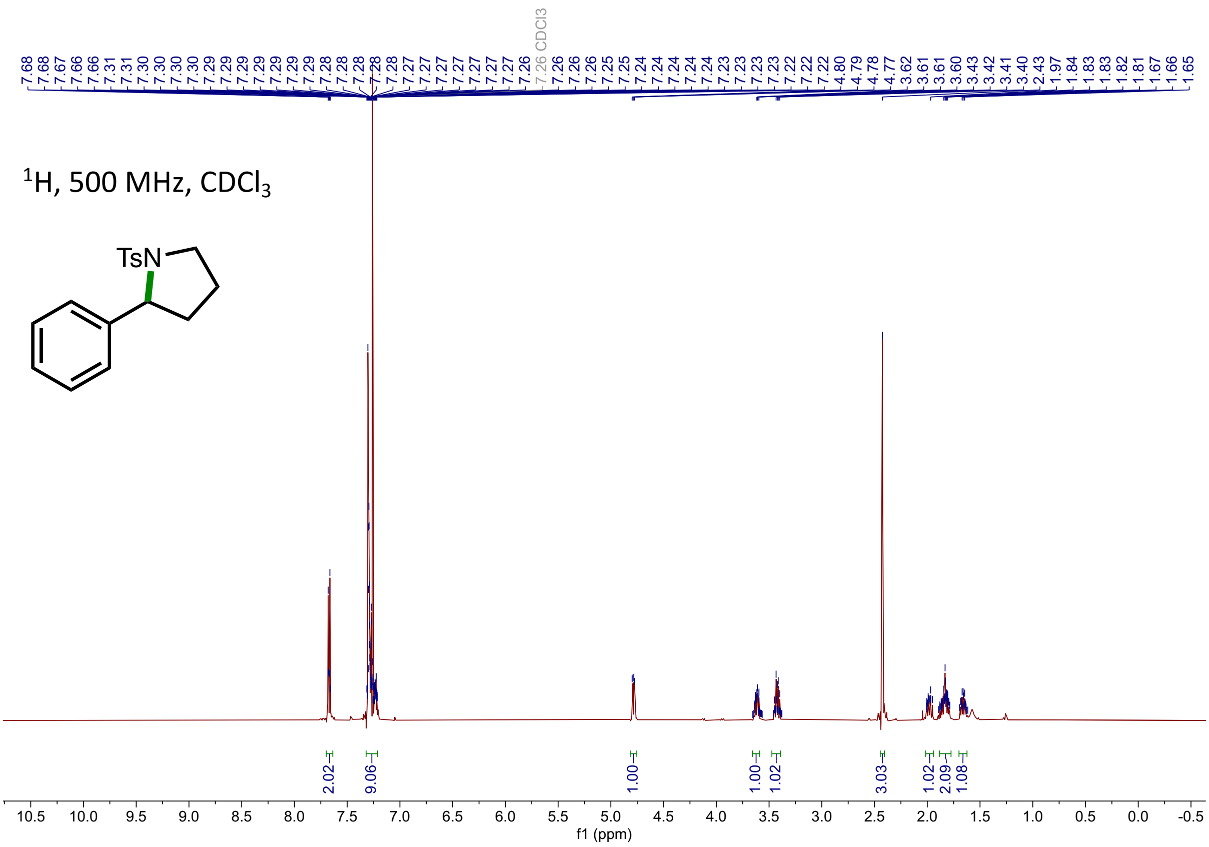

7.68 7.68 7.67 7.66 7.66 7.31 7.31 7.30 7.30 7.30 7.30 7.29 7.29 7.29 7.29 7.29 7.29 7.29 7.28 7.28 7.28 7.28 7.28 7.27 7.27 7.27 7.27 7.27 7.27 7.27 7.26 7.26 CDCl3 7.26 7.26 7.26 7.25 7.25 7.24 7.24 7.24 7.24 7.24 7.23 7.23 7.23 7.23 7.22 7.22 7.22 4.80 4.79 4.78 4.77 3.62 3.61 3.61 3.60 3.43 3.42 3.41 3.40 2.43 1.97 1.84 1.83 1.83 1.82 1.81 1.67 1.66 1.65
1H, 500 MHz, CDCl3
TsN
2.02 9.06 1.00 1.00 1.02 3.03 1.02 2.09 1.08
10.5 10.0 9.5 9.0 8.5 8.0 7.5 7.0 6.5 6.0 5.5 5.0 4.5 4.0 3.5 3.0 2.5 2.0 1.5 1.0 0.5 0.0 -0.5
f1 (ppm)

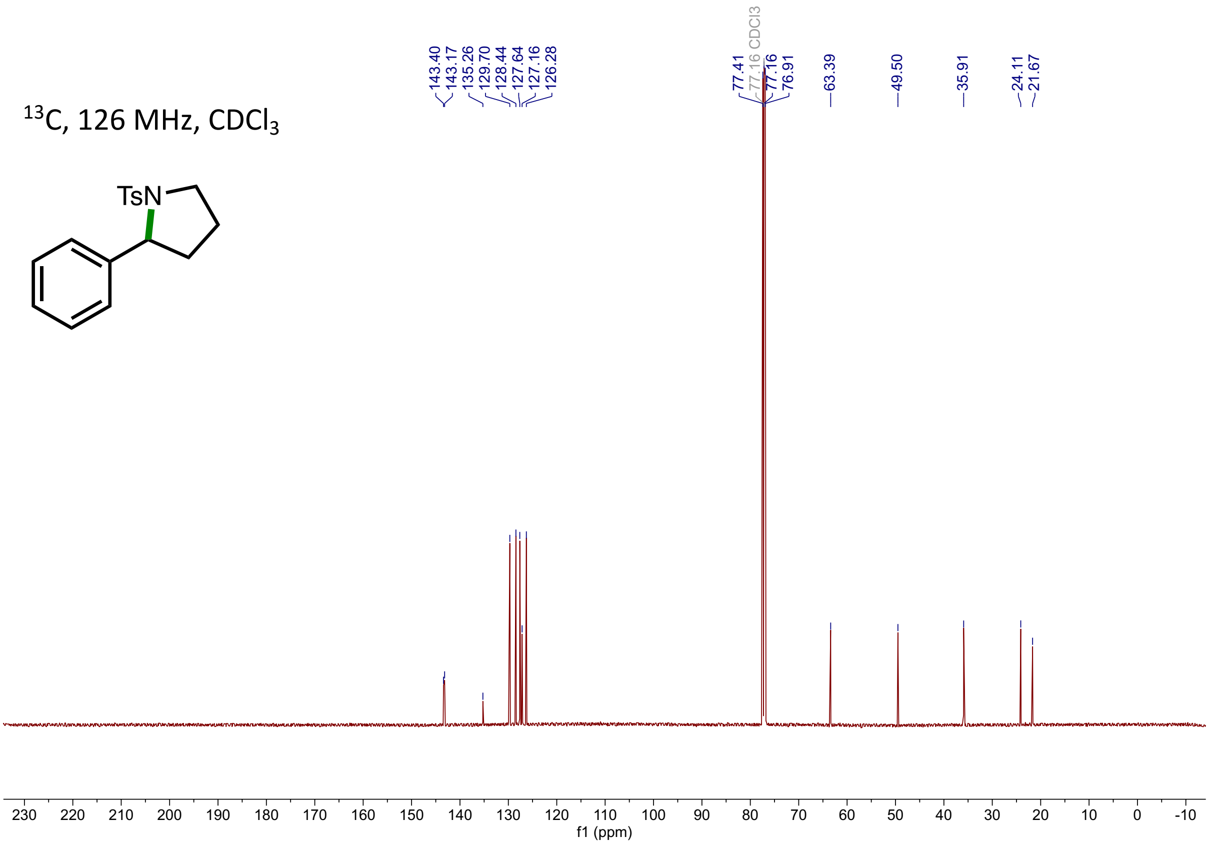

143.40 143.17 135.26 129.70 128.44 127.64 127.16 126.28
77.41 77.16 CDCl3 77.16 76.91
63.39
49.50
35.91
24.11 21.67
13C, 126 MHz, CDCl3
TsN
230 220 210 200 190 180 170 160 150 140 130 120 110 100 90 80 70 60 50 40 30 20 10 0 -10
f1 (ppm)

(P31)

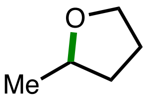

Prepared according to electrochemical general procedure using **S31** (81.1 mg, 0.30 mmol, 1 equiv.), *n*-Bu_4ClO_4 (103 mg, 0.30 mmol, 1 equiv.), dichloromethane (5.7 mL) and TFE (0.3 mL). The yield was determined by crude ^{1}H NMR using 1,3,5-trimethylbenzene (42 µL, 1 equiv.) as an internal standard: 46%. Benzophenone was also observed: 82% on NMR.

$$\% \text{ Yield of the Product} = \left(\frac{0.46}{1.00}\right) \times \left(\frac{3.00}{3.00}\right) \times 100 = 46\%$$

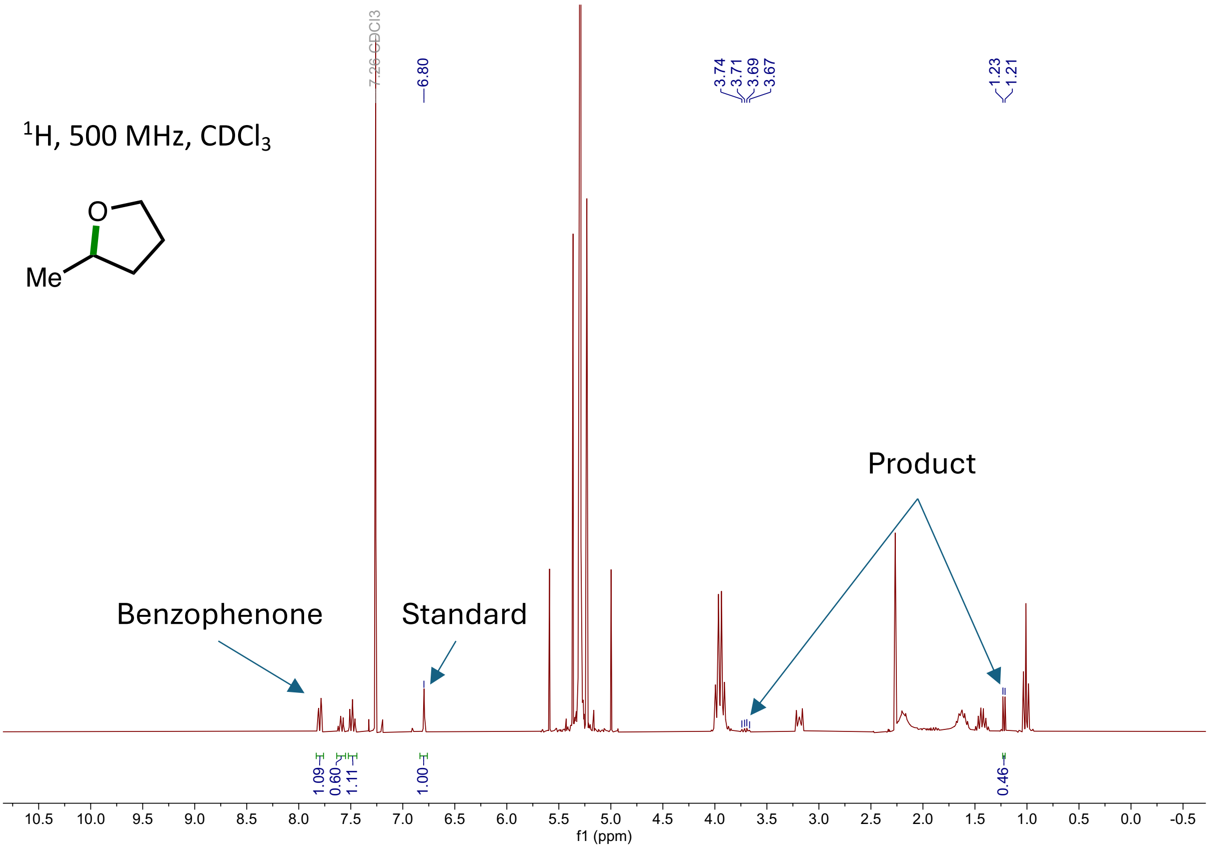

Data consistent with commercially secured 2-Methyltetrahydrofuran.

Selected data for commercially secured 2-Methyltetrahydrofuran:

^{1}H NMR (300 MHz, CDCl$_3$) δ 3.96 – 3.82 (m, 2H), 3.71 – 3.63 (m, 1H), 1.98 – 1.82 (m, 3H), 1.41 – 1.34 (m, 1H), 1.21 – 1.19 (d, *J* = 6.1 Hz, 3H).

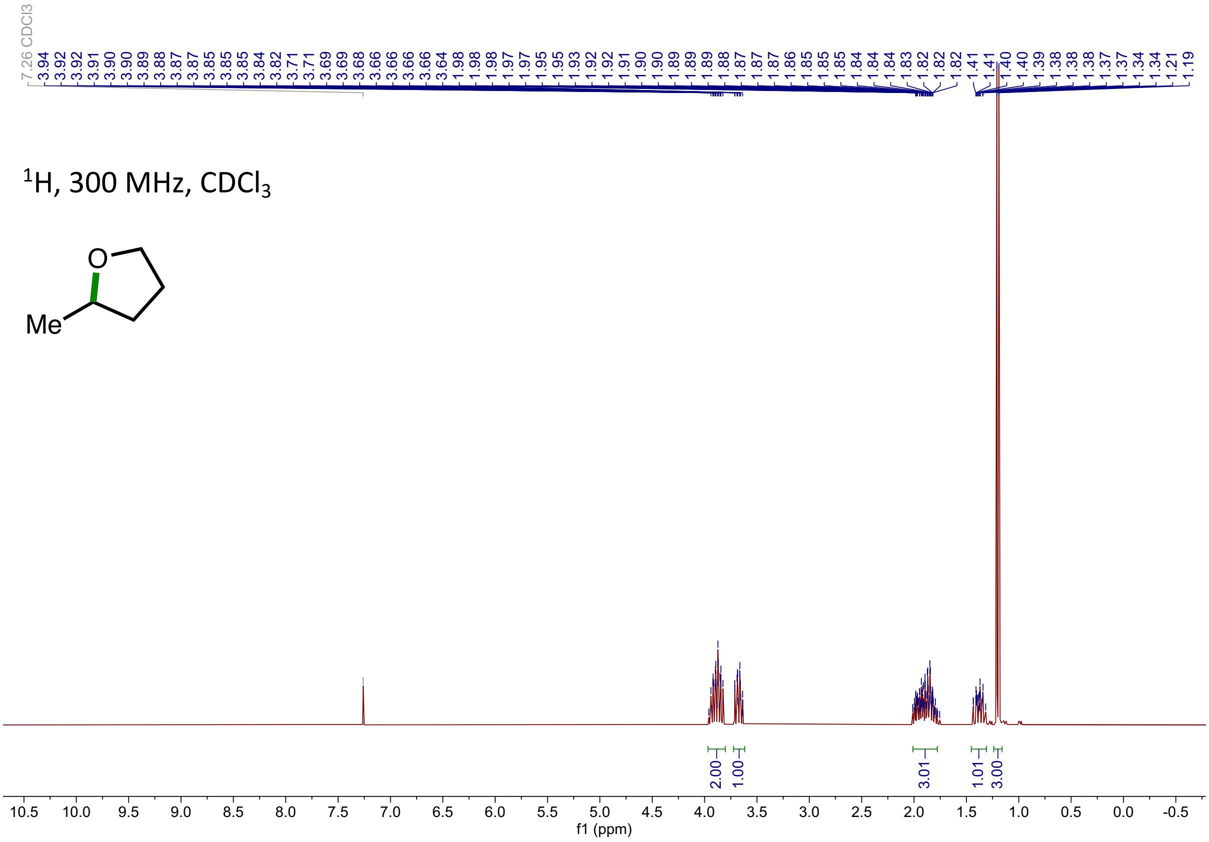

(P32)

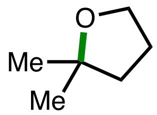

Prepared according to electrochemical general procedure using **S32** (85.4 mg, 0.30 mmol, 1 equiv.), *n*-Bu_4ClO_4 (103 mg, 0.30 mmol, 1 equiv.), dichloromethane (5.7 mL) and TFE (0.3 mL). The yield was determined by crude ^{1}H NMR using 1,3,5-trimethylbenzene (42 µL, 1 equiv.) as an internal standard: 36%. Benzophenone was also observed: 73% on NMR.

$$\% \text{ Yield of the Product} = \left(\frac{0.24}{1.00}\right) \times \left(\frac{3.00}{2.00}\right) \times 100 = 36\%$$

Selected data for the product:

^{1}H NMR (300 MHz, $CDCl_3$) δ 3.85 – 3.80 (t, *J* = 6.8 Hz, 2H), 1.96 – 1.91 (m, 2H).

Data consistent with the literature.[23]

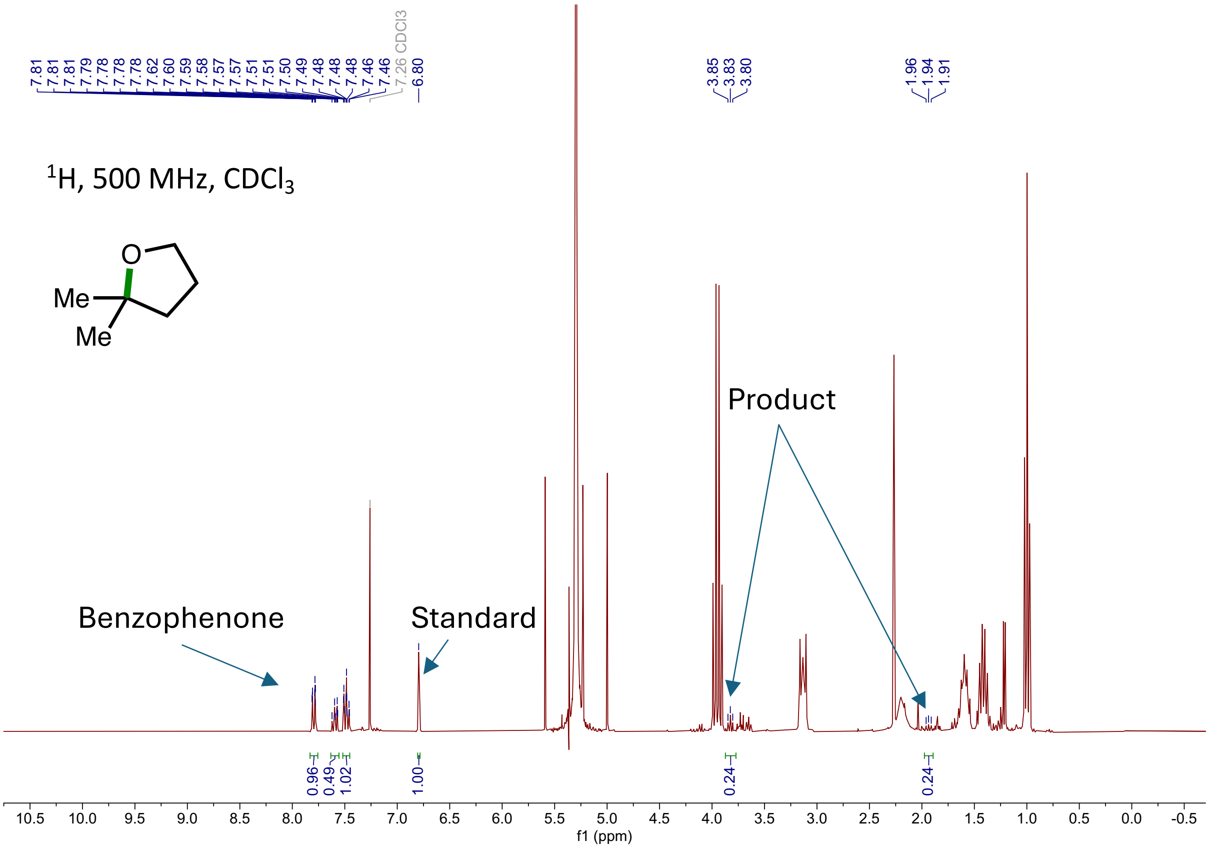

Electrochemical Flow Scale Up

General Information

The flow set-up used PFA tubing with a 0.79 ± 0.1 mm internal diameter and 1.58±0.1 mm outer diameter supplied by Polyflon. All flow fittings and connections were purchased from Kinesis (Gripper fitting nuts, part number: 002103; Adapters, part number: P-618; Omnilok type-p fitting ferrule, part number: 008FT16; Y-Connector, part number: P-512; Threaded union, part number: P-623. The syringe pumps used was the the Chemyx Fusion 100 syringe pump.

The power supply used was a Voltcraft LRP-1205 that supplied DC to the electrochemical system. The electrochemical flow cell was purchased from Cambridge Reactor Design, the Ammonite 8 (part number: 74660). The electrochemical flow cell consists of 1 carbon/PVDF electrode and one platinum electrode that are fixed either side of a FFKM gasket with a channel groove length of 1000 mm and an internal volume of 2.5 mL, of which 1 mL is exposed to the electrodes. The inlet and outlet fittings of the ammonite 8 cell were modified from 1/16” ID to accommodate 1/32” ID PFA tubing using Swagelok reducers, nuts and ferrules. The ammonite reactor was cell was dismantled for electrode cleaning every 2 passes. The graphite electrode was cleaned with acetone, water, acetone rinses and then the surface regenerated by rubbing with silica gel and cotton wool to remove surface contaminants. The platinum electrode was cleaned with acetone, water acetone rinses, left to dry and then burned with a blow torch.

The electrochemical flow system was used only in single pass electrolysis. Recirculating flow was not examined.

Gram Scale Flow Experimental

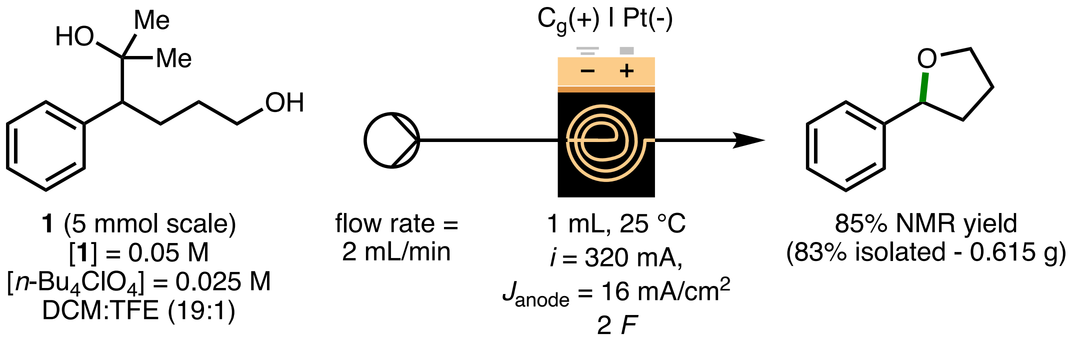

A 250 mL round-bottom flask was flame dried and charged with 5-methyl-4-phenylhexane-1,5-diol **1** (1.146 g, 5.5 mmol, 1 equiv.) and n-Bu_4ClO_4 (940 mg, 2.75 mmol, 0.025 M) and sealed with a Suba-seal. The reagent flask was evacuated on a Schlenk line and back filled with nitrogen gas for three cycles. Dry CH_2Cl_2 (104.5 mL) and TFE (5.5 mL) were then added. After deoxygenating, (bubbling with nitrogen for 5 min) the mixture was drawn up into two 60 mL syringes (2 x 55 mL) and loaded onto a syringe pump.

The ammonite8 electrochemical flow reactor set up with a graphite anode and platinum cathode, was flushed with nitrogen gas for 5 min by connecting the inlet tubing directly to a dry nitrogen line. The reagent solution in 2 x 60 mL syringes were then connected to the flow set up via a T-piece. The syringe pumps were set to 1 mL/min, giving a combined total flow rate of 2 mL/min at the mixing-tee. The electrochemical set up was primed by pulling through 2 mL of reaction mixture to fill the volume of the flow reactor and tubing. The power supply was set (320 mA, constant current) and attached to the ammonite 8 flow reactor. The power supply was switched on and syringe pumping was initiated, and the outlet of the flow system was set to waste for the first 3 mL (representing 1 whole flow path volume, inclusive of tubing, connectors and reactor) of reaction mixture to allow the flow system to be filled. The outlet stream of the flow system was then collected for 100 mL (representing 5.0 mmol of material processed) at an operating potential of 5.7-5.8 V. Mesitylene (348 µL, 0.5 equiv.) was added to the product mixture, stirred for 5 min and then the ^{1}H NMR spectrum was recorded to give a crude reaction yield of 85%.

The reaction mixture was then concentrated *in vacuo*. The crude residue was purified by flash column chromatography (eluent = 5 to 15% EtOAc in hexanes, silica gel) to yield pure product as a colourless oil (0.615 g, 83% yield).

$$\% \text{ NMR Yield of the Products} = \left(\frac{\text{Compound Integral}}{\text{Standard Integral}}\right) \times \left(\frac{\text{Standard Proton}}{\text{Compound Proton}}\right) \times 100$$

$$\% \text{ Yield of the Product} = \left(\frac{0.57}{1.00}\right) \times \left(\frac{1.50\ (0.50\ \text{equiv.})}{1.00\ (1.00\ \text{equiv.})}\right) \times 100 = 85\%$$

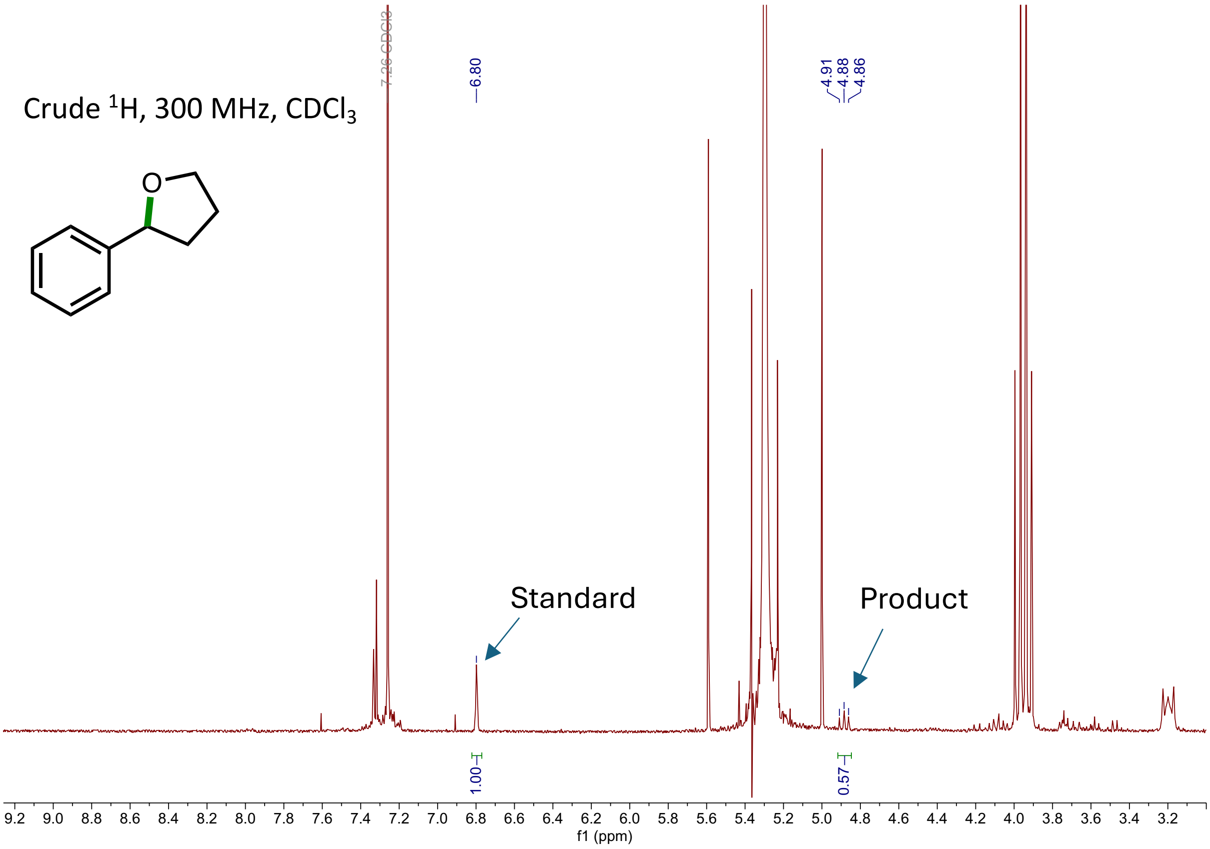

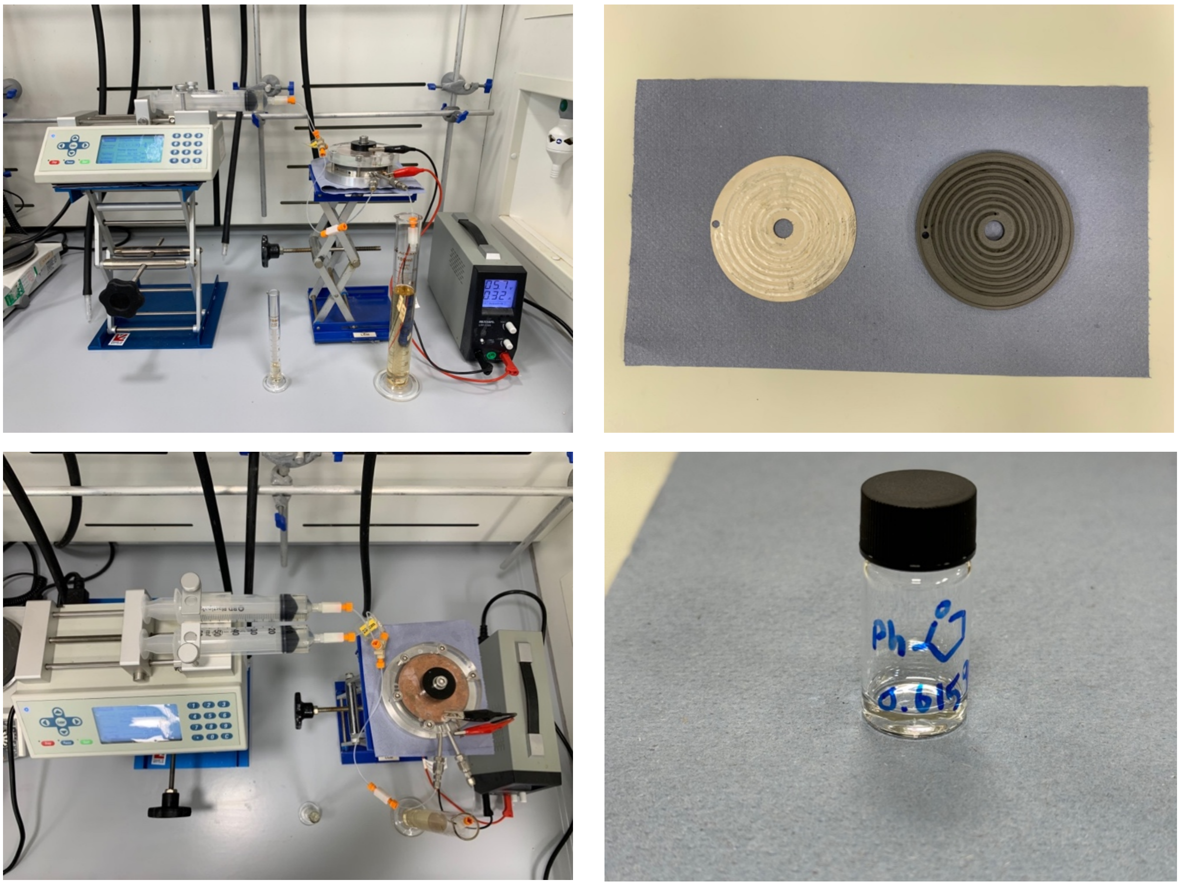

Figure S5. *Electrochemical flow set up: Ammonite8 Reactor attached to a syringe pump and potentiostat (top left and bottom left). Prepared graphite and platinum electrodes (top right). Isolated product - 0.615 g (bottom right).*

Cyclic Voltammetry Studies of Selected Substrates

Oxidative Potential Window (0.0 – 2.5 V vs. Fc/Fc^+); IUPAC convention.

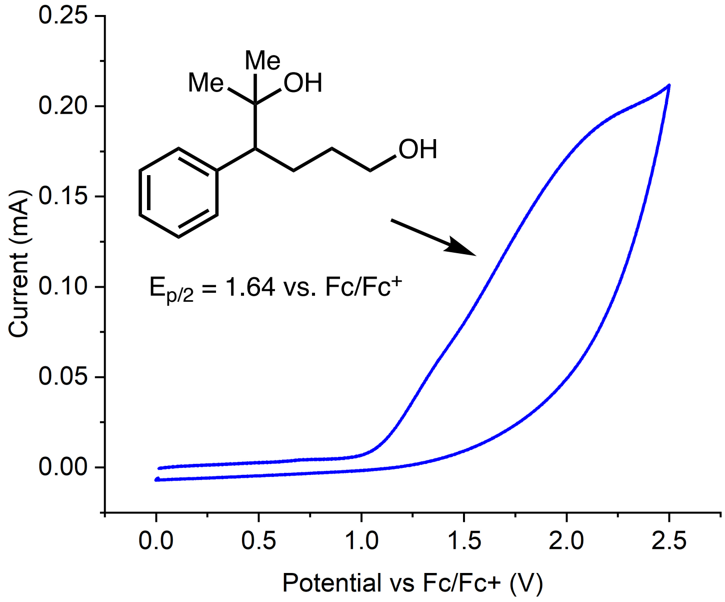

Figure S6. *CV of Compound **1** (5 mM in DCM), n-Bu_4PF_6 (0.1 M), Scan Rate: 100 mV/s*

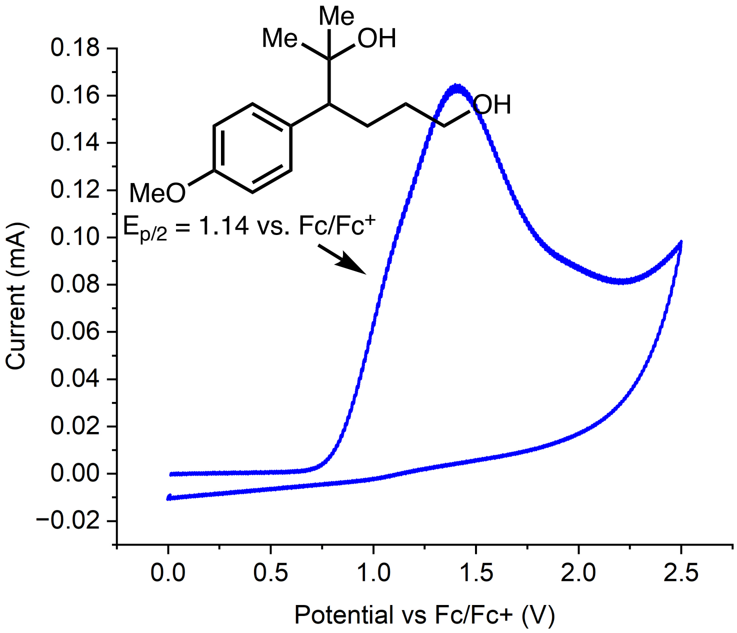

Figure S7. *CV of Compound **S8** (5 mM in DCM), n-Bu_4PF_6 (0.1 M), Scan Rate: 100 mV/s*

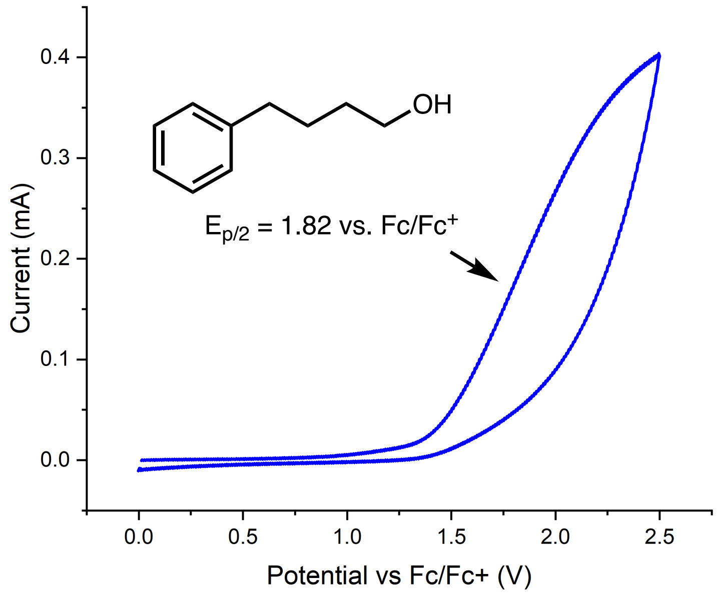

Figure S8. *CV of Compound **S38** (5 mM in DCM), n-Bu$_4$PF$_6$ (0.1 M), Scan Rate: 100 mV/s*

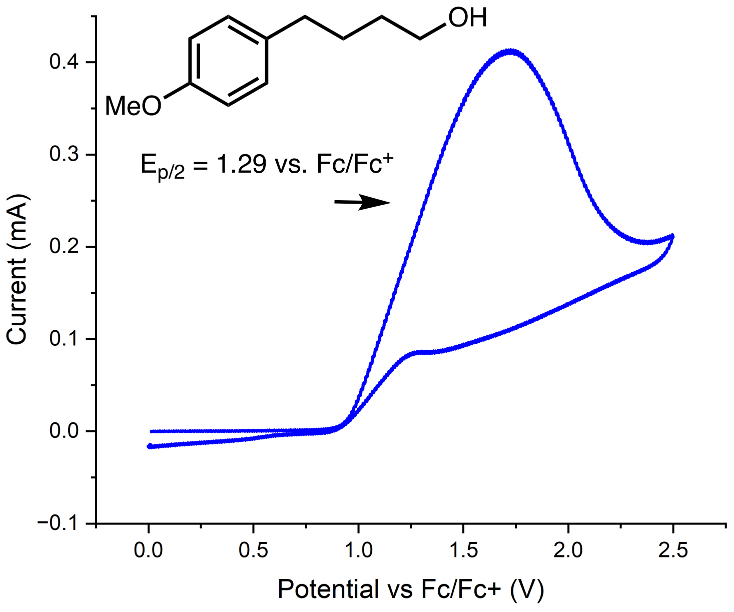

Figure S9. *CV of Compound **S40** (5 mM in DCM), n-Bu$_4$PF$_6$ (0.1 M), Scan Rate: 100 mV/s*

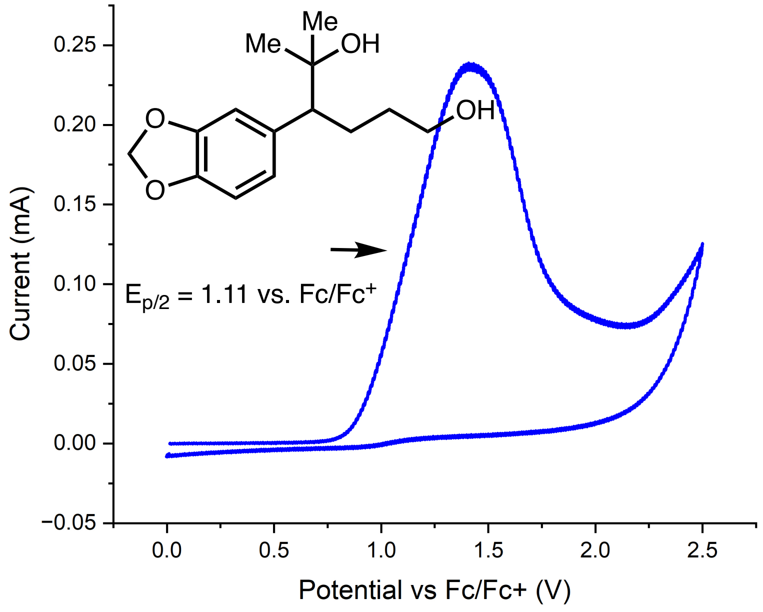

Figure S10. *CV of Compound **S20** (5 mM in DCM), n-Bu_4PF_6 (0.1 M), Scan Rate: 100 mV/s*

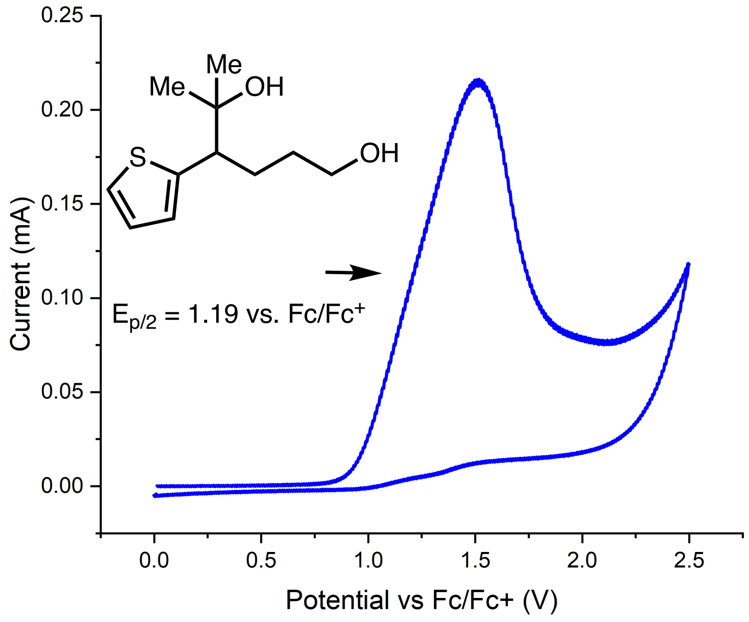

Figure S11. *CV of Compound **S21** (5 mM in DCM), n-Bu_4PF_6 (0.1 M), Scan Rate: 100 mV/s*

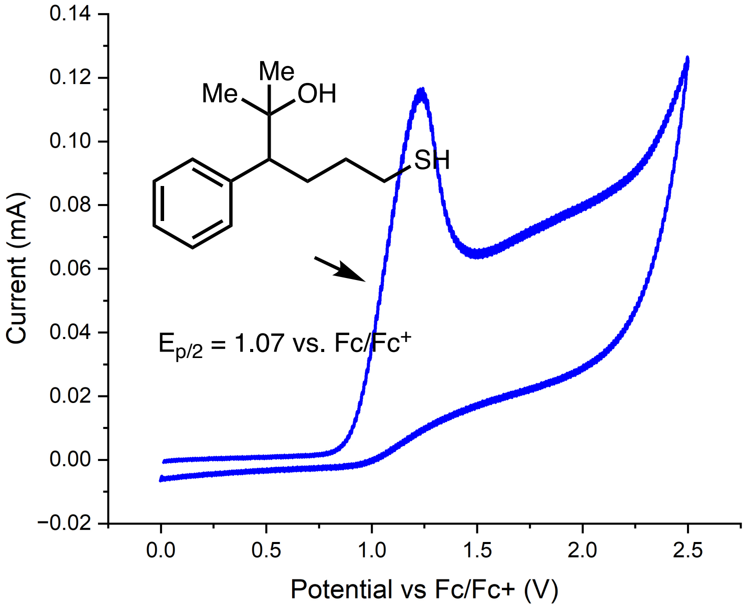

Figure S12. *CV of Compound **S30** (5 mM in DCM), n-Bu$_4$PF$_6$ (0.1 M), Scan Rate: 100 mV/s*

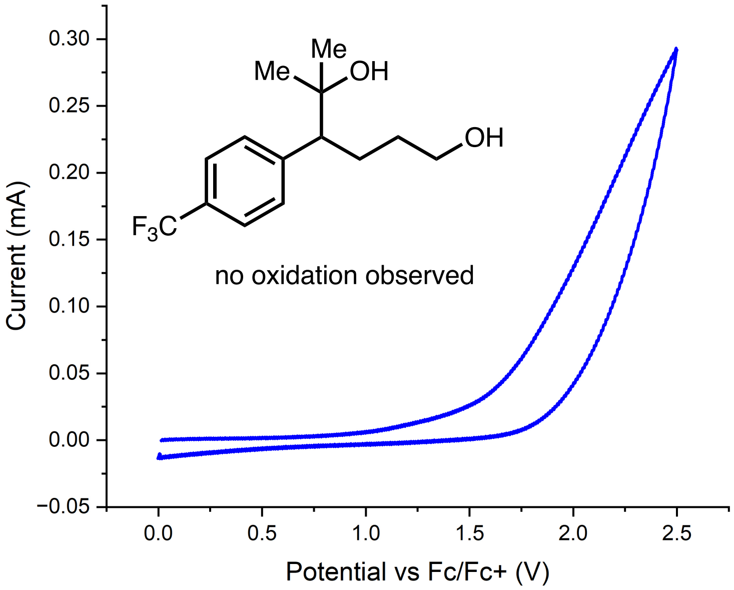

Figure S13. *CV of Compound **S12** (5 mM in DCM), n-Bu$_4$PF$_6$ (0.1 M), Scan Rate: 100 mV/s*

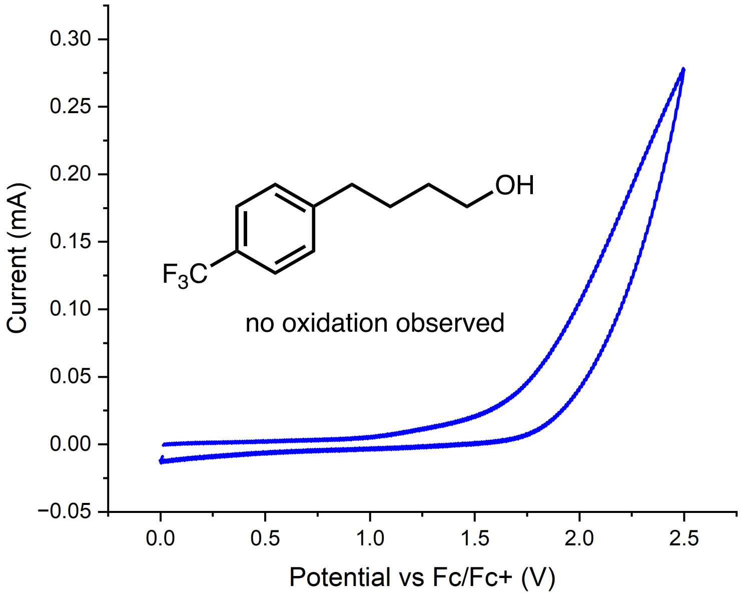

Figure S14. *CV of Compound **S41** (5 mM in DCM), n-Bu_4PF_6 (0.1 M), Scan Rate: 100 mV/s*

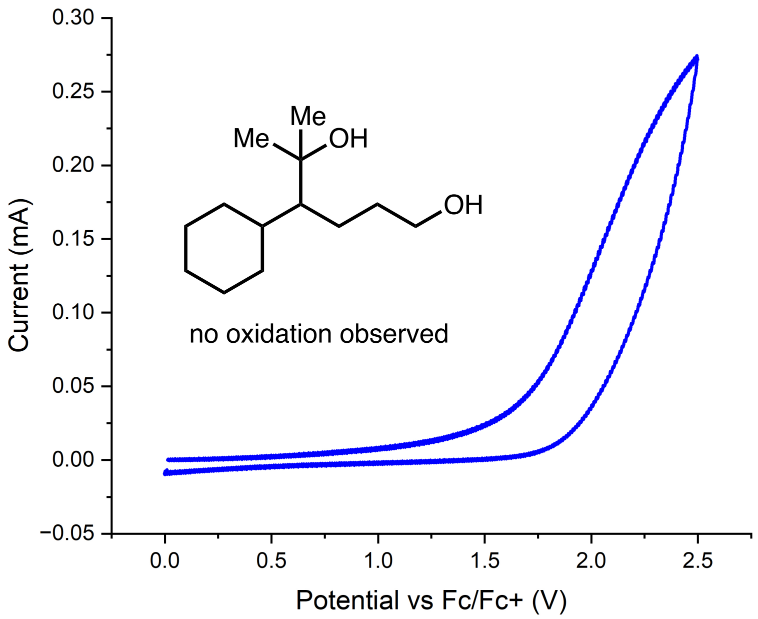

Proposed Mechanism

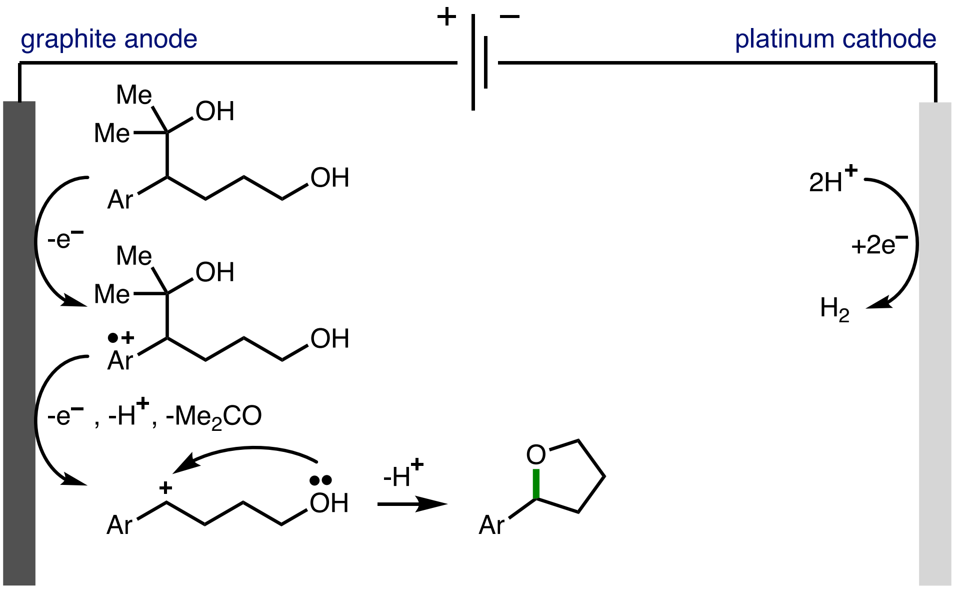

References

(1) Stivala, C. E.; Zakarian, A. Highly Enantioselective Direct Alkylation of Arylacetic Acids with Chiral Lithium Amides as Traceless Auxiliaries. *J Am Chem Soc* **2011**, *133* (31), 11936–11939. https://doi.org/10.1021/JA205107X/SUPPL_FILE/JA205107X_SI_002.PDF.

(2) Alliot, J.; Gravel, E.; Pillon, F.; Buisson, D. A.; Nicolas, M.; Doris, E. Enantioselective Synthesis of Levomilnacipran. *Chemical Communications* **2012**, *48* (65), 8111–8113. https://doi.org/10.1039/C2CC33743F.

(3) Yang, X. H.; Yue, H. T.; Yu, N.; Li, Y. P.; Xie, J. H.; Zhou, Q. L. Iridium-Catalyzed Asymmetric Hydrogenation of Racemic α-Substituted Lactones to Chiral Diols. *Chem Sci* **2017**, *8* (3), 1811–1814. https://doi.org/10.1039/C6SC04609F.

(4) De Filippis, A.; Gomez Pardo, D.; Cossy, J. Palladium-Catalyzed α-Arylation of N-Protected 2-Piperidinones. *Tetrahedron* **2004**, *60* (43), 9757–9767. https://doi.org/10.1016/J.TET.2004.06.152.

(5) Almena, J.; Foubelo, F.; Yus, M. Reductive Opening of Phenyl Substituted Thiacycloalkanes: New Way for Sulphur-Containing Organolithium Compounds. *Tetrahedron* **1997**, *53* (15), 5563–5572. https://doi.org/10.1016/S0040-4020(97)00211-1.

(6) Fanourakis, A.; Williams, B. D.; Paterson, K. J.; Phipps, R. J. Enantioselective Intermolecular C-H Amination Directed by a Chiral Cation. *J Am Chem Soc* **2021**, *143* (27), 10070–10076. https://doi.org/10.1021/JACS.1C05206/ASSET/IMAGES/LARGE/JA1C05206_0005.JPEG.

(7) Nuzzi, A.; Fiasella, A.; Ortega, J. A.; Pagliuca, C.; Ponzano, S.; Pizzirani, D.; Bertozzi, S. M.; Ottonello, G.; Tarozzo, G.; Reggiani, A.; Bandiera, T.; Bertozzi, F.; Piomelli, D. Potent α-Amino-β-Lactam Carbamic Acid Ester as NAAA Inhibitors. Synthesis and Structure–Activity Relationship (SAR) Studies. *Eur J Med Chem* **2016**, *111*, 138–159. https://doi.org/10.1016/J.EJMECH.2016.01.046.

(8) Li, L.; Li, Z.; Sun, W.; Li, C. A Convergent Paired Electrochemical Strategy for Decarboxylative C(Sp2)–C(Sp3) Bond Formation. *Organic Chemistry Frontiers* **2024**, *11* (7), 1941–1948. https://doi.org/10.1039/D3QO02141F.

(9) Gong, Y.; Su, L.; Zhu, Z.; Ye, Y.; Gong, H. Nickel-Catalyzed Thermal Redox Functionalization of C(Sp3)−H Bonds with Carbon Electrophiles**. *Angewandte Chemie International Edition* **2022**, *61* (22), e202201662. https://doi.org/10.1002/ANIE.202201662.

(10) Lindroth, R.; Ondrejková, A.; Wallentin, C. J. Visible-Light Mediated Oxidative Fragmentation of Ethers and Acetals by Means of Fe(III) Catalysis. *Org Lett* **2022**, *24* (8), 1662–1667. https://doi.org/10.1021/ACS.ORGLETT.2C00231/ASSET/IMAGES/LARGE/OL2C00231_0006.JPEG.

(11) Koch, V.; Bräse, S. An Intramolecular Iodine-Catalyzed C(Sp3)−H Oxidation as a Versatile Tool for the Synthesis of Tetrahydrofurans. *European J Org Chem* **2021**, *2021* (24), 3478–3483. https://doi.org/10.1002/EJOC.202100652.

(12) Dilauro, G.; Cicco, L.; Vitale, P.; Perna, F. M.; Capriati, V. Ligand-Free Pd-Catalyzed Reductive Mizoroki-Heck Reaction Strategy for the One-Pot Synthesis of Functionalized Oxygen Heterocycles in Deep Eutectic Solvents. *European J Org Chem* **2023**, *26* (3), e202200814. https://doi.org/10.1002/EJOC.202200814.

(13) Li, W.; Yang, C.; Gao, G. L.; Xia, W. Visible-Light-Induced Cyclization of Electron-Enriched Phenyl Benzyl Sulfides: Synthesis of Tetrahydrofurans and Tetrahydropyrans. *Synlett* **2016**, *27* (9), 1391–1396. https://doi.org/10.1055/S-0035-1561393/ID/JR000-1002/BIB.

(14) Cong, F.; Lv, X. Y.; Day, C. S.; Martin, R. Dual Catalytic Strategy for Forging Sp2-Sp3and Sp3-Sp3Architectures via β -Scission of Aliphatic Alcohol Derivatives. *J Am Chem Soc* **2020**, *142* (49), 20594–20599. https://doi.org/10.1021/JACS.0C11172/SUPPL_FILE/JA0C11172_SI_002.PDF.

(15) Shields, B. J.; Doyle, A. G. Direct C(Sp3)-H Cross Coupling Enabled by Catalytic Generation of Chlorine Radicals. *J Am Chem Soc* **2016**, *138* (39), 12719–12722. https://doi.org/10.1021/JACS.6B08397/ASSET/IMAGES/LARGE/JA-2016-08397X_0009.JPEG.

(16) Huy, P. H.; Koskinen, A. M. P. Efficient, Stereodivergent Access to 3-Piperidinols by Traceless P(OEt)3 Cyclodehydration. *Org Lett* **2013**, *15* (20), 5178–5181. https://doi.org/10.1021/OL4026588/SUPPL_FILE/OL4026588_SI_001.PDF.

(17) Singh, P. P.; Gudup, S.; Aruri, H.; Singh, U.; Ambala, S.; Yadav, M.; Sawant, S. D.; Vishwakarma, R. A. Organic & Biomolecular Chemistry New Method for C-H Arylation/Alkylation at α-Position of Cyclic Aliphatic Ethers by Iron-Oxide Mediated Reaction †. *Org. Biomol. Chem* **2012**, *10*, 1587. https://doi.org/10.1039/c1ob06660a.

(18) Dhiman, S.; Ramasastry, S. S. V. Organic & Biomolecular Chemistry Taming Furfuryl Cations for the Synthesis of Privileged Structures and Novel Scaffolds † ‡. *Org. Biomol. Chem* **2013**, *11*, 4299. https://doi.org/10.1039/c3ob40814k.

(19) Hayashi, N.; Shibata, I.; Baba, A. Inter- and Intramolecular Radical Couplings of Ene-Ynes or Halo-Alkenes Promoted by an InCl3/MeONa/Ph2SiH2 System. *Org Lett* **2005**, *7* (14), 3093–3096. https://doi.org/10.1021/OL051114O/SUPPL_FILE/OL051114OSI20050603_101523.PDF.

(20) Fujita, S.; Abe, M.; Shibuya, M.; Yamamoto, Y. Intramolecular Hydroalkoxylation of Unactivated Alkenes Using Silane-Iodine Catalytic System. *Org Lett* **2015**, *17* (15), 3822–3825. https://doi.org/10.1021/ACS.ORGLETT.5B01797/SUPPL_FILE/OL5B01797_SI_001.PDF.

(21) Veselý, O.; Živný, M.; Nigríni, M.; Veselý, J.; Čejka, J. The Effect of the Active Site and Substrate Structure in Preparation of Substituted Tetrahydropyrans via Intramolecular Cyclization. *Catal Today* **2024**, *429*, 114465. https://doi.org/10.1016/J.CATTOD.2023.114465.

(22) Ye, W.; Xiong, H.; Wang, M.; Chang, J.; Yu, W. Iodine-Mediated δ-Amination of Sp3 C-H Bonds. *Journal of Organic Chemistry* **2024**, *89* (5), 3481–3490. https://doi.org/10.1021/ACS.JOC.3C02901/SUPPL_FILE/JO3C02901_SI_001.PDF.

(23) Cicco, L.; Sblendorio, S.; Mansueto, R.; Perna, F. M.; Salomone, A.; Florio, S.; Capriati, V. Water Opens the Door to Organolithiums and Grignard Reagents: Exploring and Comparing the Reactivity of Highly Polar Organometallic Compounds in Unconventional Reaction Media towards the Synthesis of Tetrahydrofurans. *Chem Sci* **2016**, *7* (2), 1192–1199. https://doi.org/10.1039/C5SC03436A.
